# Direct allylic acylation via cross-coupling involving cooperative *N*‑heterocyclic carbene, hydrogen atom transfer, and photoredox catalysis

**DOI:** 10.1038/s41467-023-38743-8

**Published:** 2023-05-23

**Authors:** Xiaochen Wang, Rongxin Yang, Binbing Zhu, Yuxiu Liu, Hongjian Song, Jianyang Dong, Qingmin Wang

**Affiliations:** State Key Laboratory of Elemento-Organic Chemistry, Research Institute of Elemento-Organic Chemistry, Frontiers Science Center for New Organic Matter, College of Chemistry, Nankai University, 300071 Tianjin, China

**Keywords:** Catalyst synthesis, Reaction mechanisms, Synthetic chemistry methodology

## Abstract

Herein, we report a mild, operationally simple, multicatalytic method for the synthesis of *β,γ*-unsaturated ketones via allylic acylation of alkenes. Specifically, the method combines *N*‑heterocyclic carbene catalysis, hydrogen atom transfer catalysis, and photoredox catalysis for cross-coupling reactions between a wide range of feedstock carboxylic acids and readily available olefins to afford structurally diverse *β,γ*-unsaturated ketones without olefin transposition. The method could be used to install acyl groups on highly functionalized natural-product-derived compounds with no need for substrate pre-activation, and C–H functionalization proceed with excellent site selectivity. To demonstrate the potential applications of the method, we convert a representative coupling product into various useful olefin synthons.

## Abstract (web summary)

The versatility and importance of *β,γ*-unsaturated ketones has been appreciated for a long time in synthesis and medicinal chemistry. Here, the authors use carboxylic acids and readily available olefins as substrates for transformations involving *N*‑heterocyclic carbene catalysis, hydrogen atom transfer catalysis, and photoredox catalysis for the synthesis of β,γ-unsaturated ketones.

## Introduction

**Fig. 1:**
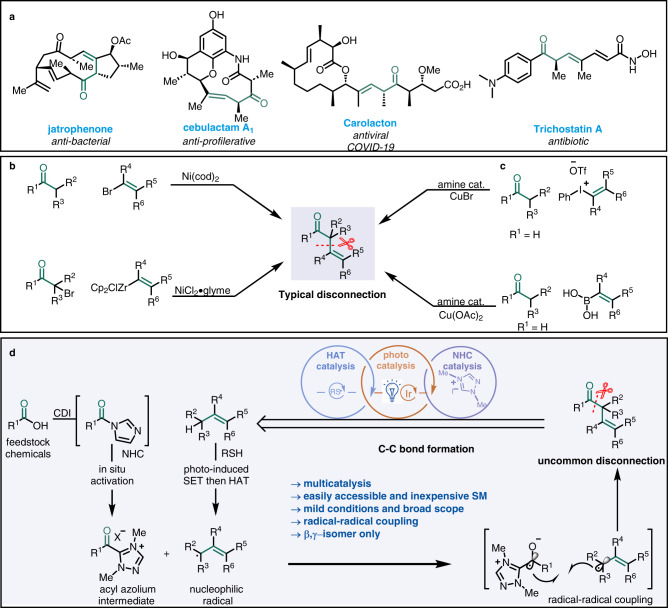
Bioactive compounds with *β,γ*-unsaturated ketone motifs and approaches for their synthesis. **a** Examples of pharmaceutically active agents possessing *β,γ*-unsaturated ketone motifs. **b**, **c** Classic catalytic approaches for *β,γ*-unsaturated ketone synthesis. **d** Strategy used in this study.

*α, β*-Unsaturated ketones are common structures in functional organic molecules and are easy to synthesize. In contrast, robust synthetic methods for *β,γ*-unsaturated ketones are lacking, despite the fact that these moieties are found in many bioactive molecules and natural products and can be used as building blocks for complex structures (Fig. 1a)^1–6^. Many of the reported methods are based on disconnection of the bond between the *α* and *β* carbons which means *α*-alkenylation of an enolate or enolate equivalent;^7–12^ whereas disconnection of the bond between the carbonyl group and the *α* carbon—that is, allylation of an acyl donor^13–17^—has not been thoroughly explored. In addition, most methods for synthesizing *β,γ*-unsaturated ketones require a prefunctionalized starting material, which limits the applications of the methods to relatively simple targets. Moreover, these methods suffer from low *β,γ* regioselectivity^18,19^. To address these issues, investigators have recently developed a number of mild catalytic reactions. For example, the Helquist group reported a procedure for Ni-catalyzed cross-coupling of ketone enolates with allyl bromides (Fig. 1b, top)^20^, Fu and a co-worker reported Ni-catalyzed enantioselective cross-coupling of secondary *α*-bromoketones with vinyl zirconium reagents (Fig. 1b, bottom)^21^, and MacMillan and colleagues realized enantioselective *α*-vinylation of aldehydes with catalysis by Cu(I) and chiral amines (Fig. 1c)^22–24^. However, these elegant methods are based on disconnection of the *α,β* bond, which has hampered their wider applications. Only a few examples of retrosynthetic analyses based on radical reaction of homolytic disconnection of the bond between the *α* carbon and the carbonyl carbon have been reported^25,26^. Given that *β,γ*-unsaturated ketones are privileged scaffolds, their synthesis remains an important challenge, and there is a growing need to blossom new C–H bond activation and late-stage functionalization reactions in an unconventional manner.

We reasoned that combining *N*-heterocyclic carbine (NHC) catalysis with other types of catalysis might be useful for *β,γ*-unsaturated ketone synthesis. NHCs are unique Lewis basic catalysts that use polarity reversal to mediate various organic transformations^27–29^. The revival of photocatalysis^30–36^ and electrocatalysis has accelerated the development of free radical chemistry^37–46^, and visible-light catalysis has been combined with NHC catalysis to achieve NHC-mediated radical reactions under mild conditions. For example, reactions of radicals generated by single-electron oxidation of Breslow intermediates have been reported by the groups of Scheidt^47,48^, Studer^49,50^, Chi^51–55^, Rovis^56,57^, Sun^58^, Ye^59–62^, Ohmiya^63–66^, and others^67–74^. In contrast, single-electron reduction of NHC-bound acyl azolium intermediates in radical reactions has not been as thoroughly explored^75–84^. In above processes, the NHC catalytic cycle is intertwined with the second catalytic cycle, and biosyntheses of complex natural products often proceed via multicatalytic pathways, combining three or more catalysts is an attractive strategy for the development of new reactions^85,86^. However, the use of NHCs in multicatalytic systems is in its budding stage^87^, and its challenge lies in compatibility issues between catalysts and intermediates, so we aim to use the unique single-electron reduction characteristics of acyl azolium intermediates with the combination of photocatalysis and hydrogen atom transfer catalysis to further bridge the existing gaps in this field. Aromatic carboxylic acids were chosen as substrates because they are inexpensive, readily available, highly reactive, can be used to quickly generate libraries of structurally complex small molecules^88,89^.

In this work, we disclose a strategy for coordinating triple catalysis to direct allylic acylation of alkenes with carboxylic acids (Fig. 1d). Specifically, a carboxylic acid would be activated in situ by *N,N*-carbonyldiimidazole (CDI), and then the addition of a NHC would afford an acyl azolium intermediate^90^, which would undergo single-electron reduction upon irradiation with visible light in the presence of a photocatalyst to generated an azolium radical anion. Meanwhile, single-electron oxidation of a thiolate generated in situ would provide a thiyl radical, which would undergo a HAT reaction with the olefin to produce a nucleophilic allylic radical, and coupling of this radical with the azolium radical anion would form the *β,γ*-unsaturated ketone. The key challenge posed by this strategy was the identification of three highly selective but independent catalysts that functioned harmoniously. Several formidable challenges comprise (1) The HAT catalyst would have to discriminate between the reactants and the products. (2) Thiyl radical would selectively undergo a HAT reaction rather than direct coupling with the azolium radical anion. (3) The formation of *α,β*-isomers as by-products would have to be avoided. (4) Because the redox activity of the three catalysts would be interdependent, regeneration of the ground-state photocatalyst, the NHC catalyst, and the HAT catalyst would require careful synchronization of the three catalytic cycles.

## Results

### Optimization of reaction conditions

We commenced our investigation by using **1a**, an acyl imidazole derivative of *p*-toluic acid, and cyclohexene (**2a**) as model substrates (Table 1). After extensive exploration of the reaction parameters, we found that upon irradiation of **1a** and **2a** in DCM with blue LEDs in the presence of PC-I as the photocatalyst, NHC **A** as the NHC catalyst, triisopropylsilanethiol (HAT-**1**) as the HAT catalyst, and potassium phosphate and cesium carbonate as bases (20 mol % each), we could obtain target *β,γ*-unsaturated ketone product **3** in 77% isolated yield (entry 1). When other photocatalysts were used, the yield of **3** decreased substantially (entries 2–4). Replacing NHC **A** with other catalysts led to sharply lower yields (entries 5–9). Evaluation of solvent effects revealed that CH_3_CN and THF were inferior to DCM (entries 10 and 11). Under the same conditions, other thiol catalysts HAT-**2**–HAT-**5** gave considerably lower yields (entry 12). This result is mainly due to the mismatch of BDE values (e.g., methyl thiosalicylate S−H BDE = 78.7 kcal/mol; cyclohexene allylic C−H BDE = 83.2 kcal/mol and toluene benzylic C−H BDE = 89.9 kcal/mol) and other on long-standing and well-established physical properties (that is, oxidation potentials, hydrogen atom transfer exchange constants)^91–94^. Replacing K_3_PO_4_ or Cs_2_CO_3_ with other bases substantially decreased the yields (entries 13–17). Control experiments showed that all three catalysts, both bases, and light were crucial to the reaction (entries 18–22).

**Table 1:** Optimization of reaction conditions^a^


Entry	Deviation from standard conditions	Yield (%)^b^
1	None	83 (77^c^)
2	PC-II instead of PC-I	29
3	PC-III instead of PC-I	31
4	PC-IV instead of PC-I	NR
5	NHC B instead of NHC A	NR
6	NHC C instead of NHC A	<5
7	NHC D instead of NHC A	45
8	NHC E instead of NHC A	NR
9	NHC F instead of NHC A	<5
10	MeCN instead of DCM	27
11	THF instead of DCM	NR
12	HAT-2–HAT-5	0–31
13	^*n*^Bu_4_NOAc instead of K_3_PO_4_	36
14	Cs_2_CO_3_ instead of K_3_PO_4_	27
15	DBU instead of K_3_PO_4_	19
16	NaHCO_3_ instead of Cs_2_CO_3_	30
17	K_3_PO_4_ instead of Cs_2_CO_3_	27
18	No PC	NR
19	No NHC	NR
20	No base	NR
21	No HAT	NR
22	No light	NR

NR, no reaction.

^a^Standard conditions: **1a** (0.3 mmol), **2a** (0.6 mmol), NHC A catalyst (0.06 mmol), photocatalyst (0.003 mmol), Cs_2_CO_3_ (0.06 mmol), K_3_PO_4_ (0.06 mmol), and HAT-1 catalyst (0.06 mmol) in DCM (4 mL) were irradiated with blue LEDs under Ar at room temperature.

^b^Determined by 1H NMR spectroscopy with dibromomethane as an internal standard.

^c^Isolated yield.

### Substrate scope with respect to the carboxylic acid

**Fig. 2:**
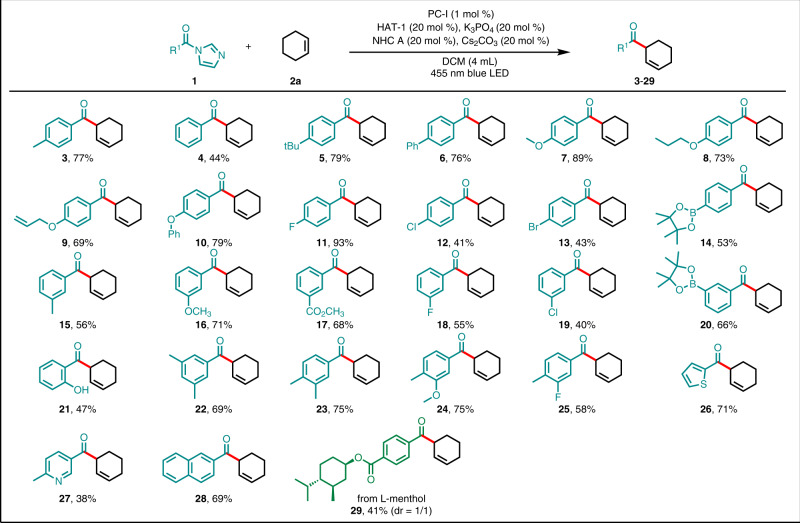
Scope of the reaction with respect to the carboxylic acid. Reaction conditions: **1** (0.3 mmol), **2a** (0.6 mmol), NHC A catalyst (0.06 mmol), PC-I (0.003 mmol), Cs_2_CO_3_ (0.06 mmol), K_3_PO_4_ (0.06 mmol), and HAT-1 (0.06 mmol) in DCM (4 mL) were irradiated with a 36 W blue LED under Ar at room temperature, isolated yields were given.

After optimizing the reaction conditions, we investigated the suitability of acyl imidazole derivatives of various aryl carboxylic acids (Fig. 2). In reactions with **2a**, acyl imidazoles **1** with a substituent on the meta or para position of the aryl ring were tolerated, affording the corresponding *β,γ*-unsaturated ketone products (**4**–**20**) in moderate to high yields, regardless of the electronic nature of the substituent. Moreover, a product with an unprotected ortho hydroxyl group (**21**) could be obtained, albeit in moderate yield. Disubstituted substrates were also amenable to the reaction conditions, affording compounds **22**–**25**. Notably, halogen atoms remained intact, and thus products **11**–**13,** **18,** **19**, and **25** had the potential to undergo subsequent functionalization. The mild conditions were compatible with a range of functional groups, including phenyl (**6**), ethers (**7**–**10,** **16** and **24**), esters (**17**), and alkyl-substituted phenyl rings (**5,** **15,** **22** and **23**). Moreover, several relatively sensitive but versatile functional groups—an olefin (**9**) and boronic esters (**14** and **20**)–also tolerated the reaction conditions well, indicating the potential utility of this method for pharmaceutical and synthetic chemistry. Notably, we did not detect a reaction at the allylic C − H bonds position of substrate of **9**. Because of the prevalence of heteroaryl groups in pharmaceutical compounds, we were pleased to find that thiophene and pyridine substrates were amenable to the reaction, yielding **26** and **27** in 71% and 38% yields, respectively. In addition, 2-naphthoic acid underwent the desired transformation to afford **28** in 69% yield. Finally, late-stage modification of a derivative of menthol, delivered the corresponding product **29** in 41% yield. Potentially because of the instability of aliphatic azolium radicals, the scope was primarily limited to aryl substrates.

### Substrate scope with respect to the alkene

**Fig. 3:**
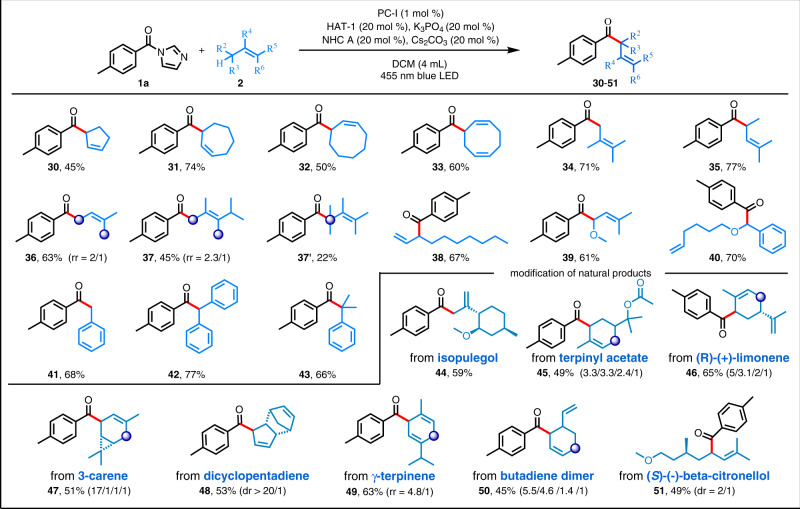
Reactions of allylic/benzylic C(sp^3^)–H substrates. Reaction conditions: **1a** (0.3 mmol), **2** (0.6 mmol), NHC A catalyst (0.06 mmol), PC-I (0.003 mmol), Cs_2_CO_3_ (0.06 mmol), K_3_PO_4_ (0.06 mmol), and HAT-1 (0.06 mmol) in DCM (4 mL) were irradiated with a 36 W blue LED under Ar at room temperature, isolated yields were given.

The reaction conditions could also be applied to alkenes **2** with a diverse array of substitution patterns and electronic properties (Fig. 3). Both cyclic and acyclic alkenes were suitable substrates for reactions with **1a**. For example, a series of simple cyclic olefins with various ring sizes afforded the corresponding *β,γ*-unsaturated ketones (**30**–**33**).

Evaluation of various acyclic alkenes revealed that compounds with long or short alkyl chains, terminal olefins, or internal olefins with two or three substituents could be used as substrates and afforded the corresponding products (**34**–**40**) in moderate to good yields. Notably, the isomeric products (**37** and **37′**) of an asymmetrically substituted olefin could be separated. The exclusive formation of the benzyl acylation product (**40**) though bearing a terminal doble bond can be readily rationalized by consideration of the ortho oxygen reducing the BDE value of this site and its stabilizing effect on free radicals. Moreover, a series of benzylic C(sp^3^)–H substrates were successfully employed in this coupling reaction; specifically, primary, secondary, and tertiary benzyl substrates gave products **41**–**43**, respectively, in 66–77% yields. Finally, the mild reaction conditions were suitable for the modification of natural products with various biological activities. For example, acylation of flavor molecules isopulegol, terpinyl acetate, limonene, 3-carene, and dicyclopentadiene afforded **44**–**48**, respectively. Unique site selectivity mainly benefits from steric hindrance or more stable secondary radicals. Naturally occurring *γ*-terpinene, which have multiple allylic C(sp^3^)–H sites, selectively reacted at the cyclic carbon to deliver products **49** in 63% yield. Butadiene dimer, which is used as an indigo dye, underwent direct allylic acylation at two sites, affording a mixture of isomers (**50**). In addition, modification of derivative of (*S*)-*β*-citronellol, a molecule with pesticidal activity, could be achieved by means of this method, which gave product **51** in 49% yield.

### Transformations of *β,γ*-unsaturated ketone products

**Fig. 4:**
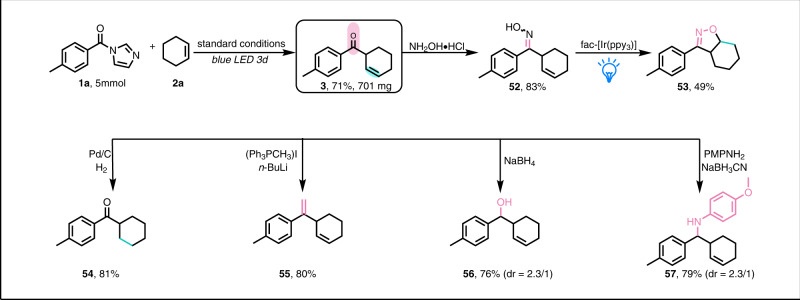
Transformations of a representative *β,γ*-unsaturated ketone. Except for the gram-scale synthesis, the other reactions were performed on a 0.2 mmol scale. PMPNH_2_: 4-methoxyaniline.

The *β,γ*-unsaturated ketones generated by means of our method could be scaled up in a slightly lower yield (71%) after 3 days of irradiation and easily be transformed to other useful molecules (Fig. 4). For example, treatment of **3** with hydroxylamine hydrochloride afforded *β,γ*-unsaturated ketoxime **52**, which was a convenient raw material for the synthesis of isoxazoline **53**^95–100^. The C=C bond of **3** could also be selectively reduced to afford **54**. Moreover, terminal olefin **55,** *β,γ*-unsaturated alcohol **56**, and secondary amine **57** could be obtained via Wittig reaction, sodium borohydride reduction, and reductive amination, respectively.

### Development of one-pot protocol

**Fig. 5:**
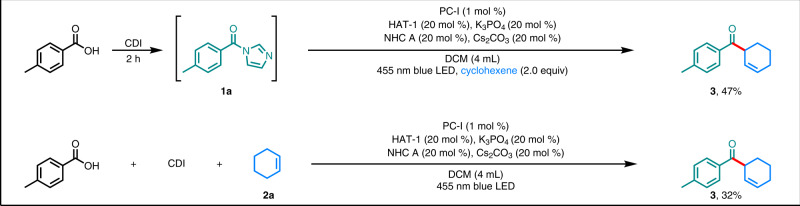
One-pot synthesis of *β,γ*-unsaturated ketone 3 from *p*-toluic acid. Reactions were performed on a 0.2 mmol scale. CDI: *N,N-carbonyldiimidazole*.

In addition, to demonstrate the operational simplicity and utility of the method, we synthesized *β,γ*-unsaturated ketone **3** in one pot directly from *p*-toluic acid (Fig. 5). The acyl imidazole was generated in situ by reaction of the carboxylic acid with CDI, and then reaction with cyclohexene (**2a**) under the standard conditions produced **3** in 47% yield (compared with the 77% yield obtained when the acylimidazole was used as the substrate). Furthermore, a one-pot reaction of *p*-toluic acid, CDI, and cyclohexene under the standard conditions afforded **3** in 32% yield.

### Mechanistic studies

**Fig. 6:**
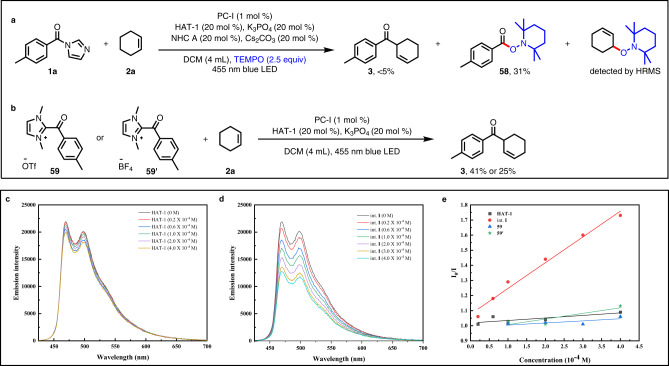
Mechanistic experiments. **a** Reaction of **1a** and **2a** in the presence of TEMPO (2,2,6,6-tetramethylpiperidinooxy). **b** Reaction of **2a** with intermediate **59** or **59’**. **c** Stern–Volmer quenching experiments involving PC-**I** and thiol HAT**−1**. **d** Stern–Volmer quenching experiments involving PC-**I** and thiolate int. **I**. **e** Stern–Volmer analysis.

Next, we turned our attention to the mechanism of this multicatalytic process. First, to determine whether radicals were involved, we performed radical-trapping experiment using TEMPO (2,2,6,6-tetramethylpiperidine oxide) as a radical scavenger. We found that reaction of **1a** and **2a** was greatly suppressed by the scavenger, and the addition product of **2a** and TEMPO was detected by mass spectrometry. In addition, radical capture product **58** was isolated from the reaction, supporting the formation of an acyl radical intermediate (Fig. 6a). Notably, reaction of **2a** with acyl azolium ion **59** or **59’** under photoredox catalysis conditions provided ketone **3** in 41% and 25% yields; this result indicates that the acyl azolium species, generated in situ from the acylimidazole and NHC catalyst, might be the intermediate (Fig. 6b). Next, we performed Stern–Volmer quenching experiments using intermediate **59,** **59’**, ^*i*^Pr_3_SiSH (HAT**−1**) and ^*i*^Pr_3_SiS^−^ (int. **I**) as quenching reagents (Fig. 6c–e). The results of these experiments showed that reduction of the excited photocatalyst by the thiol in the presence of ^*n*^Bu_4_NOAc resulted in strong fluorescence quenching that was linearly correlated with thiolate concentration (Fig. 6e, red line). In contrast, thiol (HAT**−1**), **59** and **59’** did not obviously quench the excited catalyst (Fig. 6e, black, blue and green line). Finally, a light on/off experiment showed that the reaction stopped completely in the absence of light and then resumed when the light was turned back on, indicating that light was essential (data not shown). This result indicates that any radical chain processes were short-lived.

**Fig. 7:**
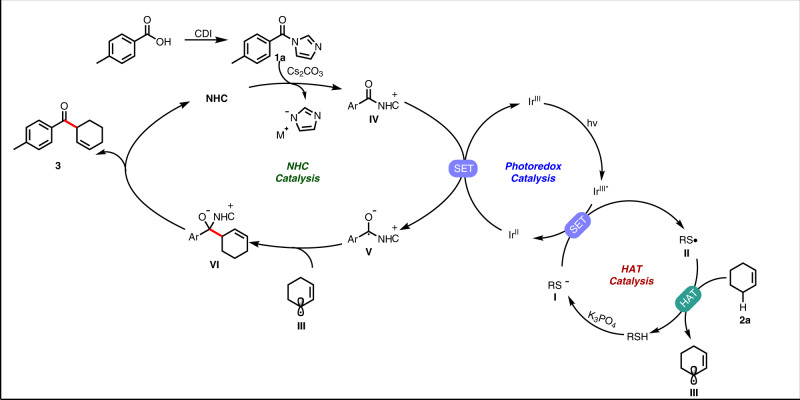
Proposed mechanism. NHC, HAT and photocatalyst co-catalyzed mechanism.

Based on the above mechanism experiments and literature reports, we propose that the mechanism of photoredox/NHC/HAT catalyzed formation of *β,γ*-unsaturated ketones from carboxylic acids and olefins is carried out through the mechanism in Fig. 7. Blue light irradiation converts the Ir^III^ photocatalyst into a long-lived triplet excited state Ir^III*^ complex, which is reduced to an Ir^II^ species by thiolate **I** generated in situ. The resulting electrophilic thiyl radical (**II**) can be used as a powerful HAT catalyst to abstract allylic hydrogen from cyclohexene to generate transient radical **III**. At the same time, the carboxylic acid is activated in situ by reaction with CDI, and then NHC catalyst is added to the activated acid (**1a**) to generate azolium intermediate I**V**, which can be reduced by Ir^II^ species to provide azolium radical **V** and regenerate the ground state Ir^III^ photocatalyst. Subsequently the coupling of **V** with radical **III** ultimately yields the target β, γ-unsaturated ketone **3** and releases the NHC catalyst.

## Discussion

In summary, we have developed a method for preparing *β,γ*-unsaturated ketones from carboxylic acids and alkenes by means of a process involving single-electron reduction of acyl azolium intermediates. The co-catalytic mode of triple catalysis involving photoredox, NHC, and HAT catalyst, enable the single-electron reduction of the acyl azolium intermediates; and subsequent radical–radical coupling readily forms a C–C bond under mild conditions to generate *β,γ*-unsaturated ketones. The method uses easy-to-prepare or commercially available starting materials and has a wide substrate scope and excellent functional group tolerance; our successful modification of various natural product and drug molecules demonstrates the potential utility of the method. The products could also undergo subsequent transformations. Continued research on such multicatalytic processes can be expected to facilitate the development of additional carbene-mediated reactions.

## Methods

### General procedure for the radical reaction

An 8 mL glass vial was charged with PC-I (3.4 mg, 0.003 mmol, 1 mol%), **1** (0.3 mmol, 1.0 equiv), **2** (0.6 mmol, 2.0 equiv), NHC **A** (13.5 mg, 0.06 mmol, 20 mol%), Cs_2_CO_3_ (21.2 mg, 0.06 mmol, 20 mol%), HAT-1 (11.4 mg, 0.06 mmol, 20 mol%), K_3_PO_4_ (12.7 mg, 0.06 mmol, 20 mol%), and 4 mL of anhydrous DCM. Argon was bubbled through the reaction mixture for 15 s via an outlet needle, and the vial was sealed with a PTFE cap. The mixture was then stirred rapidly while being irradiated with a 36 W blue LED (placed approximately 2 cm away from the vial) at room temperature for 24 h. The mixture was concentrated in vacuo, and the residue was subjected to flash chromatography on silica gel to afford the desired product in pure form.

## Supplementary information

Supplementary Information

## Data Availability

All data generated in this study are provided in the Supplementary Information, and can be obtained from the authors upon request.
