# Supplementary material for: Direct allylic acylation via cross-coupling involving cooperative N‑heterocyclic carbene, hydrogen atom transfer, and photoredox catalysis
Source: Nat Commun. 2023 May 23;14:2951. doi: 10.1038/s41467-023-38743-8 (PMC10206076; doi:10.1038/s41467-023-38743-8)
Supplement: Supplementary file 1 — Supplementary Information [file 41467_2023_38743_MOESM1_ESM.pdf]

Supplementary Information

Direct Allylic Acylation via Cross-Coupling Involving Cooperative N-Heterocyclic Carbene, Hydrogen Atom Transfer, and Photoredox Catalysis

Xiaochen Wang[1], Rongxin Yang[1], Binbing Zhu[1], Yuxiu Liu[1], Hongjian Song[1], Jianyang Dong[1] and Qingmin Wang[1]*

[1]State Key Laboratory of Elemento-Organic Chemistry, Research Institute of Elemento-Organic Chemistry, Frontiers Science Center for New Organic Matter, College of Chemistry, Nankai University, Tianjin 300071, China

*Email: wangqm@nankai.edu.cn.

Table of contents

Supplementary Notes……………………………………………………………………….S3

Supplementary Discussion……………………………………………………………………S3

Supplementary Methods……………………………………………………………………S11

Appendix: ^{1}H and ^{13}C NMR spectra for new compounds……………………………………S32

Supplementary References…………………………………………………………………S90

1. Supplementary Notes

Reagents were purchased from commercial sources and were used as received. ^{1}H and ^{13}C Nuclear Magnetic Resonance (NMR) spectra were recorded on Bruker Avance 400 Ultrashield NMR spectrometers. Chemical shifts (δ) were given in parts per million (ppm) and were measured downfield from internal tetramethylsilane. High-resolution mass spectrometry (HRMS) data were obtained on an FTICR-MS instrument (Ionspec 7.0 T). The melting points were determined on an X-4 microscope melting point apparatus and are uncorrected. Conversion was monitored by thin layer chromatography (TLC). Flash column chromatography was performed over silica gel (100-200 mesh). Blue LED (36 W, λmax = 470 nm) purchased from JIADENG (LS) was used for blue light irradiation. A fan attached to the apparatus was used to maintain the reaction temperature at room temperature

2. Supplementary Discussion

2.1 Investigation of the key reaction parameters.

Supplementary Table 1. control experiment[a]

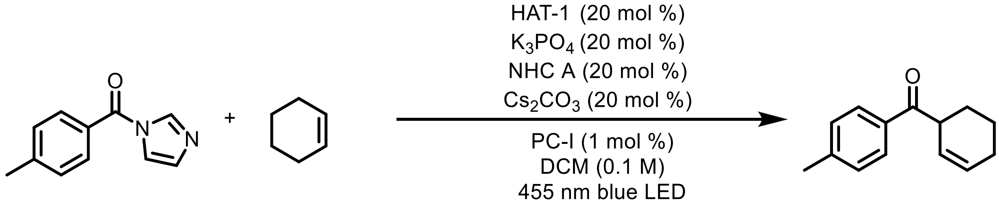

entry	conditions	yield (%) [b]
1	w/o Ir*	NR
2	w/o NHC	NR
3	w/o light	NR
4	w/o base	NR

[a] General conditions, unless otherwise noted: **1a** (0.3 mmol), **2a** (0.6 mmol), NHC A catalyst (0.06 mmol), photocatalyst (0.003 mmol), Cs_2CO_3 (0.06 mmol), K_3PO_4 (0.06 mmol), HAT-1 (0.06 mmol) and CH_3CN (3 mL) under Ar atmosphere. [b] Determined by ^{1}H NMR spectroscopy with dibromomethane as an internal standard. NR, no reaction.

Supplementary Table 2. Screening of different solvents[a]

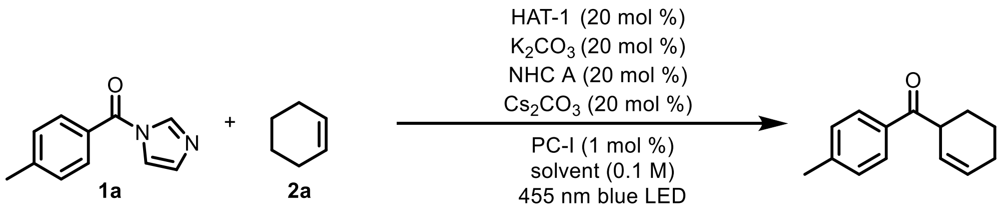

entry	solvent	yield (%) [b]
1	CH_3CN	27
2	DCM	41
3 [c]	DCM	55
4	DCE	14
5	DMF	NR
6	ACE	27
7	$CHCl_3$	30
8	THF	NR

[a] General conditions, unless otherwise noted: **1a** (0.3 mmol), **2a** (0.6 mmol), NHC catalyst (0.06 mmol), photocatalyst (0.003 mmol), Cs_2CO_3 (0.06 mmol), K_2CO_3 (0.06 mmol), HAT-1 (0.06 mmol) and solvent (3 mL) under Ar atmosphere. [b] Determined by ^{1}H NMR spectroscopy with dibromomethane as an internal standard. NR, no reaction. [c] dry solvent

Supplementary Table 3. Screening of NHC catalysts[a]

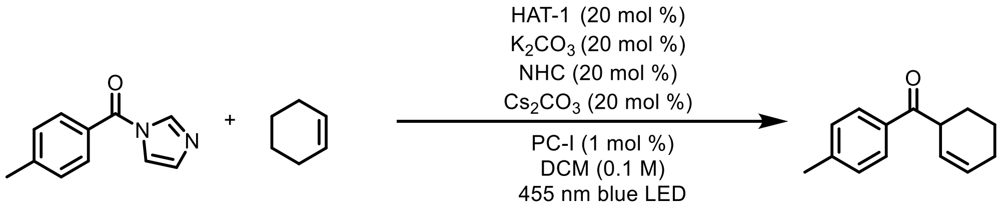

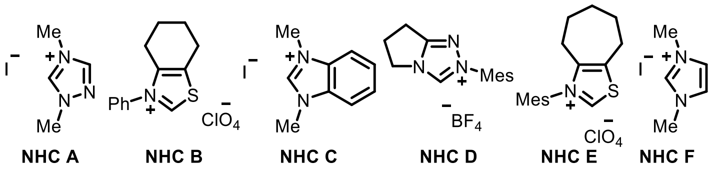

entry	NHC	yield (%) [b]
1	A	55
2	B	NR
3	C	<5
4	D	45
5	E	NR
6	F	<5

[a] General conditions, unless otherwise noted: **1a** (0.3 mmol), **2a** (0.6 mmol), NHC catalyst (0.06 mmol), photocatalyst (0.003 mmol), Cs_2CO_3 (0.06 mmol), K_2CO_3 (0.06 mmol), HAT-1 (0.06 mmol) and DCM (3 mL) under Ar atmosphere. [b] Determined by ^{1}H NMR spectroscopy with dibromomethane as an internal standard. NR, no reaction.

Supplementary Table 4. Screening of base 1[a]

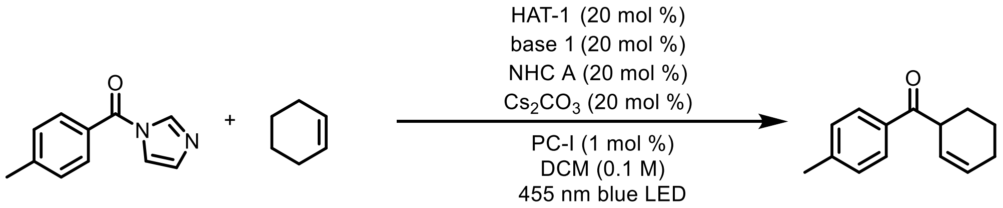

entry	base	yield (%) [b]
1	$NaHCO_3$	54
2	Na_2CO_3	41
3	Cs_2CO_3	27
4	K_2CO_3	55
5	K_3PO_4	60
6	Na_2HPO_4	31
7	NaOAc	19
8	$^{n}Bu_4NOAc$	36
9	Pyridine	15
10	DBU	19
11	NEt_3	13

[a] General conditions, unless otherwise noted: **1a** (0.3 mmol), **2a** (0.6 mmol), NHC A catalyst (0.06 mmol), photocatalyst (0.003 mmol), Cs_2CO_3 (0.06 mmol), base 1 (0.06 mmol), HAT-1 (0.06 mmol) and DCM (3 mL) under Ar atmosphere. [b] Determined by ^{1}H NMR spectroscopy with dibromomethane as an internal standard. NR, no reaction.

Supplementary Table 5. Screening of base 2[a]

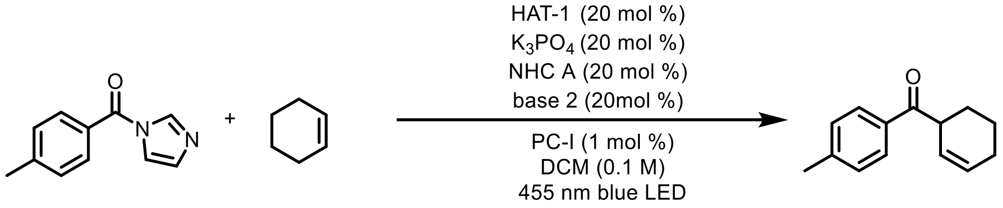

entry	base 2	yield (%) [b]
1	$NaHCO_3$	30
2	Na_2CO_3	27
3	K_3PO_4	27
4	Cs_2CO_3	60
5	K_2CO_3	12
6	Na_2HPO_4	32
7	NEt_3	26
8	DIPEA	NR

[a] General conditions, unless otherwise noted: **1a** (0.3 mmol), **2a** (0.6 mmol), NHC A catalyst (0.06 mmol), photocatalyst (0.003 mmol), K_3PO_4 (0.06 mmol), base 2 (0.06 mmol), HAT-1 (0.06 mmol) and DCM (3 mL) under Ar atmosphere. [b] Determined by ^{1}H NMR spectroscopy with dibromomethane as an internal standard. NR, no reaction.

Supplementary Table 6. Screening of concentration[a]

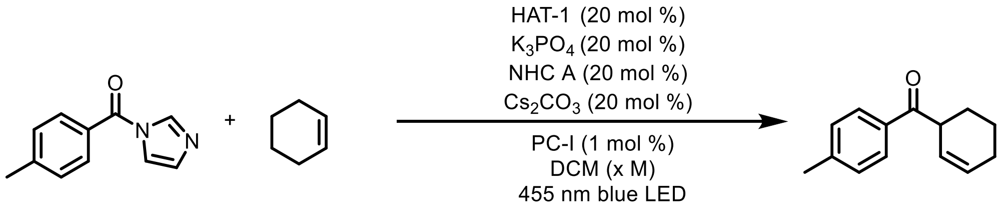

entry	concentration	yield (%) [b]
1	0.6	24
2	0.3	37
3	0.2	46
4	0.1	60
5	0.075	77

[a] General conditions, unless otherwise noted: **1a** (0.3 mmol), **2a** (0.6 mmol), NHC A catalyst (0.06 mmol), photocatalyst (0.003 mmol), Cs_2CO_3 (0.06 mmol), K_3PO_4 (0.06 mmol), HAT-1 (0.06 mmol) and DCM (x M) under Ar atmosphere. [b] Determined by ^{1}H NMR spectroscopy with dibromomethane as an internal standard. NR, no reaction.

Supplementary Table 7. Screening of photocatalysts[a]

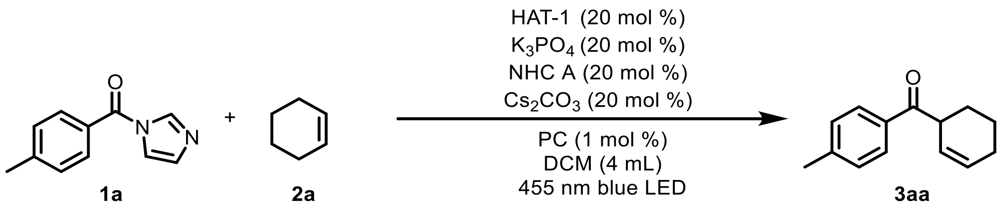

1a **2a** **3aa**

entry	photocatalyst	yield (%) [b]
1	$[Ru(bpy)_3]Cl_2 \cdot 6H_2O$	31
2	$[Ru(bpy)_3](PF_6)_2$	12
3	$[Ir\{dFCF_3ppy\}_2(bpy)]PF_6$	83 (77) [c]
4	$Ir(ppy)_3$	29
5	4CzIPN	NR

[a] General conditions, unless otherwise noted: **1a** (0.3 mmol), **2a** (0.6 mmol), NHC catalyst (0.045 mmol), photocatalyst (0.003 mmol), Cs_2CO_3 (0.045 mmol), Cs_2CO_3 (0.06 mmol), K_3PO_4 (0.06 mmol), HAT-1 (0.06 mmol) and DCM (4 mL) were irradiated with a 36 W blue LED under Ar at rt. [b] NMR yield Determined by ^{1}H NMR spectroscopy with dibromomethane as an internal standard. NR, no reaction. [c]isolated yield

Supplementary Table 8. Screening of the amount of photocatalysts[a]

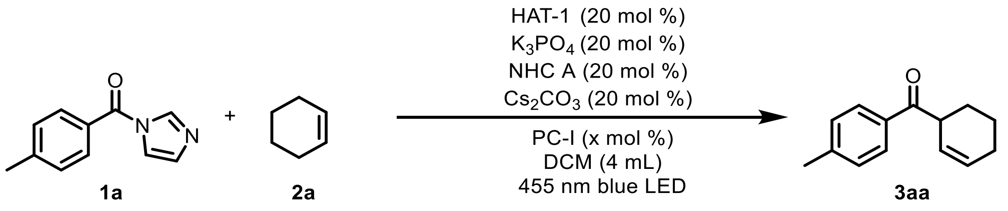

entry	x	yield (%) [b]
1	0	NR
2	0.5	41
3	1	83 (77) [c]
4	3	79[c]

[a] General conditions, unless otherwise noted: **1a** (0.3 mmol), **2a** (0.6 mmol), NHC catalyst (0.045 mmol), photocatalyst (0.003x mmol), Cs_2CO_3 (0.06 mmol), Cs_2CO_3 (0.06 mmol), K_3PO_4 (0.06 mmol), HAT-1 (0.06 mmol) and DCM (4 mL) were irradiated with a 36 W blue LED under Ar at rt. [b] Determined by ^{1}H NMR spectroscopy with dibromomethane as an internal standard. NR, no reaction. [c]isolated yield

Supplementary Table 9. Screening of the HAT catalysts[a]

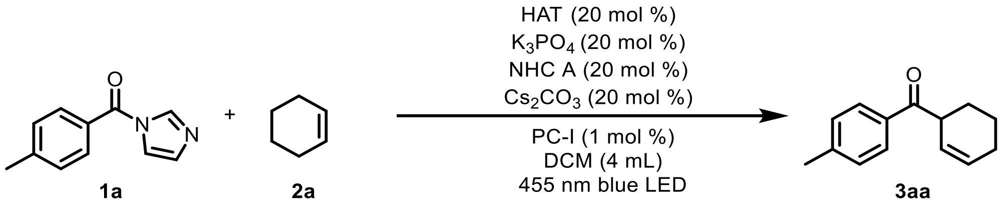

entry	HAT catalysts	yield (%) [b]
1	HAT-1	83 (77) [c]
2	HAT-2	31
3	HAT-3	17
4	HAT-4	NR
5	HAT-5	11

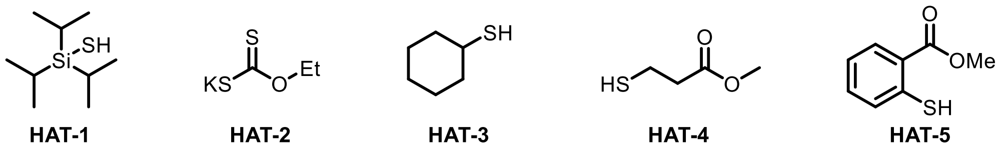

[a] General conditions, unless otherwise noted: **1a** (0.3 mmol), **2a** (0.6 mmol), NHC catalyst (0.045 mmol), photocatalyst (0.003 mmol), Cs_2CO_3 (0.06 mmol), Cs_2CO_3 (0.06 mmol), K_3PO_4 (0.06 mmol), HAT (0.06 mmol) and DCM (4 mL) were irradiated with a 36 W blue LED under Ar at rt. [b] Determined by ^{1}H NMR spectroscopy with dibromomethane as an internal standard. NR, no reaction. [c]isolated yield

2.2 Investigation of the mechanism.

2.2.1 TEMPO was used as radical scavengers.

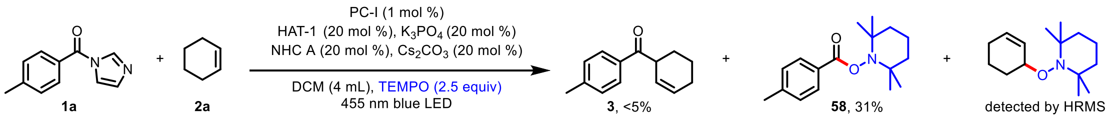

Supplementary Figure 1 Radical trapping experiment

To a 8 mL glass vial was added PC-I (3.4 mg, 0.003 mmol, 1 mol %), **1a** (55.86 mg, 0.3 mmol, 1.0

equiv), **2a** (49.2 mg, 0.6 mmol, 2.0 equiv), TEMPO (117 mg, 0.75 mmol, 2.5 equiv), NHC A (13.5 mg, 0.06 mmol, 20 mol %), Cs_2CO_3 (21.2 mg, 0.06 mmol, 20 mol%), HAT-1 (11.4 mg, 0.06 mmol, 20 mol%), K_3PO_4 (12.7 mg, 0.06 mmol, 20 mol%) and 4.0 mL of anhydrous DCM. The reaction mixture was degassed by bubbling with Ar for 15 s with an outlet needle and the vial was sealed with PTFE cap. The mixture was then stirred rapidly and irradiated with a 36 W Blue LED (approximately 2 cm away from the light source) at room temperature for 24 h. The corresponding product **3** was not observed based on ^{1}H NMR analysis in this case, and the corresponding product of radical trapping, 1-(cyclohex-2-en-1-yloxy)-2,2,6,6-tetramethylpiperidine, was observed by mass spectrometry. And the corresponding product of radical trapping 2,2,6,6-tetramethylpiperidin-1-yl 4-methylbenzoate (**58**) was isolated in 31% yield.

2,2,6,6-tetramethylpiperidin-1-yl 4-methylbenzoate (58)

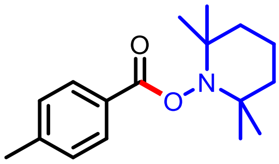

According to the *general procedure*. colorless oil (25.6 mg, 31%)

^{1}H NMR (400 MHz, $CDCl_3$) δ 7.89 (d, J = 8.0 Hz, 2H), 7.17 (d, J = 8.0 Hz, 2H), 2.34 (s, 3H), 1.35 (dt, J = 15.2, 7.6 Hz, 4H), 1.21 – 1.17 (m, 2H), 1.08 (s, 6H), 1.06 (s, 6H).

^{13}C NMR (100 MHz, $CDCl_3$) δ 165.3, 142.5, 129.2, 128.0, 127.7, 20.6, 16.9, 11.1, 0.1.

HRMS (ESI) calcd for $C_{17}H_{26}NO$ $[M + H]^+$276.1958, found 276.1958

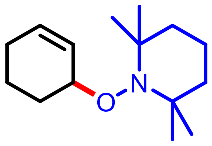

HRMS (ESI) calcd for $C_{15}H_{28}NO$ $[M + H]^+$238.2165, found 238.2167

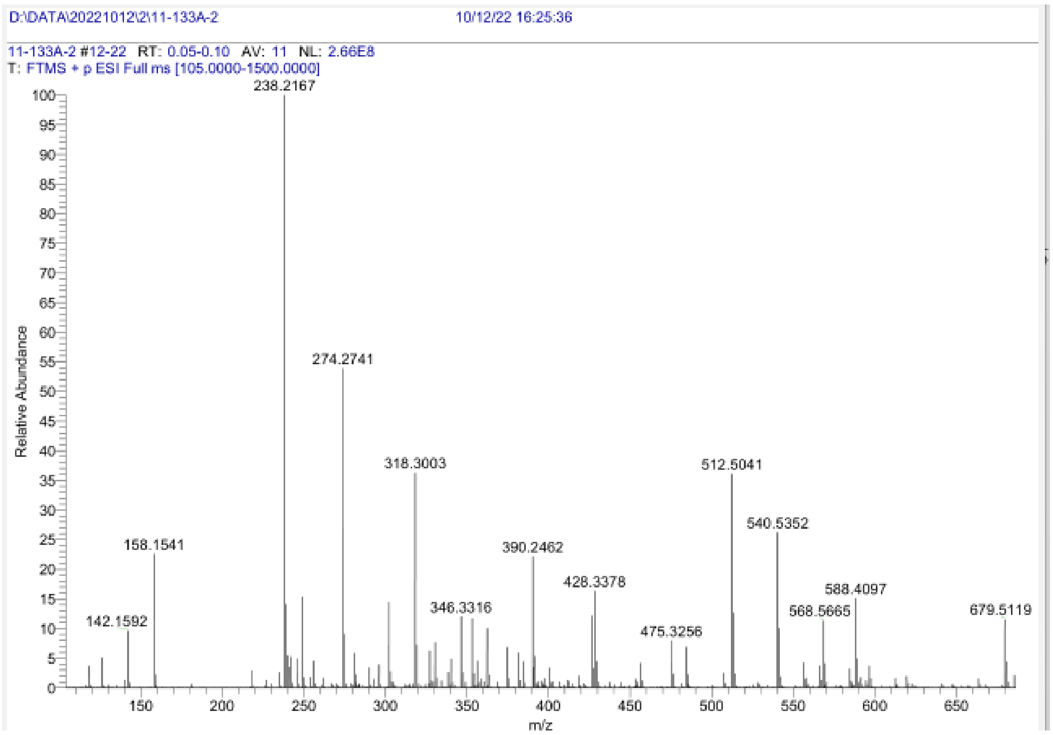

Supplementary Figure 2 HRMS of radical trapping product

2.2.2 59 or 59' was used as a intermediate.

To a 8 mL glass vial was added **59** (109.3 mg, 0.3 mmol, 1.0 equiv) or **59'** (90.6 mg, 0.3 mmol, 1.0 equiv), PC-I (3.4 mg, 0.003 mmol, 1 mol %), **2a** (49.3mg, 0.6 mmol, 2.0 equiv), HAT-1 (11.7 mg, 0.06 mmol, 20 mol%), K_3PO_4 (12.7 mg, 0.06 mmol, 20 mol%) and 4.0 mL of anhydrous DCM. The reaction mixture was degassed by bubbling with Ar for 15 s with an outlet needle and the vial was sealed with PTFE cap. The mixture was then stirred rapidly and irradiated with a 36 W Blue LED (approximately 2 cm away from the light source) at room temperature for 24 h. The mixture was concentrated in vacuo. Purification of the crude product by flash chromatography on silica gel (PE/EA = 20: 1) afforded the desired product.

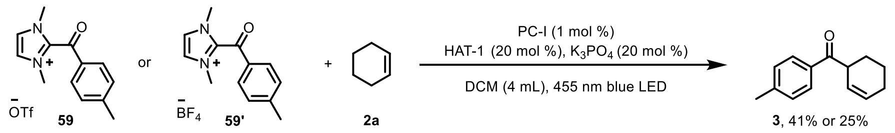

Supplementary Figure3 59 or 59' was used as intermediate

2.2.3 Stern-Volmer quenching experiments of PC-I

Quenching experiments were carried out using a 1 x 10^{-5} M solution of PC-I in DCM and variable concentrations of quencher thiol (20, 60, 100, 200, 300, 400 μM) in the presence of $^{n}Bu_4NOAc$ (same concentration of the thiol) in DCM. Stock solution of thiol and the base was prepared by stirring the thiol (1.0 equiv) and $^{n}Bu_4NOAc$ (1.0 equiv) in DCM overnight. The quenching rate constant (k_q = 7.42 x 10^8 $M^{-1}s^{-1}$) was calculated by using the reported[1] lifetime of PC-I (2300 ns).

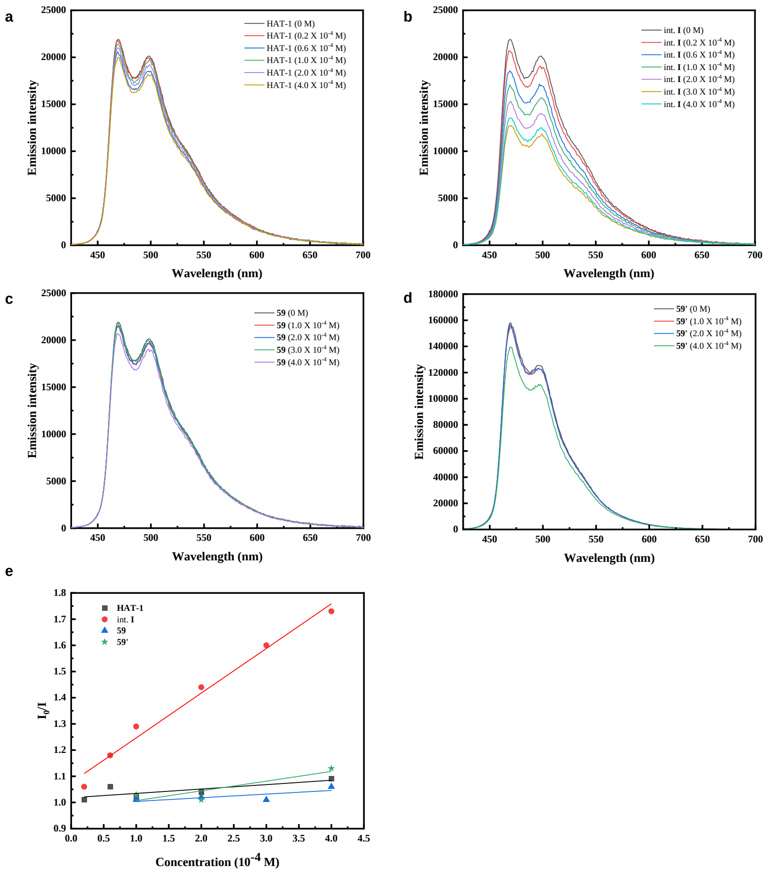

Supplementary Figure 4 Mechanistic experiments. (a) Stern–Volmer quenching experiments between PC-**I** and HAT-**1**. (b) Stern–Volmer quenching experiments between PC-**I** and int. **I**. (c) Stern–

Volmer quenching experiments between PC-**I** and **59**. (d) Stern–Volmer quenching experiments between PC-**I** and **59'**. (e) Stern–Volmer analysis

2.2.4 Light/dark experiment.

Supplementary Table 10 Light/dark experiment.

entry	light on and off conditions	yield (%)
1	on 2 hours	30
2	off 2 hours	30
3	on 2 hours	54
4	off 2 hours	55
5	on 2 hours	65
6	off 2 hours	65
7	on 2 hours	71
8	off 2 hours	71

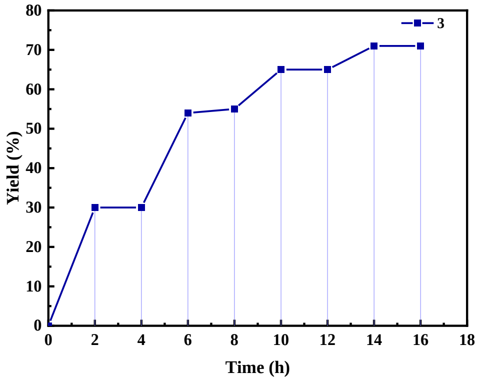

Supplementary Figure 5 Light/dark experiment

2.2.5 Exploration the role of K_3PO_4 and Cs_2CO_3

In order to infer the effect of each base, we performed the following experiments:

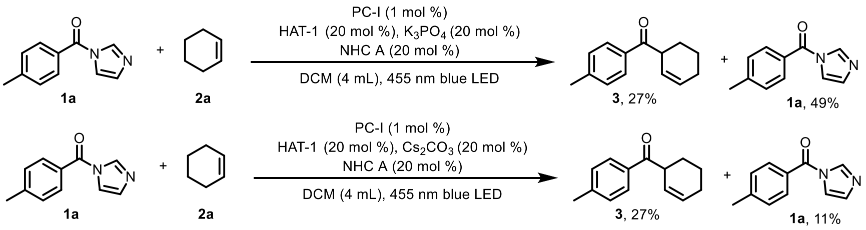

Supplementary Figure 6 The role of Cs_2CO_3

When we use a single potassium phosphate as a catalyst in the template reaction, we can separate a large amount of unreacted substrate **1a** (49%), but when using a single cesium carbonate as a catalyst, the amount of substrate remaining is less (11%). It's reflected from the side that Cs_2CO_3 probably acts on reaction of NHC precursor and acylimidazole to form acyl azolium intermediate which means Cs_2CO_3 mainly played the role in facilitating NHC precatalyst to NHC catalyst.

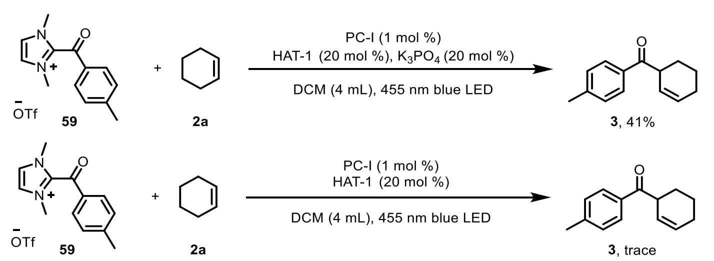

Supplementary Figure 7 The role of K_3PO_4

In mechanistic experiments we found that reaction of **2a** with acyl azolium ion **59** under photoredox catalysis conditions provided ketone **3** in 41% yield, this experiment was carried out under the condition of K_3PO_4 as base. But when we removed K_3PO_4, the reaction cannot be carried out and the product was not obtained, this result showed that K_3PO_4 mainly acts on deprotonation of thiol to generate sulfur anion to mediate the formation of allyl radicals.

In summary, Cs_2CO_3 mainly acts on the formation of NHC catalyst, that is, the production of azolium radical, while K_3PO_4 mainly acts on the deprotonation process of thiols, that is, the formation of allyl radicals. However, in the reaction system, two bases cannot completely act independently, and our experiment is only to prove the main role of two bases, and it cannot be absolutely said that one base only plays an independent role, a mixture of both bases provided the best result, as discussed in the manuscript.

3. Supplementary Methods

3.1 General procedure for the synthesis of acyl imidazoles

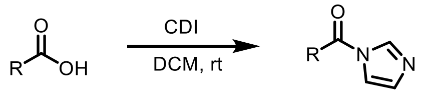

Supplementary Figure 8 Synthesis of acyl imidazoles

Acyl imidazoles were prepared based on the literature[2]: The appropriate acid (10 mmol, 1.0 equiv) was dissolved in dry dichloromethane (0.3 M), and CDI (carbonyldiimidazole, 15 mmol, 1.5 equiv) was added slowly (caution, exothermic). The resulting mixture was stirred for 12 h at room temperature. Upon completion, the solution was transferred to a separatory funnel and washed with deionized water (2 x 25 mL), and then the organic layer was dried over $MgSO_4$. Concentration under reduced pressure afforded the acyl imidazole, which was used in the following reaction without further purification.

3.2 General procedure for the synthesis of NHC A[3]

A mixture of 1,2,4-triazole (1.0 g, 14.5 mmol), iodomethane (6.2 g, 43.5 mmol), and potassium carbonate (3.0 g, 21.7 mmol) in acetonitrile (8 mL) and methanol (2 mL) was heated at 40 °C for 3 days. The white mixture was filtered with a Buckner funnel, and the white solid was washed with CH_2Cl_2. The filtrate was concentrated to give 2,4-dimethyl-1,2,4-triazolium iodide (white solid, 3.28 g, 100%)

3.3 Preparation of carboxylic acids, derived from diacetone-D-glucose, pregnenolone, L-Menthol

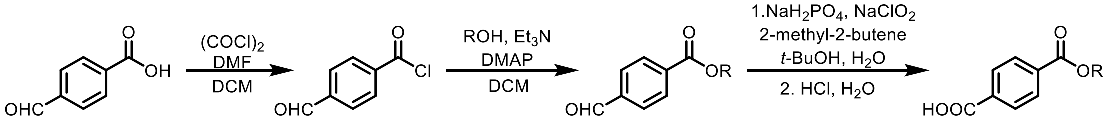

Supplementary Figure 9 Preparation of carboxylic acids

Step 1[4]: To a solution of 4-formylbenzoic acid (1.0 equiv) in dichloromethane (0.3 M) was added oxalyl chloride (1.5 equiv) dropwise at 0 °C, and one drop of DMF was subsequently added to the solution. Then the mixture was transferred to room temperature and stirred at the same temperature overnight. After the indicated time, the mixture was evaporated to dry under reduced pressure, and the crude acyl chloride was used directly for the next step without further purification.

Step 2[5]: To the mixture of ROH (1.0 equiv), Et_3N (1.0 equiv) and DMAP (0.05 equiv) in DCM (1.0 M) was added TsCl (1.l equiv) dropwise at 0 °C. The temperature was maintained at 0 °C for 3 h, and stirred at room temperature overnight, after which the reaction was quenched by saturated $NaHCO_3$ (20 mL) and extracted by DCM (20 mL × 3). The combined organic phase was dried over Na_2SO_4, filtered, concentrated, and purified by flash chromatography with silica gel column, affording the corresponding aromatic aldehydes.

Step 3[6]: To a solution of aromatic aldehyde (1 equiv), NaH_2PO_4 (1 equiv), 2-methyl-2-butene (4.42 equiv) in *tert*-BuOH (0.16 M) and water (0.6 M) was added $NaClO_2$ (3.4 equiv) and the mixture was stirred for 50 min at room temperature. The reaction mixture was adjusted to pH of 4 by addition of 1 M HCl. The aqueous layer was extracted with CH_2Cl_2. The organic layers were combined, washed with brine, dried over anhydrous Na_2SO_4. Purification by flash chromatography (petroleum ether/EtOAc), afforded the corresponding aromatic carboxylic acids.

3.4 General procedure for the radical reaction:

To a 8 mL glass vial was added PC-I (3.4 mg, 0.003 mmol, 1 mol %), **1** (0.3 mmol, 1.0 equiv), **2** (0.6 mmol, 2.0 equiv), NHC A (13.5 mg, 0.06 mmol, 20 mol %), Cs_2CO_3 (21.2 mg, 0.06 mmol, 20 mol%), HAT-1 (11.4 mg, 0.06 mmol, 20 mol%), K_3PO_4 (12.7 mg, 0.06 mmol, 20 mol%) and 4.0 mL of anhydrous DCM. The reaction mixture was degassed by bubbling with Ar for 15 s with an outlet needle and the vial was sealed with PTFE cap. The mixture was then stirred rapidly and irradiated with a 36 W Blue LED (approximately 2 cm away from the light source) at room temperature for 24 h. The mixture was concentrated in vacuo. Purification of the crude product by flash chromatography on silica gel using the indicated solvent system afforded the desired product.

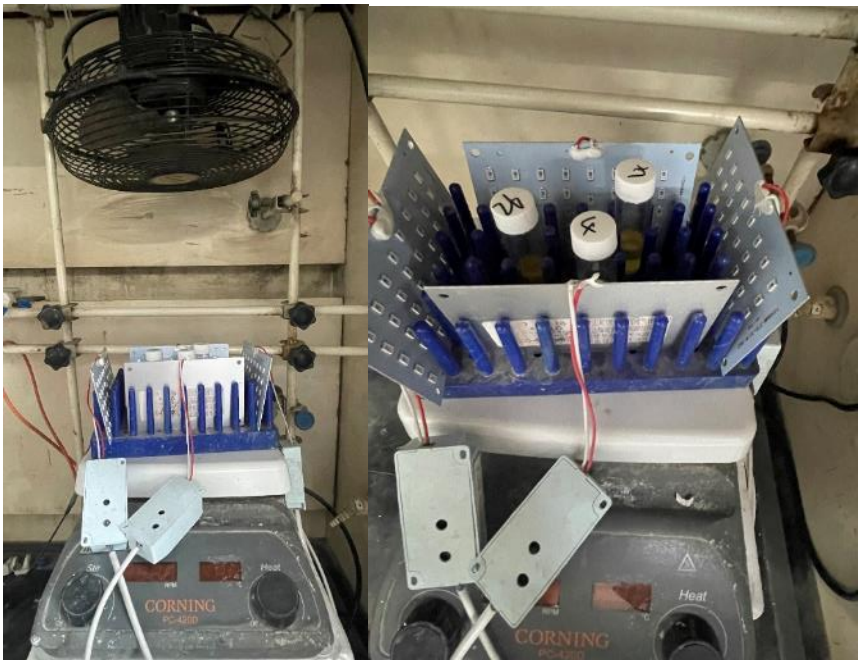

Supplementary Figure 10 Set-up of the reaction

3.5 Derivatization of products 3:

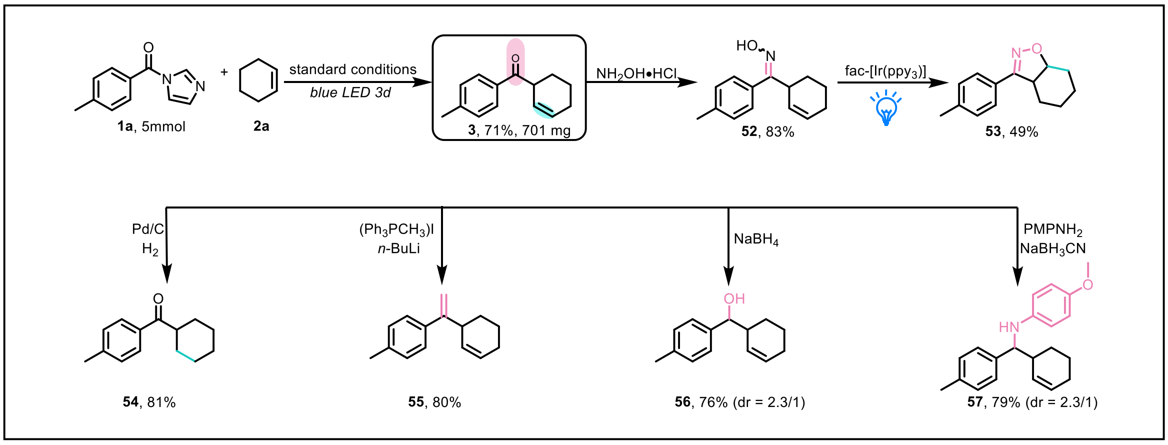

Supplementary Figure 11 Derivatization of products 3

a) General procedure for gram-scale reaction

To an oven dried Schlenk tube was added PC-I (56 mg, 0.05 mmol, 1 mol %), **1a** (931.0 mg, 5 mmol, 1.0 equiv), **2a** (821.5 mg, 10 mmol, 2.0 equiv), NHC A (225.0 mg, 0.1 mmol, 20 mol %), Cs_2CO_3 (325.8 mg, 0.06 mmol, 20 mol%), HAT-1 (190.4 mg, 0.1 mmol, 20 mol%), K_3PO_4 (212.3 mg, 0.1 mmol, 20 mol%) and 67.0 mL of anhydrous DCM. The tube was evacuated and backfilled with Ar (this process was repeated three times). The mixture was then stirred rapidly and irradiated with a 36 W Blue LED (approximately 2 cm away from the light source) at room temperature for 72 h. The mixture was concentrated in vacuo. After purification by flash column chromatography on silica gel, the product **3** was obtained in 71% (0.70 g).

b) General procedure for reaction with NH_2OH•HCl[7]

A round-bottom flask was charged with a solution of hydroxylamine hydrochloride (69.5 mg, 1.0 mmol, 5.0 equiv) in ethanol (0.6 mL). Sodium acetate (190.5 mg, 1.4 mmol, 7.0 equiv) was dissolved in water (0.6 mL) and the solution was added to the flask. The ketone **3** (40.4 mg, 0.2 mmol, 1.0 equiv) was dissolved in ethanol (0.6 mL) and added to the solution. The resulting suspension was stirred for 18 h at room temperature. The reaction was concentrated in vacuo and extracted with ethyl acetate (3 x 5 mL). The organic layers were dried over Na_2SO_4. The solvent was removed under vacuum and purified by silica gel chromatography (gradient of 10:1 PE: EA) to afford **52** as a yellow oil with 83% yield.

c) General procedure for the synthesis of isoxazolines[8]

A flame dry 8 mL tube was charged with oxime (43.2 mg, 0.2 mmol, 1.0 equiv), Na_2CO_3 (31.8 mg, 0.3 mmol, 1.5 equiv), *fac*-[Ir(ppy$_3$)] (2.6 mg, 0.004mmol, 1.5 mol%) in anhydrous $CHCl_3$ (2.5 mL) under argon atmosphere. Then, the resulting mixture was degassed via argon bubbling. The resulting suspension was stirred 36 h with the irradiation of 460 nm blue LEDs at room temperature. The mixture was extracted with ethyl acetate (3 x 15 mL). The organic layers were dried over Na2SO4. The solvent was removed under vacuum and purified by silica gel chromatography (gradient of 20:1 PE: EA) to afford **53** as a yellow oil with 49% yield.

d**) General procedure for reaction with Pd/C, H_2**[9]

To a Schlenk tube fulfilled with argon were added Pd/C (8.5 mg, 0.004 mmol, 2 mol%) and the ketone **3** (40.4 mg, 0.2 mmol, 1.0 equiv) sequentially. After addition of these chemicals, the tube was degassed and refilled with H_2 by a balloon of H_2. Then EtOAc (2 mL) was added and the resulting mixture was stirred at room temperature for 60 h. The H_2 balloon was removed and the

resulting mixture was filtered through a short column of silica gel (2 cm), eluted with ethyl acetate (5 mL x 3). The solvent was removed under vacuum and purified by silica gel chromatography (gradient of 20:1 PE: EA) to afford **54** as a colorless oil with 81% yield.

e) General procedure for Wittig reaction[10]

To an oven dried round bottom flask under argon was added methyl triphenylphosphonium iodide (162.4 mg, 0.4 mmol, 2.0 equiv) followed by THF (3.6 mL) and reaction cooled to 0 °C. *n*-BuLi (2.5 M in hexanes, 160 μL, 0.4 mmol, 2.0 equiv) was added dropwise and solution turned yellow. The reaction was stirred for 15 min. The ketone **3** (40.4 mg, 0.2 mmol, 1.0 equiv) in THF (0.4 mL) was added dropwise. The reaction was allowed to warm up to 25 °C over 30 min and stirring continued for an additional hour. The reaction was quenched by the addition of aq. NaCl. The aqueous phase was extracted with Et_2O (10 mL ×3), and the combined organic layers were dried over $MgSO_4$. The solvent was removed under vacuum and purified by silica gel chromatography (gradient of PE) to afford **55** as a colorless oil with 80% yield.

f) General procedure for reduction with $NaBH_4$[11]

A methanolic solution (1 mL) of the ketone **3** (40.4 mg, 0.2 mmol, 1.0 equiv) was cooled to 0 °C, charged with $NaBH_4$ (11.3 mg, 0.3 mmol, 1.5 equiv) portion wise, and allowed to warm to rt over 30 min. The reaction mixture was quenched with 2M HCl, concentrated in vacuo to a slurry and alkalized to pH 8 with sat. $NaHCO_3$ (2 mL). The aqueous layer was extracted with CH_2Cl_2 (3×10 mL) and the combined organic layers were dried over Na_2SO_4, filtered, and concentrated in vacuo. The resulting residue was purified by silica gel chromatography (gradient of 10:1 PE: EA) to afford **56** as a colorless oil with 76% yield.

g) Reductive amination of 3[12]

To a solution of **3** (40.4 mg, 0.2 mmol, 1.0 equiv) in MeOH (2.0 mL) were added $PMPNH_2$ (70.2 mg, 0.6 mmol, 3.0 equiv), $NaBH_3CN$ (37.8 mg, 0.6 mmol, 3.0 equiv), and two drops of HOAc sequentially. The mixture was stirred at room temperature and monitored by TLC. After the completion of the reaction, the solvent was removed under reduced pressure. Next, the residue was purified by flash column chromatography (PE/EA = 15: 1) to give **57** in 79% yield as colorless oil.

3.6 Examples of unsuccessful substrates

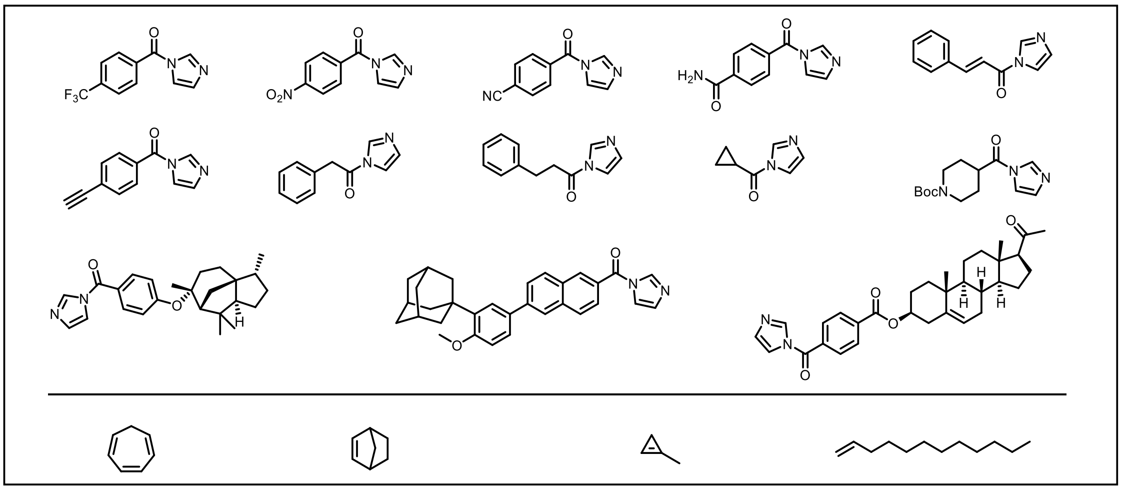

Supplementary Figure 12 Examples of unsuccessful substrates

3.7 Product characterization

cyclohex-2-en-1-yl(*p*-tolyl)methanone (3)[13]

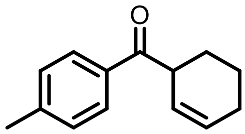

According to the *general procedure*. colorless oil (46.2 mg, 77%)

^{1}H NMR (400 MHz, $CDCl_3$) δ 7.87 (d, J = 8.0 Hz, 2H), 7.27 (d, J = 5.6 Hz, 2H), 5.95 – 5.88 (m, 1H), 5.76 – 5.70 (m, 1H), 4.10 – 4.01 (m, 1H), 2.41 (s, 3H), 2.11 – 2.05 (m, 2H), 2.01 – 1.94 (m, 1H), 1.88 – 1.81 (m, 2H), 1.71 – 1.65 (m, 1H).

^{13}C NMR (100 MHz, $CDCl_3$) δ 201.5, 143.6, 133.7, 130.0, 129.3, 128.6, 125.0, 43.8, 26.0, 24.8, 21.6, 21.0.

HRMS (ESI) calcd for $C_{14}H_{16}NaO$ $[M + Na]^+$223.1093, found 223.1093

cyclohex-2-en-1-yl(phenyl)methanone (4)[13]

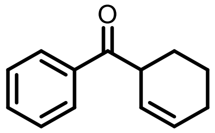

According to the *general procedure*. colorless oil (24.6 mg, 44%)

^{1}H NMR (400 MHz, $CDCl_3$) δ 7.96 (d, J = 8.0 Hz, 2H), 7.56 (dd, J = 10.4, 4.0Hz, 1H), 7.48 (d, J = 7.6 Hz, 2H), 5.97 – 5.89 (m, 1H), 5.75 (d, J = 10.0 Hz, 1H), 4.14 – 4.04 (m, 1H), 2.12 – 2.05 (m, 2H), 2.02 – 1.94 (m, 1H), 1.91 – 1.81 (m, 2H), 1.70 (dd, J = 6.5, 3.3 Hz, 1H).

^{13}C NMR (100 MHz, $CDCl_3$) δ 201.8, 136.3, 132.8, 130.1, 128.6, 128.5, 124.8, 43.9, 25.9, 24.8, 20.9.

HRMS (ESI) calcd for $C_{13}H_{15}O$ $[M + H]^+$187.1117, found 187.1119

(4-(*tert*-butyl)phenyl)(cyclohex-2-en-1-yl)methanone (5)[14]

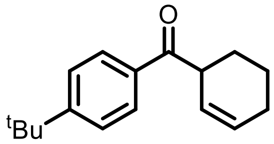

According to the *general procedure*. colorless oil (57.4 mg, 79%)

^{1}H NMR (400 MHz, $CDCl_3$) δ 7.91 (d, J = 8.4 Hz, 2H), 7.48 (d, J = 8.4 Hz, 2H), 5.96 – 5.88 (m, 1H), 5.80 – 5.71 (m, 1H), 4.12 – 4.03 (m, 1H), 2.12 – 2.04 (m, 2H), 2.01 – 1.93 (m, 1H), 1.90 – 1.80 (m, 2H), 1.73 – 1.64 (m, 1H), 1.35 (s, 9H).

^{13}C NMR (100 MHz, $CDCl_3$) δ 201.5, 156.6, 133.6, 130.0, 128.5, 125.6, 125.0, 43.8, 35.1, 31.1, 26.0, 24.8, 21.0.

HRMS (ESI) calcd for $C_{17}H_{23}O$ $[M + H]^+$243.1743, found 243.1745

[1,1'-biphenyl]-4-yl(cyclohex-2-en-1-yl)methanone (6)

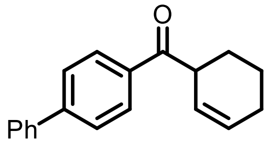

According to the *general procedure*. colorless oil (59.8 mg, 76%)

^{1}H NMR (400 MHz, $CDCl_3$) δ 8.04 (d, J = 8.0 Hz, 2H), 7.69 (d, J = 8.0 Hz, 2H), 7.63 (d, J = 7.2 Hz, 2H), 7.50 – 7.36 (m, 3H), 6.00 – 5.89 (m, 1H), 5.83 – 5.71 (m, 1H), 4.23 – 4.01 (m, 1H), 2.16 – 2.06 (m, 2H), 2.04 – 1.97 (m, 1H), 1.94 – 1.83 (m, 2H), 1.74 – 1.65 (m, 1H).

^{13}C NMR (100 MHz, $CDCl_3$) δ 201.4, 145.6, 140.0, 134.9, 130.2, 129.1, 129.0, 128.2, 127.3, 127.3, 124.8, 44.0, 25.9, 24.8, 21.0.

HRMS (ESI) calcd for $C_{19}H_{19}O$ $[M + H]^+$263.1430, found 263.1431

cyclohex-2-en-1-yl(4-methoxyphenyl)methanone (7)[15]

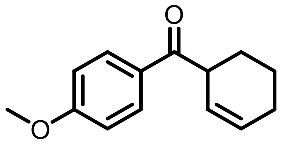

According to the *general procedure*. colorless oil (57.7 mg, 89%)

^{1}H NMR (400 MHz, $CDCl_3$) δ 8.00 – 7.87 (m, 2H), 7.03 – 6.84 (m, 2H), 5.97 – 5.85 (m, 1H), 5.78 – 5.67 (m, 1H), 4.04 (m, 1H), 3.87 (s, 3H), 2.14 – 2.03 (m, 2H), 2.00 – 1.92 (m, 1H), 1.88 – 1.78 (m, 2H), 1.73 – 1.64 (m, 1H).

^{13}C NMR (100 MHz, $CDCl_3$) δ 200.4, 163.3, 130.8, 129.9, 125.1, 113.8, 55.5, 43.6, 26.1, 24.8, 21.0.

HRMS (ESI) calcd for $C_{14}H_{16}NaO_2$ $[M + Na]^+$239.1043, found 239.1041

cyclohex-2-en-1-yl(4-propoxyphenyl)methanone (8)

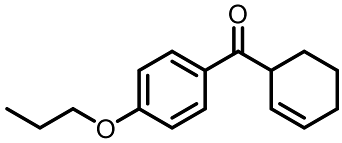

According to the *general procedure*. colorless oil (53.5mg, 73%)

^{1}H NMR (400 MHz, $CDCl_3$) δ 7.95 (dd, J = 8.8, 3.2 Hz, 2H), 6.93 (dd, J = 8.8, 3.2 Hz, 2H), 5.96 – 5.88 (m, 1H), 5.77 – 5.66 (m, 1H), 4.04 (d, J = 2.1 Hz, 1H), 4.01 – 3.93 (m, 2H), 2.12 – 2.04 (m, 2H), 2.01 – 1.92 (m, 1H), 1.91 – 1.80 (m, 4H), 1.73 – 1.66 (m, 1H), 1.05 (td, J = 7.4, 3.4 Hz, 3H).

^{13}C NMR (100 MHz, $CDCl_3$) δ 200.4, 163.0, 130.8, 129.8, 128.9, 125.2, 114.2, 69.7, 43.6, 26.1, 24.8, 22.5, 21.0, 10.5.

HRMS (ESI) calcd for $C_{16}H_{20}NaO_2$ $[M + Na]^+$267.1356, found 267.1356

(4-(allyloxy)phenyl)(cyclohex-2-en-1-yl)methanone (9)

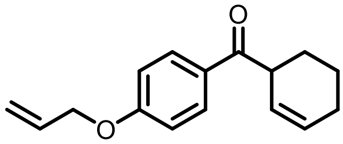

According to the *general procedure*. colorless oil (50.2mg, 69%)

^{1}H NMR (400 MHz, $CDCl_3$) δ 7.97 (d, J = 8.8 Hz, 2H), 6.97 (d, J = 8.8 Hz, 2H), 6.14 – 6.01 (m, 1H), 5.97 – 5.90 (m, 1H), 5.75 (dd, J = 10.0, 2.0 Hz, 1H), 5.45 (dd, J = 17.2, 1.2 Hz, 1H), 5.34 (dd, J = 10.4, 1.2 Hz, 1H), 4.62 (d, J = 5.2 Hz, 2H), 4.11 – 4.01 (m, 1H), 2.13 – 2.06 (m, 2H), 2.02 – 1.94 (m, 1H), 1.91 – 1.82 (m, 2H), 1.75 – 1.68 (m, 1H).

^{13}C NMR (100 MHz, $CDCl_3$) δ 200.4, 162.3, 132.5, 130.8, 129.9, 129.2, 125.1, 118.2, 114.5, 68.9, 43.6, 26.1, 24.8, 21.0.

HRMS (ESI) calcd for $C_{16}H_{19}O_2$ $[M + H]^+$243.1380, found 243.1379

cyclohex-2-en-1-yl(4-phenoxyphenyl)methanone (10)

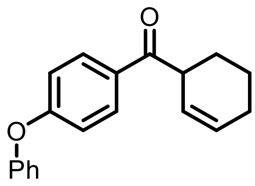

According to the *general procedure*. colorless oil (66.0mg, 79%)

^{1}H NMR (400 MHz, $CDCl_3$) *δ* 7.95 (d, *J* = 8.8 Hz, 2H), 7.39 (dd, *J* = 8.4, 7.6 Hz, 2H), 7.20 (t, *J* = 7.6 Hz, 1H), 7.07 (dd, *J* = 8.4, 0.8 Hz, 2H), 7.03 – 6.97 (m, 2H), 5.92 (ddd, *J* = 10.0, 6.0, 3.6 Hz, 1H), 5.73 (dd, *J* = 10.0, 2.0 Hz, 1H), 4.04 (ddd, *J* = 10.4, 5.6, 2.8 Hz, 1H), 2.08 (dd, *J* = 6.8, 4.4 Hz, 2H), 2.00 – 1.94 (m, 1H), 1.89 – 1.78 (m, 2H), 1.67 (dd, *J* = 4.8, 2.8 Hz, 1H).

^{13}C NMR (100 MHz, $CDCl_3$) *δ* 200.4, 161.8, 155.5, 130.8, 130.1, 124.9, 124.6, 120.2, 117.4, 43.8, 26.0, 24.8, 21.0.

HRMS (ESI) calcd for $C_{19}H_{19}O_2$ $[M + H]^+$279.1380, found 279.1379

cyclohex-2-en-1-yl(4-fluorophenyl)methanone (11)[13]

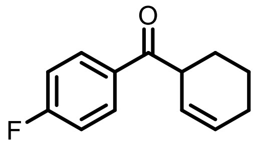

According to the *general procedure*. colorless oil (56.9mg, 93%)

^{1}H NMR (400 MHz, $CDCl_3$) *δ* 7.99 (dd, *J* = 8.4, 5.6 Hz, 2H), 7.14 (t, *J* = 8.4 Hz, 2H), 5.98 – 5.90 (m, 1H), 5.72 (dd, *J* = 10.0, 2.4 Hz, 1H), 4.04 (ddd, *J* = 10.4, 5.4, 2.8 Hz, 1H), 2.08 (dd, *J* = 7.6, 5.4 Hz, 2H), 2.00 – 1.93 (m, 1H), 1.88 – 1.80 (m, 2H), 1.74 – 1.65 (m, 1H).

^{13}C NMR (100 MHz, $CDCl_3$) *δ* 200.2, 165.6 (d, *J* = 260.0 Hz), 132.6 (d, *J* = 3.0 Hz), 131.1(d, *J* = 9.3 Hz), 130.3, 124.5, 115.7 (d, *J* = 20.0 Hz), 43.9, 25.9, 24.8, 20.9.

HRMS (ESI) calcd for $C_{13}H_{14}FO$ $[M + H]^+$205.1023, found 205.1024

(4-chlorophenyl)(cyclohex-2-en-1-yl)methanone (12)[16]

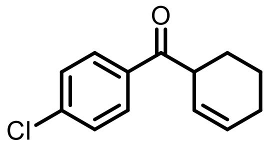

According to the *general procedure*. colorless oil (27.1mg, 41%)

^{1}H NMR (400 MHz, $CDCl_3$) *δ* 7.90 (d, *J* = 8.4 Hz, 2H), 7.44 (d, *J* = 8.4 Hz, 2H), 5.93 (ddd, *J* = 10.0, 6.0, 3.6 Hz, 1H), 5.71 (dd, *J* = 10.0, 2.0 Hz, 1H), 4.03 (dd, *J* = 5.2, 2.4 Hz, 1H), 2.12 – 2.04 (m, 2H), 1.96 (ddd, *J* = 13.2, 6.8, 3.6 Hz, 1H), 1.87 – 1.78 (m, 2H), 1.70 – 1.66 (m, 1H).

^{13}C NMR (100 MHz, $CDCl_3$) *δ* 200.6, 139.3, 134.5, 130.4, 129.9, 129.0, 124.3, 43.9, 25.8, 24.8, 20.8.

HRMS (ESI) calcd for $C_{13}H_{14}ClO$ $[M + H]^+$221.0728, found 221.0728

(4-bromophenyl)(cyclohex-2-en-1-yl)methanone (13)

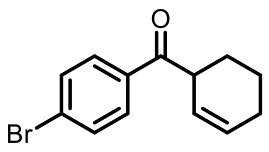

According to the *general procedure*. colorless oil (34.2 mg, 43%)

^{1}H NMR (400 MHz, $CDCl_3$) δ 7.82 (d, J = 7.2 Hz, 2H), 7.61 (d, J = 7.2 Hz, 2H), 5.98 – 5.88 (m, 1H), 5.78 – 5.64 (m, 1H), 4.08 – 3.94 (m, 1H), 2.12 – 2.04 (m, 2H), 1.99 – 1.92 (m, 1H), 1.89 – 1.79 (m, 2H), 1.73 – 1.63 (m, 1H).

^{13}C NMR (100 MHz, $CDCl_3$) δ 200.7, 134.9, 132.0, 130.4, 130.1, 128.0, 124.3, 43.9, 25.8, 24.8, 20.8.

HRMS (ESI) calcd for $C_{13}H_{14}BrO$ $[M + H]^+$265.0223, found 265.0224

cyclohex-2-en-1-yl(4-(4,4,5,5-tetramethyl-1,3,2-dioxaborolan-2-yl)phenyl)methanone (14)

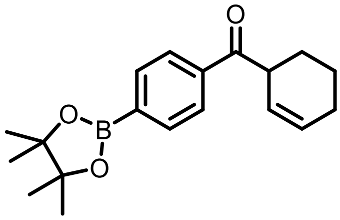

According to the *general procedure*. colorless oil (49.6mg, 53%)

^{1}H NMR (400 MHz, $CDCl_3$) δ 7.98 – 7.90 (m, 4H), 5.99 – 5.87 (m, 1H), 5.75 (dd, J = 10.0, 2.0 Hz, 1H), 4.15 – 4.02 (m, 1H), 2.09 (s, 2H), 2.03 – 1.95 (m, 1H), 1.91 – 1.80 (m, 2H), 1.75 – 1.69 (m, 1H), 1.38 (s, 12H).

^{13}C NMR (100 MHz, $CDCl_3$) δ 202.2, 138.2, 135.0, 130.2, 127.5, 124.6, 84.2, 44.0, 25.8, 24.9, 24.8, 20.9, 17.9.

HRMS (ESI) calcd for $C_{19}H_{26}BO_3$ $[M + H]^+$313.1970, found 313.1968

cyclohex-2-en-1-yl(m-tolyl)methanone (15)

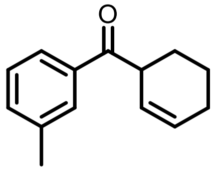

According to the *general procedure*. colorless oil (33.6 mg, 56%)

^{1}H NMR (400 MHz, $CDCl_3$) δ 7.76 (d, J = 6.4 Hz, 2H), 7.35 (d, J = 6.4 Hz, 2H), 5.97 – 5.87 (m, 1H), 5.74 (d, J = 10.0 Hz, 1H), 4.13 – 4.02 (m, 1H), 2.41 (s, 3H), 2.12 – 2.03 (m, 2H), 2.01 – 1.93 (m, 1H), 1.89 – 1.81 (m, 2H), 1.73 – 1.65 (m, 1H).

^{13}C NMR (100 MHz, $CDCl_3$) δ 202.1, 138.4, 136.3, 133.6, 130.0, 129.0, 128.5, 125.7, 124.9, 43.9, 25.9, 24.8, 21.4, 21.0.

HRMS (ESI) calcd for $C_{14}H_{17}O$ $[M + H]^+$223.1093, found 223.1093

cyclohex-2-en-1-yl(3-methoxyphenyl)methanone (16)

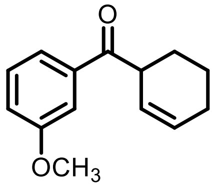

According to the *general procedure*. colorless oil (46.1 mg, 71%)

^{1}H NMR (400 MHz, $CDCl_3$) δ 7.54 (d, J = 7.6 Hz, 1H), 7.49 (dd, J = 2.4, 1.6 Hz, 1H), 7.38 (t, J = 8.0 Hz, 1H), 7.10 (ddd, J = 8.0, 2.4, 0.8 Hz, 1H), 5.96 – 5.87 (m, 1H), 5.77 – 5.70 (m, 1H), 4.06 (ddd, J = 10.8, 5.4, 2.8 Hz, 1H), 3.86 (s, 3H), 2.08 (tt, J = 8.4, 4.4 Hz, 2H), 1.96 (dd, J = 7.1, 3.2 Hz, 1H), 1.90 – 1.80 (m, 2H), 1.71 – 1.65 (m, 1H).

^{13}C NMR (100 MHz, $CDCl_3$) *δ* 201.7, 159.9, 137.6, 130.1, 129.6, 124.8, 121.0, 119.2, 112.9, 55.4, 44.0, 25.9, 24.8, 20.9.

HRMS (ESI) calcd for $C_{14}H_{17}O_2$ $[M + H]^+$217.1223, found 217.1222

methyl 3-(cyclohex-2-ene-1-carbonyl)benzoate (17)

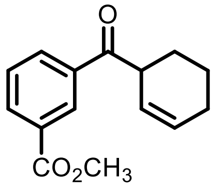

According to the *general procedure*. colorless oil (49.8 mg, 68%)

^{1}H NMR (400 MHz, $CDCl_3$) *δ* 8.59 (s, 1H), 8.23 (d, *J* = 7.6 Hz, 1H), 8.15 (d, *J* = 7.6 Hz, 1H), 7.63 – 7.49 (m, 1H), 6.00 – 5.90 (m, 1H), 5.79 – 5.69 (m, 1H), 4.18 – 4.07 (m, 1H), 3.96 (s, 3H), 2.15 – 2.04 (m, 2H), 2.00 – 1.93 (m, 1H), 1.90 – 1.79 (m, 2H), 1.76 – 1.65 (m, 1H).

^{13}C NMR (100 MHz, $CDCl_3$) *δ* 201.0, 166.4, 136.6, 133.7, 132.7, 130.7, 130.5, 129.6, 129.0, 124.3, 52.4, 44.0, 25.7, 24.8, 20.8.

HRMS (ESI) calcd for $C_{15}H_{17}O_3$ $[M + H]^+$245.1172, found 245.1171

cyclohex-2-en-1-yl(3-fluorophenyl)methanone (18)

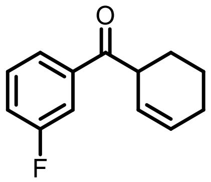

According to the *general procedure*. colorless oil (33.7 mg, 55%)

^{1}H NMR (400 MHz, $CDCl_3$) *δ* 7.74 (d, *J* = 7.6 Hz, 1H), 7.64 (d, *J* = 9.6 Hz, 1H), 7.45 (td, *J* = 8.0, 5.6 Hz, 1H), 7.29 – 7.26 (m, 1H), 5.94 (ddd, *J* = 10.0, 6.0, 3.6 Hz, 1H), 5.72 (dd, *J* = 10.0, 2.0 Hz, 1H), 4.02 (ddd, *J* = 10.4, 5.6, 2.8 Hz, 1H), 2.12 – 2.04 (m, 2H), 1.99 – 1.93 (m, 1H), 1.90 – 1.81 (m, 2H), 1.72 – 1.65 (m, 1H).

^{13}C NMR (100 MHz, $CDCl_3$) *δ* 200.5, 163.0 (d, *J* = 250.0 Hz),144.8, 138.4, 130.5, 130.1 (d, *J* = 20.0 Hz), 124.1, 119.9 (d, *J* = 20.0 Hz), 115.3 (d, *J* = 20.0 Hz), 44.1, 25.7, 24.8, 20.8.

HRMS (ESI) calcd for $C_{13}H_{14}FO$ $[M + H]^+$205.1023, found 205.1023

(3-chlorophenyl)(cyclohex-2-en-1-yl)methanone (19)

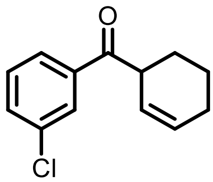

According to the *general procedure*. colorless oil (26.5 mg, 40%)

^{1}H NMR (400 MHz, $CDCl_3$) *δ* 7.92 (s, 1H), 7.83 (d, *J* = 7.6 Hz, 1H), 7.52 (t, *J* = 8.8 Hz, 1H), 7.42 (dd, *J* = 7.8, 6.0 Hz, 1H), 5.94 (d, *J* = 10.0 Hz, 1H), 5.71 (d, *J* = 10.0 Hz, 1H), 4.12 – 3.95 (m, 1H), 2.14 – 2.02 (m, 2H), 2.00 – 1.93 (m, 1H), 1.90 – 1.79 (m, 2H), 1.75 – 1.65 (m, 1H).

^{13}C NMR (100 MHz, $CDCl_3$) *δ* 200.5, 137.9, 135.0, 132.8, 130.6, 130.0, 128.6, 126.6, 124.2, 44.1, 25.7, 24.8, 20.8.

HRMS (ESI) calcd for $C_{13}H_{14}ClO$ $[M + H]^+$221.0728, found 221.0728

cyclohex-2-en-1-yl(3-(4,4,5,5-tetramethyl-1,3,2-dioxaborolan-2-yl)phenyl)methanone (20)

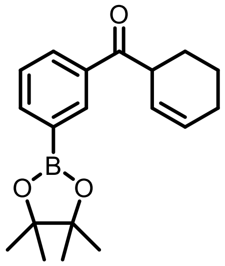

According to the *general procedure*. colorless oil (61.8 mg, 66%)

^{1}H NMR (400 MHz, $CDCl_3$) δ 8.32 (s, 1H), 8.02 (d, *J* = 7.6 Hz, 1H), 7.96 (d, *J* = 7.2 Hz, 1H), 7.45 (t, *J* = 7.6 Hz, 1H), 5.94 – 5.85 (m, 1H), 5.74 – 5.67 (m, 1H), 4.17 – 4.09 (m, 1H), 2.11 – 2.01 (m, 2H), 1.98 – 1.90 (m, 1H), 1.87 – 1.76 (m, 2H), 1.72 – 1.65 (m, 1H), 1.34 (s, 12H).

^{13}C NMR (100 MHz, $CDCl_3$) δ 202.2, 139.1, 135.8, 134.6, 131.2, 130.1, 128.1, 124.8, 84.2, 43.8, 25.7, 24.9, 24.8, 20.9.

HRMS (ESI) calcd for $C_{19}H_{26}BO_3$ $[M + H]^+$313.1970, found 313.1965

cyclohex-2-en-1-yl(2-hydroxyphenyl)methanone (21)

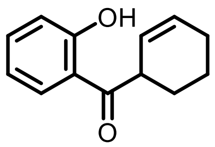

According to the *general procedure*. colorless oil (28.5 mg, 47%)

^{1}H NMR (400 MHz, $CDCl_3$) δ 12.41 (s, 1H), 7.81 (d, *J* = 8.0 Hz, 1H), 7.50 – 7.44 (m, 1H), 7.00 (d, *J* = 8.4 Hz, 1H), 6.94 – 6.88 (m, 1H), 6.02 – 5.95 (m, 1H), 5.71 (dd, *J* = 10.1, 2.1 Hz, 1H), 4.19 – 4.12 (m, 1H), 2.13 – 2.07 (m, 2H), 2.02 (dd, *J* = 6.5, 3.4 Hz, 1H), 1.92 – 1.83 (m, 2H), 1.74 – 1.66 (m, 1H).

^{13}C NMR (100 MHz, $CDCl_3$) δ 208.1, 163.4, 136.3, 130.8, 130.6, 130.1, 124.3, 118.9, 118.3, 43.5, 26.4, 24.7, 20.9.

HRMS (ESI) calcd for $C_{13}H_{15}O_2$ $[M + H]^+$203.1067, found 203.1066

cyclohex-2-en-1-yl(3,5-dimethylphenyl)methanone (22)

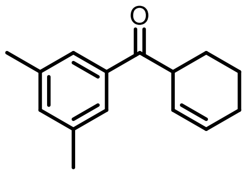

According to the *general procedure*. colorless oil (44.4 mg, 69%)

^{1}H NMR (400 MHz, $CDCl_3$) δ 7.55 (s, 2H), 7.19 (s, 1H), 5.91 (ddd, *J* = 9.6, 6.4, 3.6 Hz, 1H), 5.77 – 5.66 (m, 1H), 4.09 – 3.98 (m, 1H), 2.37 (s, 6H), 2.06 (s, 2H), 1.98 – 1.91 (m, 1H), 1.89 – 1.78 (m, 2H), 1.73 – 1.64 (m, 1H).

^{13}C NMR (100 MHz, $CDCl_3$) δ 202.3, 138.2, 136.4, 134.5, 130.0, 126.2, 125.0, 43.9, 25.9, 24.8, 21.28, 21.0.

HRMS (ESI) calcd for $C_{15}H_{19}O$ $[M + H]^+$215.1430, found 215.1430

cyclohex-2-en-1-yl(3,4-dimethylphenyl)methanone (23)

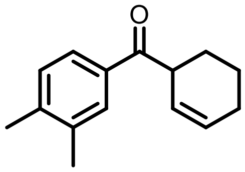

According to the *general procedure*. colorless oil (48.2 mg, 75%)

^{1}H NMR (400 MHz, $CDCl_3$) δ 7.75 – 7.67 (m, 2H), 7.21 (d, J = 7.6 Hz, 1H), 5.91 (ddd, J = 9.6, 6.0, 3.2 Hz, 1H), 5.73 (dd, J = 10.0, 2.0 Hz, 1H), 4.10 – 4.01 (m, 1H), 2.32 (s, 6H), 2.10 – 2.02 (m, 2H), 1.99 – 1.93 (m, 1H), 1.89 – 1.79 (m, 2H), 1.73 – 1.65 (m, 1H).

^{13}C NMR (100 MHz, $CDCl_3$) δ 201.8, 142.4, 137.0, 134.1, 129.9, 129.8, 129.6, 126.2, 125.1, 43.8, 26.0, 24.8, 21.0, 20.0, 19.8.

HRMS (ESI) calcd for $C_{15}H_{19}O$ [M + H]$^+$215.1430, found 215.1430

cyclohex-2-en-1-yl(3-methoxy-4-methylphenyl)methanone (24)

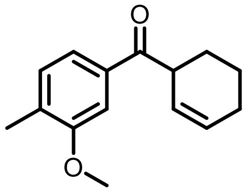

According to the *general procedure*. colorless oil (48.2 mg, 75%)

^{1}H NMR (400 MHz, $CDCl_3$) δ 7.51 – 7.45 (m, 2H), 7.22 (d, J = 7.6 Hz, 1H), 5.98 – 5.89 (m, 1H), 5.76 (dd, J = 10.0, 2.0 Hz, 1H), 4.09 (ddd, J = 10.8, 5.6, 2.8 Hz, 1H), 3.91 (s, 3H), 2.29 (s, 3H), 2.14 – 2.06 (m, 2H), 2.02 – 1.95 (m, 1H), 1.92 – 1.83 (m, 2H), 1.76 – 1.69 (m, 1H).

^{13}C NMR (100 MHz, $CDCl_3$) δ 201.5, 158.1, 135.3, 132.8, 130.4, 130.0, 125.1, 121.1, 109.1, 55.4, 43.8, 26.0, 24.8, 21.0, 16.5.

HRMS (ESI) calcd for $C_{13}H_{18}NaO_2$ [M + Na]$^+$253.1199, found 253.1195

cyclohex-2-en-1-yl(3-fluoro-4-methylphenyl)methanone (25)

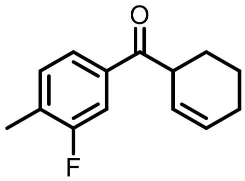

According to the *general procedure*. colorless oil (38.0 mg, 58%)

^{1}H NMR (400 MHz, $CDCl_3$) δ 7.68 – 7.56 (m, 2H), 7.29 (d, J = 7.6 Hz, 1H), 5.98 – 5.88 (m, 1H), 5.75 – 5.66 (m, 1H), 4.06 – 3.93 (m, 1H), 2.34 (s, 3H), 2.13 – 2.05 (m, 2H), 2.03 – 1.91 (m, 1H), 1.90 – 1.79 (m, 2H), 1.75 – 1.65 (m, 1H).

^{13}C NMR (100 MHz, $CDCl_3$) δ 200.4, 161.3 (d, J = 246.6 Hz), 135.9 (d, J = 5.7 Hz), 131.6 (d, J = 4.2 Hz), 130.6 (d, J = 17.6 Hz), 130.3 (d, J = 0.7 Hz), 124.5, 124.0 (d, J = 3.0 Hz), 114.9 (d, J = 24.0 Hz), 43.9, 25.9, 24.8, 20.9, 14.8, 14.8.

HRMS (ESI) calcd for $C_{14}H_{16}FO$ [M + H]$^+$219.1180, found 219.1180

cyclohex-2-en-1-yl(thiophen-2-yl)methanone (26)

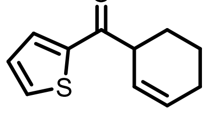

According to the *general procedure*. colorless oil (40.9 mg, 71%)

^{1}H NMR (400 MHz, $CDCl_3$) δ 7.76 (dd, *J* = 3.6, 0.8 Hz, 1H), 7.63 (dd, *J* = 4.8, 1.2 Hz, 1H), 7.14 (dd, *J* = 4.8, 3.6 Hz, 1H), 5.97 – 5.90 (m, 1H), 5.75 (dd, *J* = 10.0, 2.0 Hz, 1H), 3.92 (dtt, *J* = 8.4, 5.6, 2.8 Hz, 1H), 2.14 – 2.05 (m, 2H), 2.04 – 1.97 (m, 1H), 1.93 – 1.84 (m, 2H), 1.71 – 1.66 (m, 1H).

^{13}C NMR (100 MHz, $CDCl_3$) δ 195.0, 133.6, 132.0, 130.4, 128.1, 124.6, 45.7, 26.4, 24.7, 20.9.

HRMS (ESI) calcd for $C_{11}H_{13}OS$ [M + H]$^+$193.0682, found 193.0683

cyclohex-2-en-1-yl(6-methylpyridin-3-yl)methanone (27)

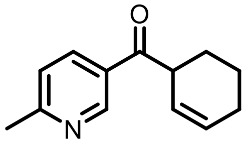

According to the *general procedure*. colorless oil (22.9 mg, 38%)

^{1}H NMR (400 MHz, $CDCl_3$) δ 9.07 (s, 1H), 8.13 (dd, *J* = 8.0, 2.0 Hz, 1H), 7.27 (d, *J* = 6.8 Hz, 1H), 5.99 – 5.90 (m, 1H), 5.73 (d, *J* = 10.0 Hz, 1H), 4.07 – 3.96 (m, 1H), 2.63 (s, 3H), 2.14 – 2.04 (m, 2H), 2.01 – 1.93 (m, 1H), 1.90 – 1.80 (m, 2H), 1.74 – 1.67 (m, 1H).

^{13}C NMR (100 MHz, $CDCl_3$) δ 200.3, 163.1, 149.6, 136.3, 130.6, 124.0, 123.4, 44.3, 25.7, 24.8, 20.8.

HRMS (ESI) calcd for $C_{13}H_{16}NO$ [M + H]$^+$202.1226, found 202.1227

cyclohex-2-en-1-yl(naphthalen-2-yl)methanone (28)

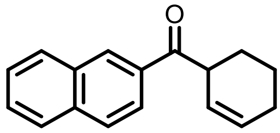

According to the *general procedure*. colorless oil (46.2 mg, 69%)

^{1}H NMR (400 MHz, $CDCl_3$) δ 8.49 (d, *J* = 3.2 Hz, 1H), 8.04 (dd, *J* = 8.4, 3.6 Hz, 1H), 8.01 – 7.96 (m, 1H), 7.94 – 7.85 (m, 2H), 7.59 (dt, *J* = 18.4, 6.0 Hz, 2H), 6.01 – 5.92 (m, 1H), 5.86 – 5.77 (m, 1H), 4.26 (d, *J* = 2.4 Hz, 1H), 2.17 – 2.09 (m, 2H), 2.07 – 2.00 (m, 1H), 1.96 – 1.84 (m, 2H), 1.78 – 1.69 (m, 1H).

^{13}C NMR (100 MHz, $CDCl_3$) δ 201.8, 135.5, 133.6, 132.6, 130.2, 129.9, 129.6, 128.5, 128.4, 127.7, 126.7, 124.9, 124.5, 44.0, 26.0, 24.8, 21.0.

HRMS (ESI) calcd for $C_{17}H_{17}O$ [M + H]$^+$237.1274, found 237.1273

(1*R*,3*R*,4*S*)-4-isopropyl-3-methylcyclohexyl 4-(cyclohex-2-ene-1-carbonyl)benzoate (29)

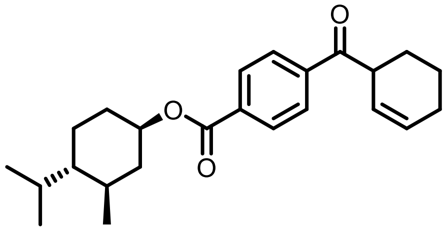

According to the *general procedure*. colorless oil (46.9 mg, 41%), dr = 1/1

^{1}H NMR (400 MHz, $CDCl_3$) δ 8.13 (d, *J* = 8.0 Hz, 2H), 7.99 (d, *J* = 8.0 Hz, 2H), 5.99 – 5.90 (m, 1H), 5.74 (s, 1H), 4.96 (dd, *J* = 10.8, 6.8 Hz, 1H), 4.13 – 4.03 (m, 1H), 2.12 (dd, *J* = 17.6, 9.4 Hz, 4H), 1.99 – 1.93 (m, 2H), 1.74 (d, *J* = 11.2 Hz, 4H), 1.17 – 1.08 (m, 4H), 0.95 – 0.91 (m, 6H), 0.80 (d, *J* = 6.8 Hz, 3H).

^{13}C NMR (100 MHz, $CDCl_3$) δ 192.2, 165.0, 158.2, 140.3, 134.9, 129.8, 127.0, 124.1, 47.2, 46.7, 40.9, 40.3, 34.0, 31.4, 26.4, 24.6, 23.6, 21.9, 20.6, 16.5, 15.6.

HRMS (ESI) calcd for $C_{24}H_{33}O_3$ [M + H]$^+$369.2424, found 369.2426

cyclopent-2-en-1-yl(*p*-tolyl)methanone (30)

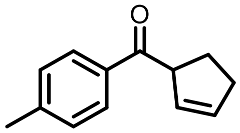

According to the *general procedure*. colorless oil (25.1 mg, 45%)

^{1}H NMR (400 MHz, $CDCl_3$) δ 7.90 (d, *J* = 8.0 Hz, 2H), 7.26 (d, *J* = 8.0 Hz, 2H), 5.95 – 5.89 (m, 1H), 5.80 – 5.72 (m, 1H), 4.47 (ddd, *J* = 11.2, 6.0, 2.4 Hz, 1H), 2.58 – 2.43 (m, 2H), 2.42 – 2.40 (m, 3H), 2.22 (s, 2H).

^{13}C NMR (100 MHz, $CDCl_3$) δ 200.5, 143.7, 133.8, 133.8, 129.3, 129.2, 128.7, 53.7, 32.4, 32.4, 26.6, 26.6, 21.6.

HRMS (ESI) calcd for $C_{13}H_{15}O$ $[M + H]^+$187.1117, found 187.1114

cyclohept-2-en-1-yl(*p*-tolyl)methanone (31)

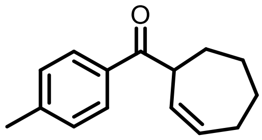

According to the *general procedure*. colorless oil (47.6 mg, 74%)

^{1}H NMR (400 MHz, $CDCl_3$) δ 7.86 (d, *J* = 8.4 Hz, 2H), 7.25 (d, *J* = 8.0 Hz, 2H), 5.96 – 5.93 (m, 1H), 4.18 (d, *J* = 10.4 Hz, 1H), 2.41 (s, 3H), 2.24 (dd, *J* = 10.0, 3.2 Hz, 2H), 2.12 – 2.06 (m, 1H), 1.97 (dd, *J* = 13.2, 2.4 Hz, 1H), 1.80 – 1.68 (m, 2H), 1.62 – 1.53 (m, 1H), 1.45 – 1.36 (m, 1H).

^{13}C NMR (100 MHz, $CDCl_3$) δ 201.5, 143.6, 133.4, 133.0, 131.3, 129.3, 128.8, 48.0, 31.0, 30.5, 28.6, 26.5, 21.6.

HRMS (ESI) calcd for $C_{15}H_{19}O$ $[M + H]^+$215.1430, found 215.1431

(*Z*)-cyclooct-2-en-1-yl(*p*-tolyl)methanone (32)

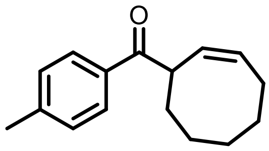

According to the *general procedure*. colorless oil (34.2 mg, 50%)

^{1}H NMR (400 MHz, $CDCl_3$) δ 7.87 (t, *J* = 7.2 Hz, 2H), 7.25 (t, *J* = 7.2 Hz, 2H), 5.80 (d, *J* = 8.0 Hz, 1H), 5.69 (d, *J* = 8.4 Hz, 1H), 4.44 – 4.29 (m, 1H), 2.42 (s, 1H), 2.41 (d, *J* = 7.2 Hz, 3H), 2.24 (s, 1H), 1.82 (dd, *J* = 21.6, 12.4 Hz, 3H), 1.72 – 1.60 (m, 3H), 1.57 – 1.35 (m, 2H).

^{13}C NMR (100 MHz, $CDCl_3$) δ 201.7, 143.6, 134.2, 131.0, 129.2, 129.1, 128.6, 44.8, 32.7, 29.4, 26.8, 26.7, 25.4, 21.6.

HRMS (ESI) calcd for $C_{16}H_{21}O$ $[M + H]^+$229.1587, found 229.1586

((2*Z*,6*Z*)-cycloocta-2,6-dien-1-yl)(*p*-tolyl)methanone (33)

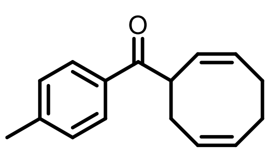

According to the *general procedure*. colorless oil (40.7mg, 60%)

^{1}H NMR (400 MHz, $CDCl_3$) δ 7.88 (d, J = 8.0 Hz, 2H), 7.27 (d, J = 7.2 Hz, 2H), 5.76 – 5.61 (m, 4H), 4.74 – 4.65 (m, 1H), 2.86 – 2.76 (m, 1H), 2.67 – 2.55 (m, 2H), 2.55 – 2.44 (m, 2H), 2.41 (s, 3H), 2.35 – 2.26 (m, 1H).

^{13}C NMR (100 MHz, $CDCl_3$) δ 200.3, 143.8, 133.9, 130.7, 129.3, 129.2, 128.7, 127.4, 127.3, 46.6, 30.9, 27.9, 27.8, 21.6.

HRMS (ESI) calcd for $C_{16}H_{19}O$ $[M + H]^+$227.1430, found 227.1431

3,4-dimethyl-1-(*p*-tolyl)pent-3-en-1-one (34)[17]

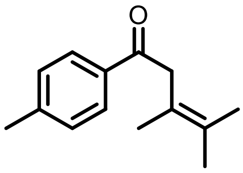

According to the *general procedure*. colorless oil (43.1 mg, 71%)

^{1}H NMR (400 MHz, $CDCl_3$) δ 7.87 (d, J = 6.4 Hz, 2H), 7.25 (d, J = 7.6 Hz, 2H), 3.72 (s, 2H), 2.41 (s, 3H), 1.74 (s, 3H), 1.69 (s, 6H).

^{13}C NMR (100 MHz, $CDCl_3$) δ 198.6, 143.6, 134.8, 129.2, 128.2, 128.0, 121.7, 44.4, 21.6, 20.8, 20.7, 19.2.

HRMS (ESI) calcd for $C_{14}H_{19}O$ $[M + H]^+$203.1430, found 203.1431

2,4-dimethyl-1-(*p*-tolyl)pent-3-en-1-one (35)[18]

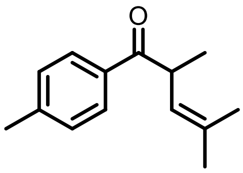

According to the *general procedure*. colorless oil (46.7 mg, 77%)

^{1}H NMR (400 MHz, $CDCl_3$) δ 7.85 (d, J = 6.4 Hz, 2H), 7.24 (d, J = 7.2 Hz, 2H), 5.21 (d, J = 9.6 Hz, 1H), 4.25 (dt, J = 6.4, 4.4 Hz, 1H), 2.40 (s, 3H), 1.75 (s, 3H), 1.70 (s, 3H), 1.24 (dd, J = 6.8, 2.0 Hz, 3H).

^{13}C NMR (100 MHz, $CDCl_3$) δ 202.1, 143.5, 134.2, 133.2, 129.2, 128.5, 125.1, 41.1, 25.8, 21.6, 18.1, 17.7.

HRMS (ESI) calcd for $C_{14}H_{19}O$ $[M + H]^+$203.1430, found 203.1432

4-methyl-1-(*p*-tolyl)pent-3-en-1-one (36)[19]

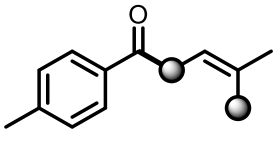

According to the *general procedure*. colorless oil (35.5 mg, 63%), rr = 2/1

^{1}H NMR (400 MHz, $CDCl_3$) δ 7.90 – 7.83 (m, 2H), 7.25 (d, J = 5.6 Hz, 2H), 5.49 – 5.33 (m, 1H), 3.70 – 3.59 (m, 2H), 2.40 (s, 3H), 1.76 (s, 2H), 1.72 (s, 1H), 1.69 (s, 2H), 1.62 (s, 1H).

^{13}C NMR (100 MHz, $CDCl_3$) δ 198.4, 143.7, 135.4, 134.4, 129.2, 129.2, 128.5, 128.4, 128.3, 123.5, 122.6, 116.5, 49.2, 41.7, 38.4, 25.8, 24.1, 21.6, 18.2, 16.2, 13.8, 13.7.

HRMS (ESI) calcd for $C_{13}H_{17}O$ $[M + H]^+$189.1274, found 189.1276

(*E*)-3,4,5-trimethyl-1-(*p*-tolyl)hex-3-en-1-one (37)

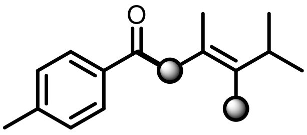

According to the *general procedure*. colorless oil (30.9 mg, 45%), rr = 2.3/1

^{1}H NMR (400 MHz, $CDCl_3$) δ 7.88 (dd, J = 16.0, 8.4 Hz, 2H), 7.24 (d, J = 8.0 Hz, 2H), 3.77 – 3.63 (m, 2H), 3.01 – 2.69 (m, 1H), 2.41 (s, 3H), 1.70 – 1.63 (m, 3H), 1.61 – 1.51 (m, 3H), 0.98 – 0.94 (m, 3H), 0.93 – 0.83 (m, 3H).

^{13}C NMR (100 MHz, $CDCl_3$) δ 198.6, 198.5, 143.6, 137.2, 137.0, 134.8, 130.6, 129.2, 129.2, 128.2, 128.1, 127.9, 120.6, 120.2, 45.0, 43.6, 36.9, 30.6, 30.0, 29.9, 21.6, 20.9, 20.7, 20.5, 19.9, 18.4, 12.4, 12.3.

HRMS (ESI) calcd for $C_{16}H_{23}O$ $[M + H]^+$231.1743, found 231.1743

2,2,3,4-tetramethyl-1-(*p*-tolyl)pent-3-en-1-one (37')

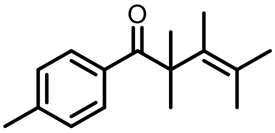

According to the *general procedure*. colorless oil (15.4 mg, 22%)

^{1}H NMR (400 MHz, $CDCl_3$) δ 7.96 (d, J = 8.0 Hz, 2H), 7.24 (d, J = 8.0 Hz, 2H), 2.41 (s, 3H), 1.58 (s, 3H), 1.46 – 1.36 (m, 3H), 1.15 – 1.13 (m, 9H).

^{13}C NMR (100 MHz, $CDCl_3$) δ 200.1, 166.4, 143.5, 130.2, 129.1, 128.8, 128.6, 29.7, 21.7, 17.9, 14.1, 12.1.

HRMS (ESI) calcd for $C_{16}H_{23}O$ $[M + H]^+$231.1743, found 231.1743

1-(*p*-tolyl)-2-vinylnonan-1-one (38)

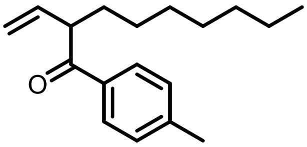

According to the *general procedure*. colorless oil (51.9 mg, 67%)

^{1}H NMR (400 MHz, $CDCl_3$) δ 7.87 (d, J = 6.4 Hz, 2H), 7.30 – 7.11 (m, 3H), 5.75 – 5.52 (m, 2H), 3.66 (d, J = 4.4 Hz, 1H), 2.41 (s, 3H), 2.12 – 2.02 (m, 2H), 1.35 – 1.18 (m, 10H), 0.93 – 0.84 (m, 3H).

^{13}C NMR (100 MHz, $CDCl_3$) δ 198.4, 143.8, 135.0, 129.2, 128.5, 122.4, 110.0, 42.5, 32.7, 31.8, 29.2, 29.2, 29.1, 22.7, 21.6, 14.1.

HRMS (ESI) calcd for $C_{18}H_{27}O$ $[M + H]^+$259.2056, found 259.2058

2-methoxy-4-methyl-1-(*p*-tolyl)pent-3-en-1-one (39)

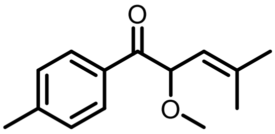

According to the *general procedure*. colorless oil (39.9 mg, 61%)

^{1}H NMR (400 MHz, $CDCl_3$) δ 7.94 (d, J = 8.4 Hz, 2H), 7.25 (d, J = 8.4 Hz, 2H), 5.31 (dd, J = 8.8, 1.2 Hz, 1H), 5.08 (d, J = 8.8 Hz, 1H), 3.38 (s, 3H), 2.41 (s, 3H), 1.84 (s, 3H), 1.76 (s, 3H).

^{13}C NMR (100 MHz, $CDCl_3$) δ 197.7, 144.1, 140.8, 132.5, 129.2, 129.0, 120.6, 82.4, 56.6, 26.1, 21.7, 18.8.

HRMS (EI) calcd for $C_{14}H_{18}NaO_2$ $[M + Na]^+$241.1199, found 241.1205

2-(hex-5-en-1-yloxy)-2-phenyl-1-(*p*-tolyl)ethan-1-one (40)

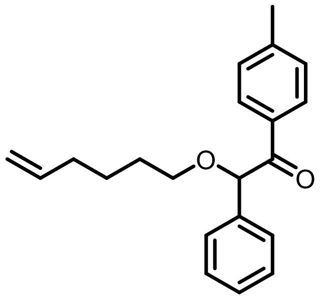

According to the *general procedure*. colorless oil (43.2 mg, 70%)

^{1}H NMR (400 MHz, $CDCl_3$) δ 7.98 (d, J = 6.0 Hz, 2H), 7.52 (d, J = 6.4 Hz, 2H), 7.36 (s, 3H), 7.21 (d, J = 6.1 Hz, 2H), 5.90 – 5.74 (m, 1H), 5.56 (d, J = 1.2 Hz, 1H), 4.99 (t, J = 14.8 Hz, 2H), 3.68 – 3.51 (m, 2H), 2.38 (s, 3H), 2.12 – 2.03 (m, 2H), 1.77 – 1.66 (m, 2H), 1.56 – 1.46 (m, 2H).

^{13}C NMR (100 MHz, $CDCl_3$) δ 197.5, 144.0, 137.0, 132.4, 129.5, 129.4, 129.1, 128.7, 128.2, 127.2, 114.6, 85.7, 69.9, 33.5, 29.2, 25.4, 21.7.

HRMS (EI) calcd for $C_{21}H_{24}NaO_2$ [M + Na]$^+$331.1669, found 331.1670

2-phenyl-1-(*p*-tolyl)ethan-1-one (41)[20]

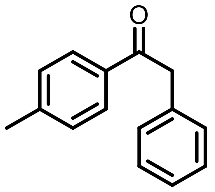

According to the *general procedure*. white solid (42.9 mg, 68%) M.p. = 65 – 67 °C

^{1}H NMR (400 MHz, $CDCl_3$) δ 7.92 (d, J = 6.4 Hz, 2H), 7.34 – 7.29 (m, 2H), 7.28 – 7.21 (m, 5H), 4.26 (s, 2H), 2.40 (s, 3H).

^{13}C NMR (100 MHz, $CDCl_3$) δ 197.3, 144.0, 134.8, 134.1, 129.4, 129.3, 128.8, 128.6, 126.8, 45.4, 21.7.

HRMS (ESI) calcd for $C_{15}H_{15}O$ [M + H]$^+$211.1117, found 211.1118

2,2-diphenyl-1-(*p*-tolyl)ethan-1-one (42)[21]

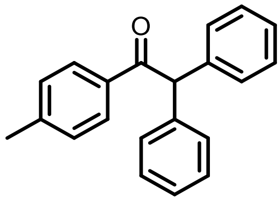

According to the *general procedure*. white solid (66.2 mg, 77%) M.p. = 99 – 101 °C

^{1}H NMR (400 MHz, $CDCl_3$) δ 7.91 (d, J = 4.8 Hz, 2H), 7.34 – 7.22 (m, 10H), 7.22 – 7.17 (m, 2H), 6.02 (s, 1H), 2.36 (s, 3H).

^{13}C NMR (100 MHz, $CDCl_3$) δ 197.8, 143.9, 139.3, 134.3, 129.3, 129.2, 129.1, 128.7, 127.1, 59.3, 21.6.

HRMS (ESI) calcd for $C_{21}H_{19}O$ [M + H]$^+$287.1430, found 287.1430

2-methyl-2-phenyl-1-(*p*-tolyl)propan-1-one (43)[22]

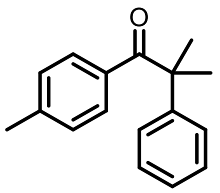

According to the *general procedure*. white solid (47.2 mg, 66%) M.p. = 39 – 41 °C

^{1}H NMR (400 MHz, $CDCl_3$) δ 7.42 (d, J = 8.0 Hz, 2H), 7.35 – 7.29 (m, 4H), 7.24 (dd, J = 7.1, 2.0 Hz, 1H), 7.02 (d, J = 8.0 Hz, 2H), 2.28 (s, 3H), 1.60 (s, 6H).

^{13}C NMR (100 MHz, $CDCl_3$) δ 203.2, 145.7, 142.3, 133.4, 130.0, 129.0, 128.6, 126.7, 125.7, 51.4, 28.0, 21.4.

HRMS (ESI) calcd for $C_{17}H_{18}NaO$ $[M + Na]^+$261.1250, found 261.1250

3-((1*S*,2*R*,4*R*)-2-methoxy-4-methylcyclohexyl)-1-(*p*-tolyl)but-3-en-1-one (44)

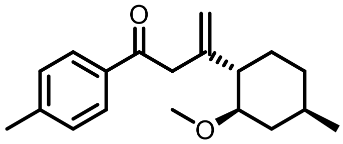

According to the *general procedure*. colorless oil (50.7 mg, 59%)

^{1}H NMR (400 MHz, $CDCl_3$) δ 7.91 (d, J = 8.0 Hz, 2H), 7.24 (d, J = 8.0 Hz, 2H), 5.02 (s, 1H), 4.82 (s, 1H), 3.70 (q, J = 15.6 Hz, 2H), 3.31 (s, 3H), 3.10 (td, J = 10.4, 3.6 Hz, 1H), 2.40 (s, 3H), 2.17 (d, J = 12.4 Hz, 1H), 2.07 – 1.99 (m, 1H), 1.83 – 1.76 (m, 1H), 1.66 (d, J = 12.8 Hz, 1H), 1.45 – 1.32 (m, 2H), 0.94 (d, J = 6.4 Hz, 3H), 0.84 (dd, J = 23.2, 12.0 Hz, 2H).

^{13}C NMR (100 MHz, $CDCl_3$) δ 198.9, 147.4, 144.0, 134.4, 129.2, 128.8, 113.3, 109.8, 83.0, 56.0, 50.7, 46.0, 39.4, 34.6, 31.4, 31.3, 22.3, 21.6.

HRMS (ESI) calcd for $C_{19}H_{26}NaO$ $[M + Na]^+$309.1825, found 309.1830

2-(4-methyl-5-(4-methylbenzoyl)cyclohex-3-en-1-yl)propan-2-yl acetate (45)

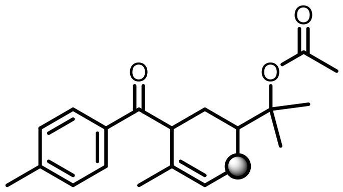

According to the *general procedure*. colorless oil (50.4 mg, 49%), isomers ratios: 3.3: 3.3: 2.4 : 1.

^{1}H NMR (400 MHz, $CDCl_3$) δ 7.98 – 7.84 (m, 2H), 7.26 (dd, J = 19.6, 8.4 Hz, 2H), 5.81 – 5.58 (m, 0.67H), 4.64 (d, J = 4.0 Hz, 0.27H), 4.27 – 3.93 (m, 0.69H), 3.87 – 3.35 (m, 0.24H), 2.42 (s, 3H), 2.02 (d, J = 6.4 Hz, 1H), 1.97 (d, J = 11.2 Hz, 3H), 1.72 (dd, J = 21.6, 10.8 Hz, 3H), 1.62 – 1.49 (m, 3H), 1.42 (d, J = 3.2 Hz, 3H), 1.39 – 1.32 (m, 2H), 1.07 – 1.00 (m, 2H).

^{13}C NMR (101 MHz, $CDCl_3$) δ 202.7, 201.2, 170.5, 144.1, 134.4, 132.0, 129.8, 129.4, 129.3, 128.6, 128.5, 124.8, 84.4, 83.7, 62.6, 49.5, 47.8, 42.0, 38.8, 37.3, 29.5, 27.5, 26.4, 26.3, 23.5, 23.4, 23.1, 22.9, 22.5, 22.5, 22.2, 21.6, 21.3, 17.7, 12.3.

HRMS (ESI) calcd for $C_{20}H_{26}NaO_3$ $[M + Na]^+$337.1774, found 337.1773

(*R*)-3-(4-methylcyclohex-3-en-1-yl)-1-(*p*-tolyl)but-3-en-1-one (46)

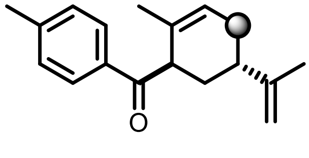

According to the *general procedure*. colorless oil (49.6 mg, 65%), isomers ratios: 5: 3.1: 2 : 1.

^{1}H NMR (400 MHz, $CDCl_3$) δ 7.91 (dd, J = 19.6, 8.4 Hz, 2H), 7.26 (d, J = 2.0 Hz, 2H), 5.77 (s, 0.18H), 5.70 (s, 0.28H), 5.29 (s, 0.45H), 4.76 – 4.64 (m, 2H), 4.64 (s, 0.09H), 4.21 – 4.01 (m, 1H), 2.81 – 2.75 (m, 0.42H), 2.41 (s, 3H), 2.33 – 2.24 (m, 0.63H), 2.20 – 1.99 (m, 2H), 1.96 – 1.84 (m, 1H), 1.72 (s, 3H), 1.66 – 1.59 (m, 3H).

^{13}C NMR (100 MHz, $CDCl_3$) δ 202.6, 201.1, 149.1, 147.9, 144.0, 143.6, 136.6, 134.3, 131.9, 129.4, 129.3, 128.7, 128.6, 125.3, 125.2, 118.6, 110.4, 109.3, 108.9, 48.5, 42.4, 41.1, 33.5, 30.8, 29.9, 27.3, 23.7, 21.6, 21.5, 21.2, 20.5.

HRMS (ESI) calcd for $C_{18}H_{23}O$ $[M + H]^+$255.1743, found 255.1746

***p*-tolyl(3,7,7-trimethylbicyclo[4.1.0]hept-3-en-2-yl)methanone (47)**

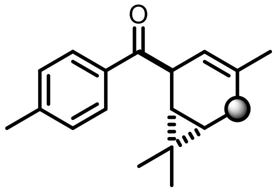

According to the *general procedure*. colorless oil (36.8 mg, 51%), isomers ratios: 17: 1: 1 : 1.

^{1}H NMR (400 MHz, $CDCl_3$) δ 7.92 (d, J = 8.0 Hz, 2H), 7.26 (d, J = 8.0 Hz, 2H), 5.76 (s, 0.05H), 5.43 (s, 0.1H), 5.33 (s, 0.85H), 3.79 (s, 1H), 3.63 (s, 0.05H), 3.62 (s, 0.05H), 3.50 (s, 0.05H), 2.41 (s, 3H), 2.34 (d, J = 18.8 Hz, 1H), 1.90 (dd, J = 13.6, 6.0 Hz, 1H), 1.65 (s, 3H), 1.08 (d, J = 4.0 Hz, 3H), 0.94 (d, J = 4.0 Hz, 2H), 0.92 (s, 3H).

^{13}C NMR (100 MHz, $CDCl_3$) δ 201.2, 143.5, 135.2, 133.6, 129.2, 129.1, 117.6, 42.2, 28.2, 25.1, 23.8, 21.6, 20.5, 19.0, 17.4, 13.8.

HRMS (ESI) calcd for $C_{17}H_{21}O$ $[M + H]^+$241.1587, found 241.1588

(3,4,7,7-tetrahydro-1H-4,7-methanoinden-1-yl)(*p*-tolyl)methanone (48)

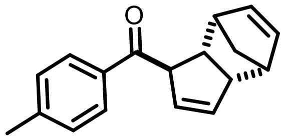

According to the *general procedure*. colorless oil (39.8 mg, 53%), dr > 20/1

^{1}H NMR (400 MHz, $CDCl_3$) δ 7.88 (d, J = 8.4 Hz, 2H), 7.29 – 7.26 (m, 2H), 6.14 (dd, J = 5.6, 2.4 Hz, 1H), 6.05 (dd, J = 5.6, 3.0 Hz, 1H), 5.72 (dd, J = 5.2, 2.4 Hz, 1H), 5.57 – 5.53 (m, 1H), 3.72 (d, J = 2.4 Hz, 1H), 3.35 (dd, J = 4.0, 2.0 Hz, 1H), 3.12 – 3.03 (m, 2H), 2.86 (s, 1H), 2.42 (s, 3H), 1.57 (d, J = 8.0 Hz, 1H), 1.38 (d, J = 8.0 Hz, 1H).

^{13}C NMR (100 MHz, $CDCl_3$) δ 200.8, 143.6, 136.7, 135.3, 132.1, 130.8, 129.3, 128.8, 109.9, 56.7, 54.8, 50.5, 45.8, 45.2, 44.8, 21.6.

HRMS (ESI) calcd for $C_{18}H_{18}NaO$ $[M + Na]^+$273.1250, found 273.1255

(2-isopropyl-5-methylcyclohexa-2,5-dien-1-yl)(*p*-tolyl)methanone (49)

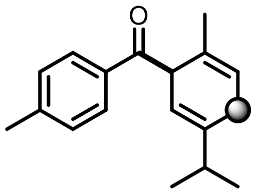

According to the *general procedure*. colorless oil (48.1 mg, 63%), rr = 4.8/1

^{1}H NMR (400 MHz, $CDCl_3$) *δ* 7.93 (d, *J* = 7.6 Hz, 2H), 7.24 (d, *J* = 7.6 Hz, 2H), 5.76 – 5.71 (m, 1H), 5.50 – 5.43 (m, 1H), 4.74 – 4.57 (m, 0.17H), 4.48 – 4.38 (m, 0.83H), 2.81 – 2.67 (m, 2H), 2.41 (s, 3H), 2.27 – 2.21 (m, 1H), 1.67 – 1.62 (m, 3H), 1.01 – 0.97 (m, 6H).

^{13}C NMR (100 MHz, $CDCl_3$) *δ* 199.2, 143.7, 143.3, 133.9, 130.4, 130.2, 129.2, 128.9, 122.1, 115.4, 52.4, 27.7, 23.9, 21.9, 21.7, 21.2, 21.0.

HRMS (ESI) calcd for $C_{18}H_{23}O$ $[M + H]^+$255.1743, found 255.1742

***p*-tolyl(5-vinylcyclohex-2-en-1-yl)methanone compound with *p*-tolyl(6-vinylcyclohex-2-en-1-yl)methanone (50)**

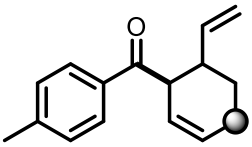

According to the *general procedure*. colorless oil (31.9 mg, 45%), isomers ratios: 5.5: 4.6: 1.4 : 1.

^{1}H NMR (400 MHz, $CDCl_3$) *δ* 7.87 (t, *J* = 6.4 Hz, 2H), 7.27 (d, *J* = 8.0 Hz, 2H), 5.97 – 5.54 (m, 3H), 5.10 – 4.91 (m, 2H), 4.23 – 3.94 (m, 1H), 2.59 – 2.44 (m, 1H), 2.42 (s, 3H), 2.30 – 2.01 (m, 2H), 2.00 – 1.85 (m, 1H), 1.78 – 1.58 (m, 1H).

^{13}C NMR (100 MHz, $CDCl_3$) *δ* 201.3, 200.8, 143.8, 143.7, 142.9, 142.5, 133.6, 129.4, 128.7, 128.6, 125.2, 124.3, 113.4, 113.2, 45.0, 42.1, 37.7, 34.0, 32.0, 31.1, 30.5, 30.2, 26.6, 21.6.

HRMS (ESI) calcd for $C_{16}H_{19}O$ $[M + H]^+$227.1430, found 227.1433

4*S*-6-methoxy-4-methyl-2-(2-methylprop-1-en-1-yl)-1-(*p*-tolyl)hexan-1-one (51)

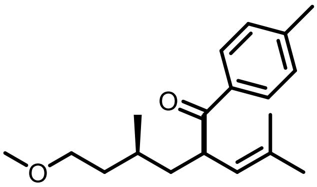

According to the *general procedure*. colorless oil (40.3 mg, 49%), dr = 2/1

^{1}H NMR (400 MHz, $CDCl_3$) *δ* 7.85 (d, *J* = 4.4 Hz, 2H), 7.26 (d, *J* = 4.4 Hz, 2H), 5.11 (s, 1H), 4.31 (s, 1H), 3.44 – 3.37 (m, 2H), 3.33 – 3.29 (m, 3H), 2.42 – 2.38 (m, 3H), 2.01 – 1.84 (m, 1H), 1.77 – 1.72 (m, 3H), 1.70 (s, 3H), 1.61 (s, 2H), 1.42 – 1.31 (m, 2H), 0.95 – 0.88 (m, 3H).

^{13}C NMR (100 MHz, $CDCl_3$) *δ* 201.8, 201.7, 143.6, 137.5, 134.7, 134.6, 134.0, 133.8, 129.7, 129.3, 128.5, 128.4, 124.4, 124.1, 71.1, 70.9, 58.6, 44.5, 40.3, 40.1, 37.0, 36.6, 28.0, 27.8, 25.9, 25.9, 21.6, 20.2, 19.8, 18.4.

HRMS (ESI) calcd for $C_{19}H_{28}NaO_2$ $[M + Na]^+$ 311.1982, found 311.1985

cyclohex-2-en-1-yl(*p*-tolyl)methanone oxime (52)

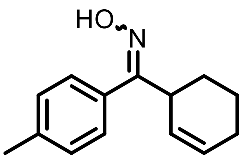

colorless oil (35.7 mg, 83%); 1.26:1 mixture of (E,Z) oxime isomers.

^{1}H NMR (400 MHz, $CDCl_3$) *δ* 7.41 (d, *J* = 7.2 Hz, 2H), 7.24 (d, *J* = 9.2 Hz, 2H), 7.12 (d, *J* = 7.2 Hz, 2H), 5.89 – 5.74 (m, 2H), 5.60 (d, *J* = 10.0 Hz, 1H), 4.37 – 4.25 (m, 1H), 3.41 – 3.33 (m, 1H), 2.36 (s, 2H), 2.34 (s, 3H), 2.12 – 2.06 (m, 2H), 2.03 – 1.98 (m, 2H), 1.86 – 1.77 (m, 2H), 1.73 – 1.65 (m, 3H), 1.58 – 1.54 (m, 1H).

^{13}**C NMR** (100 MHz, $CDCl_3$) δ 162.2, 161.2, 138.6, 132.7, 130.4, 129.1, 128.9, 128.8, 128.0, 127.9, 127.7, 127.5, 126.6, 41.7, 35.1, 26.9, 26.0, 25.0, 24.7, 22.2, 21.4, 21.3, 20.6.

HRMS (ESI) calcd for $C_{14}H_{18}NO$ [M + H]$^+$216.1383, found 216.1384

3-(*p*-tolyl)-3a,4,5,6,7,7a-hexahydrobenzo[*d*]isoxazole (53)

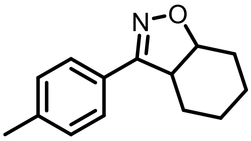

white solid (21.1 mg, 49%)

^{1}**H NMR** (400 MHz, $CDCl_3$) δ 7.60 (d, J = 7.6 Hz, 2H), 7.21 (d, J = 7.6 Hz, 2H), 4.53 – 4.44 (m, 1H), 3.24 (dd, J = 15.6, 7.2 Hz, 1H), 2.38 (s, 3H), 2.27 (d, J = 15.2 Hz, 1H), 2.00 – 1.93 (m, 1H), 1.80 – 1.67 (m, 2H), 1.58 – 1.39 (m, 2H), 1.26 – 1.22 (m, 2H).

^{13}**C NMR** (100 MHz, $CDCl_3$) δ 163.9, 140.2, 129.5, 126.9, 126.6, 44.6, 29.7, 26.5, 25.1, 22.4, 21.4, 20.3.

HRMS (ESI) calcd for $C_{14}H_{18}NO$ [M + H]$^+$216.1383, found 216.1386

cyclohexyl(*p*-tolyl)methanone (54)[23]

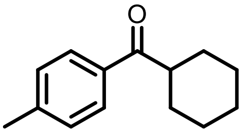

white solid (32.7 mg, 81%)

^{1}**H NMR** (400 MHz, $CDCl_3$) δ 7.85 (d, J = 8.0 Hz, 2H), 7.25 (d, J = 8.0 Hz, 2H), 3.30 – 3.14 (m, 1H), 2.41 (s, 3H), 1.85 (t, J = 11.6 Hz, 3H), 1.77 – 1.70 (m, 1H), 1.49 (dd, J = 24.0, 12.4 Hz, 2H), 1.37 (dd, J = 16.8, 7.6 Hz, 2H), 1.31 – 1.17 (m, 2H).

^{13}**C NMR** (100 MHz, $CDCl_3$) δ 203.6, 143.5, 133.8, 129.3, 128.4, 45.5, 29.5, 26.0, 25.9, 21.6.

HRMS (ESI) calcd for $C_{14}H_{19}O$ [M + H]$^+$ 203.1430, found 203.1432

1-(1-(cyclohex-2-en-1-yl)vinyl)-4-methylbenzene (55)

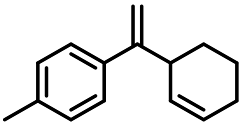

colorless oil (31.7 mg, 80%)

^{1}**H NMR** (400 MHz, $CDCl_3$) δ 7.29 (d, J = 6.6 Hz, 2H), 7.13 (d, J = 6.8 Hz, 2H), 5.83 (d, J = 9.5 Hz, 1H), 5.71 (d, J = 9.7 Hz, 1H), 5.27 (s, 1H), 5.01 (s, 1H), 3.36 (m, 1H), 2.34 (s, 3H), 2.02 (m, 2H), 1.80 (m, 1H), 1.66 (m, 1H), 1.48 (m, 16.0 Hz, 2H).

^{13}**C NMR** (100 MHz, $CDCl_3$) δ 152.2, 139.1, 136.9, 129.8, 128.9, 128.2, 126.5, 112.4, 39.8, 28.5, 25.3, 21.1, 20.4.

HRMS (EI) calcd for $C_{15}H_{18}$ [M]$^+$198.1409, found 198.1402

cyclohex-2-en-1-yl(*p*-tolyl)methanol (56)[24]

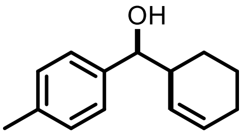 (major) 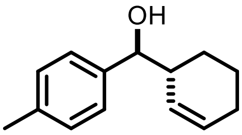 (minor)

colorless oil (30.7 mg, 76%), dr = 2.3/1

^{1}H NMR (400 MHz, $CDCl_3$) δ 7.22 (d, J = 8.0 Hz, 2H), 7.15 (d, J = 8.0 Hz, 2H), 5.85 (s, 0.6H, minor), 5.82 – 5.72 (m, 0.7H, major), 5.41 – 5.30 (m, 0.7H, major), 4.52 (d, J = 6.7 Hz, 0.7H, major), 4.41 (d, J=7.0 Hz, 0.3H, minor), 2.52 – 2.41 (m, 1H), 2.34 (s, 3H), 2.02 – 1.94 (m, 2H), 1.90 – 1.81 (m, 1H), 1.77 – 1.65 (m, 2H), 1.55 – 1.48 (m, 2H).

^{13}C NMR (100 MHz, $CDCl_3$) δ 140.6, 140.0, 137.1, 137.0, 130.2, 129.7, 129.0, 128.9, 128.1, 127.3, 126.5, 126.2, 43.0, 42.8, 26.3, 25.3, 25.3, 24.1, 21.5, 21.1.

HRMS (ESI) calcd for $C_{14}H_{18}NaO$ $[M + Na]^+$ 225.1250, found 225.1250

***N*-(cyclohex-2-en-1-yl(*p*-tolyl)methyl)-4-methoxyaniline (57)**

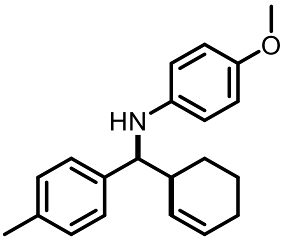
(major)

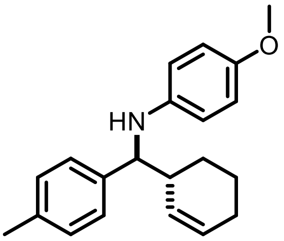
(minor)

yellow oil (48.5 mg, 79%), dr = 2.3/1

^{1}H NMR (400 MHz, $CDCl_3$) δ 7.23 (dd, J = 12.8, 4.8 Hz, 2H), 7.11 (d, J = 7.2 Hz, 2H), 6.70 – 6.63 (m, 2H), 6.48 – 6.40 (m, 2H), 5.89 – 5.81 (m, 1H), 5.63 (d, J = 10.4 Hz, 0.3H, minor), 5.52 (d, J = 10.4 Hz, 0.7H, major), 4.18 (d, J = 4.4 Hz, 1H), 3.67 (s, 3H), 2.61 – 2.50 (m, 1H), 2.32 (s, 3H), 2.04 – 1.94 (m, 2H), 1.81 – 1.69 (m, 2H), 1.53 – 1.44 (m, 2H).

^{13}C NMR (100 MHz, $CDCl_3$) δ 136.2, 130.7, 130.3, 129.4, 129.0, 129.0, 126.9, 126.8, 126.5, 114.8, 114.7, 114.0, 62.6, 55.8, 55.8, 43.2, 43.0, 27.7, 25.3, 23.8, 22.0, 21.9, 21.1.

HRMS (ESI) calcd for $C_{21}H_{26}NO$ $[M + H]^+$ 308.2009, found 308.2007

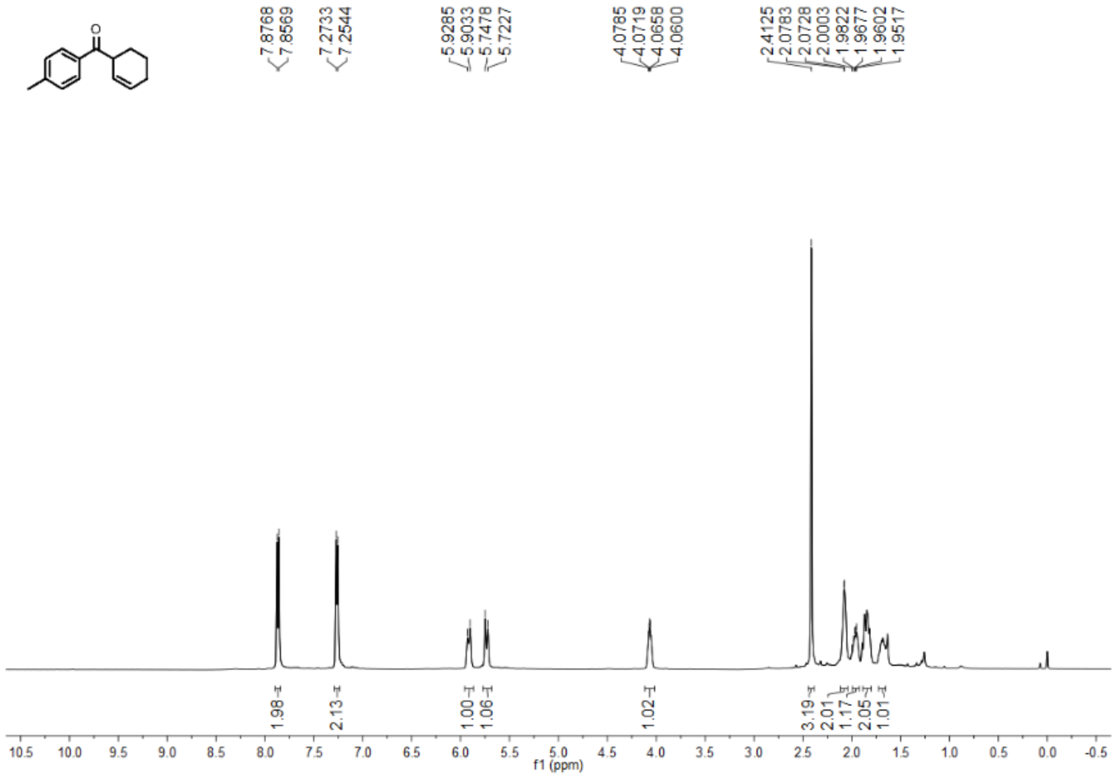

Supplementary Figure 13 ^{1}H NMR (400 MHz) spectrum of compound **3** in $CDCl_3$

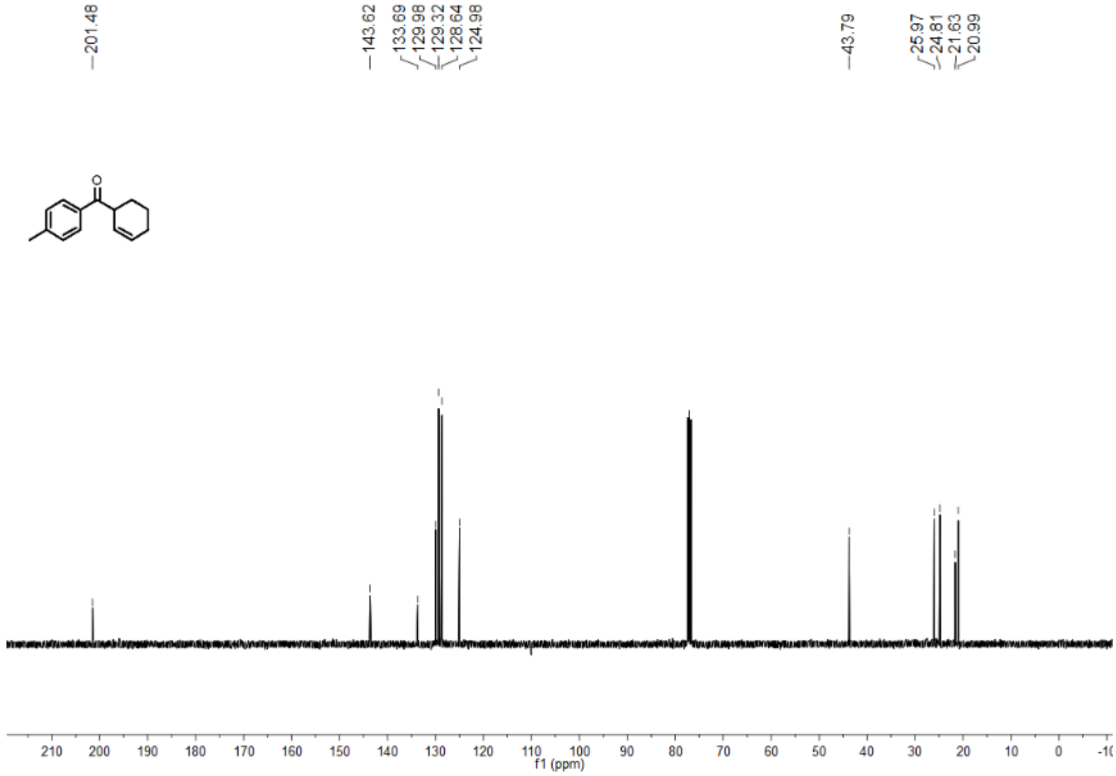

Supplementary Figure 14 ^{13}C NMR (100 MHz) spectrum of compound **3** in $CDCl_3$

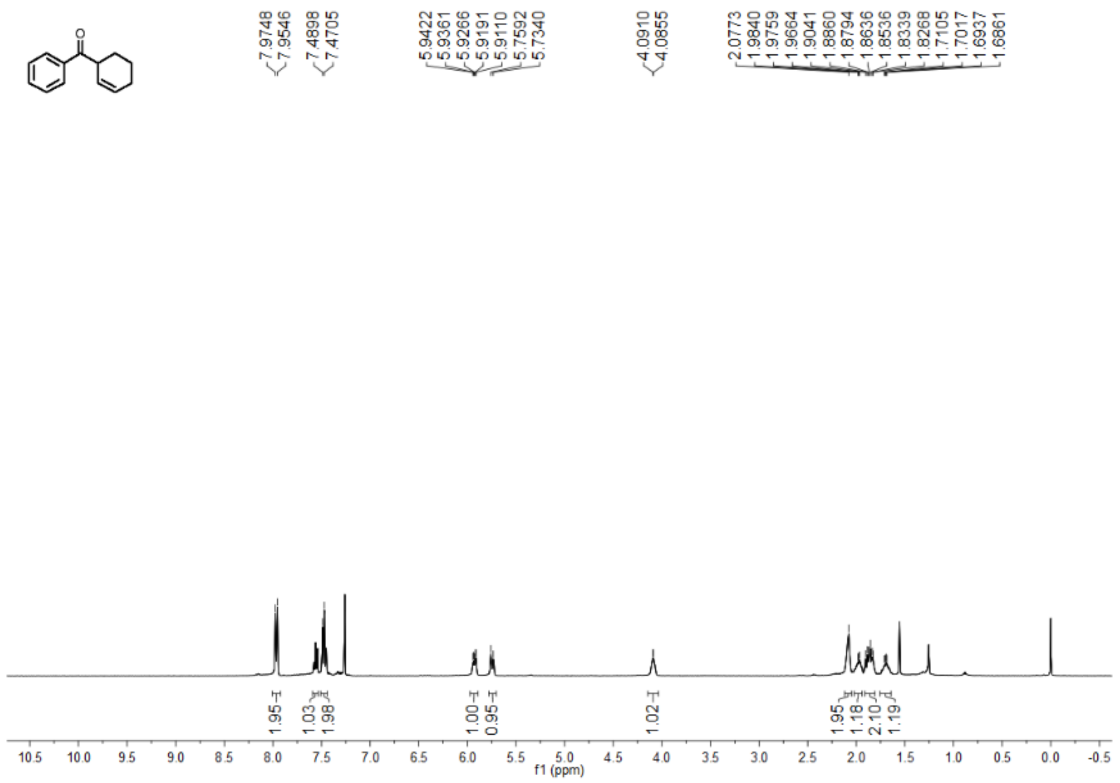

Supplementary Figure 15 ^{1}H NMR (400 MHz) spectrum of compound **4** in $CDCl_3$

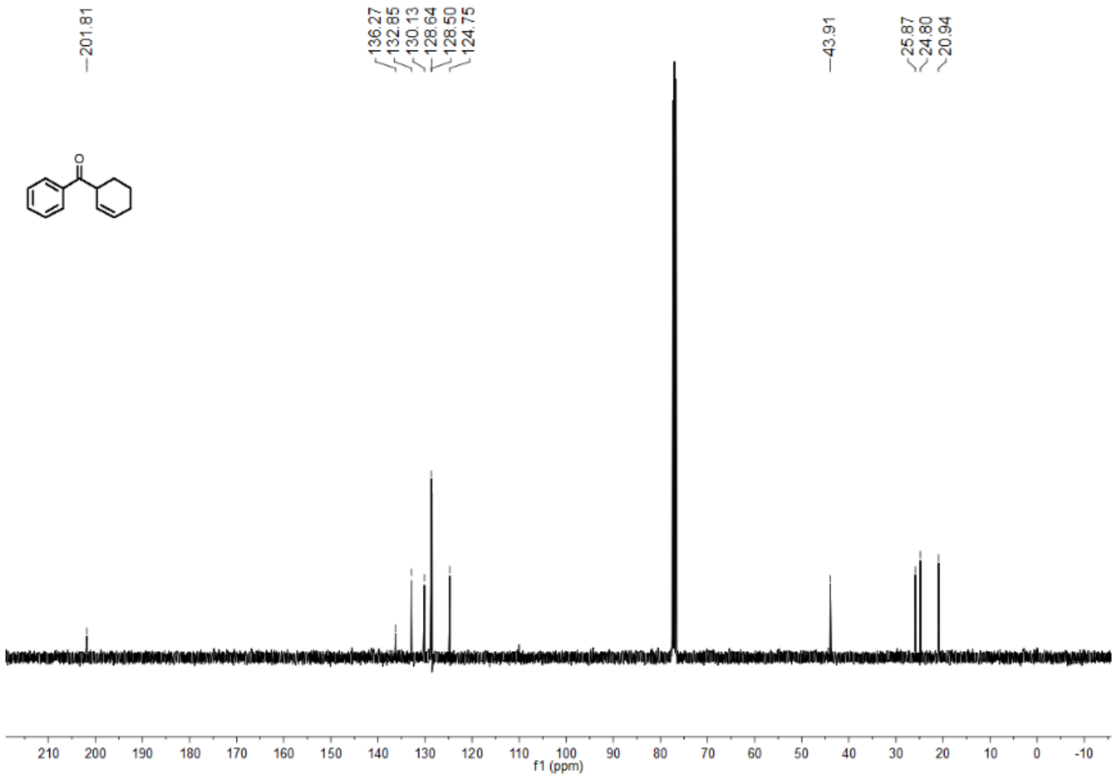

Supplementary Figure 16 ^{13}C NMR (100 MHz) spectrum of compound **4** in $CDCl_3$

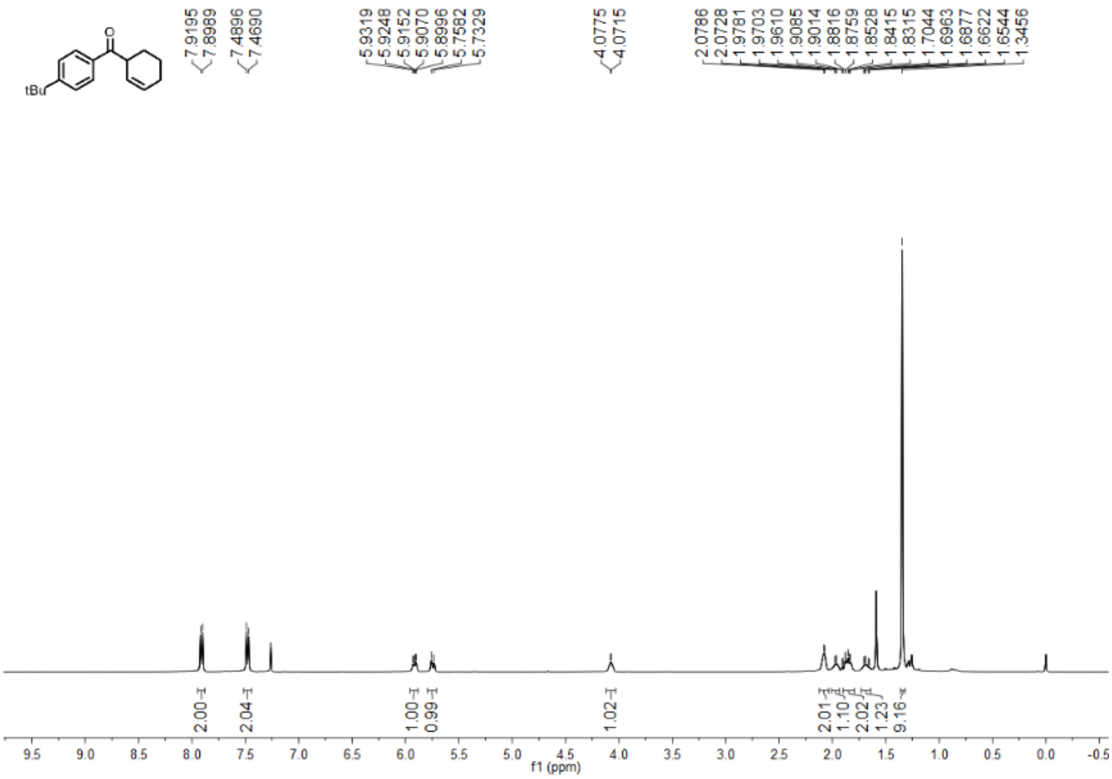

Supplementary Figure 17 ^{1}H NMR (400 MHz) spectrum of compound **5** in $CDCl_3$

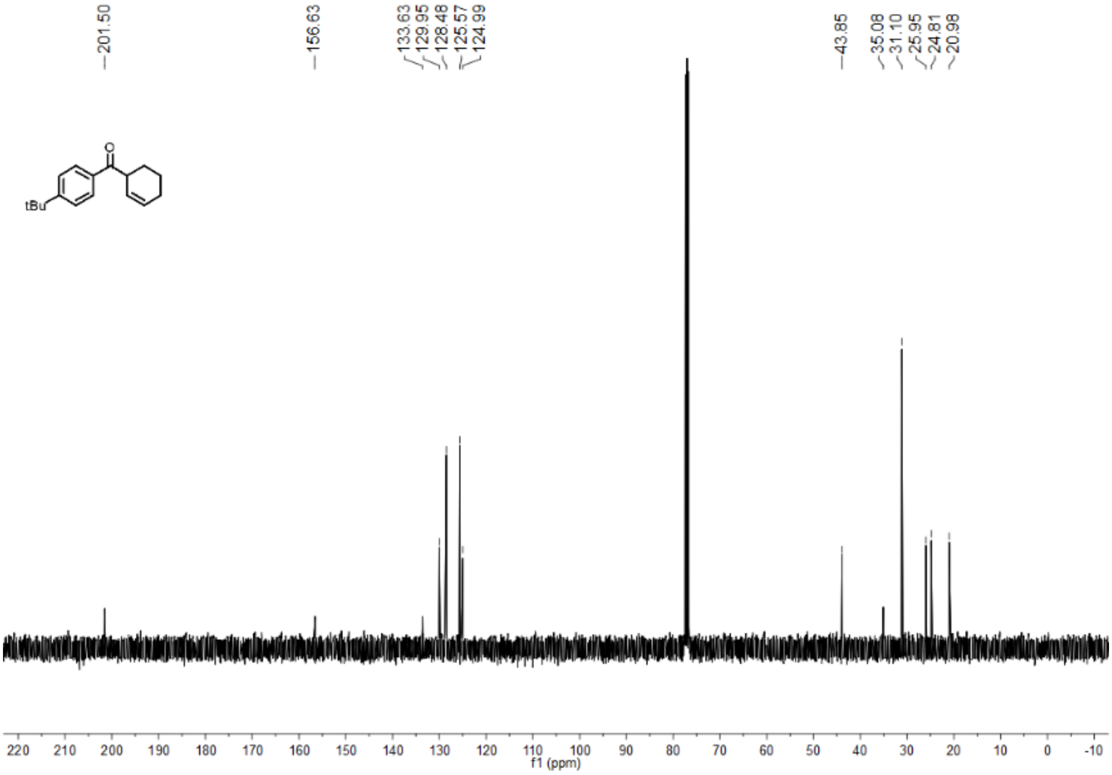

Supplementary Figure 18 ^{13}C NMR (100 MHz) spectrum of compound **5** in $CDCl_3$

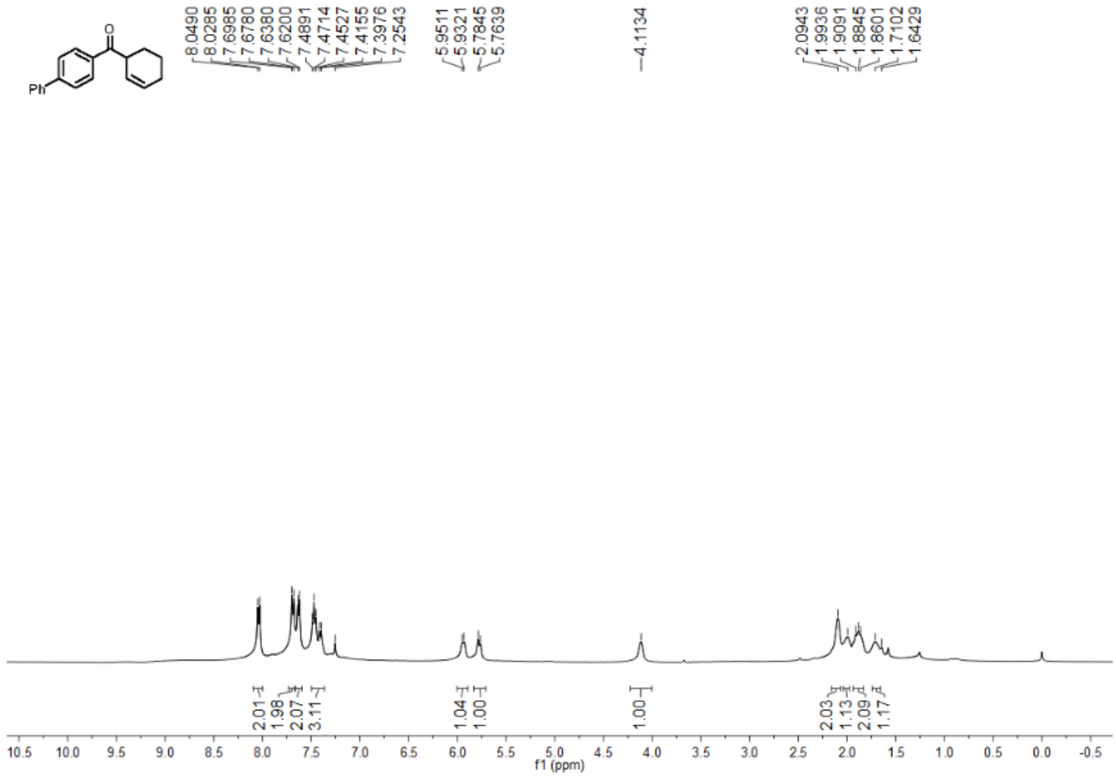

Supplementary Figure 19 ^{1}H NMR (400 MHz) spectrum of compound **6** in $CDCl_3$

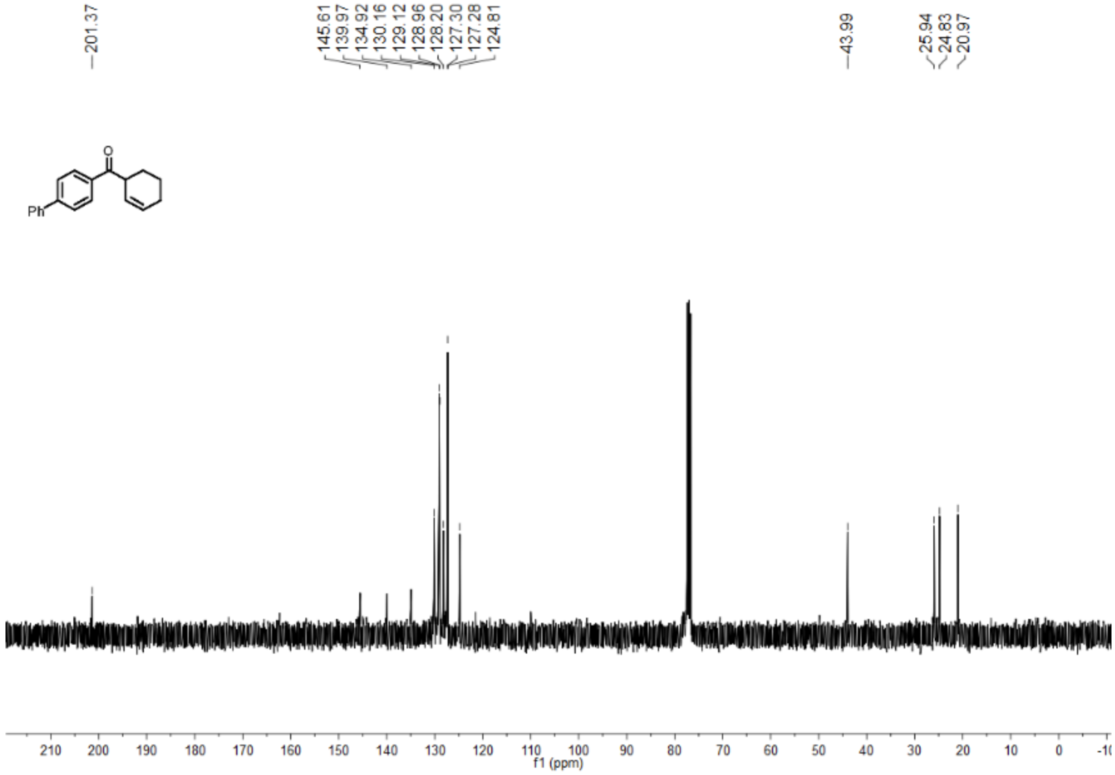

Supplementary Figure 20 ^{13}C NMR (100 MHz) spectrum of compound **6** in $CDCl_3$

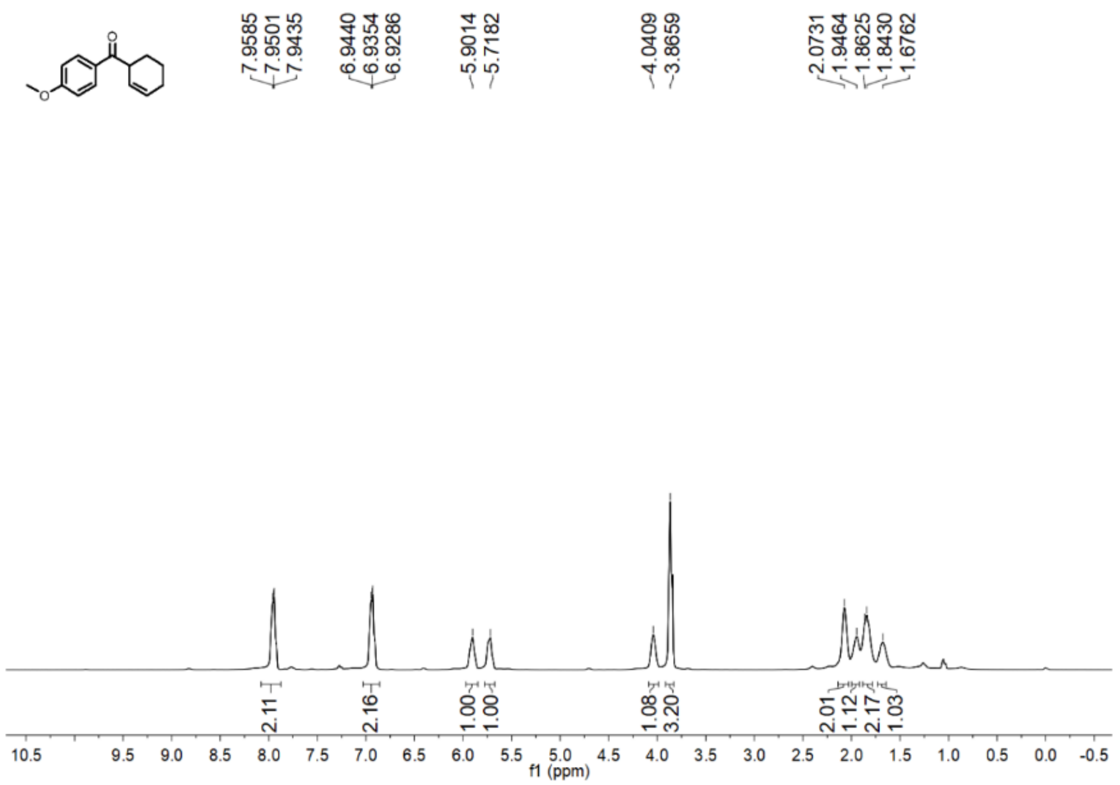

Supplementary Figure 21 ^{1}H NMR (400 MHz) spectrum of compound **7** in $CDCl_3$

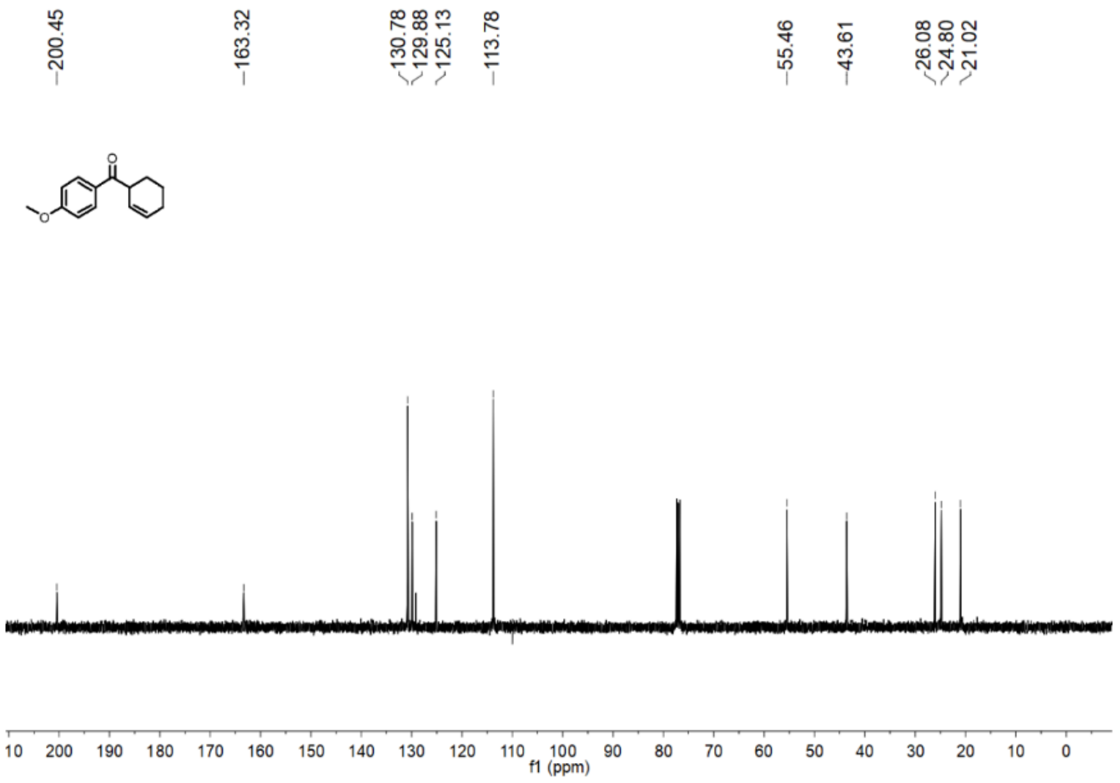

Supplementary Figure 22 ^{13}C NMR (100 MHz) spectrum of compound **7** in $CDCl_3$

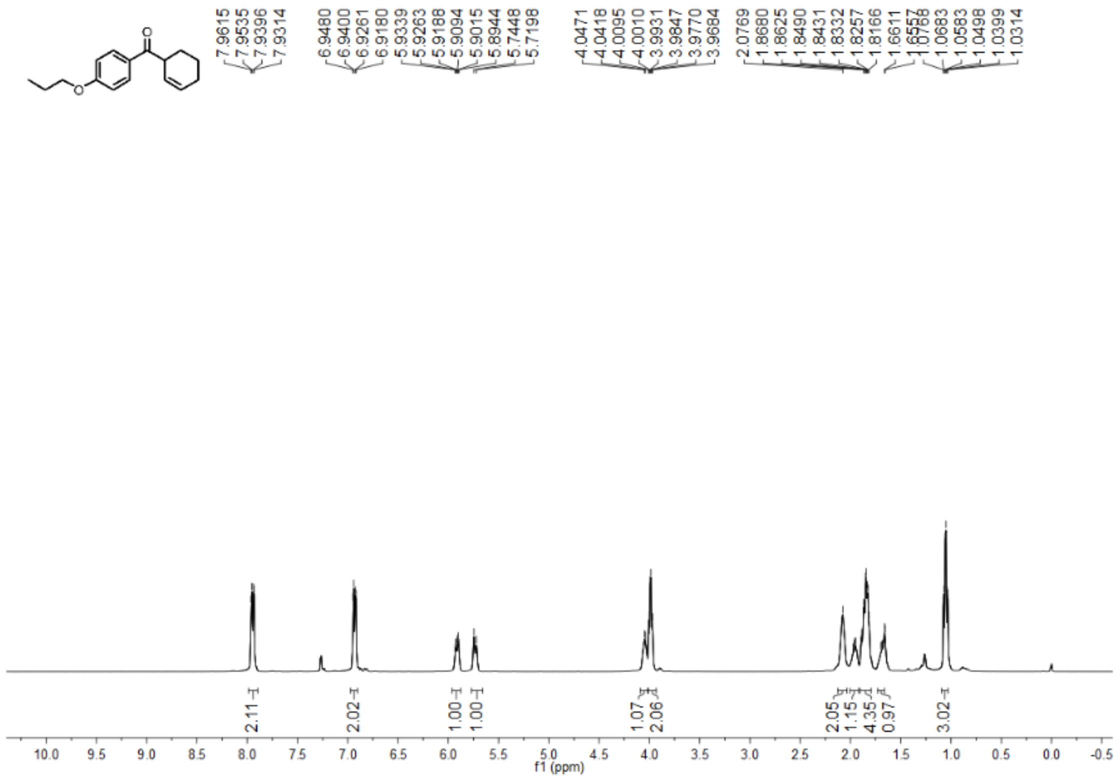

Supplementary Figure 23 ^{1}H NMR (400 MHz) spectrum of compound **8** in $CDCl_3$

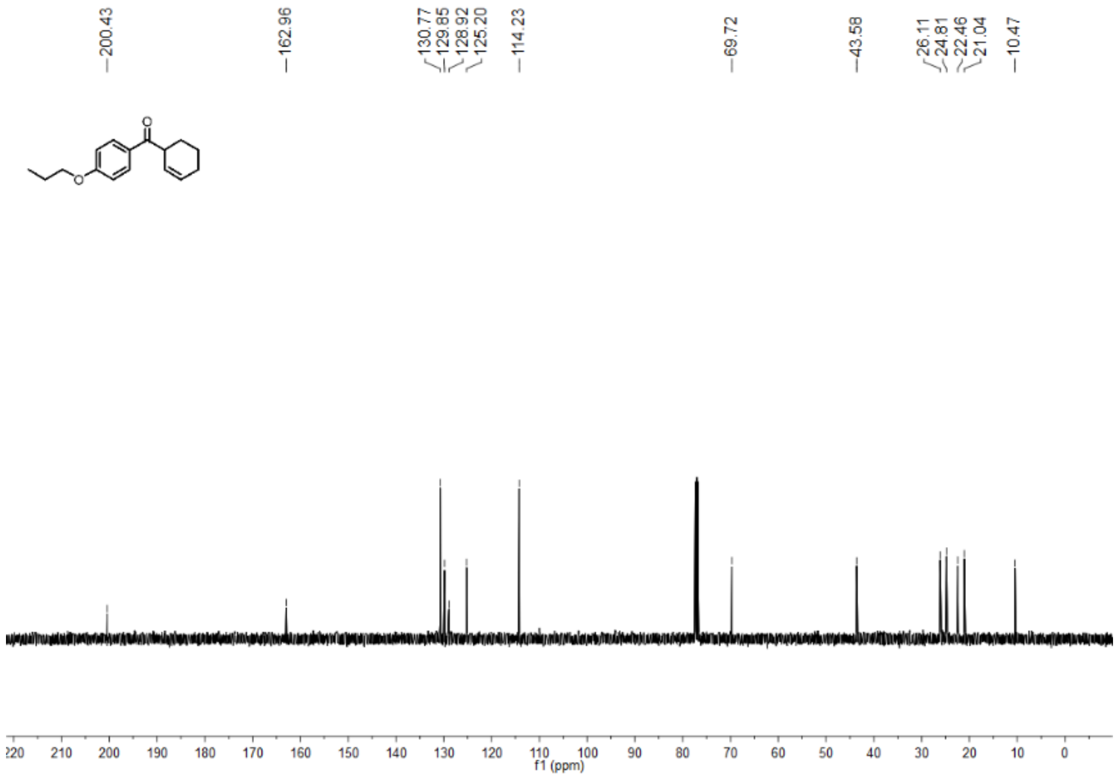

Supplementary Figure 24 ^{13}C NMR (100 MHz) spectrum of compound **8** in $CDCl_3$

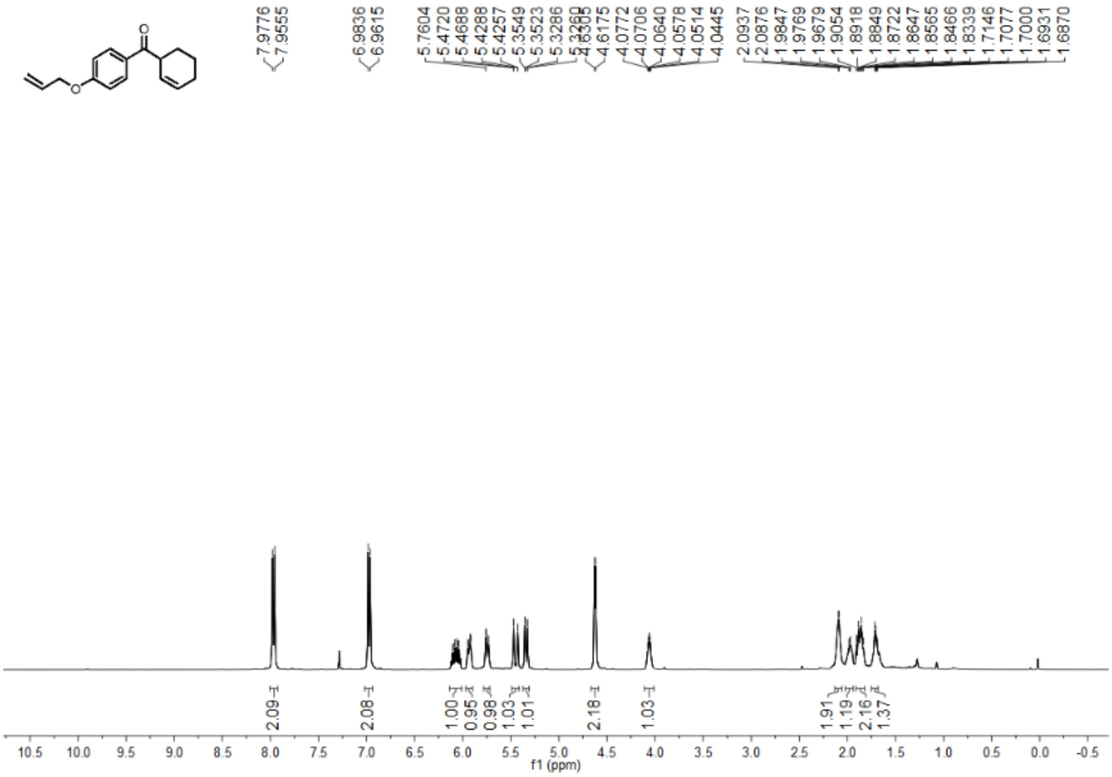

Supplementary Figure 25 ^{1}H NMR (400 MHz) spectrum of compound **9** in $CDCl_3$

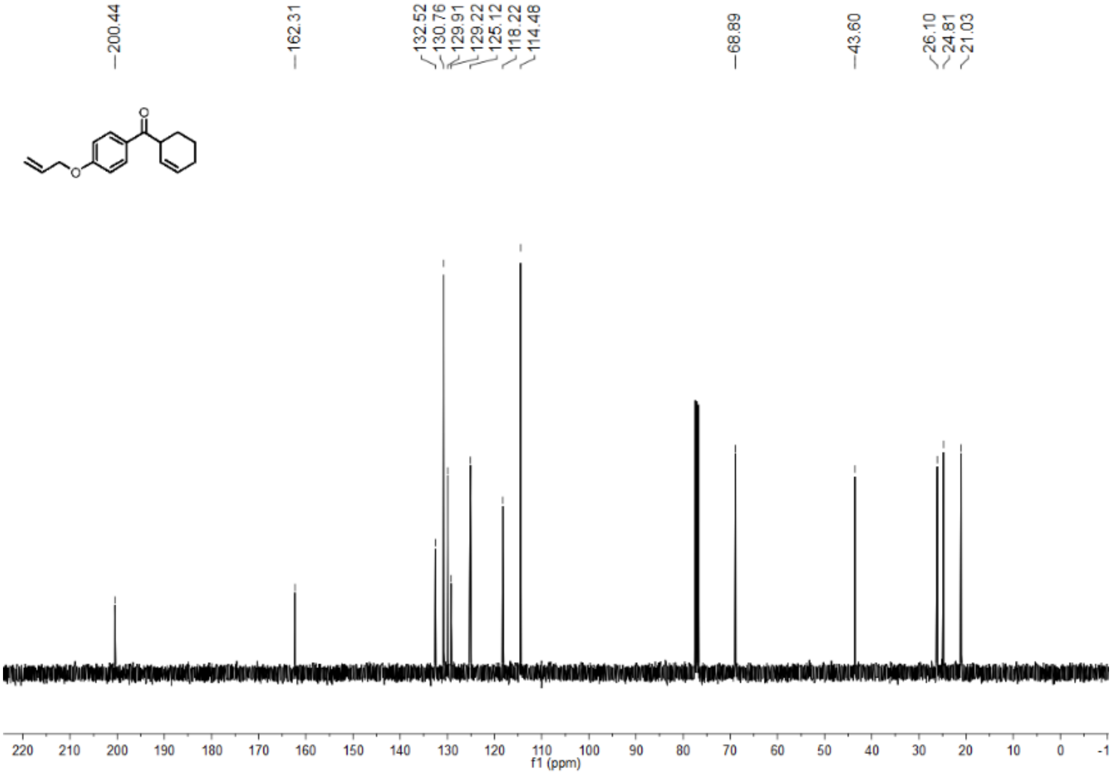

Supplementary Figure 26 ^{13}C NMR (100 MHz) spectrum of compound **9** in $CDCl_3$

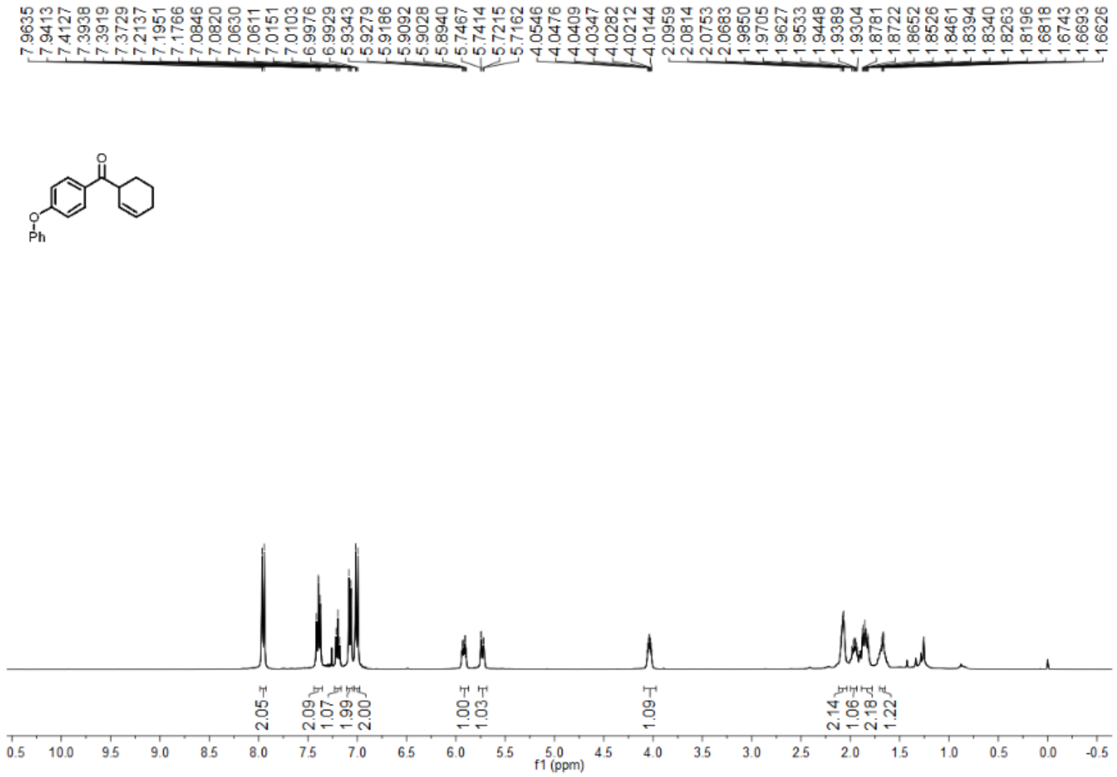

Supplementary Figure 27 ^{1}H NMR (400 MHz) spectrum of compound **10** in $CDCl_3$

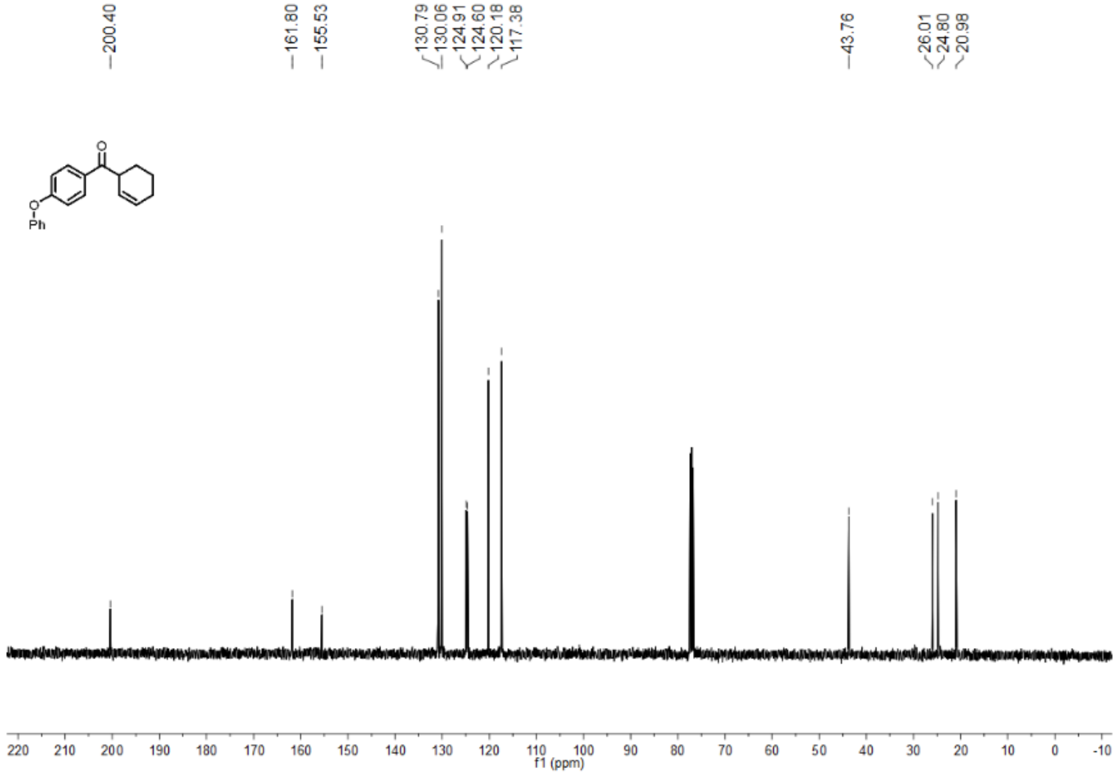

Supplementary Figure 28 ^{13}C NMR (100 MHz) spectrum of compound **10** in $CDCl_3$

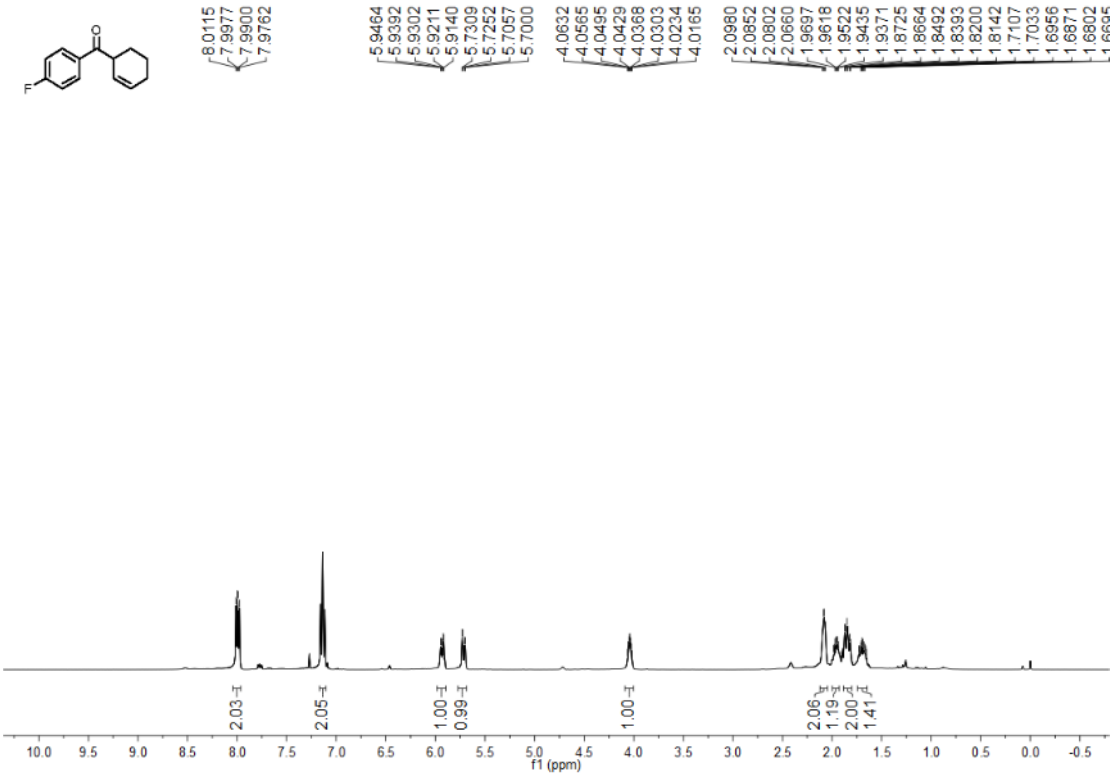

Supplementary Figure 29 ^{1}H NMR (400 MHz) spectrum of compound **11** in $CDCl_3$

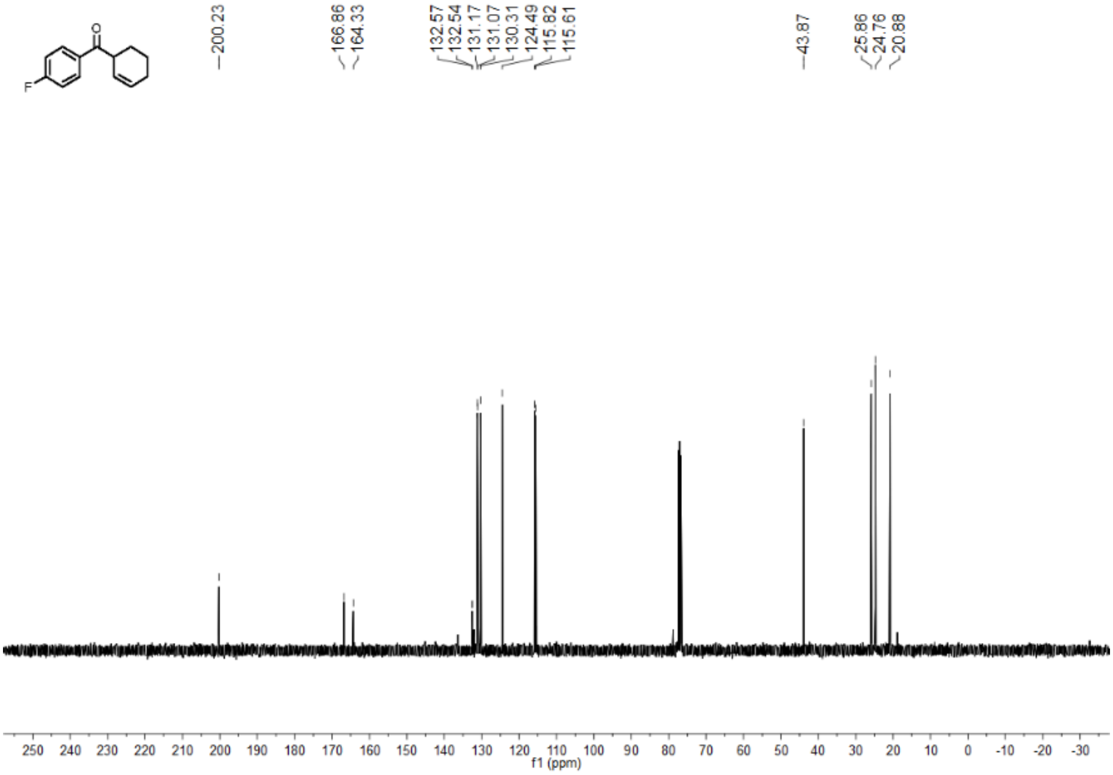

Supplementary Figure 30 ^{13}C NMR (100 MHz) spectrum of compound **11** in $CDCl_3$

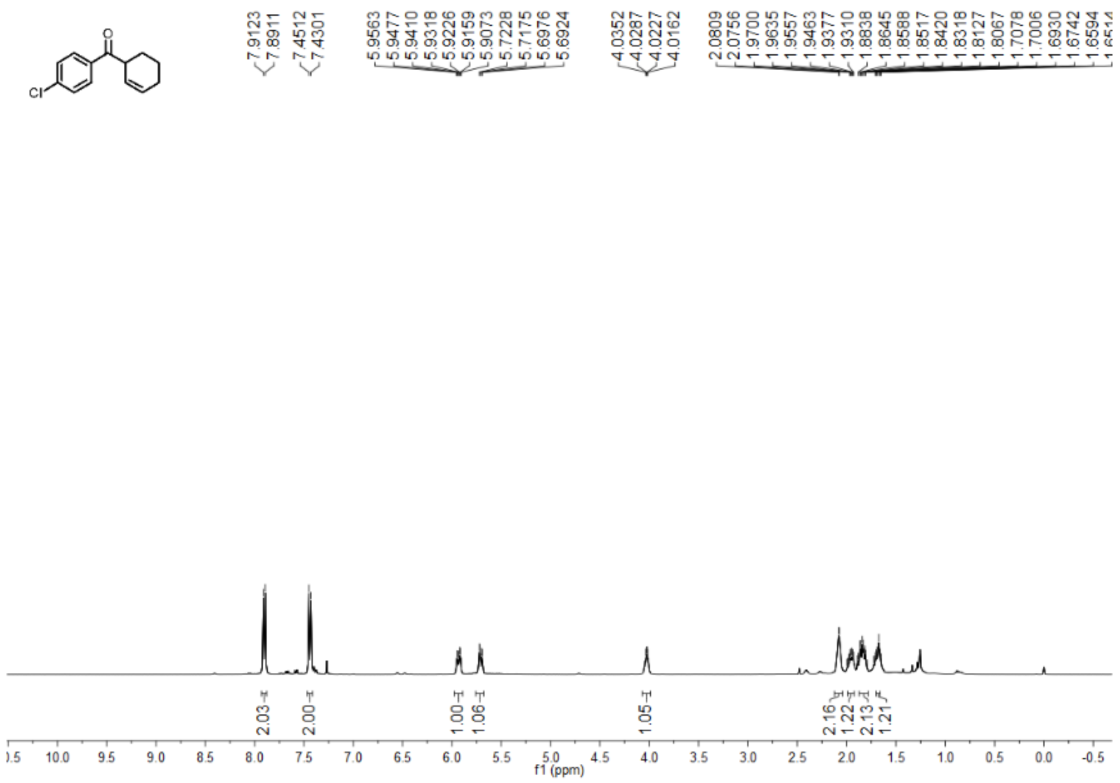

Supplementary Figure 31 ^{1}H NMR (400 MHz) spectrum of compound **12** in $CDCl_3$

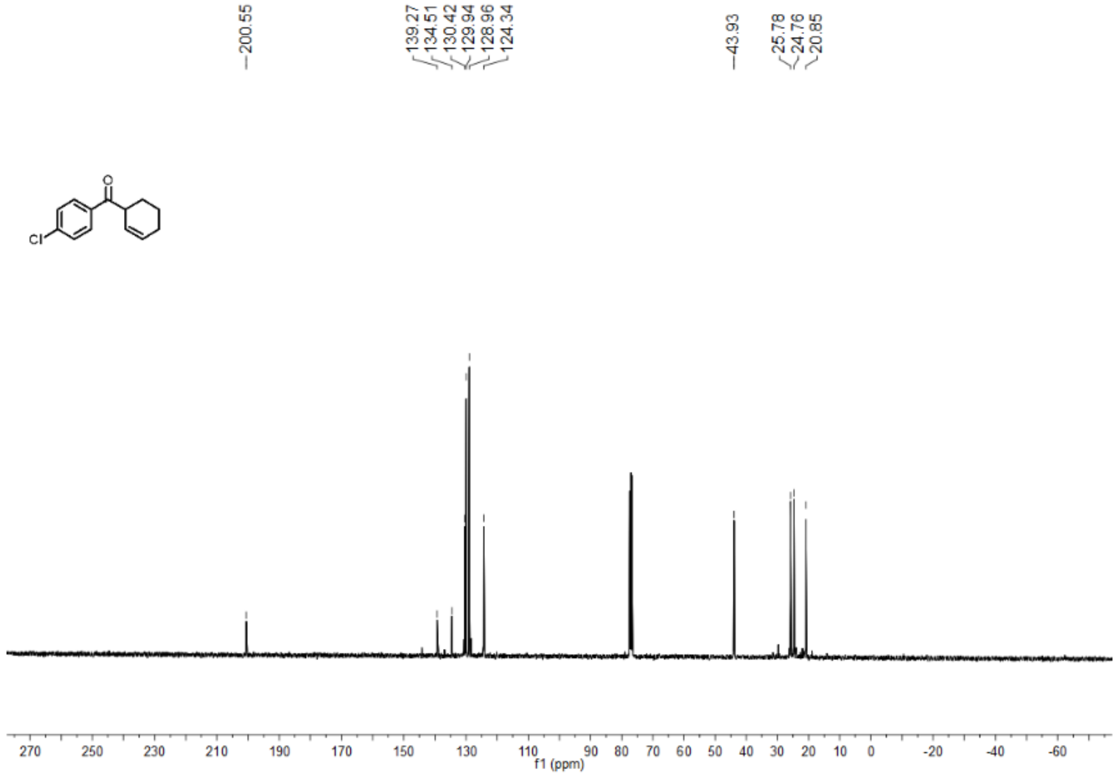

Supplementary Figure 32 ^{13}C NMR (100 MHz) spectrum of compound **12** in $CDCl_3$

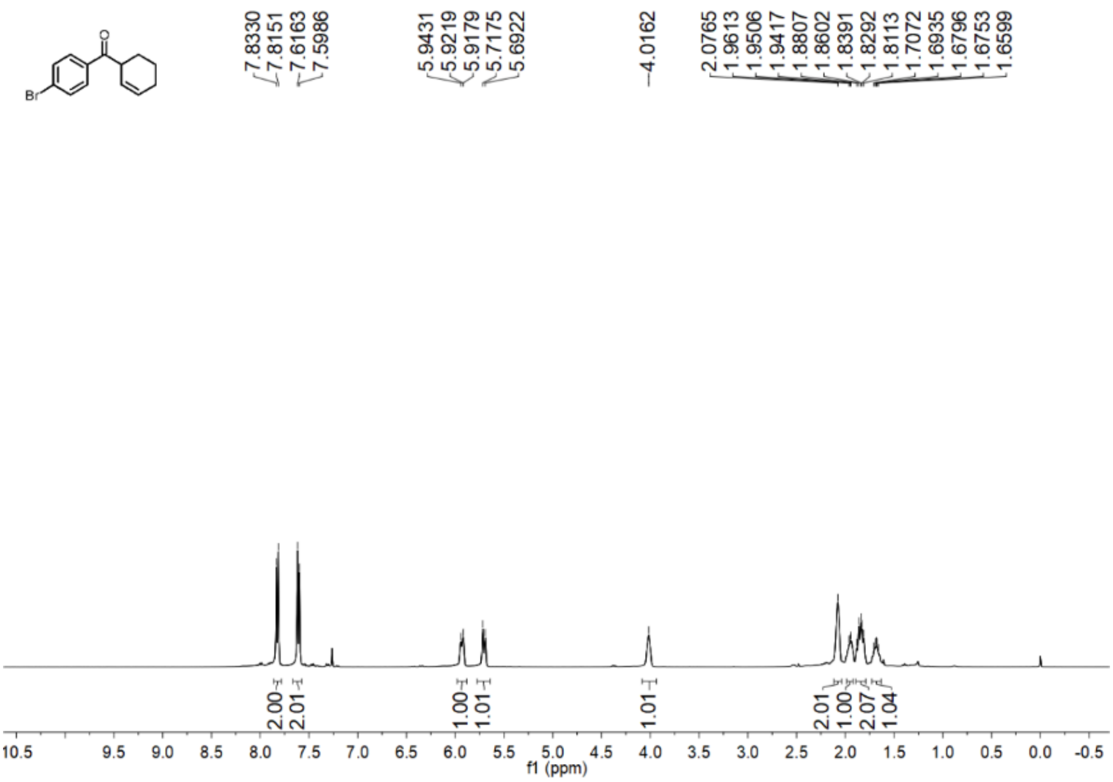

Supplementary Figure 33 ^{1}H NMR (400 MHz) spectrum of compound **13** in $CDCl_3$

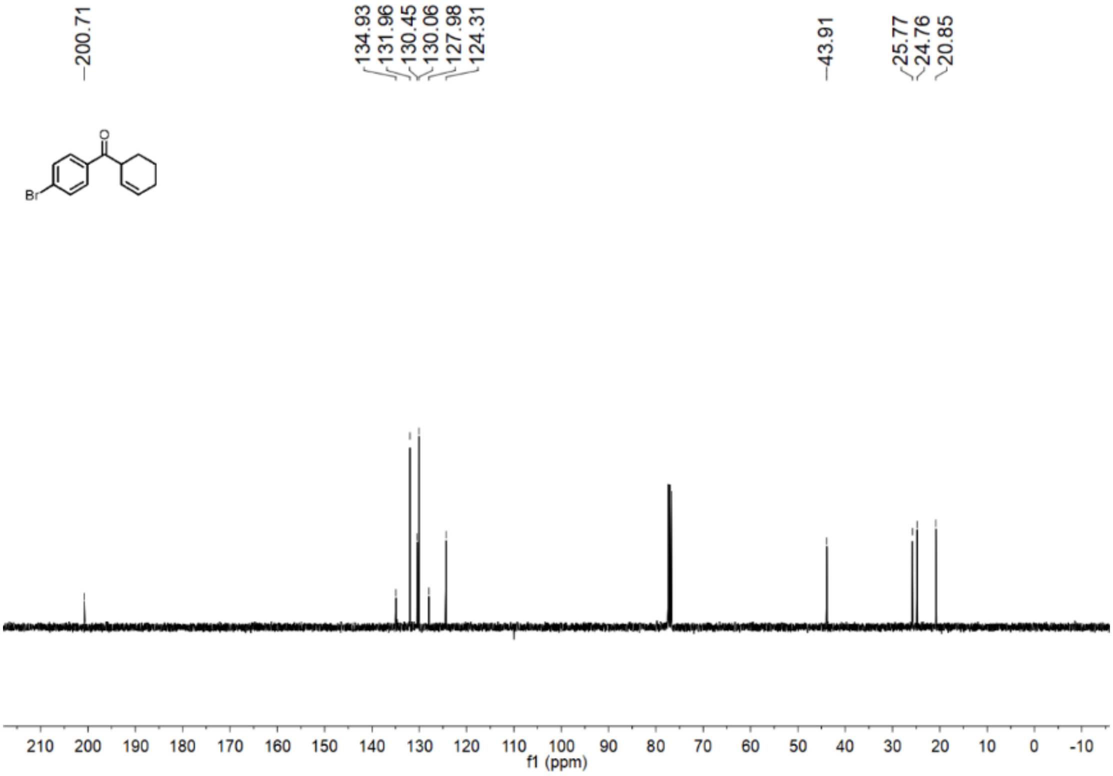

Supplementary Figure 34 ^{13}C NMR (100 MHz) spectrum of compound **13** in $CDCl_3$

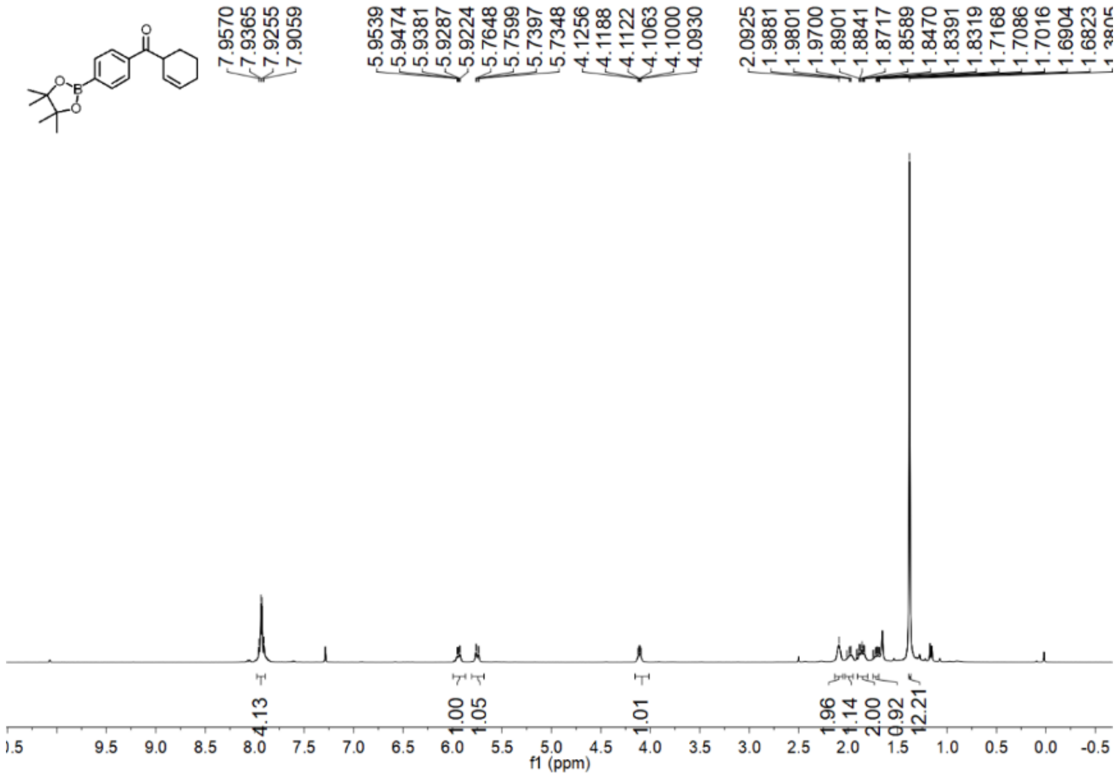

Supplementary Figure 35 ^{1}H NMR (400 MHz) spectrum of compound **14** in $CDCl_3$

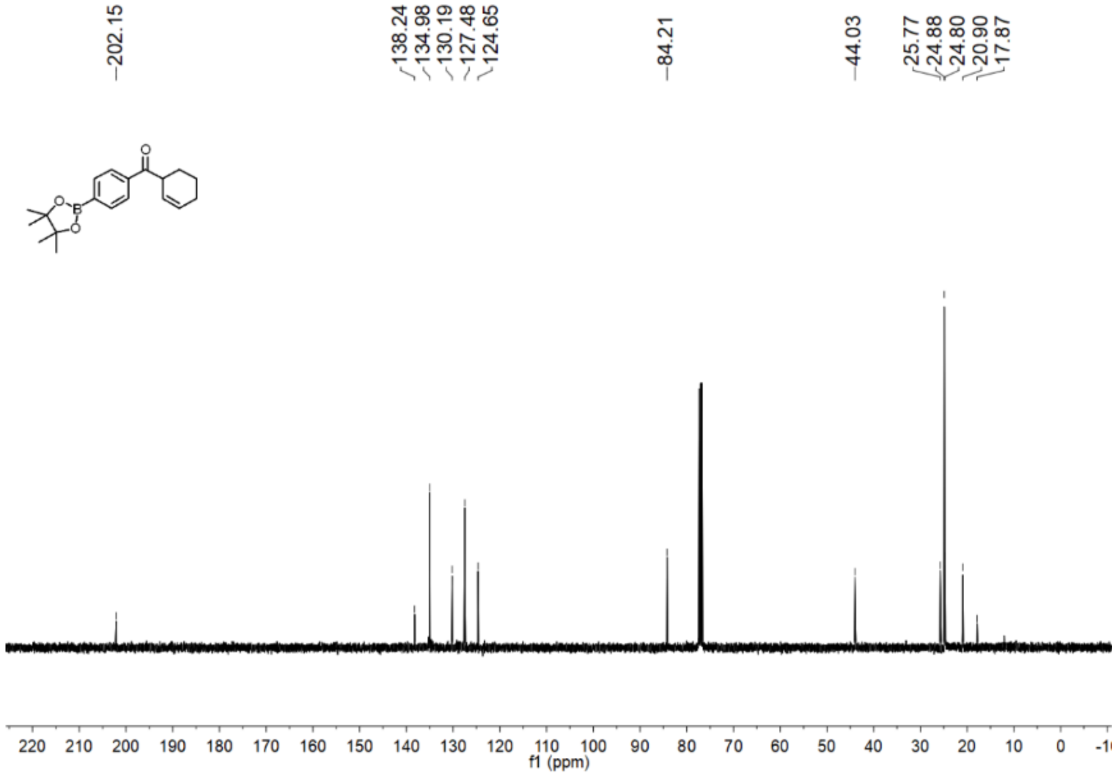

Supplementary Figure 36 ^{13}C NMR (100 MHz) spectrum of compound **14** in $CDCl_3$

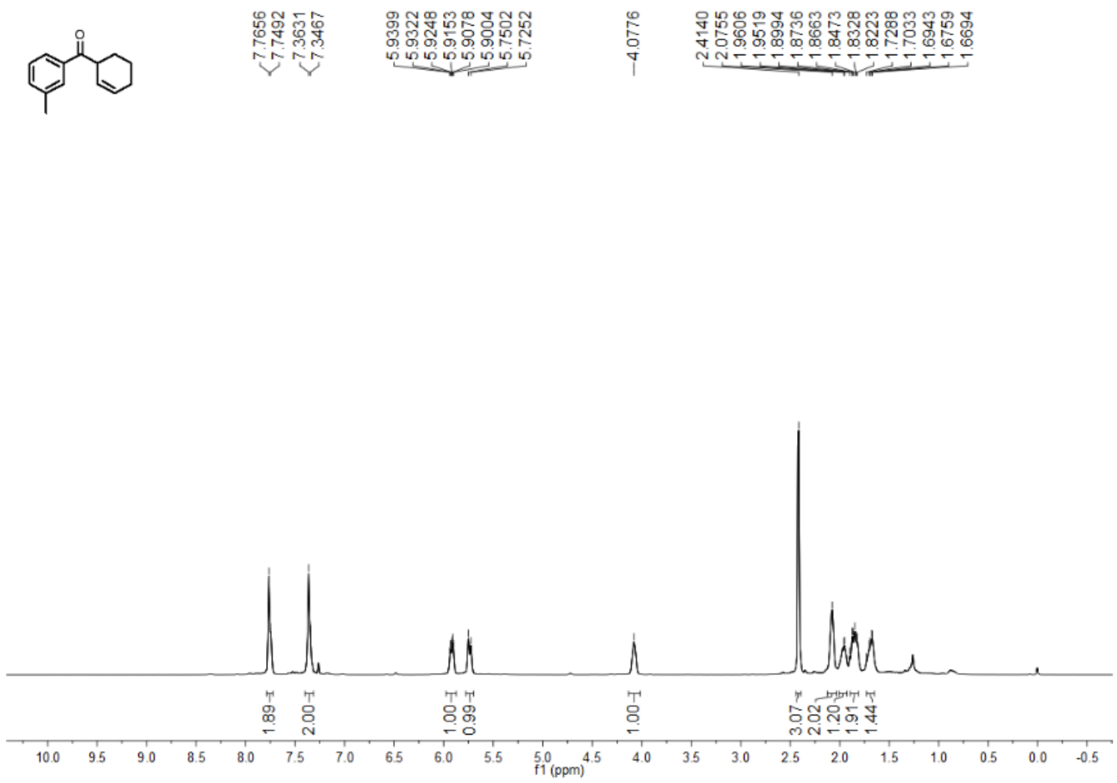

Supplementary Figure 37 ^{1}H NMR (400 MHz) spectrum of compound **15** in $CDCl_3$

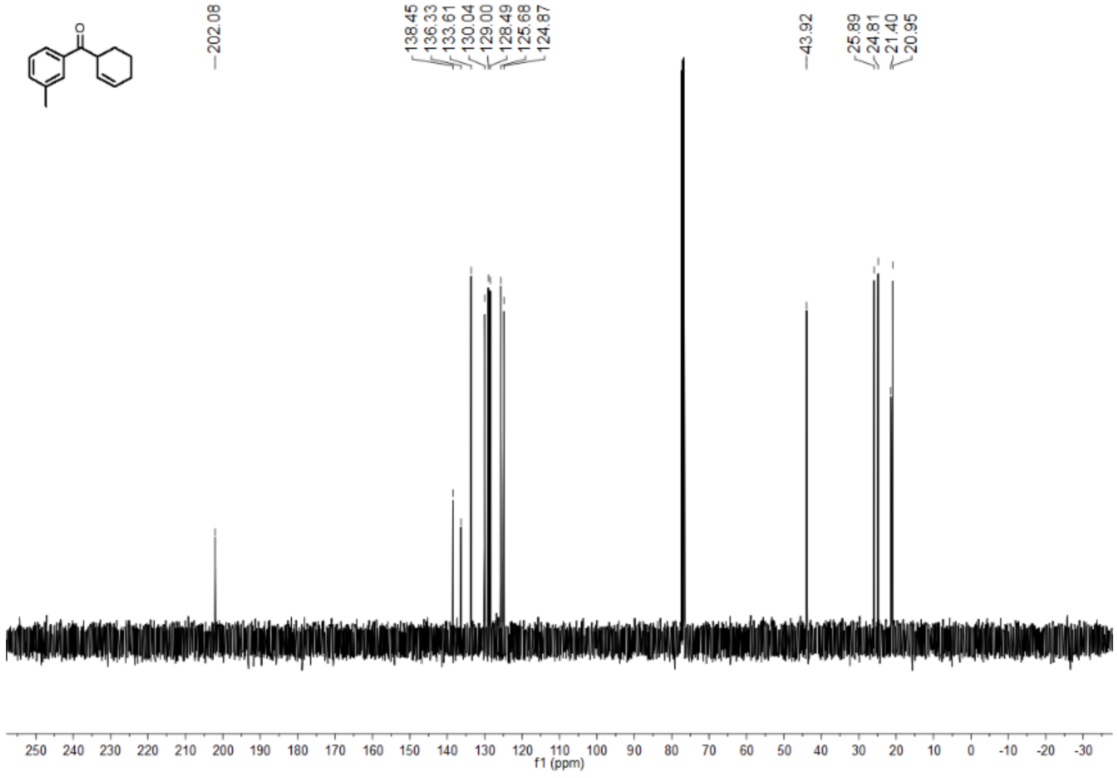

Supplementary Figure 38 ^{13}C NMR (100 MHz) spectrum of compound **15** in $CDCl_3$

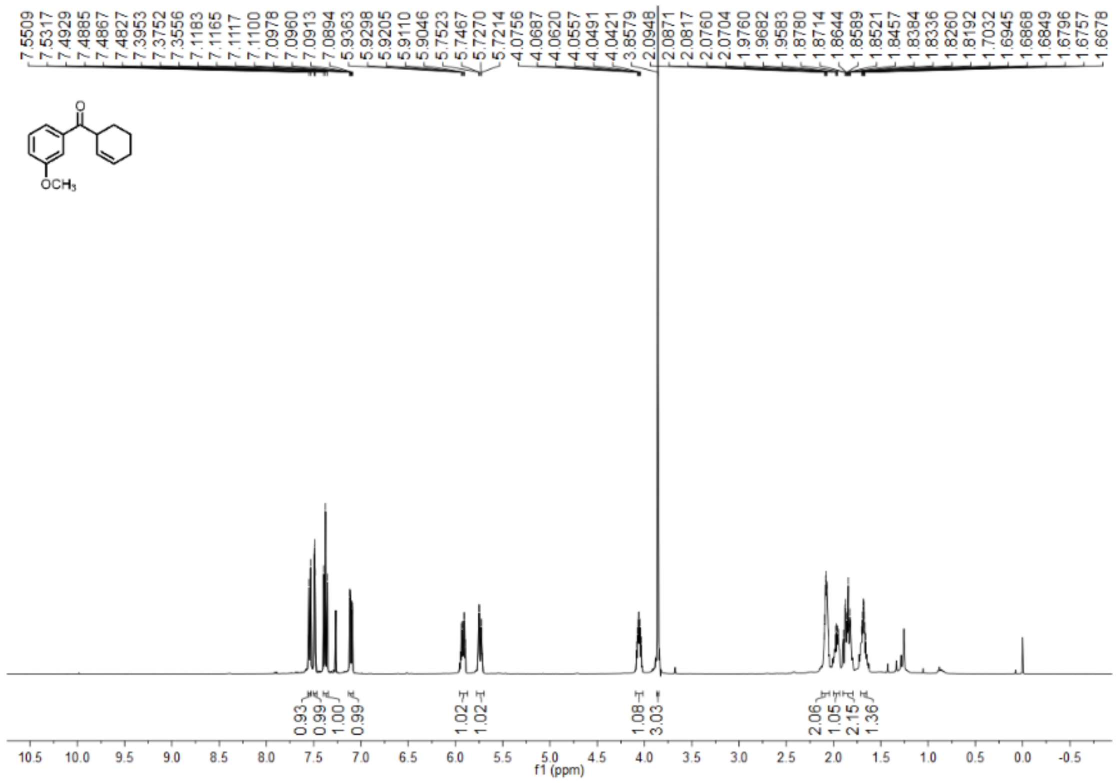

Supplementary Figure 39 ^{1}H NMR (400 MHz) spectrum of compound **16** in $CDCl_3$

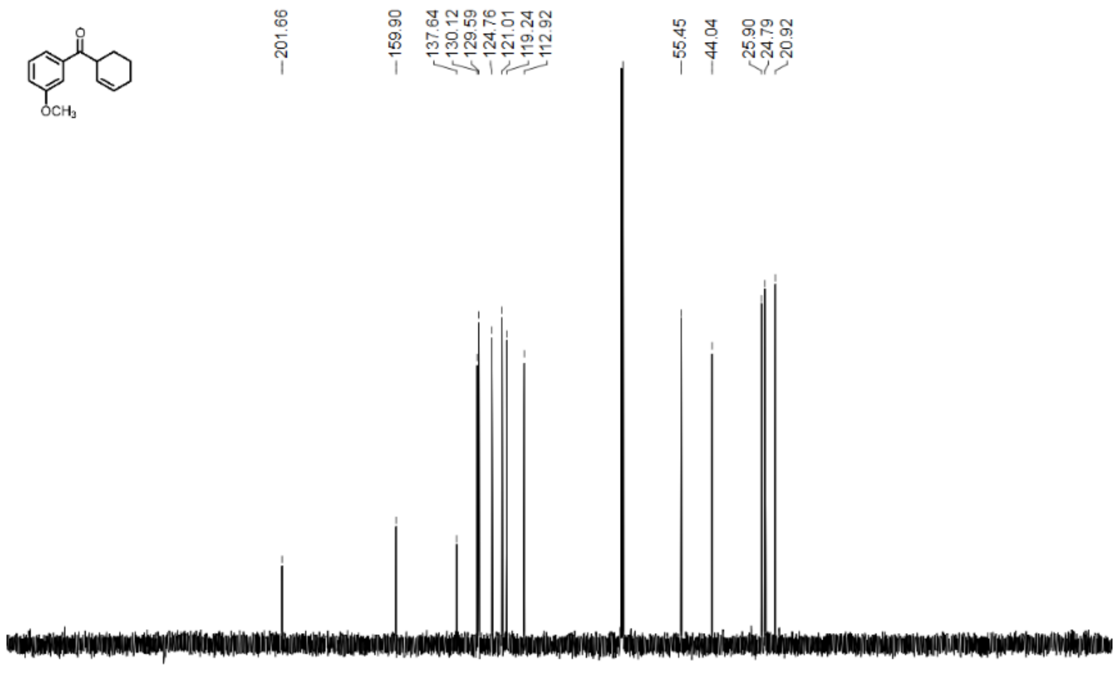

Supplementary Figure 40 ^{13}C NMR (100 MHz) spectrum of compound **16** in $CDCl_3$

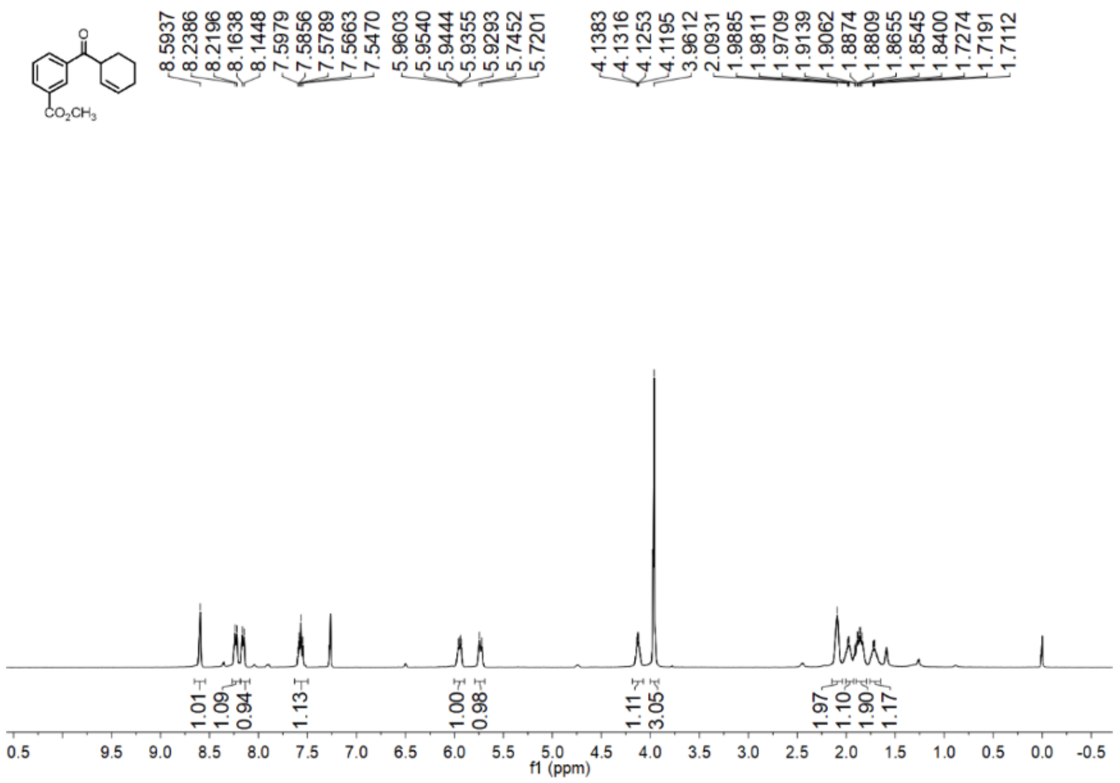

Supplementary Figure 41 ^{1}H NMR (400 MHz) spectrum of compound **17** in $CDCl_3$

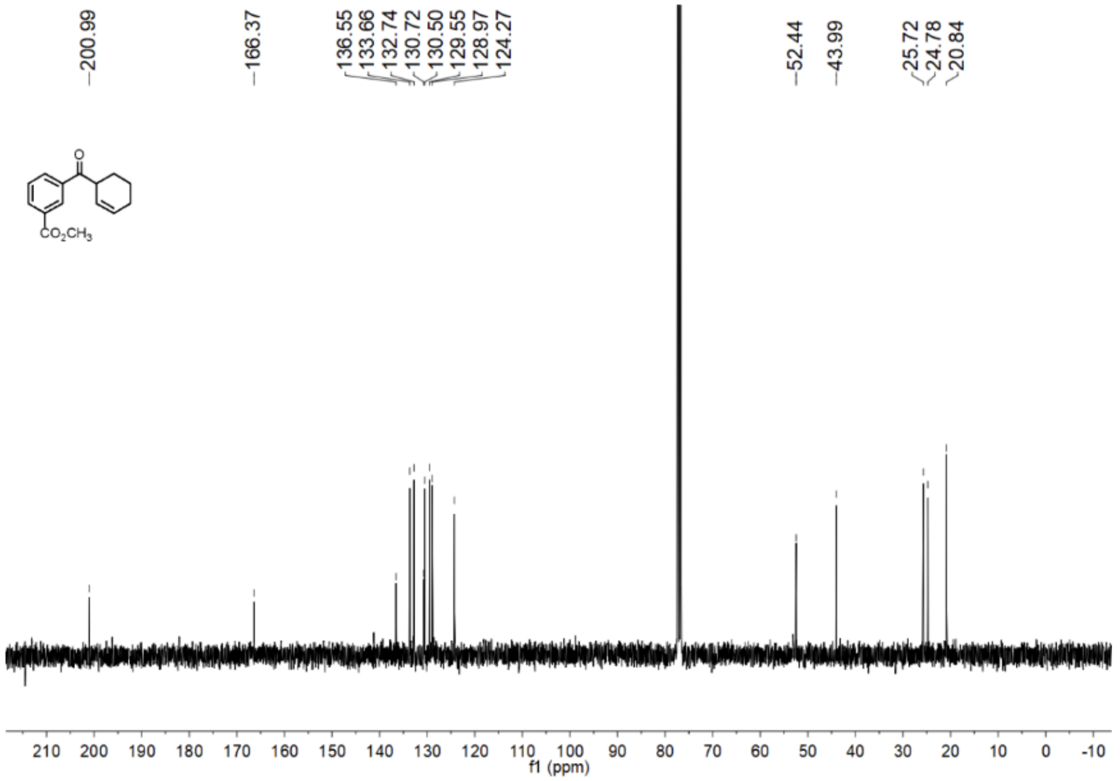

Supplementary Figure 42 ^{13}C NMR (100 MHz) spectrum of compound **17** in $CDCl_3$

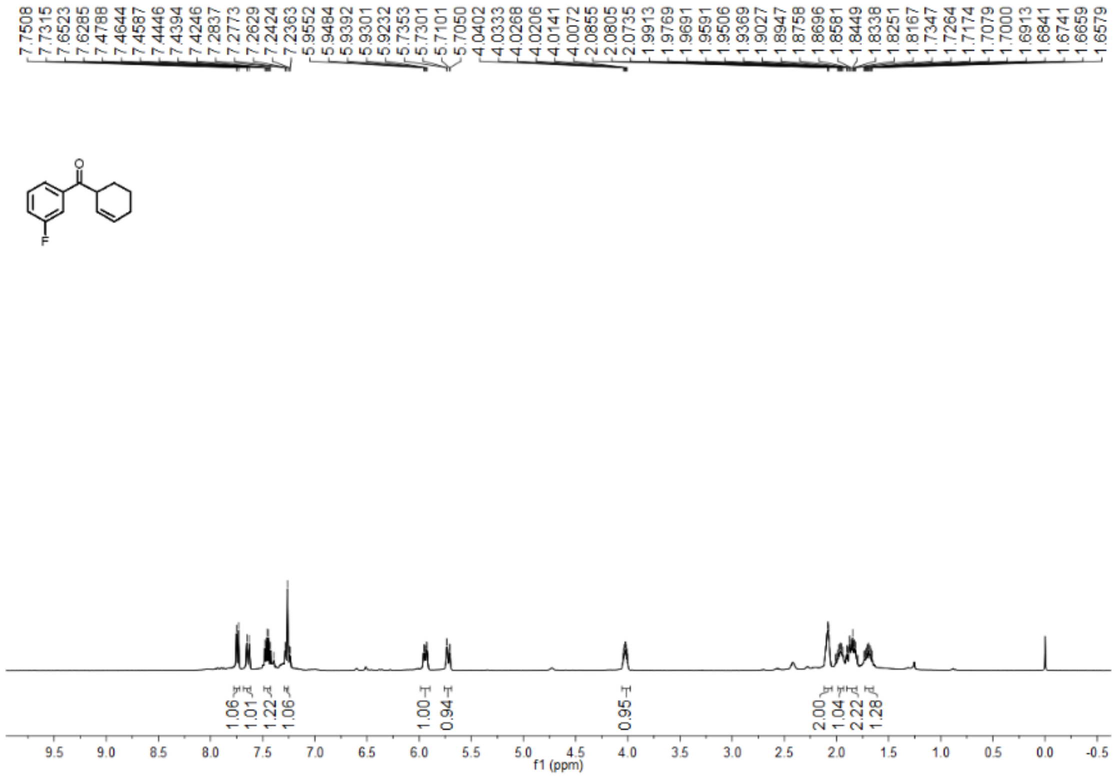

Supplementary Figure 43 ^{1}H NMR (400 MHz) spectrum of compound **18** in $CDCl_3$

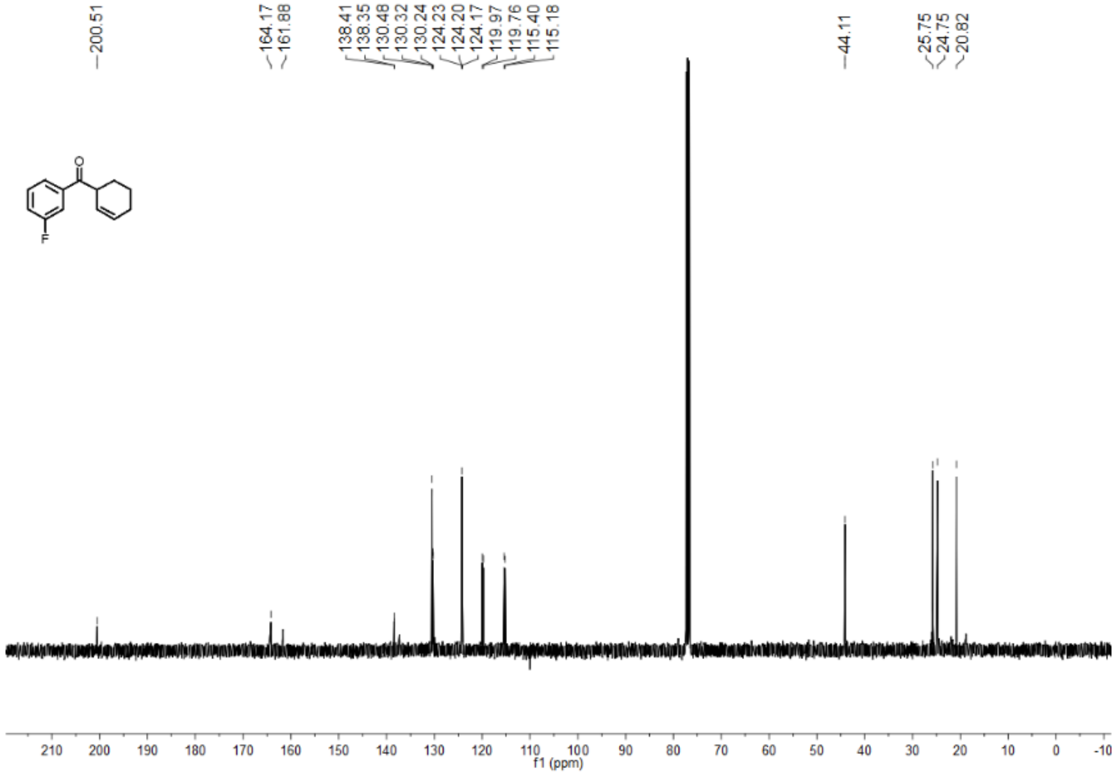

Supplementary Figure 44 ^{13}C NMR (100 MHz) spectrum of compound **18** in $CDCl_3$

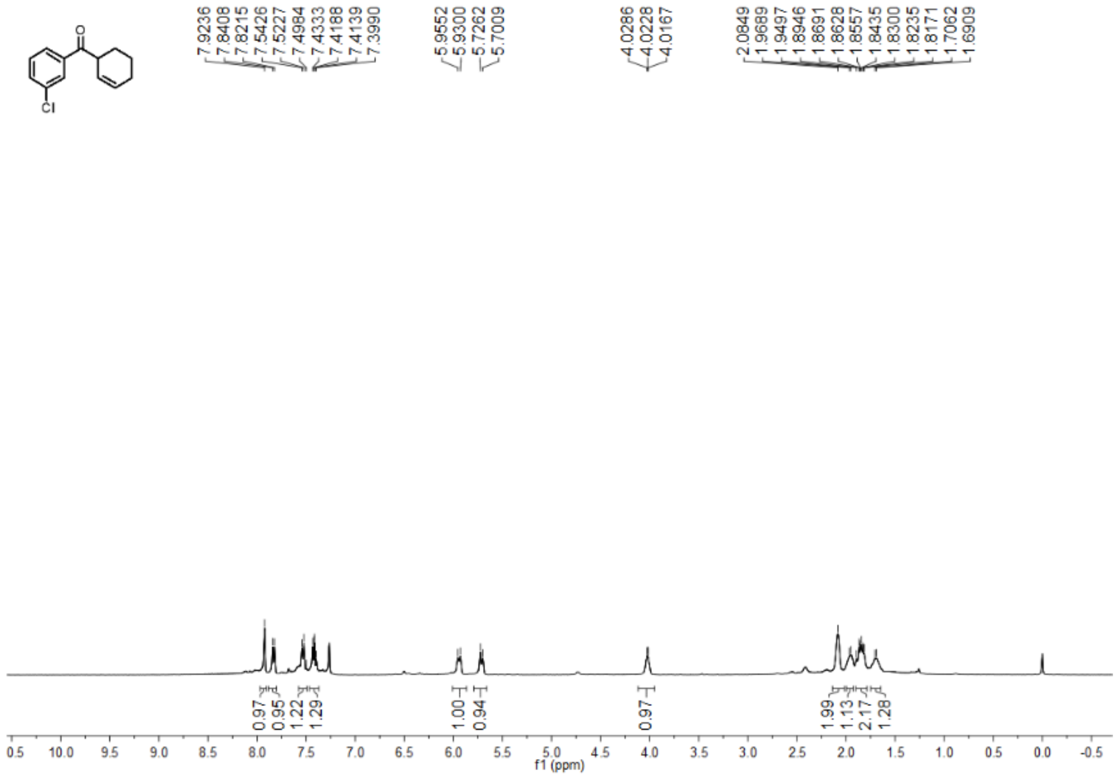

Supplementary Figure 45 ^{1}H NMR (400 MHz) spectrum of compound **19** in $CDCl_3$

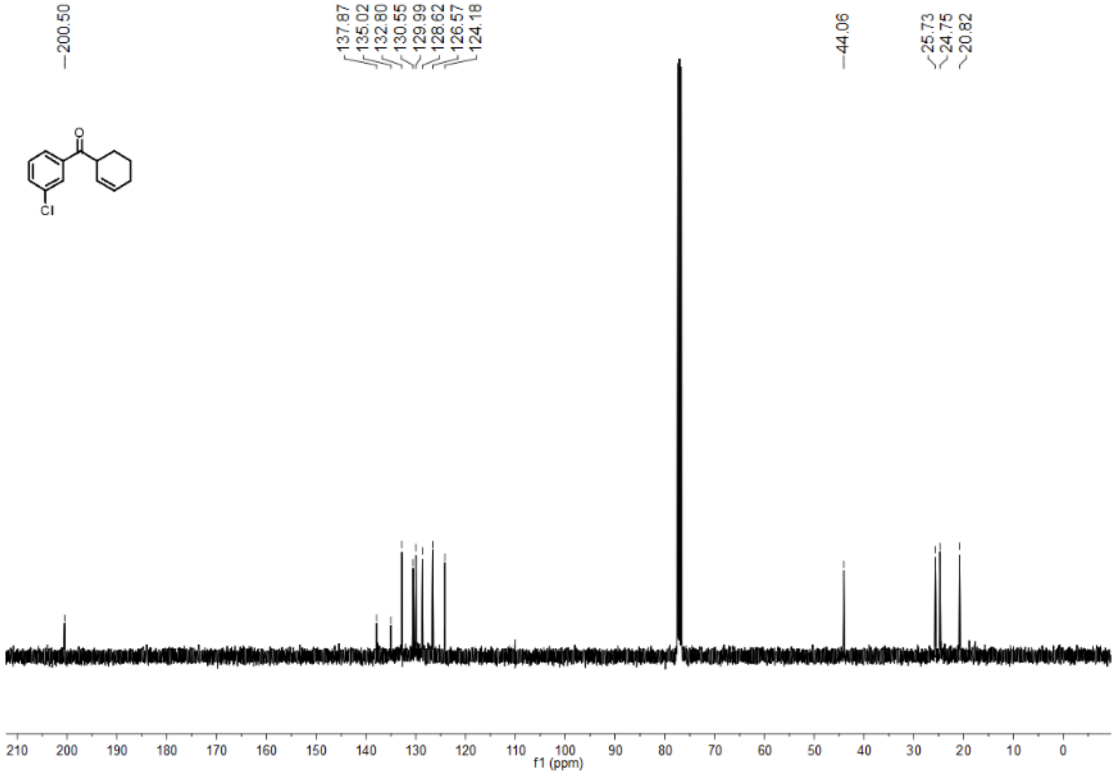

Supplementary Figure 46 ^{13}C NMR (100 MHz) spectrum of compound **19** in $CDCl_3$

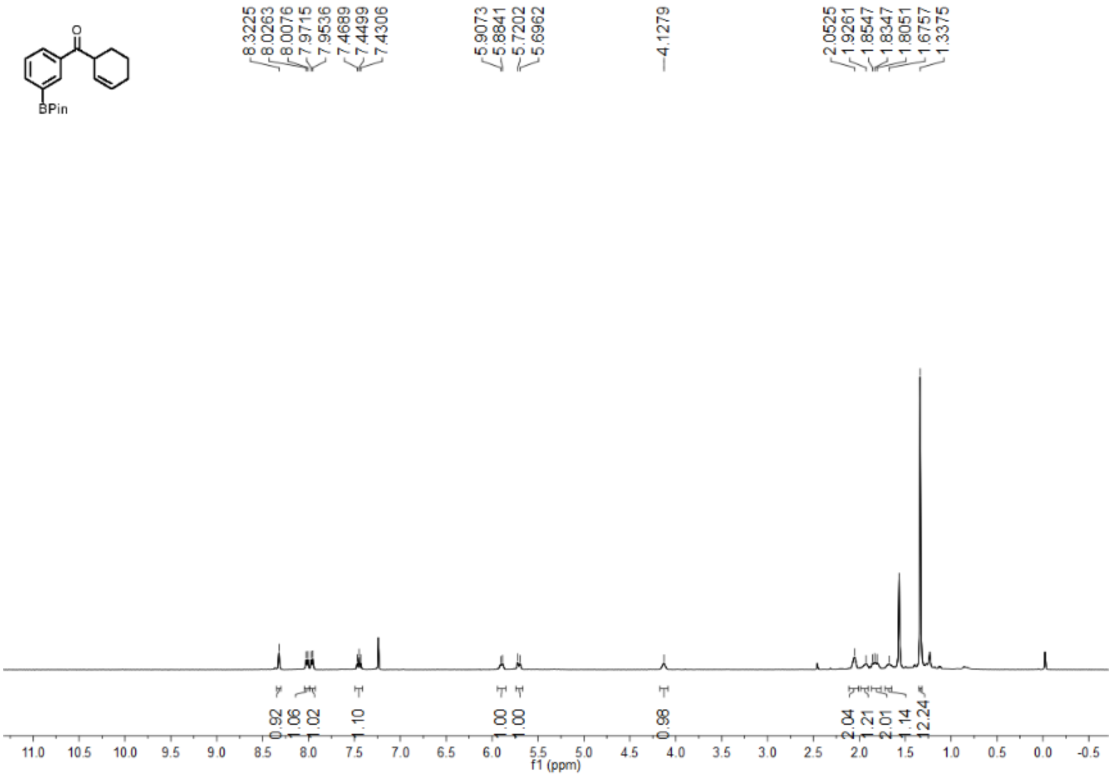

Supplementary Figure 47 ^{1}H NMR (400 MHz) spectrum of compound **20** in $CDCl_3$

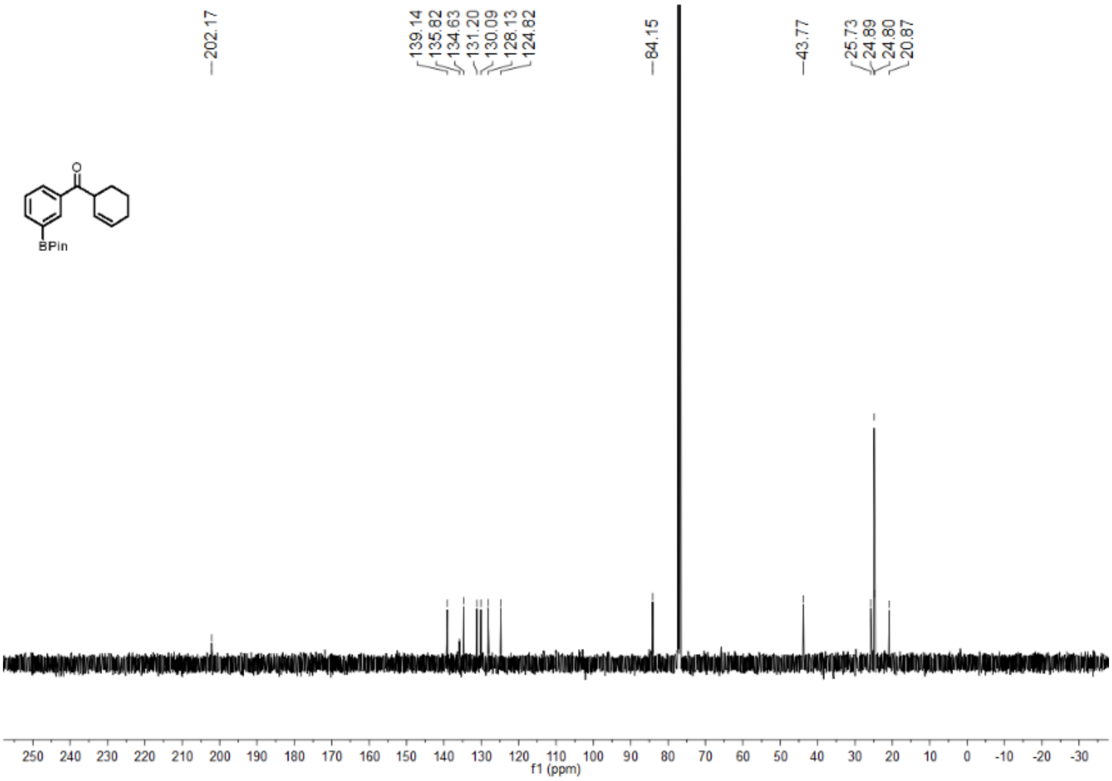

Supplementary Figure 48 ^{13}C NMR (100 MHz) spectrum of compound **20** in $CDCl_3$

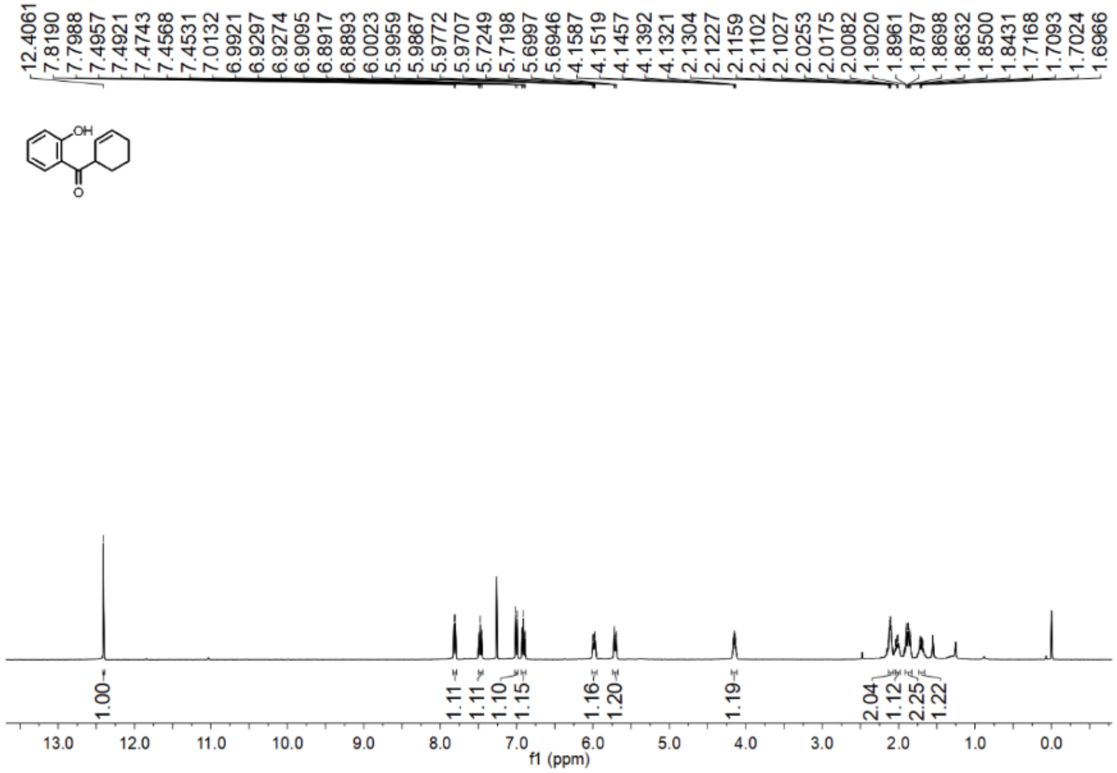

Supplementary Figure 49 ^{1}H NMR (400 MHz) spectrum of compound **21** in $CDCl_3$

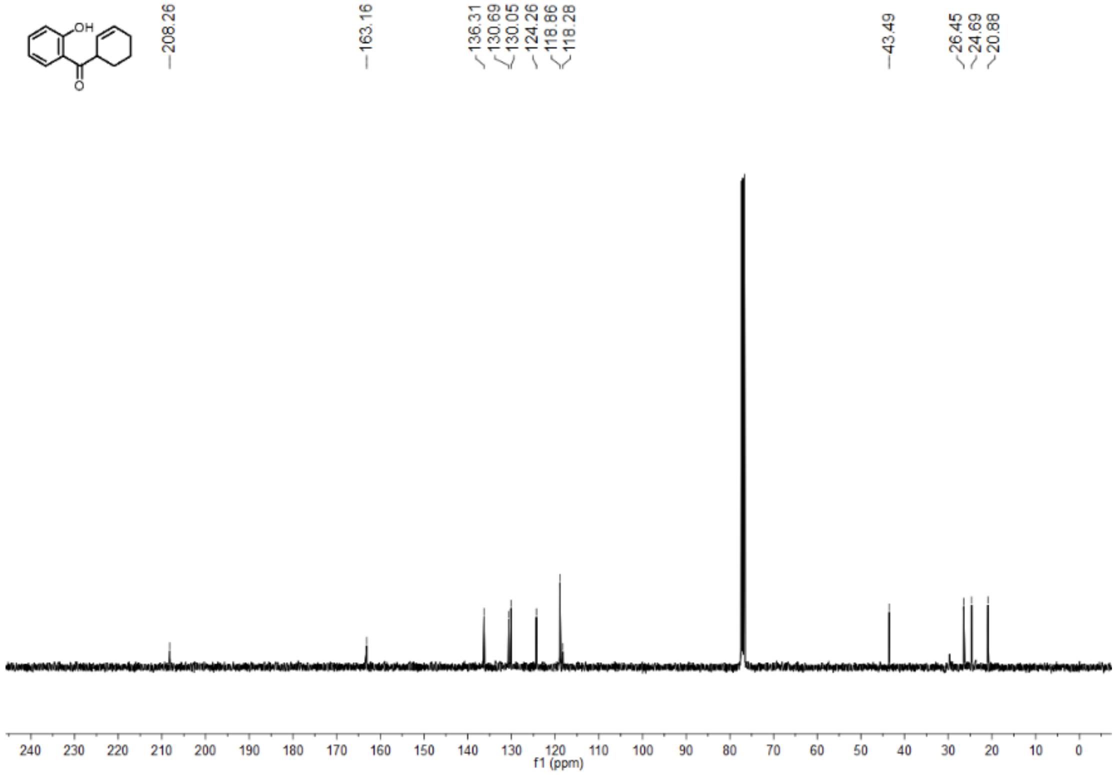

Supplementary Figure 50 ^{13}C NMR (100 MHz) spectrum of compound **21** in $CDCl_3$

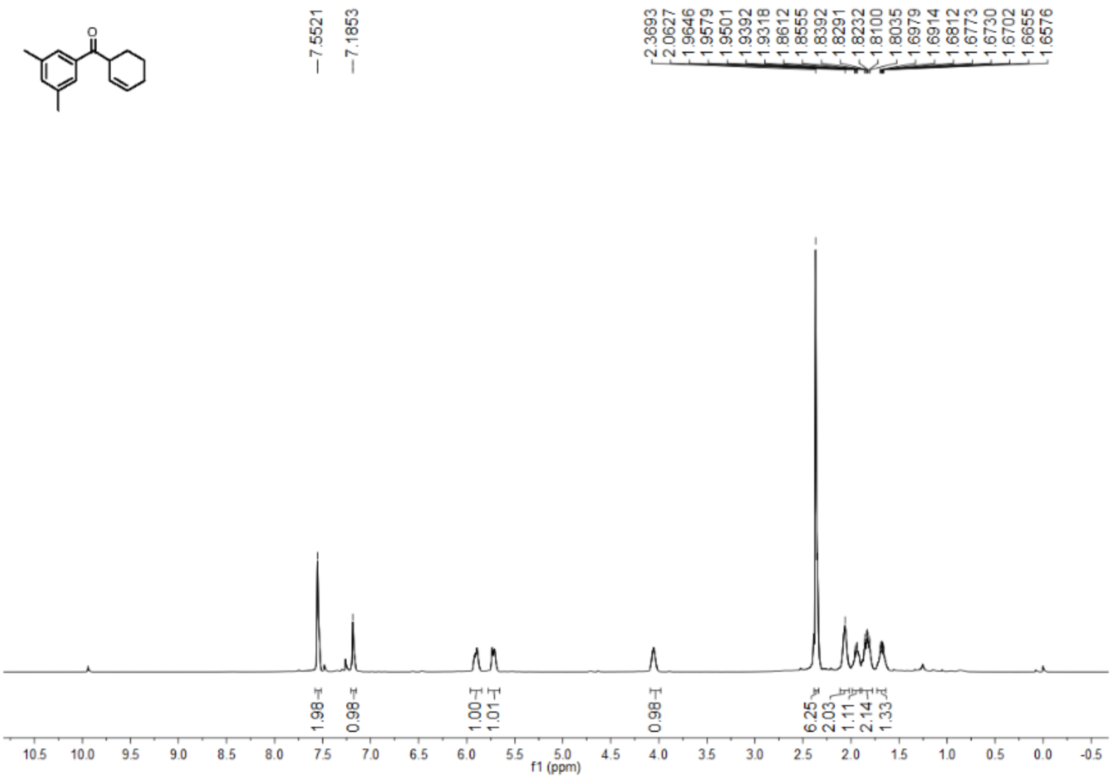

Supplementary Figure 51 ^{1}H NMR (400 MHz) spectrum of compound **22** in $CDCl_3$

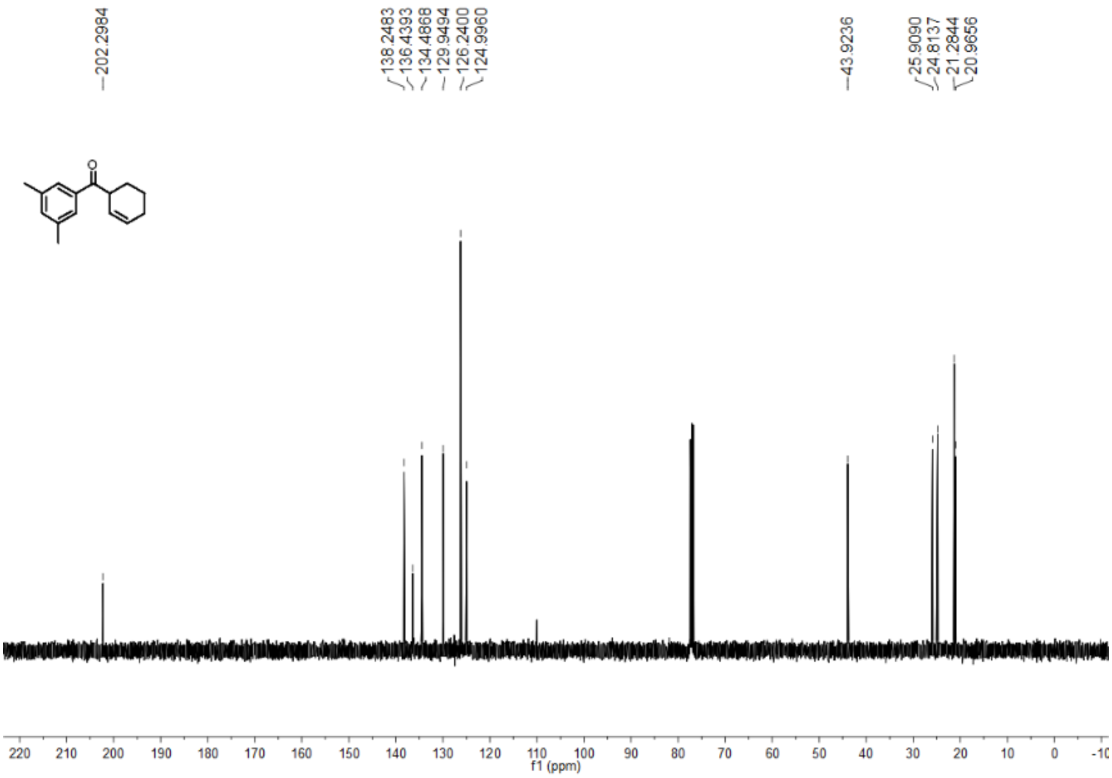

Supplementary Figure 52 ^{13}C NMR (100 MHz) spectrum of compound **22** in $CDCl_3$

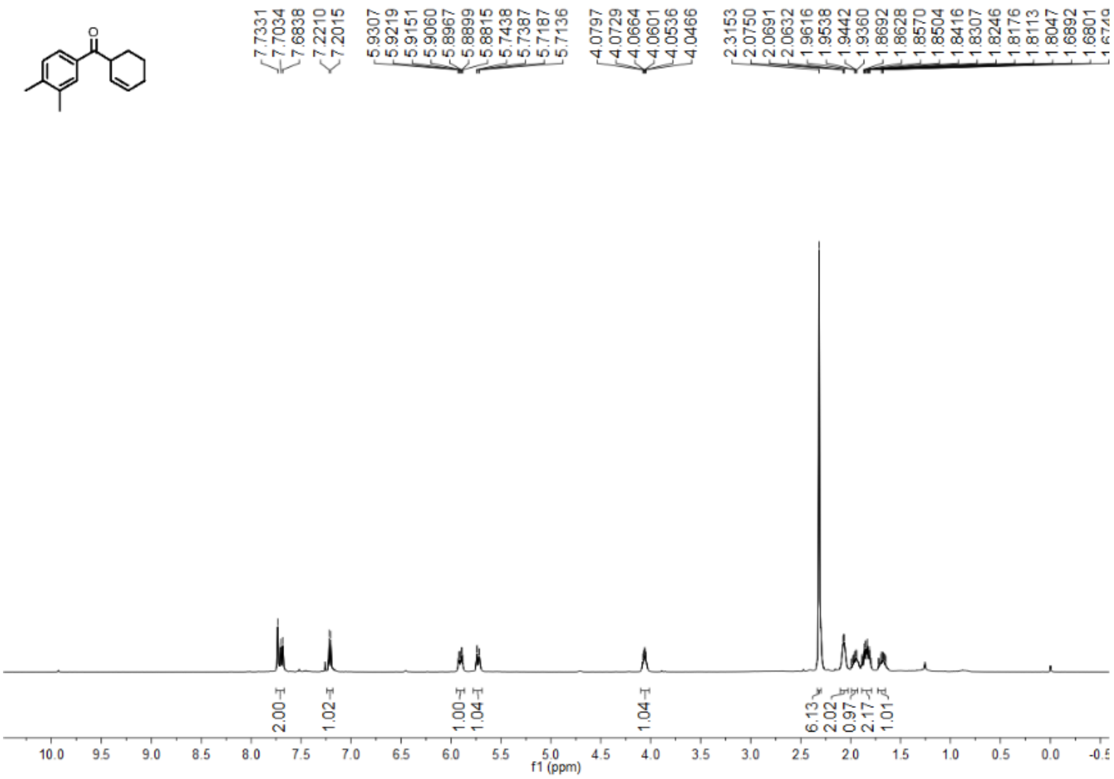

Supplementary Figure 53 ^{1}H NMR (400 MHz) spectrum of compound **23** in $CDCl_3$

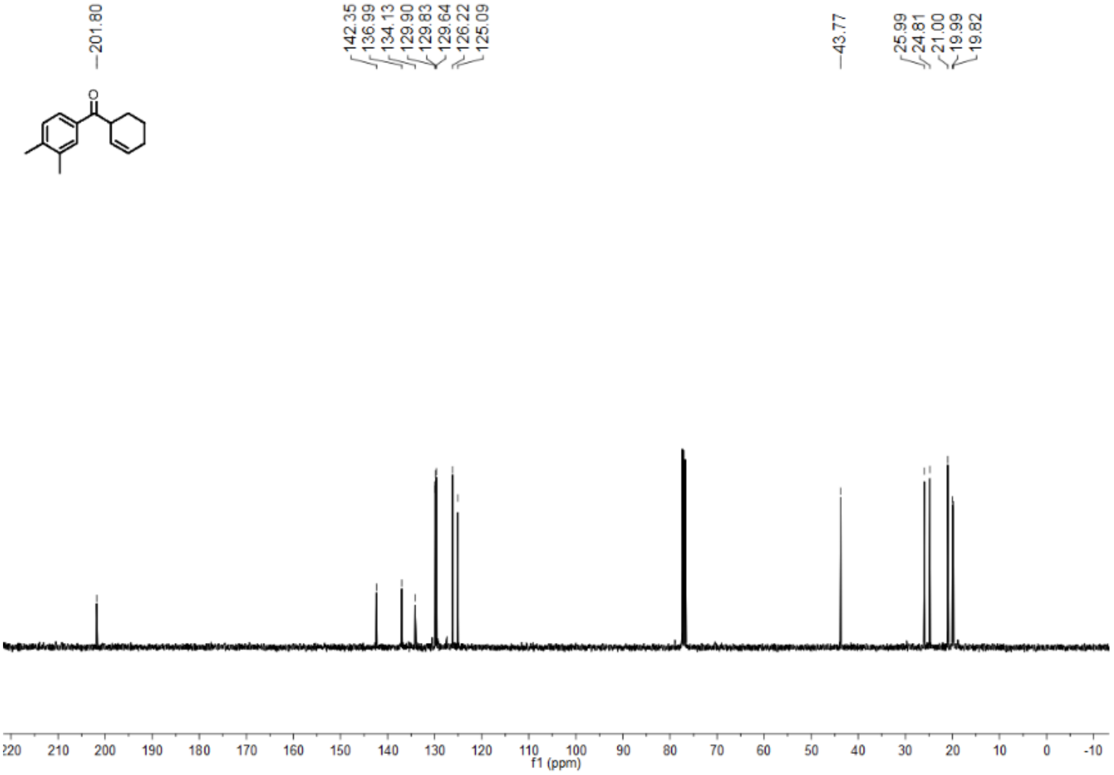

Supplementary Figure 54 ^{13}C NMR (100 MHz) spectrum of compound **23** in $CDCl_3$

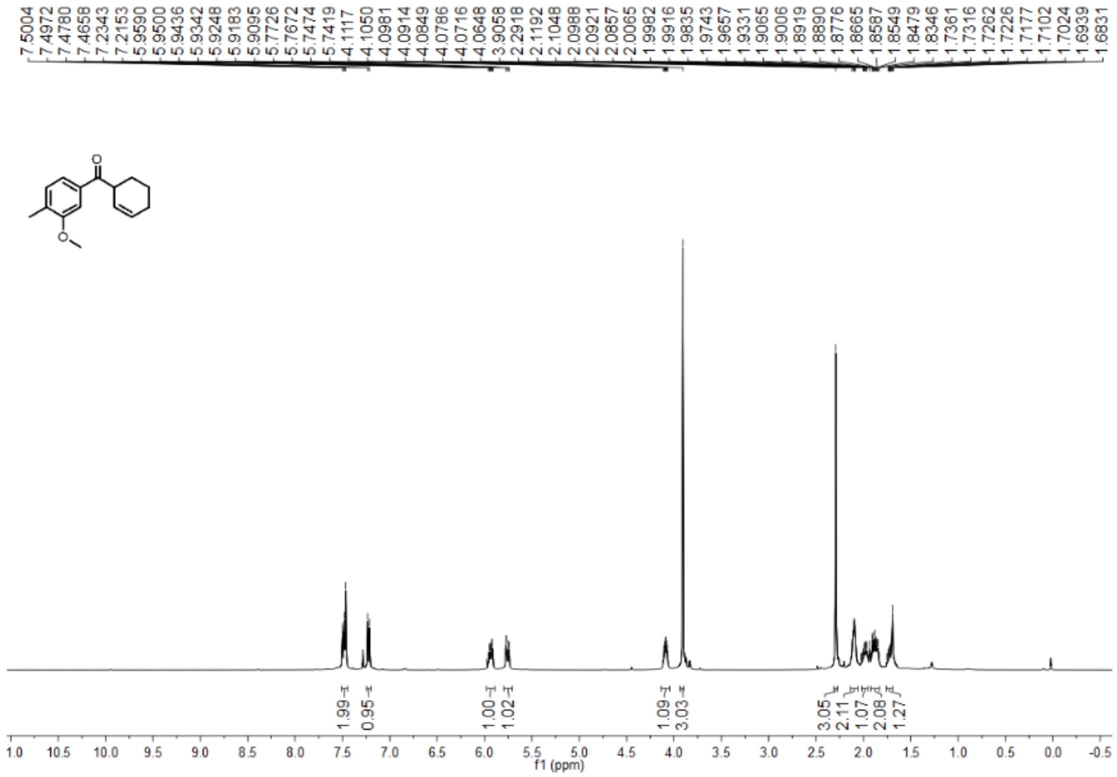

Supplementary Figure 55 ^{1}H NMR (400 MHz) spectrum of compound **24** in $CDCl_3$

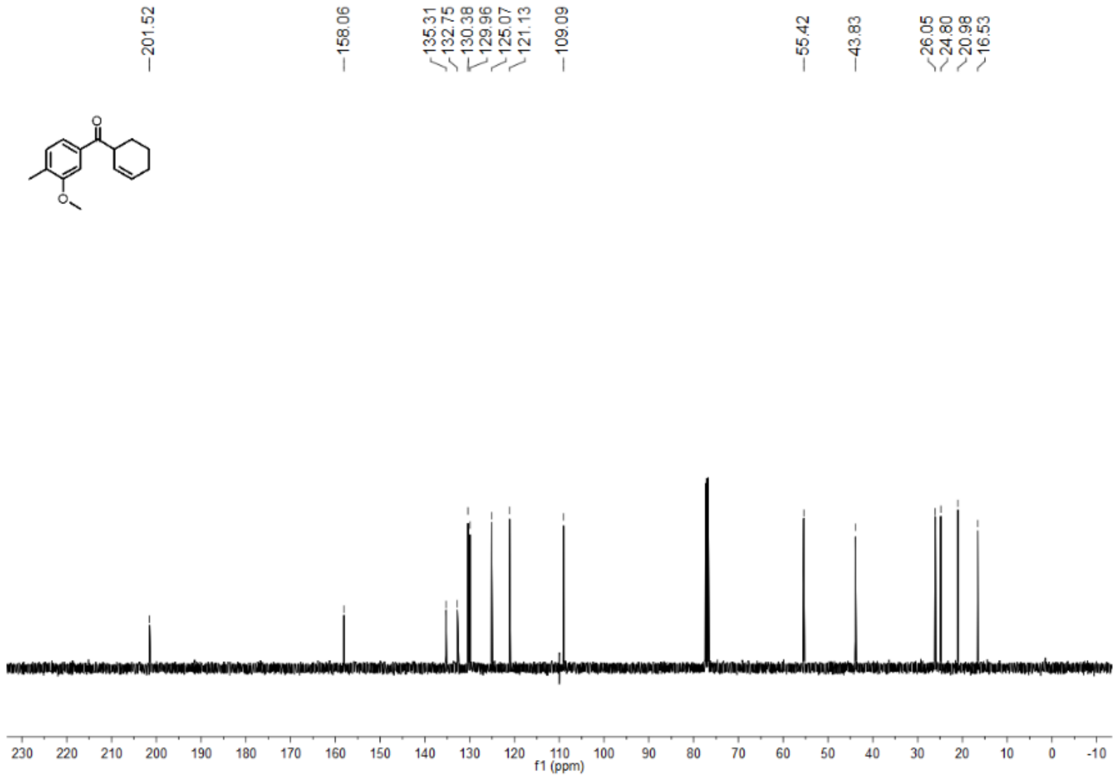

Supplementary Figure 56 ^{13}C NMR (100 MHz) spectrum of compound **24** in $CDCl_3$

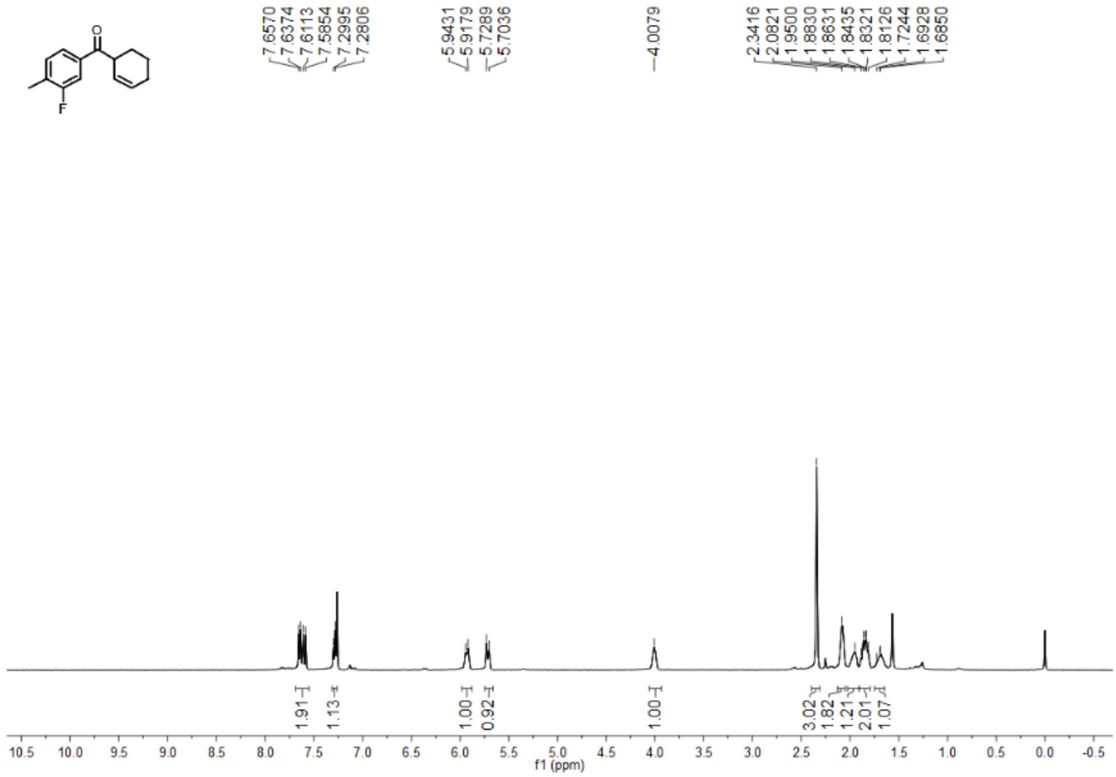

Supplementary Figure 57 ^{1}H NMR (400 MHz) spectrum of compound **25** in $CDCl_3$

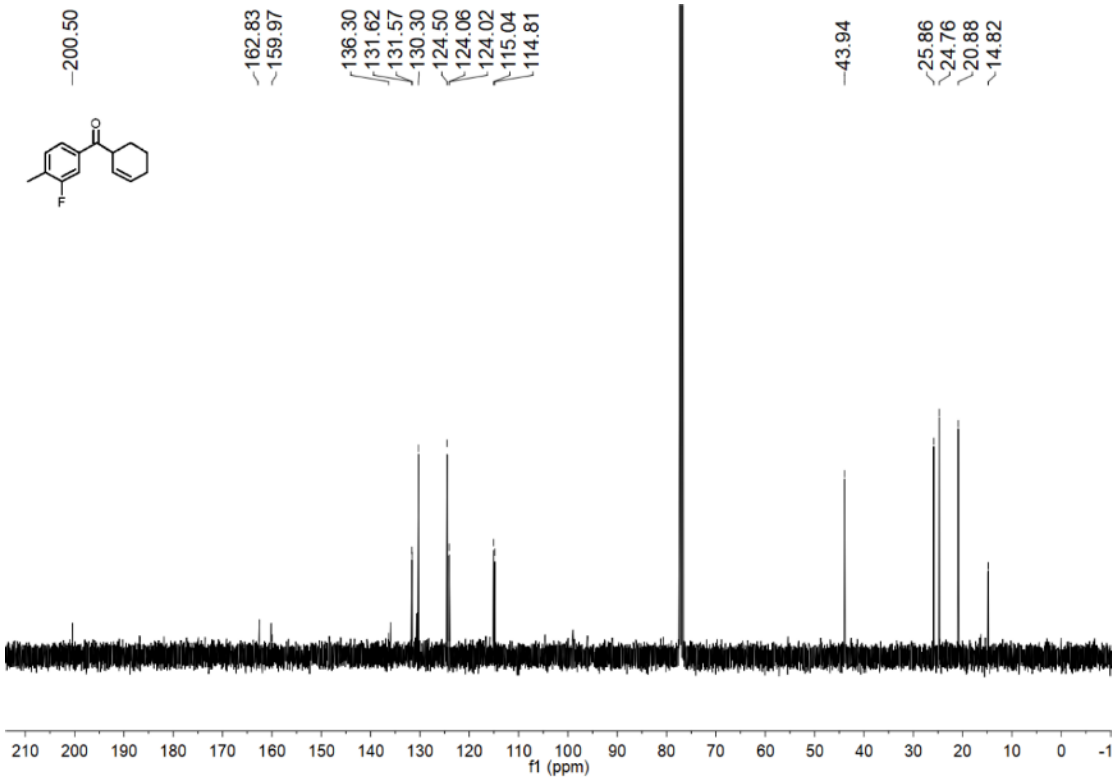

Supplementary Figure 58 ^{13}C NMR (100 MHz) spectrum of compound **25** in $CDCl_3$

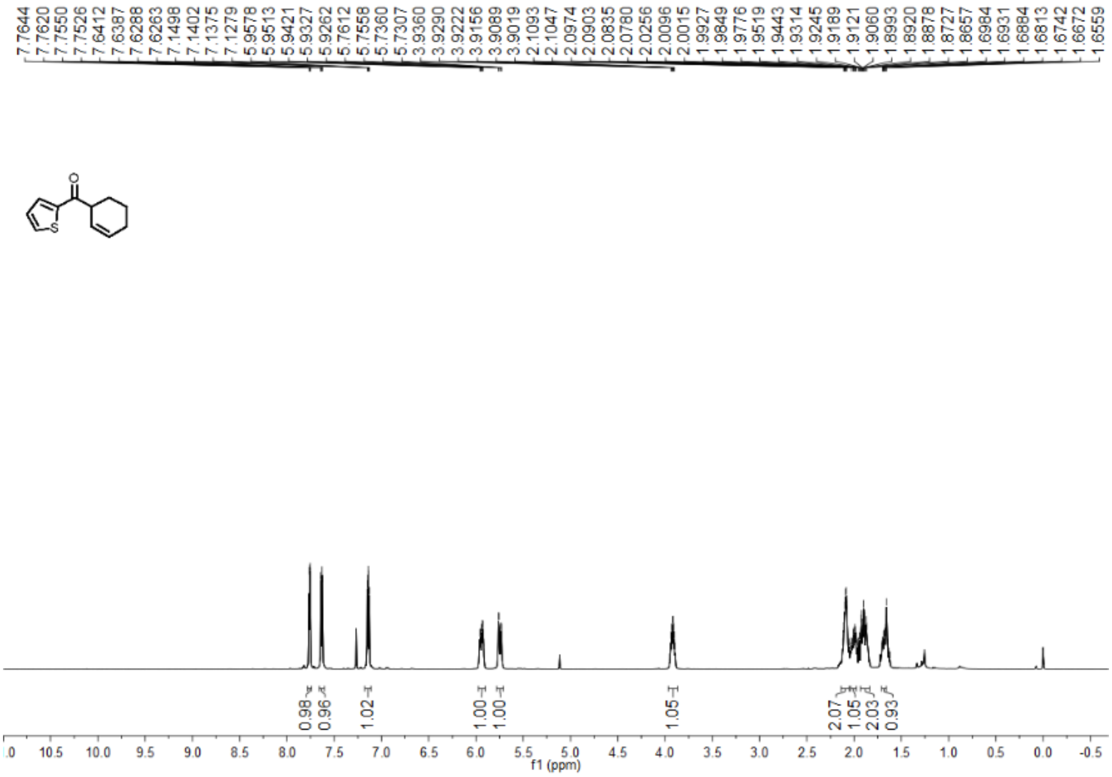

Supplementary Figure 59 ^{1}H NMR (400 MHz) spectrum of compound **26** in $CDCl_3$

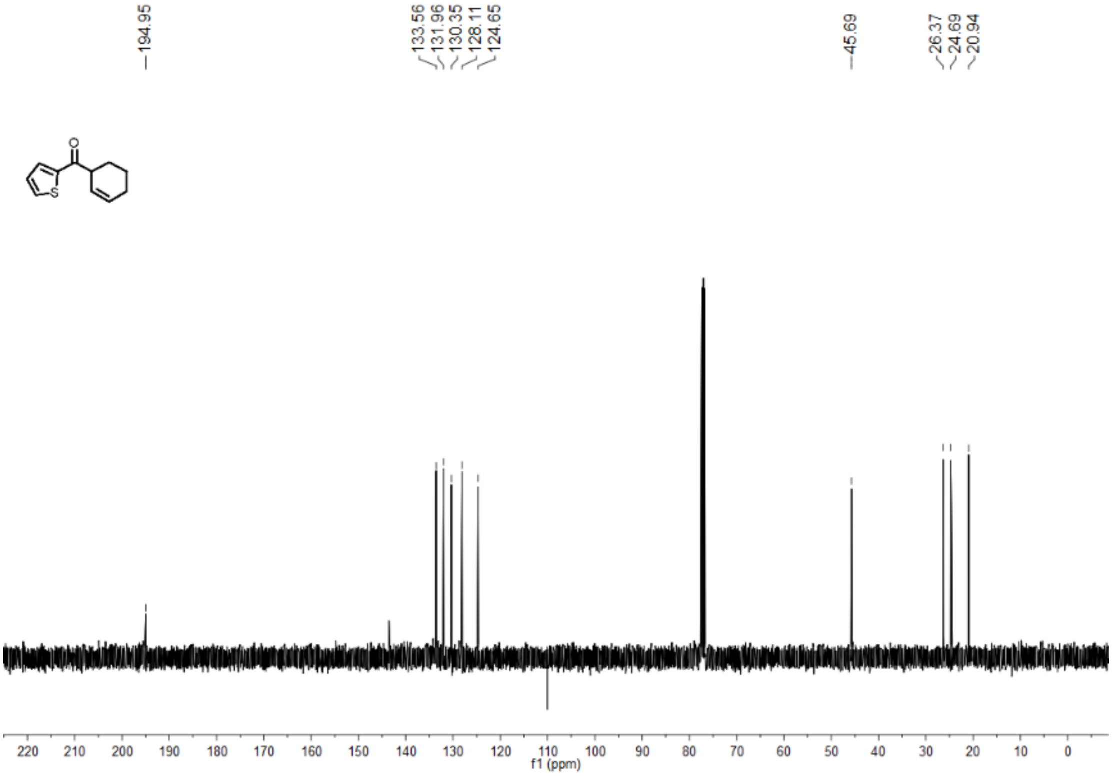

Supplementary Figure 60 ^{13}C NMR (100 MHz) spectrum of compound **26** in $CDCl_3$

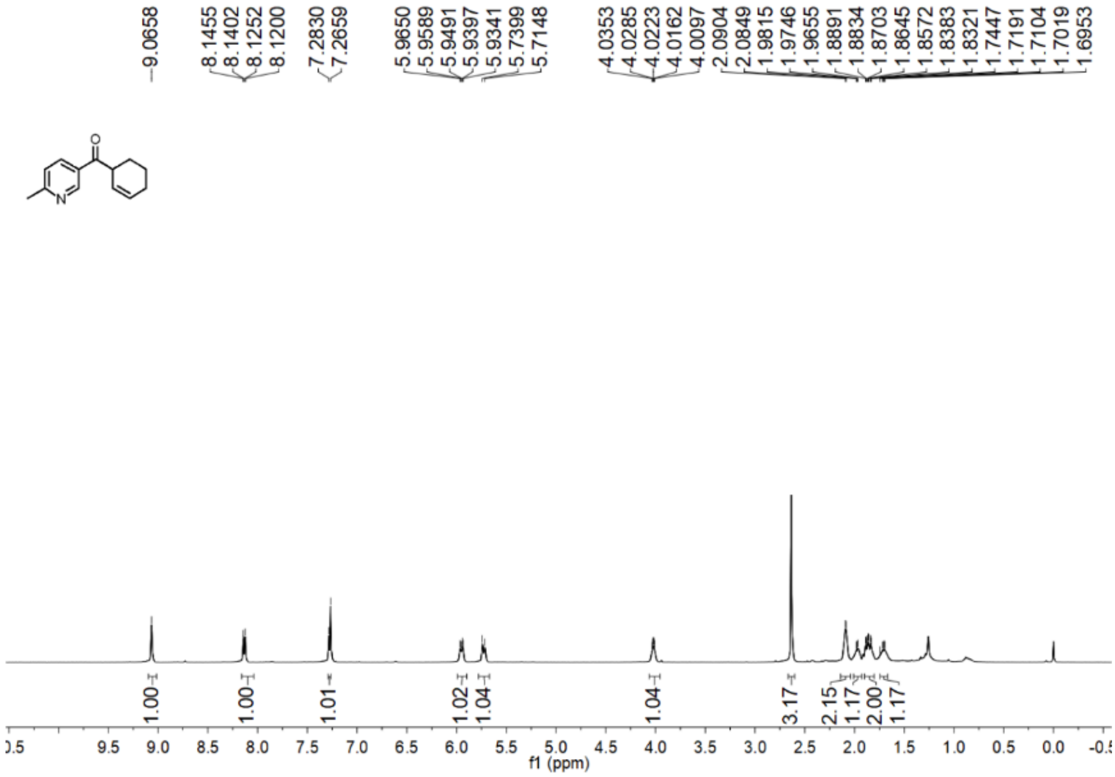

Supplementary Figure 61 ^{1}H NMR (400 MHz) spectrum of compound **27** in $CDCl_3$

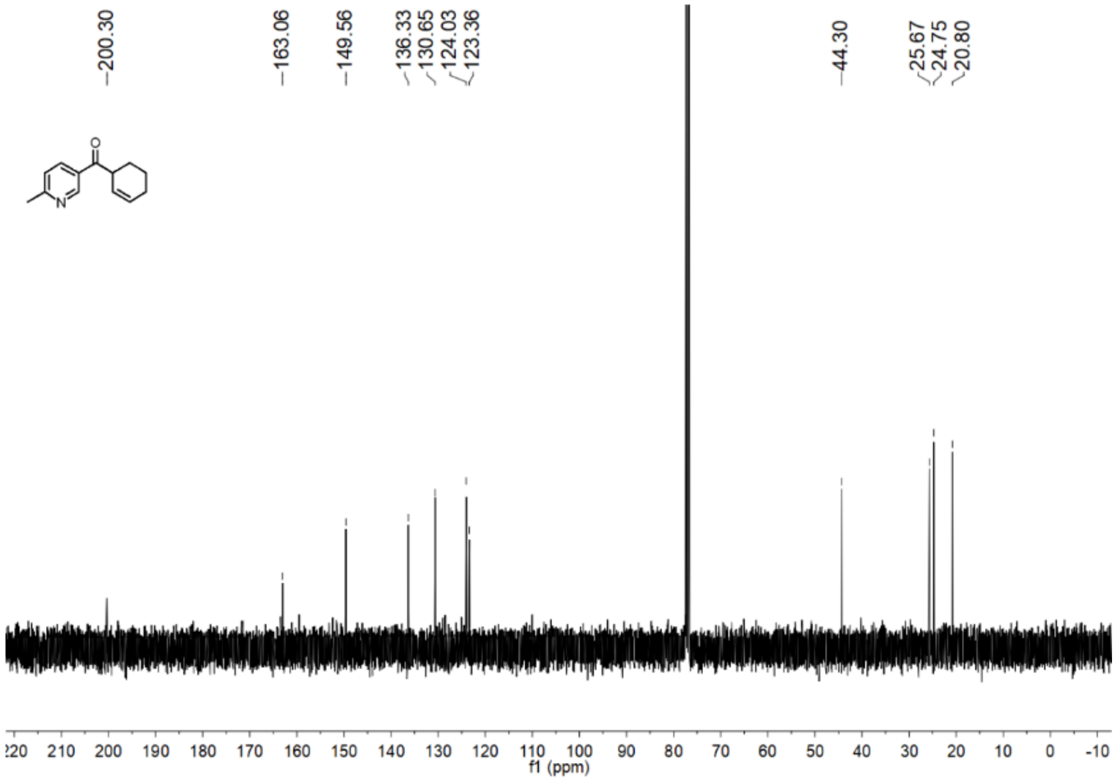

Supplementary Figure 62 ^{13}C NMR (100 MHz) spectrum of compound **27** in $CDCl_3$

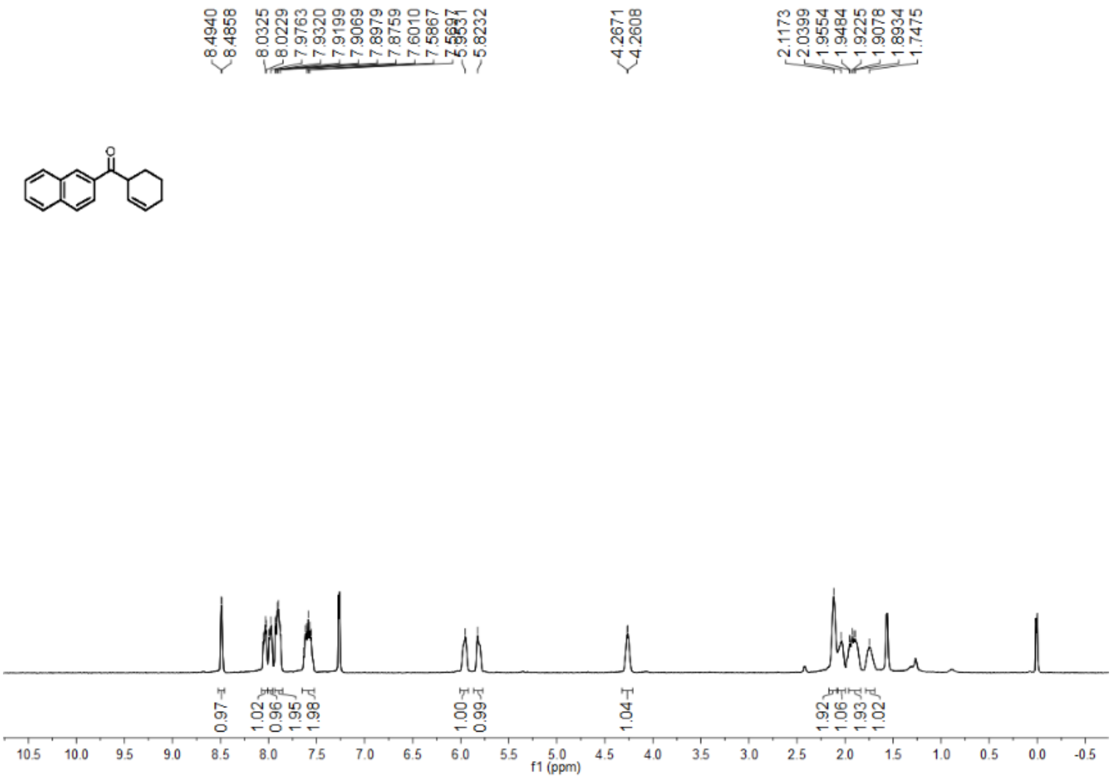

Supplementary Figure 63 ^{1}H NMR (400 MHz) spectrum of compound **28** in $CDCl_3$

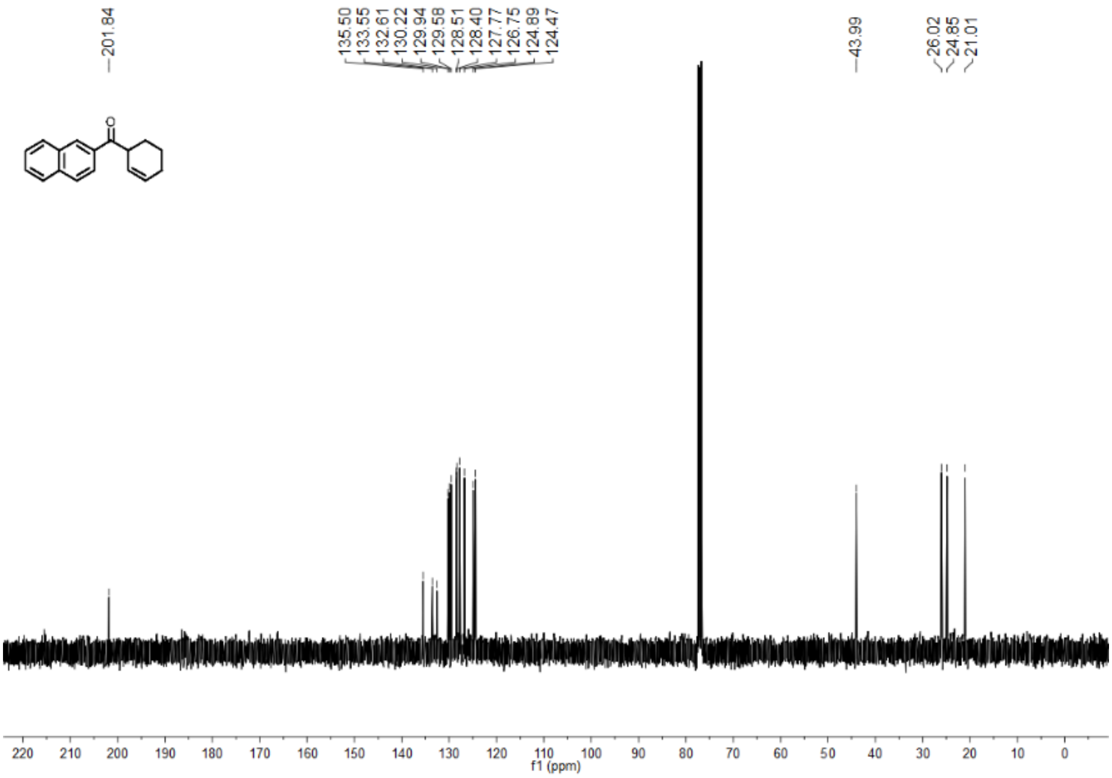

Supplementary Figure 64 ^{13}C NMR (100 MHz) spectrum of compound **28** in $CDCl_3$

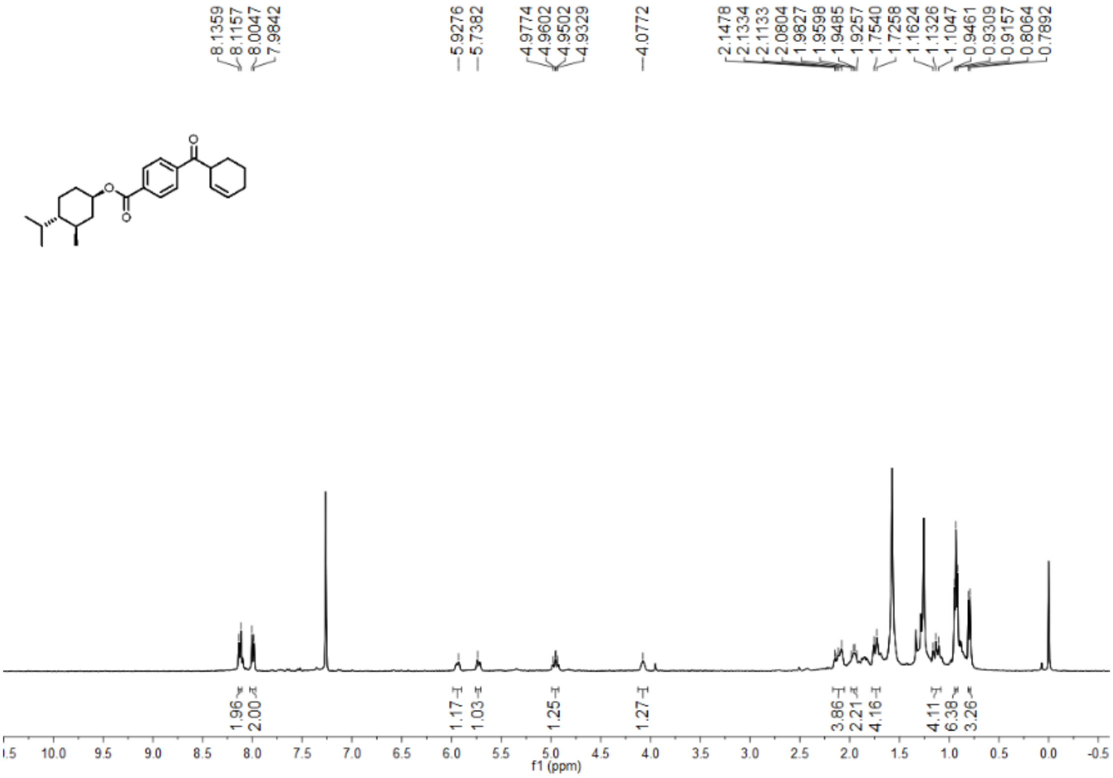

Supplementary Figure 65 ^{1}H NMR (400 MHz) spectrum of compound **29** in $CDCl_3$

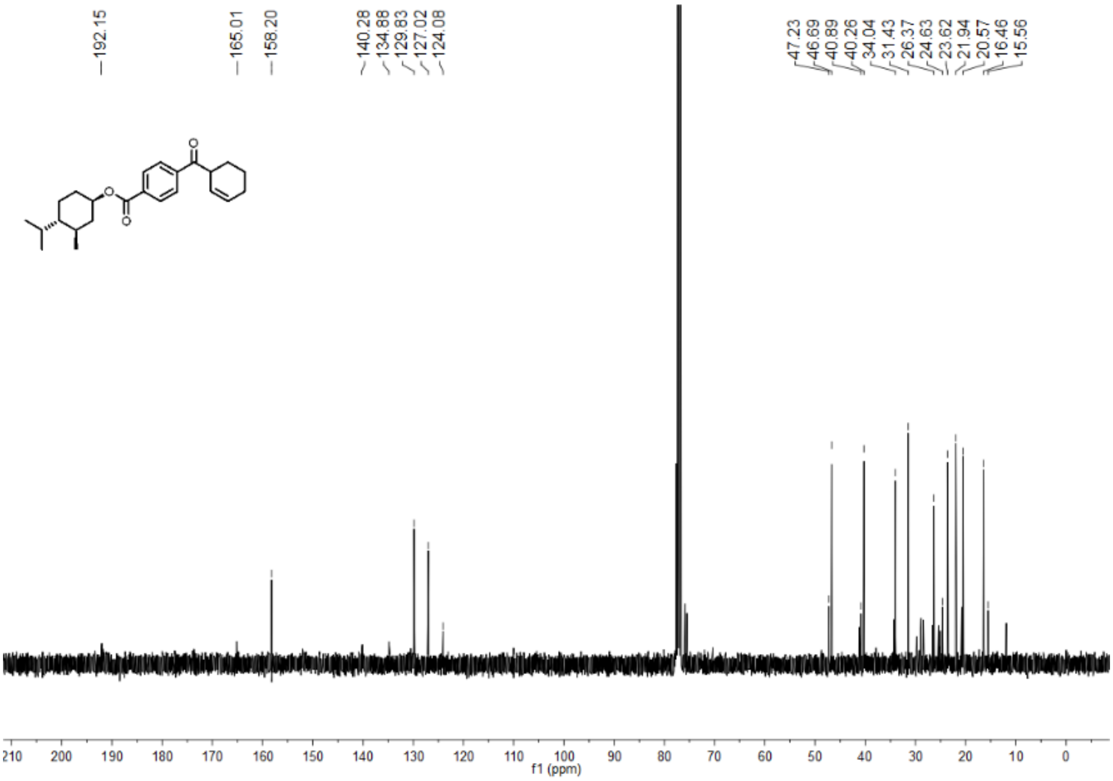

Supplementary Figure 66 ^{13}C NMR (100 MHz) spectrum of compound **29** in $CDCl_3$

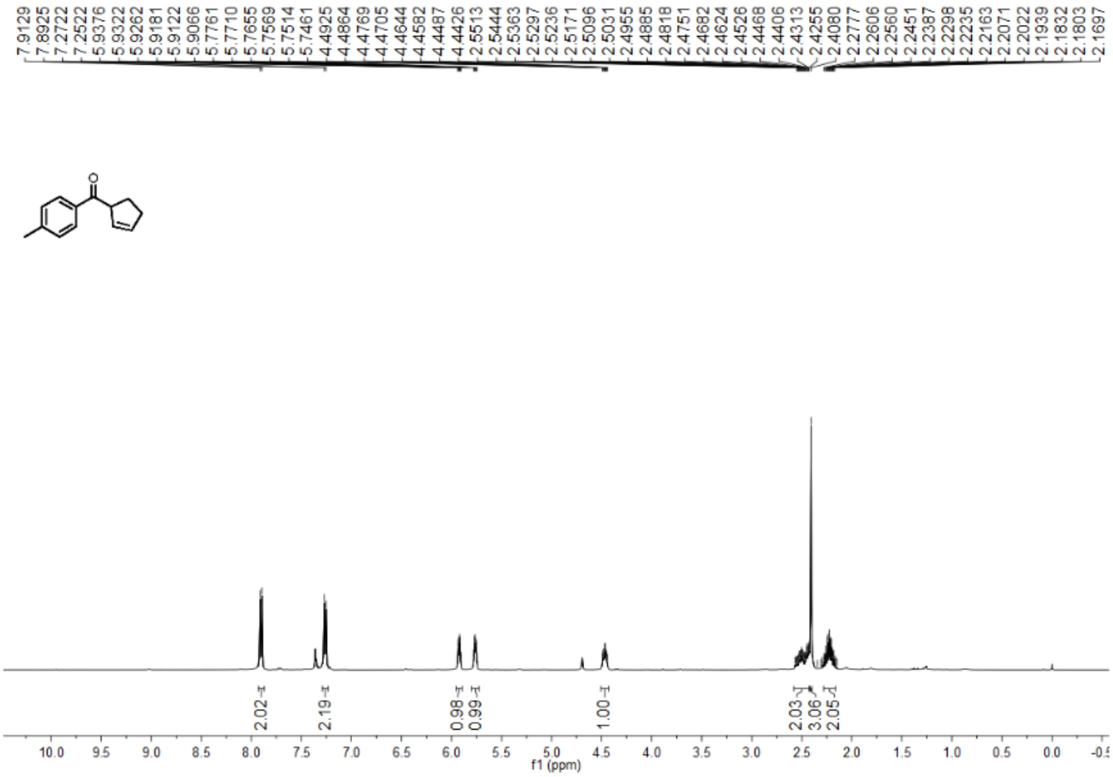

Supplementary Figure 67 ^{1}H NMR (400 MHz) spectrum of compound **30** in $CDCl_3$

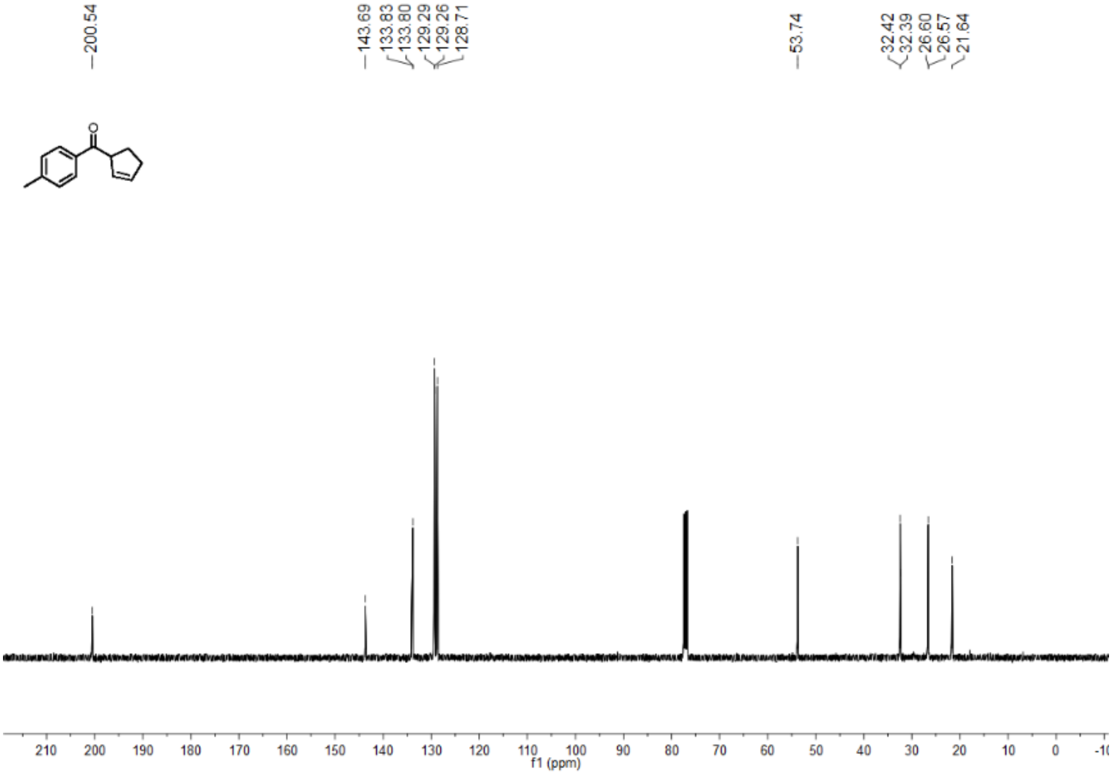

Supplementary Figure 68 ^{13}C NMR (100 MHz) spectrum of compound **30** in $CDCl_3$

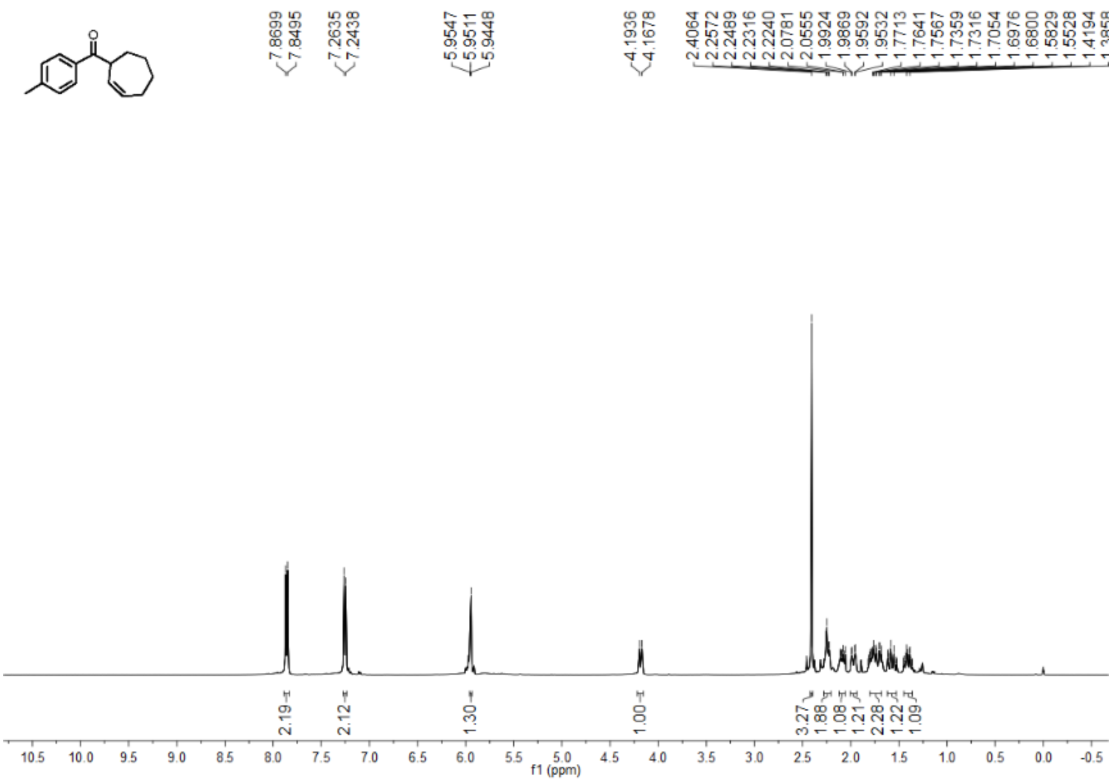

Supplementary Figure 69 ^{1}H NMR (400 MHz) spectrum of compound **31** in $CDCl_3$

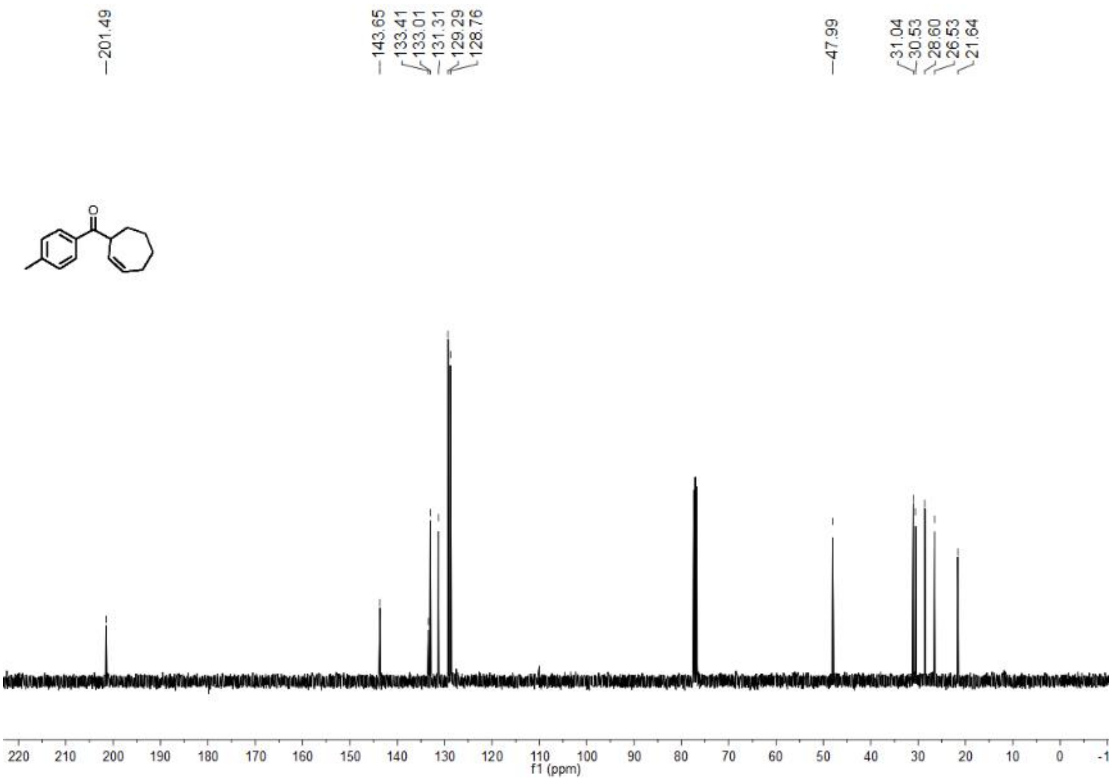

Supplementary Figure 70 ^{13}C NMR (100 MHz) spectrum of compound **31** in $CDCl_3$

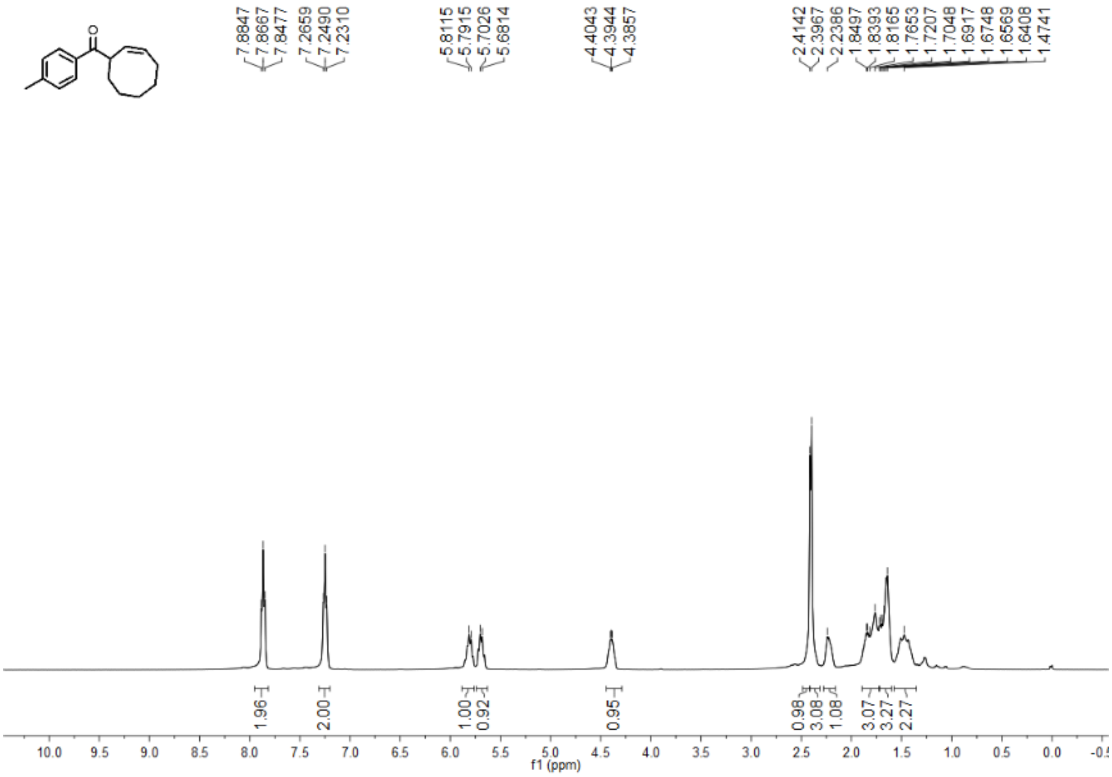

Supplementary Figure 71 ^{1}H NMR (400 MHz) spectrum of compound **32** in $CDCl_3$

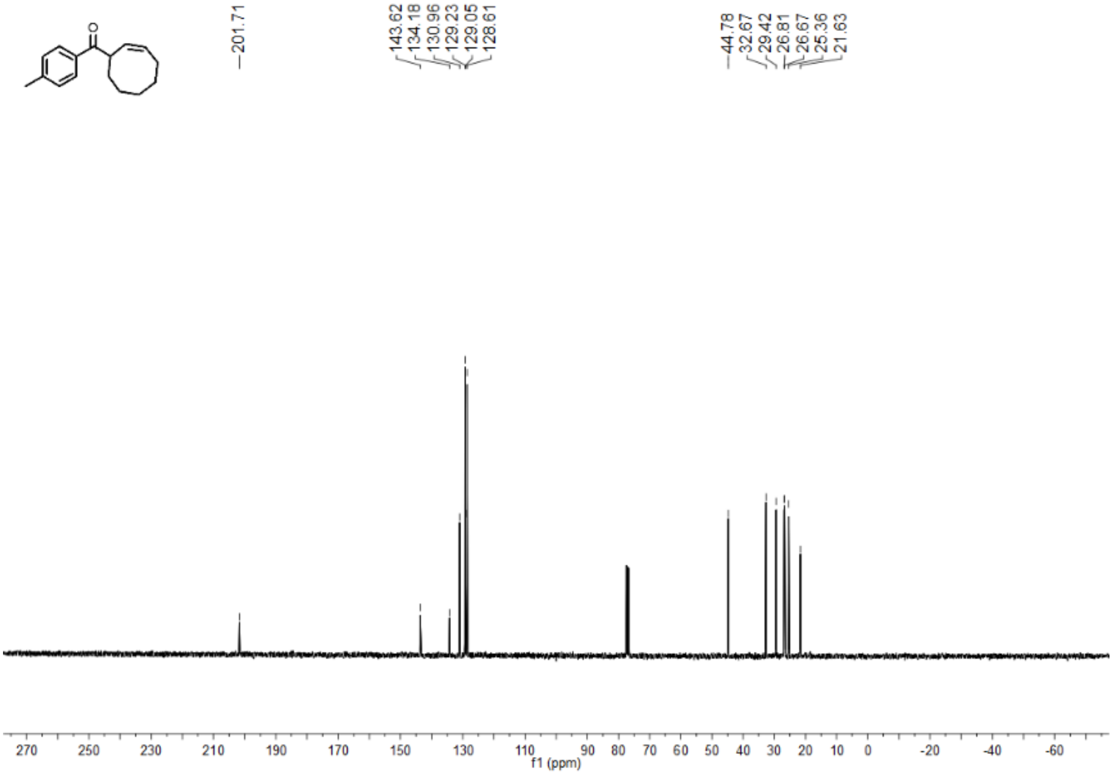

Supplementary Figure 72 ^{13}C NMR (100 MHz) spectrum of compound **32** in $CDCl_3$

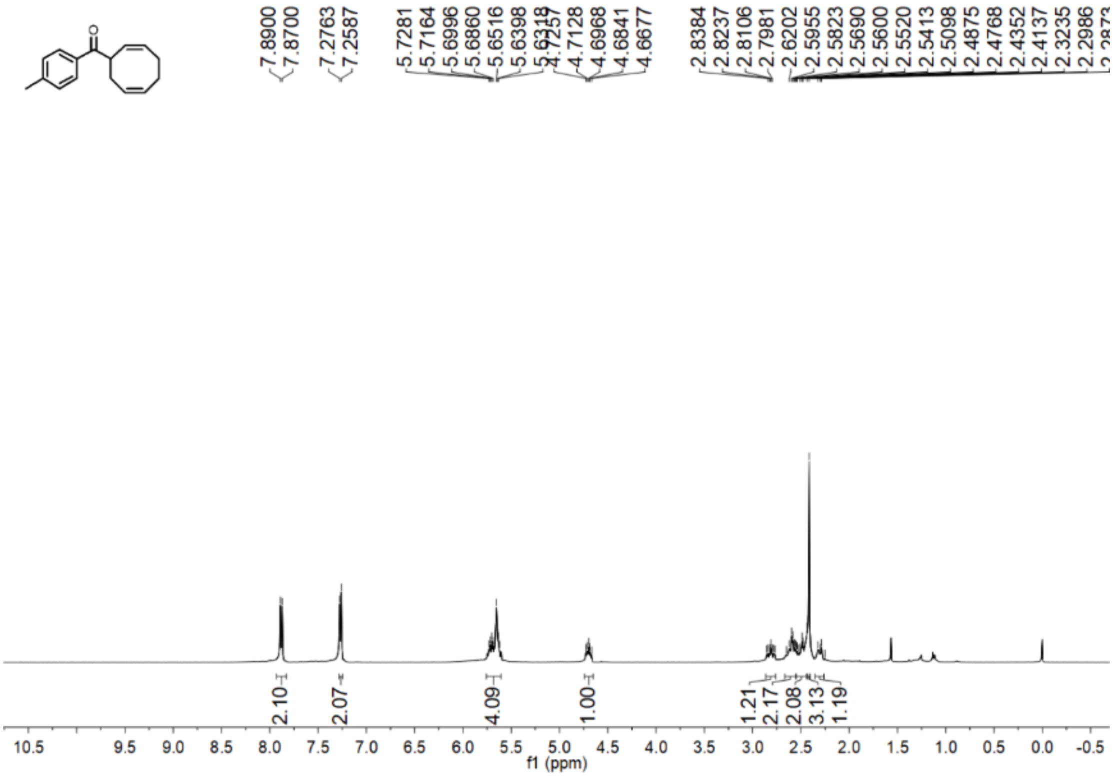

Supplementary Figure 73 ^{1}H NMR (400 MHz) spectrum of compound **33** in $CDCl_3$

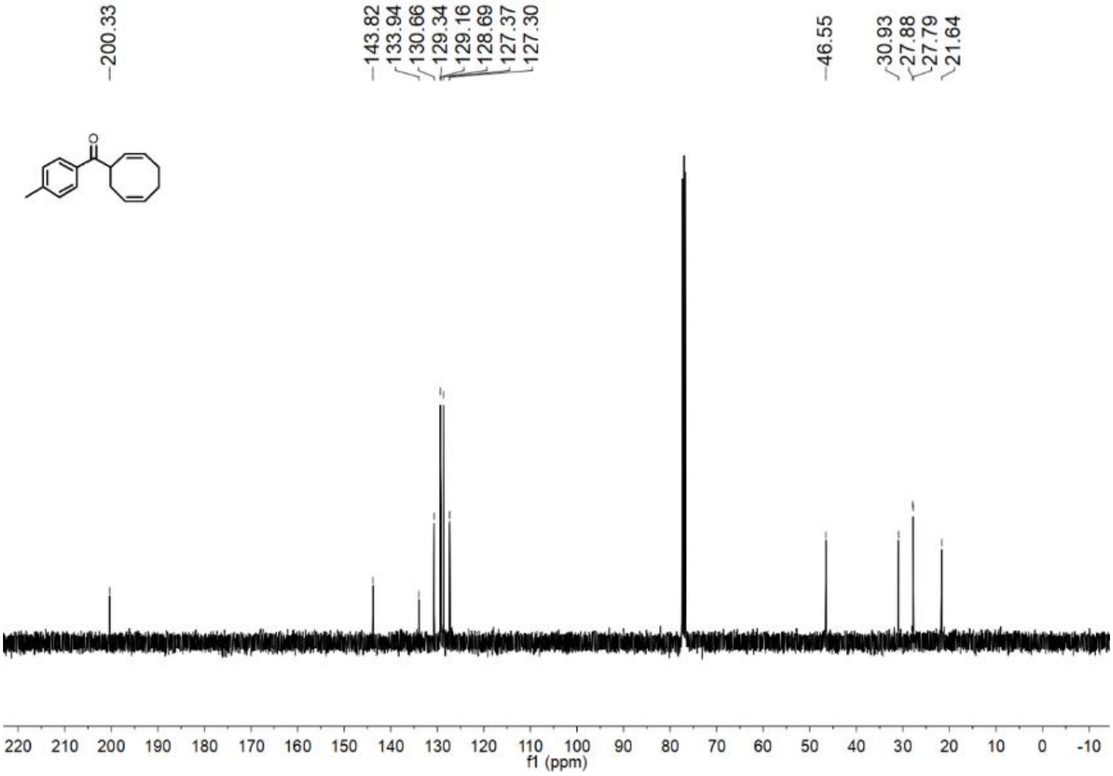

Supplementary Figure 74 ^{13}C NMR (100 MHz) spectrum of compound **33** in $CDCl_3$

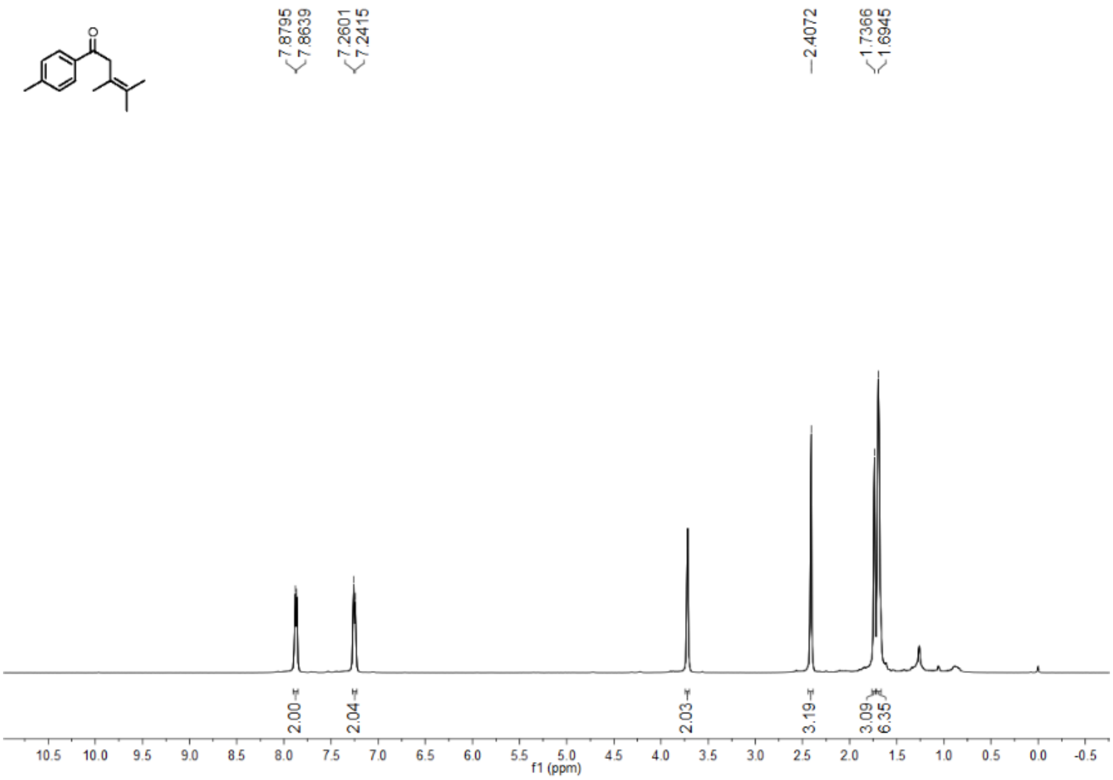

Supplementary Figure 75 ^{1}H NMR (400 MHz) spectrum of compound **34** in $CDCl_3$

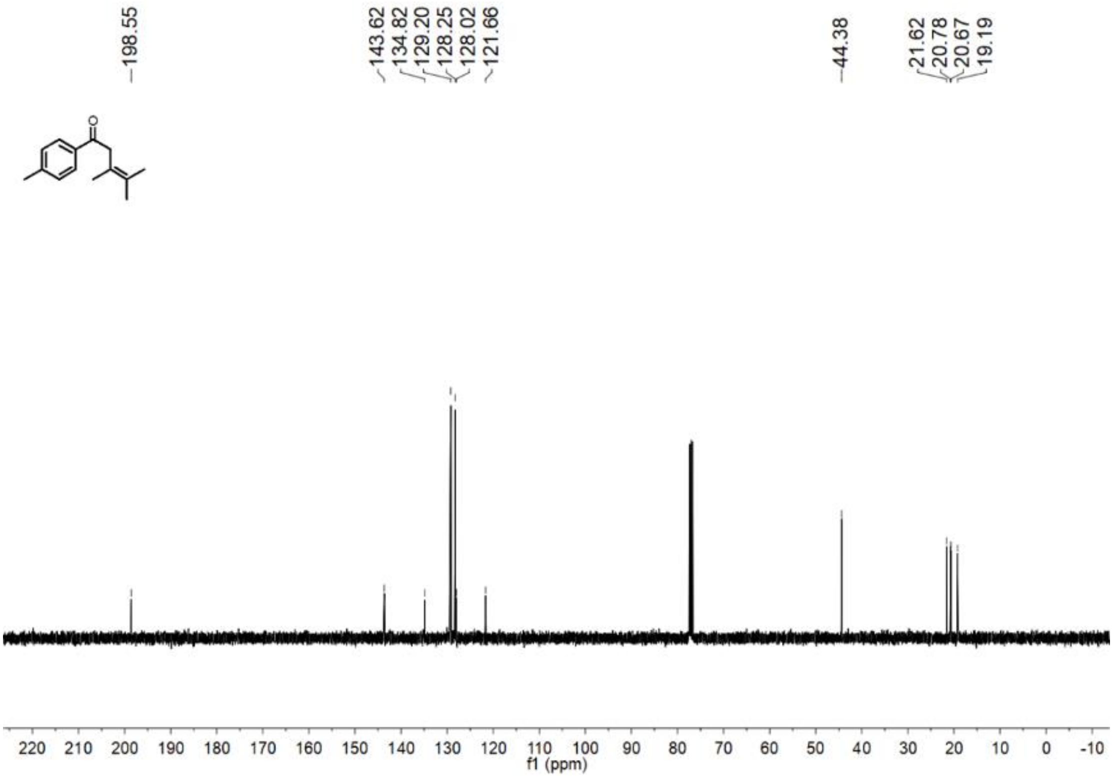

Supplementary Figure 76 ^{13}C NMR (100 MHz) spectrum of compound **34** in $CDCl_3$

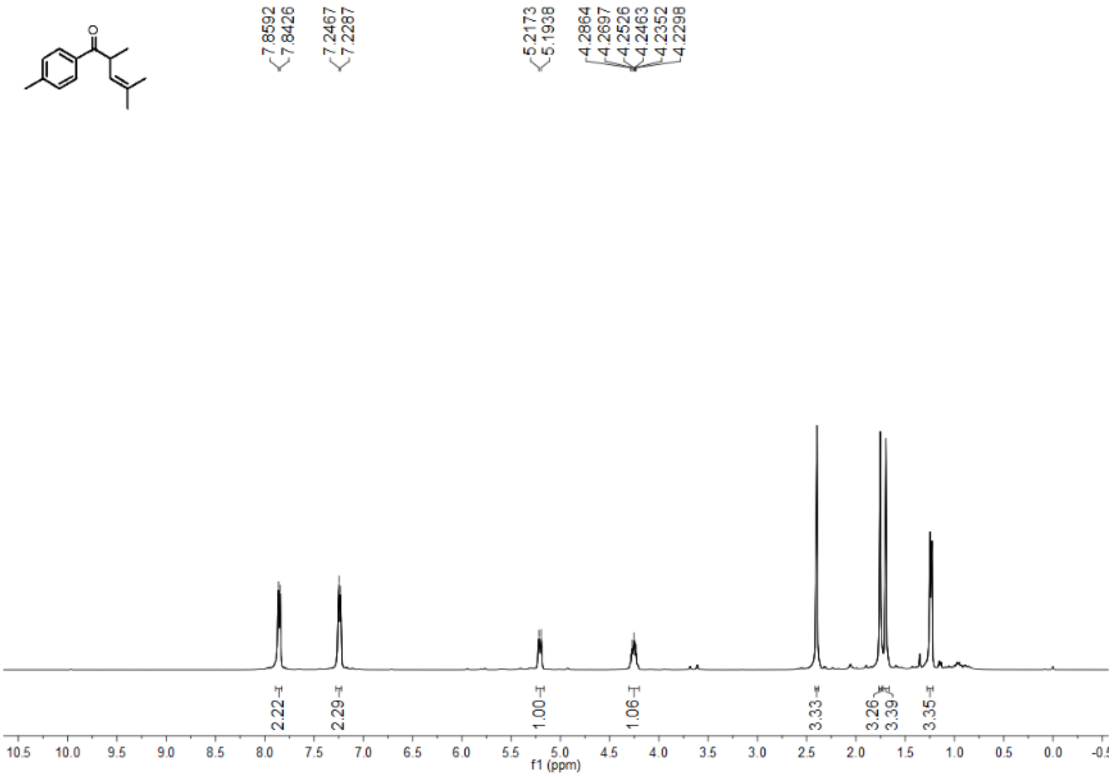

Supplementary Figure 77 ^{1}H NMR (400 MHz) spectrum of compound **35** in $CDCl_3$

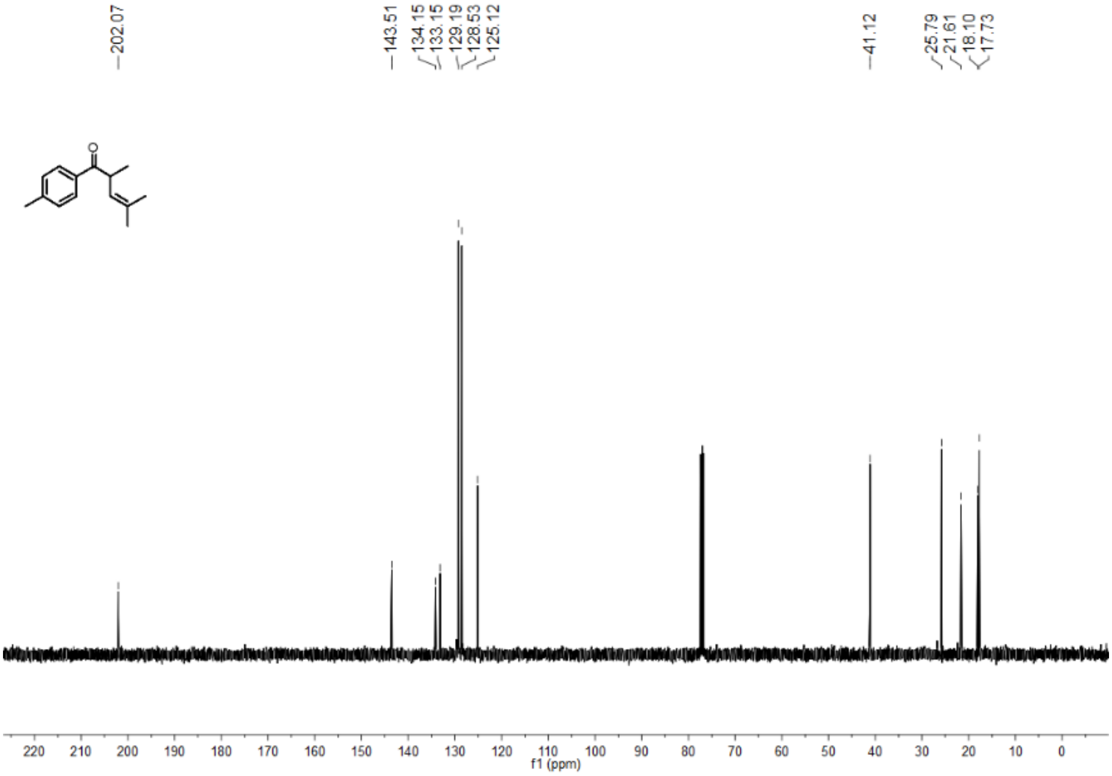

Supplementary Figure 78 ^{13}C NMR (100 MHz) spectrum of compound **35** in $CDCl_3$

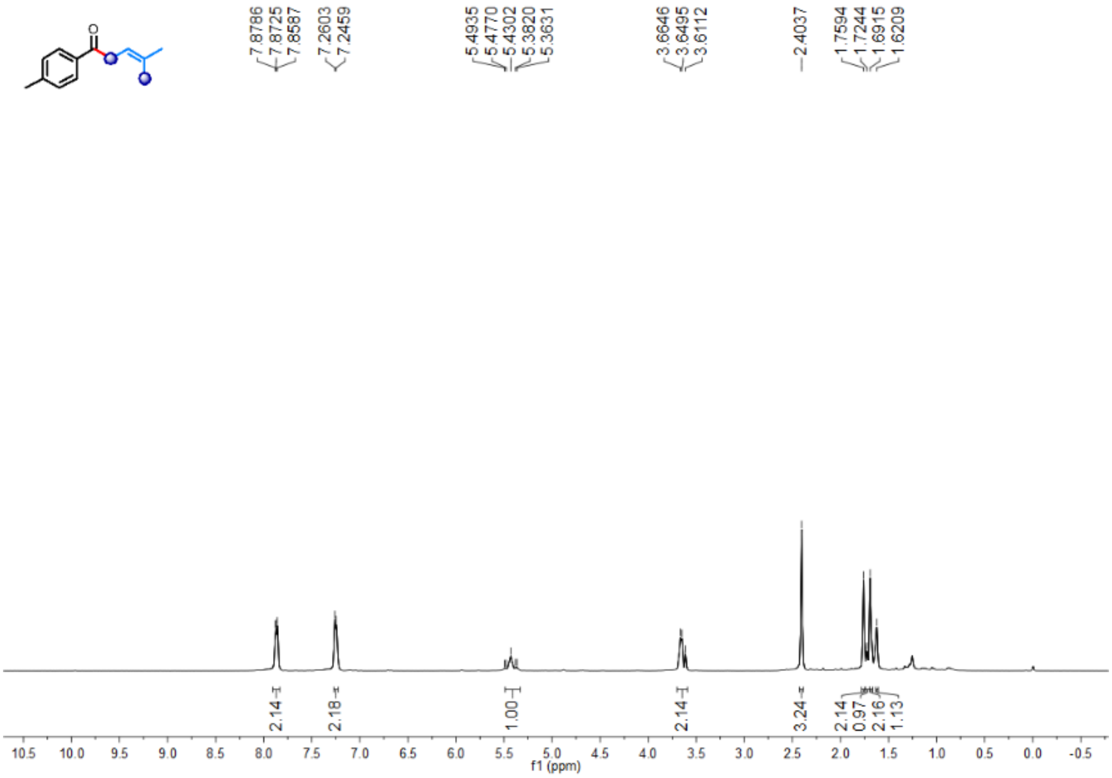

Supplementary Figure 79 ^{1}H NMR (400 MHz) spectrum of compound **36** in $CDCl_3$

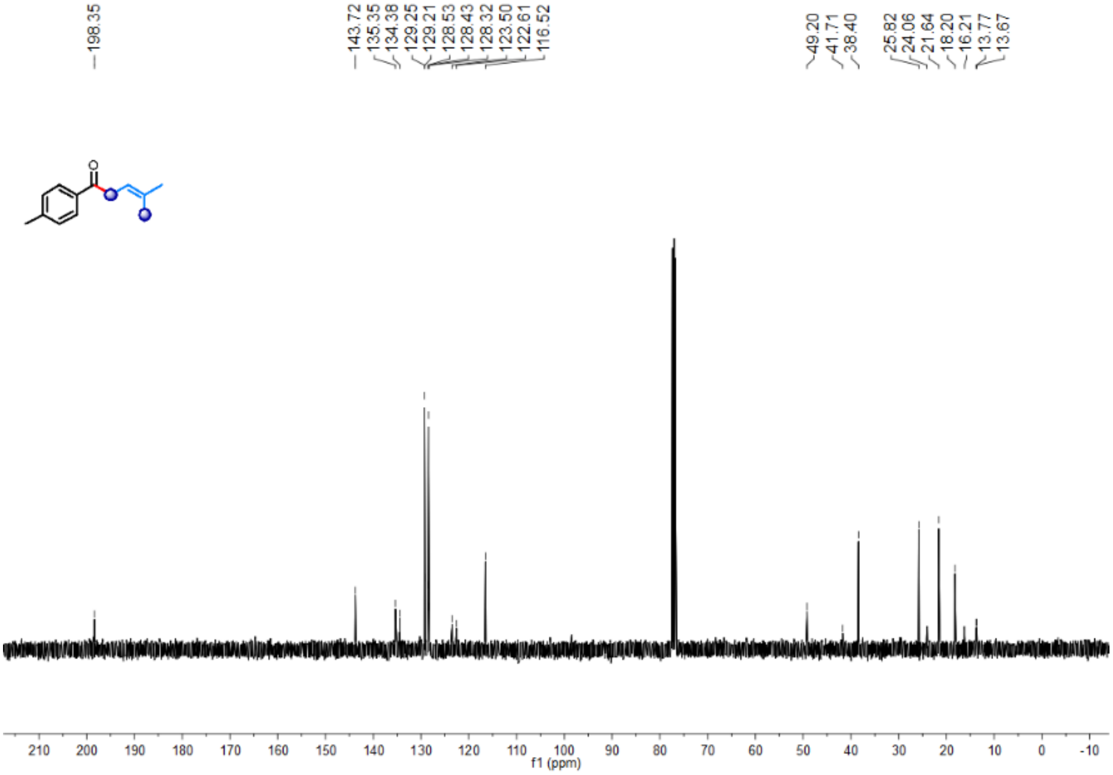

Supplementary Figure 80 ^{13}C NMR (100 MHz) spectrum of compound **36** in $CDCl_3$

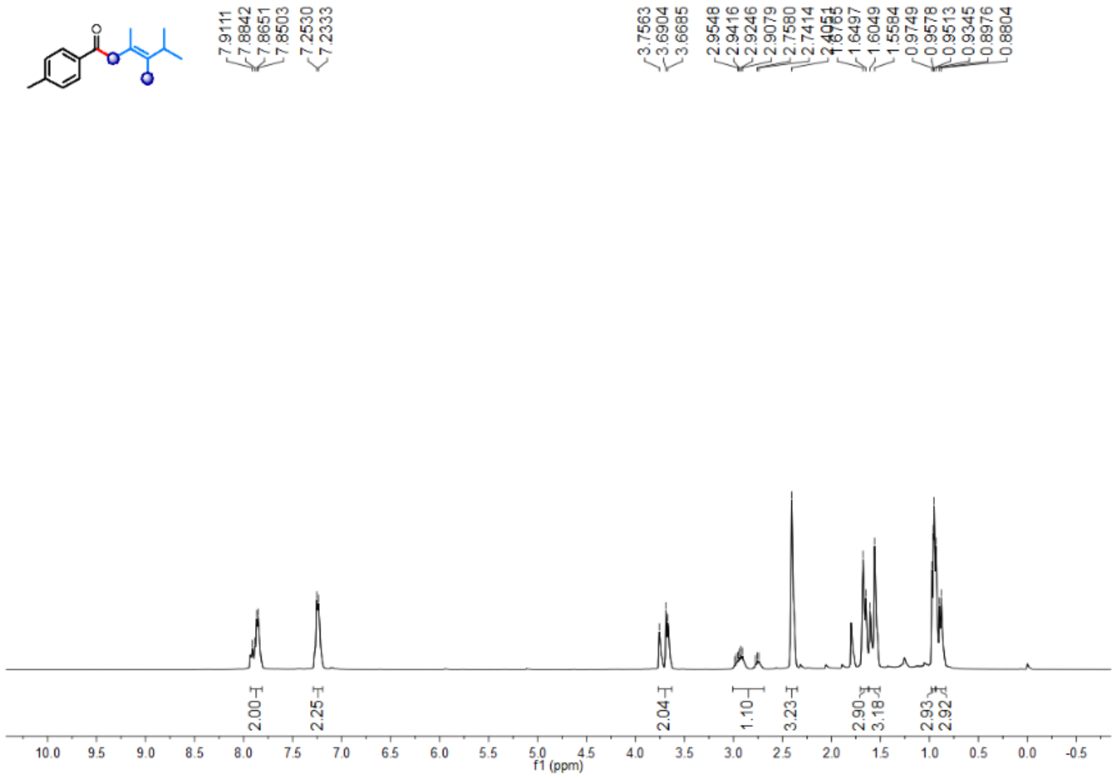

Supplementary Figure 81 ^{1}H NMR (400 MHz) spectrum of compound **37** in $CDCl_3$

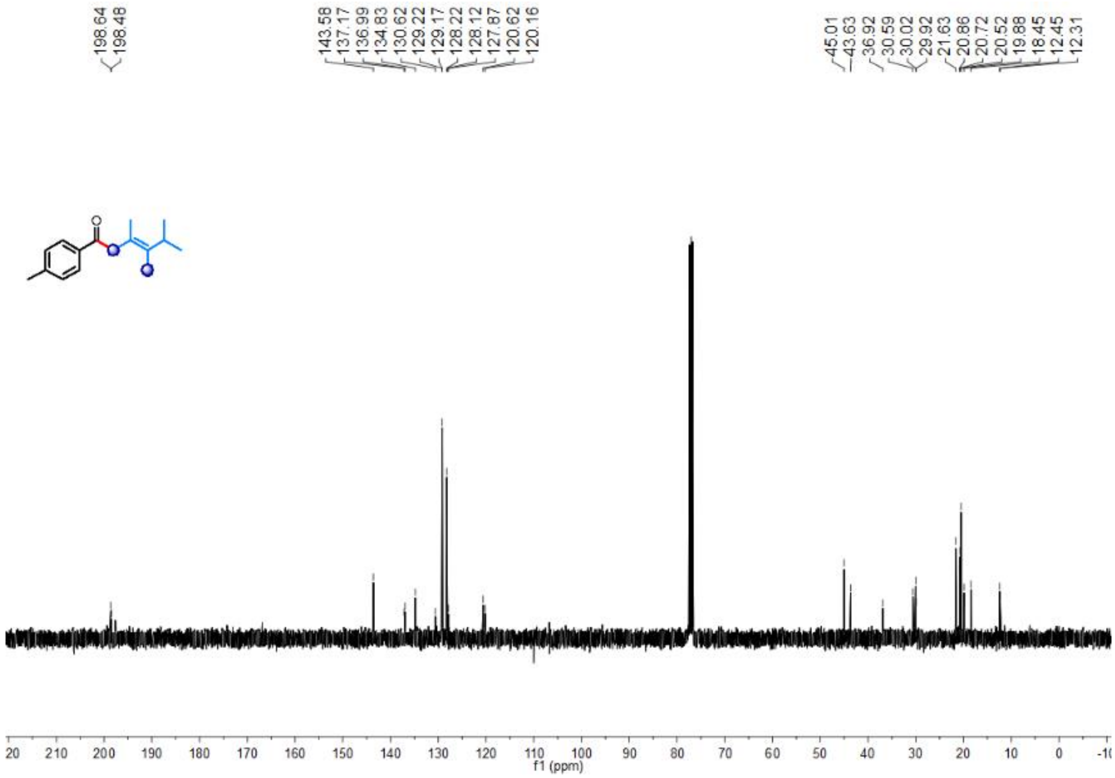

Supplementary Figure 82 ^{13}C NMR (100 MHz) spectrum of compound **37** in $CDCl_3$

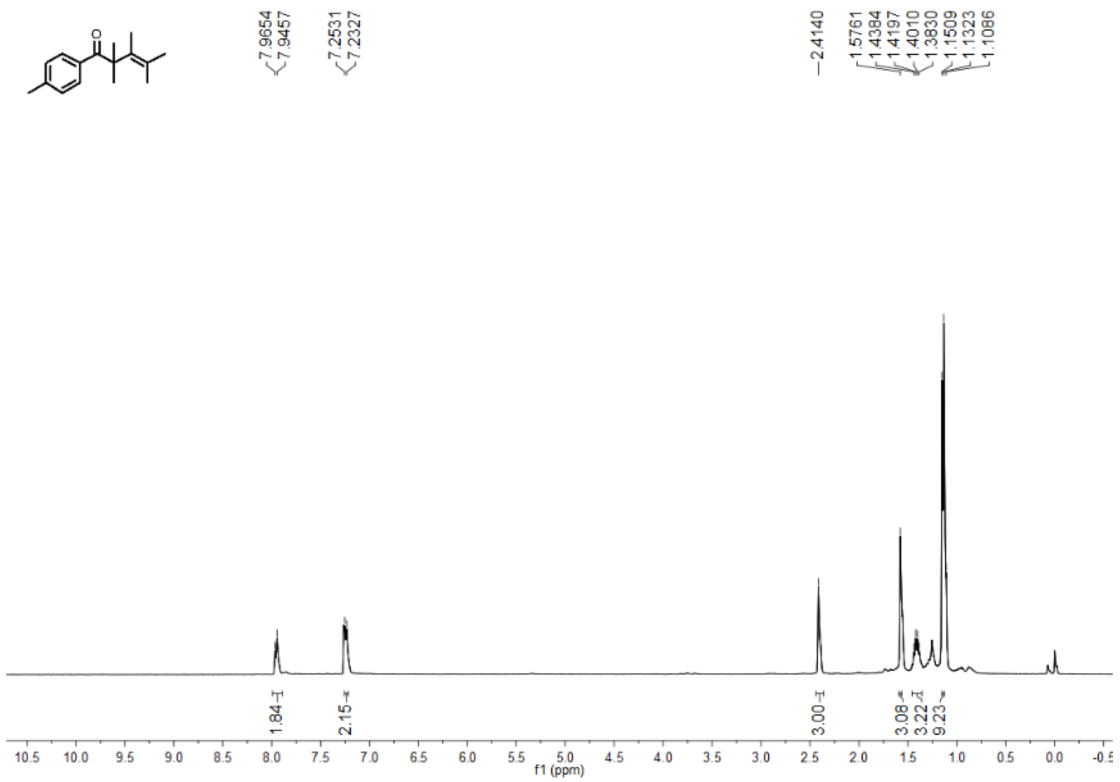

Supplementary Figure 83 ^{1}H NMR (400 MHz) spectrum of compound **37’** in $CDCl_3$

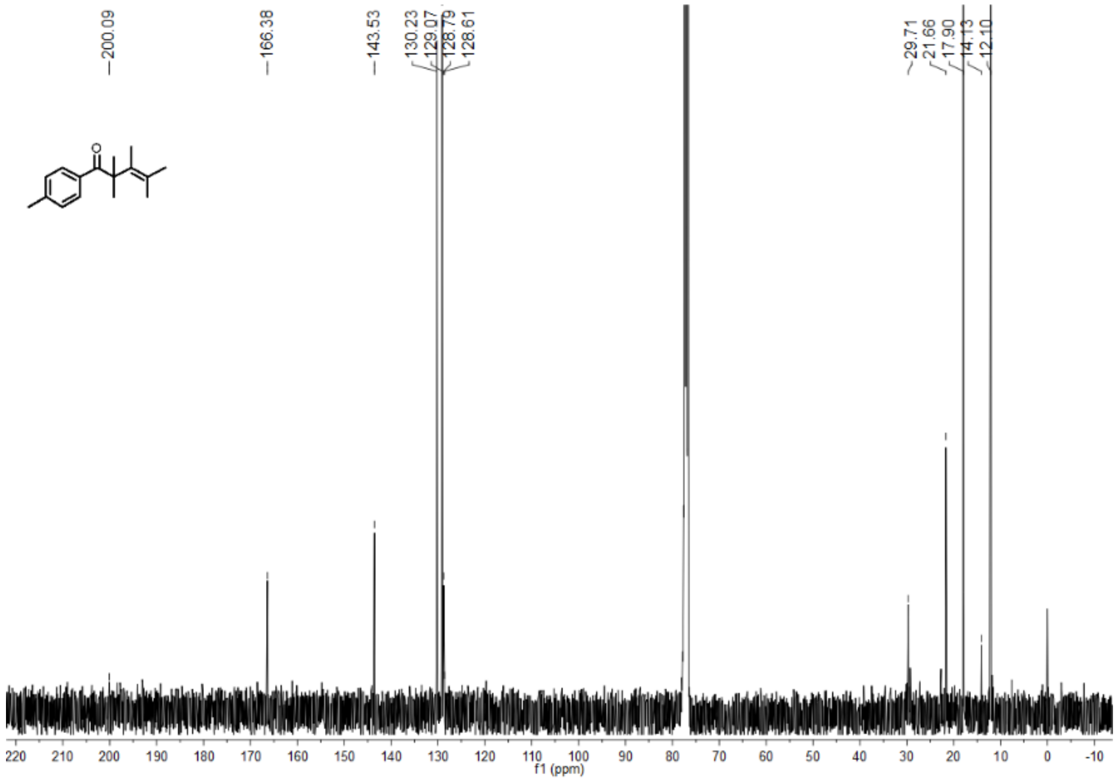

Supplementary Figure 84 ^{13}C NMR (100 MHz) spectrum of compound **37’** in $CDCl_3$

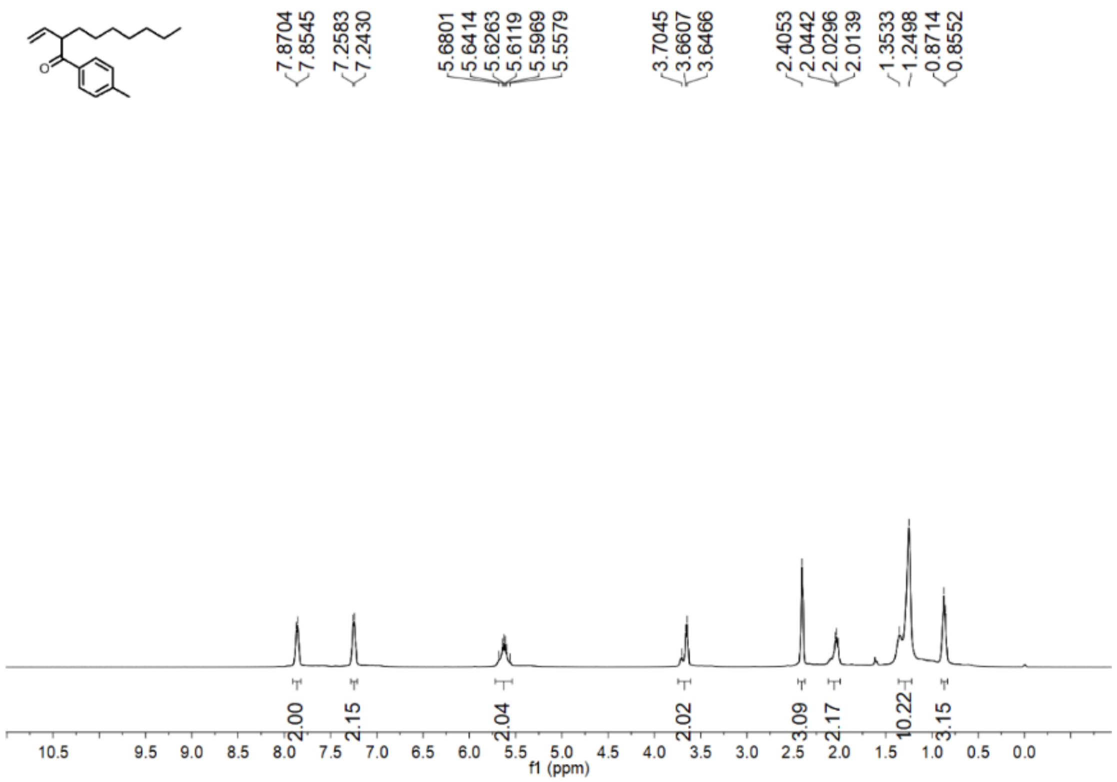

Supplementary Figure 85 ^{1}H NMR (400 MHz) spectrum of compound **38** in $CDCl_3$

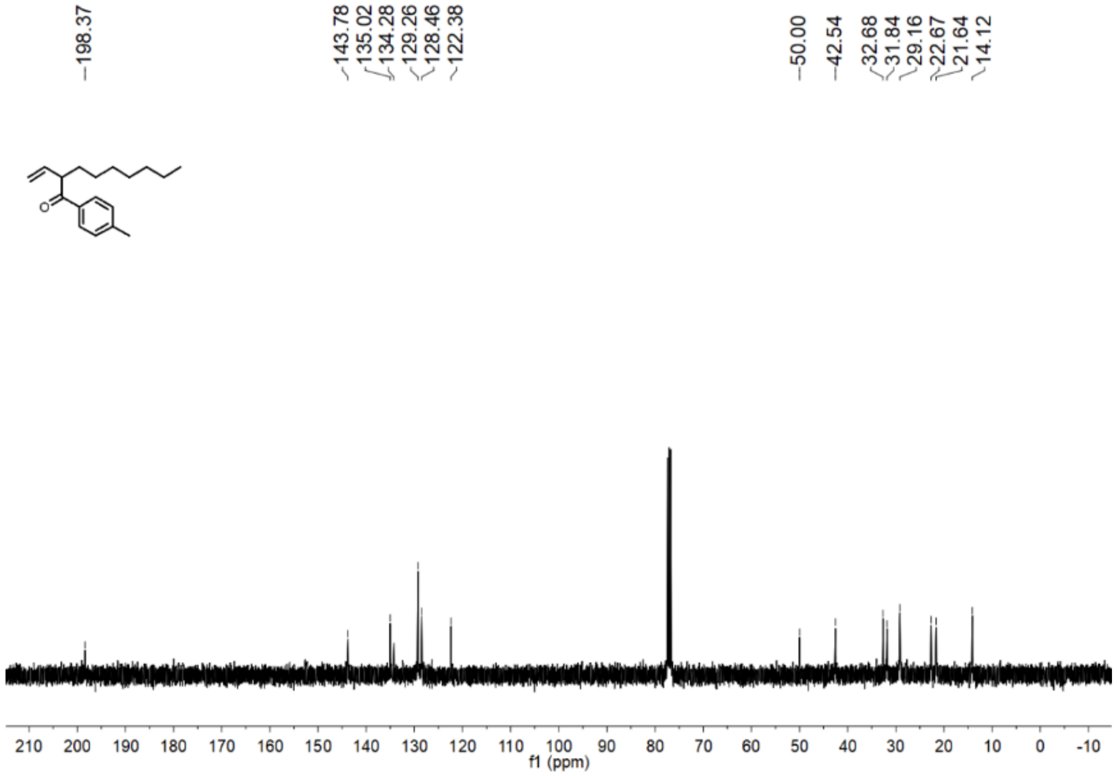

Supplementary Figure 86 ^{13}C NMR (100 MHz) spectrum of compound **38** in $CDCl_3$

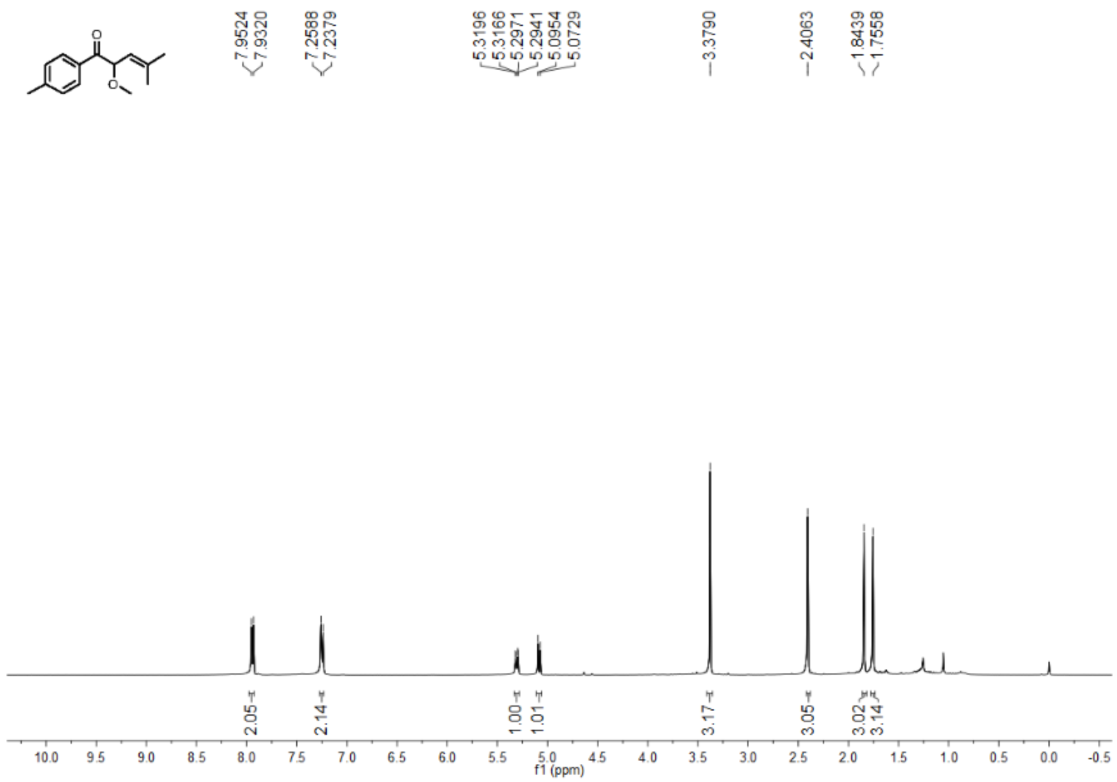

Supplementary Figure 87 ^{1}H NMR (400 MHz) spectrum of compound **39** in $CDCl_3$

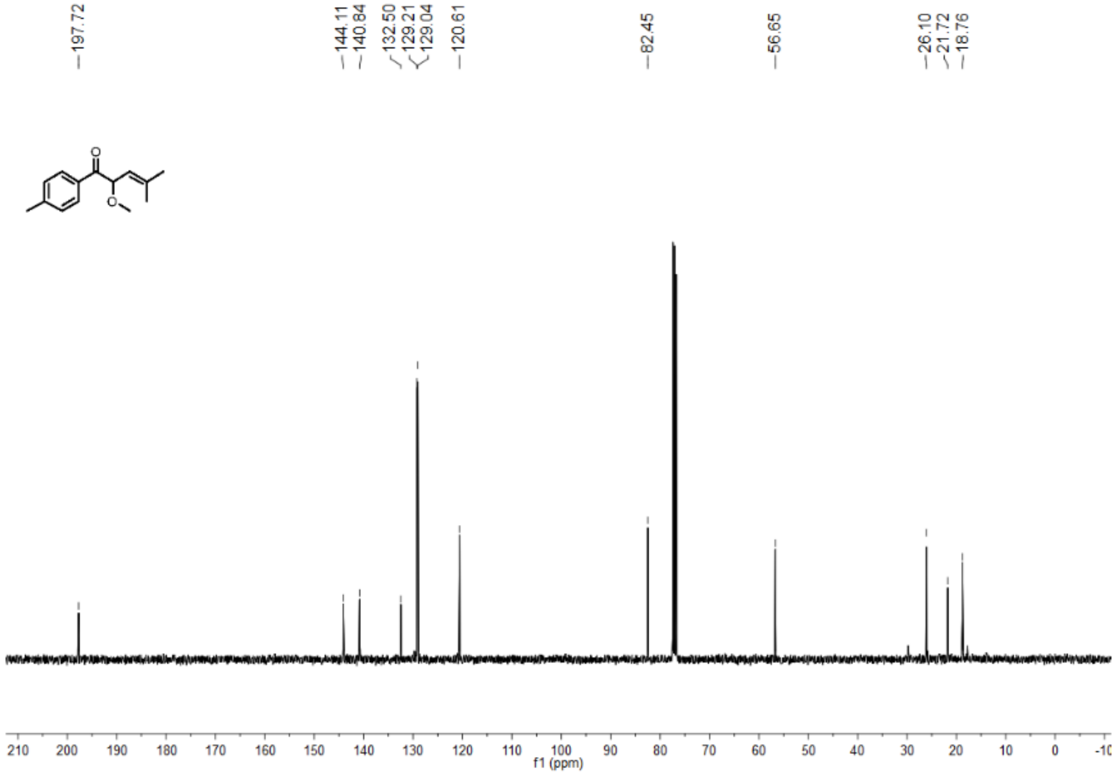

Supplementary Figure 88 ^{13}C NMR (100 MHz) spectrum of compound **39** in $CDCl_3$

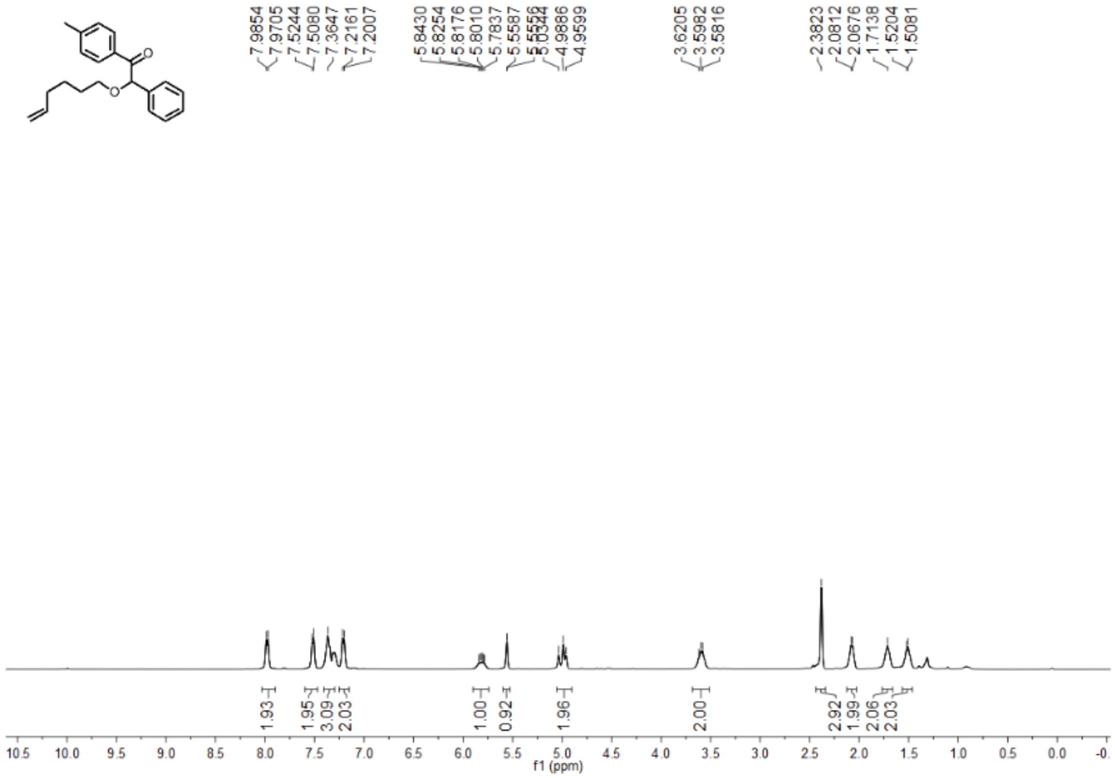

Supplementary Figure 89 ^{1}H NMR (400 MHz) spectrum of compound **40** in $CDCl_3$

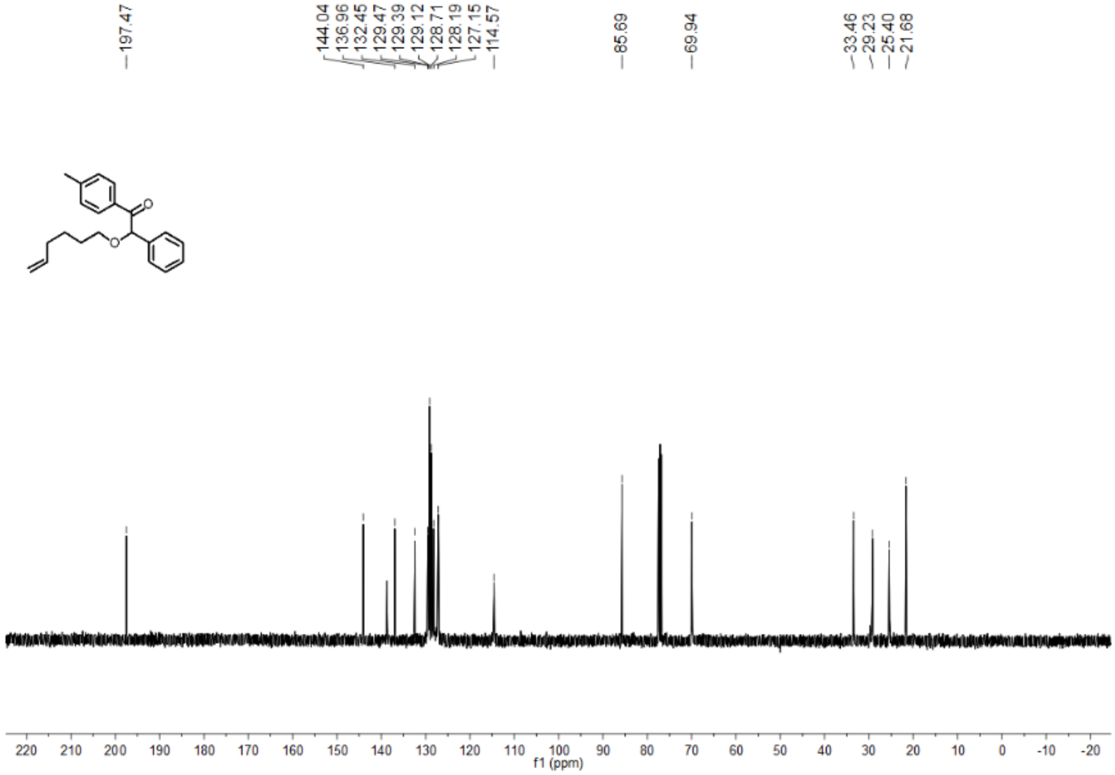

Supplementary Figure 90 ^{13}C NMR (100 MHz) spectrum of compound **40** in $CDCl_3$

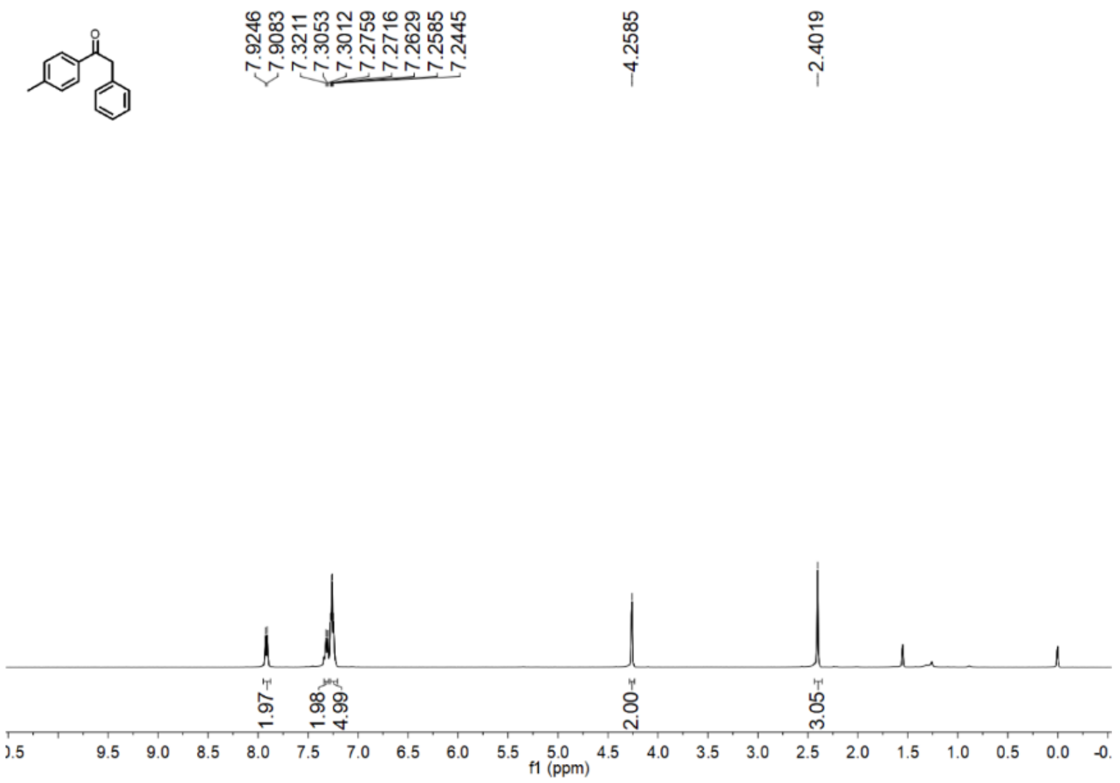

Supplementary Figure 91 ^{1}H NMR (400 MHz) spectrum of compound **41** in $CDCl_3$

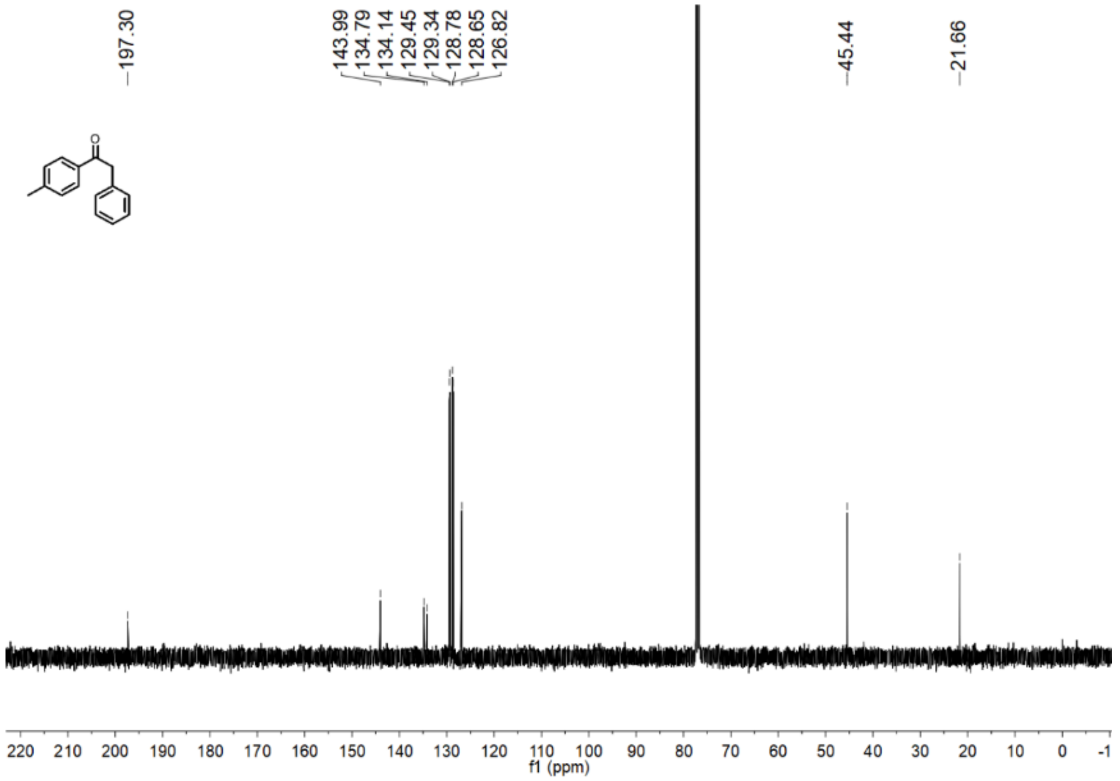

Supplementary Figure 92 ^{13}C NMR (100 MHz) spectrum of compound **41** in $CDCl_3$

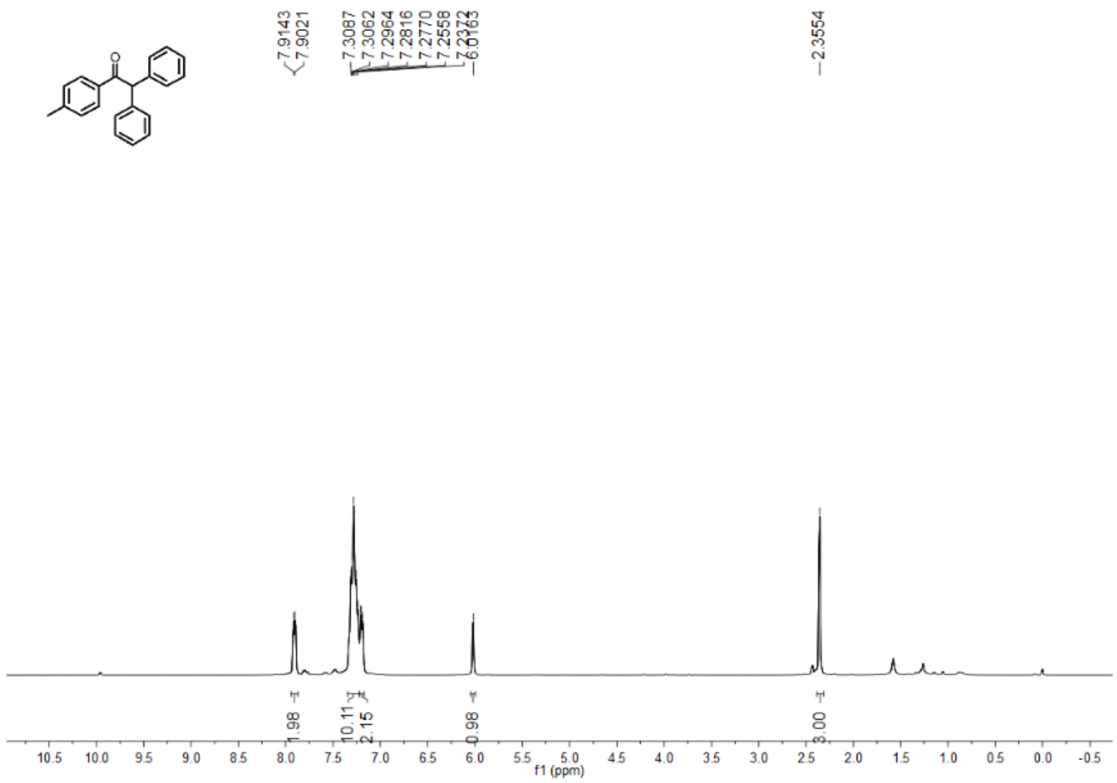

Supplementary Figure 93 ^{1}H NMR (400 MHz) spectrum of compound **42** in $CDCl_3$

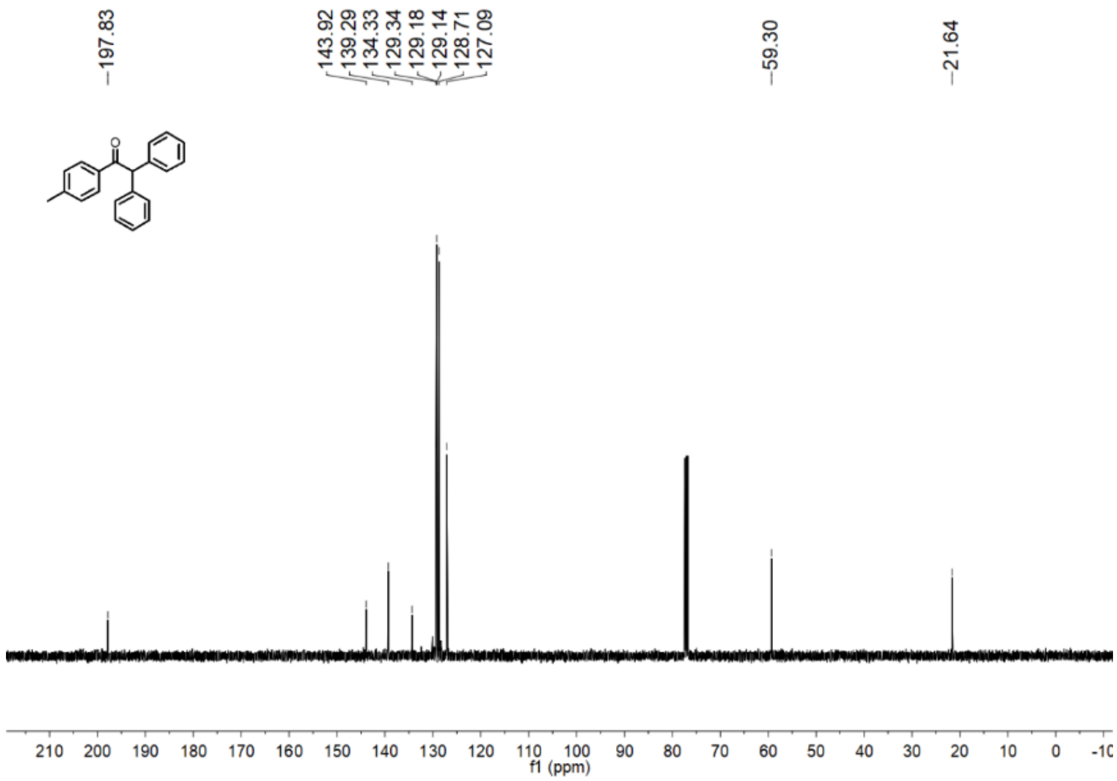

Supplementary Figure 94 ^{13}C NMR (100 MHz) spectrum of compound **42** in $CDCl_3$

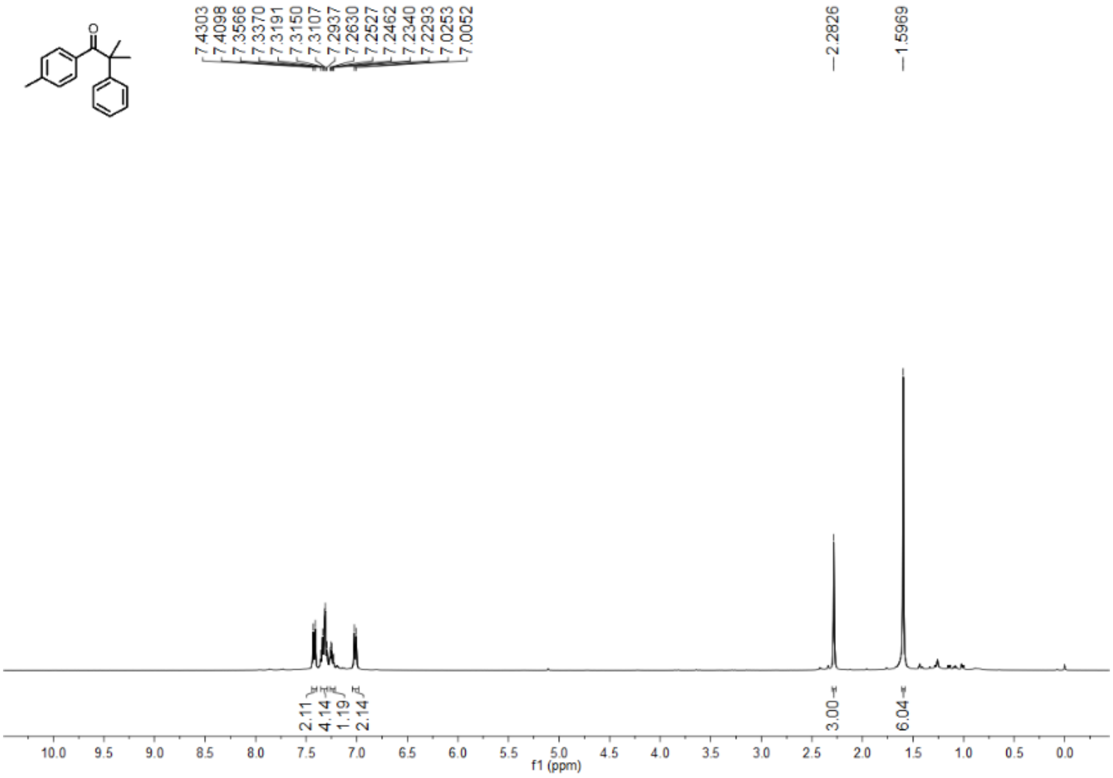

Supplementary Figure 95 ^{1}H NMR (400 MHz) spectrum of compound **43** in $CDCl_3$

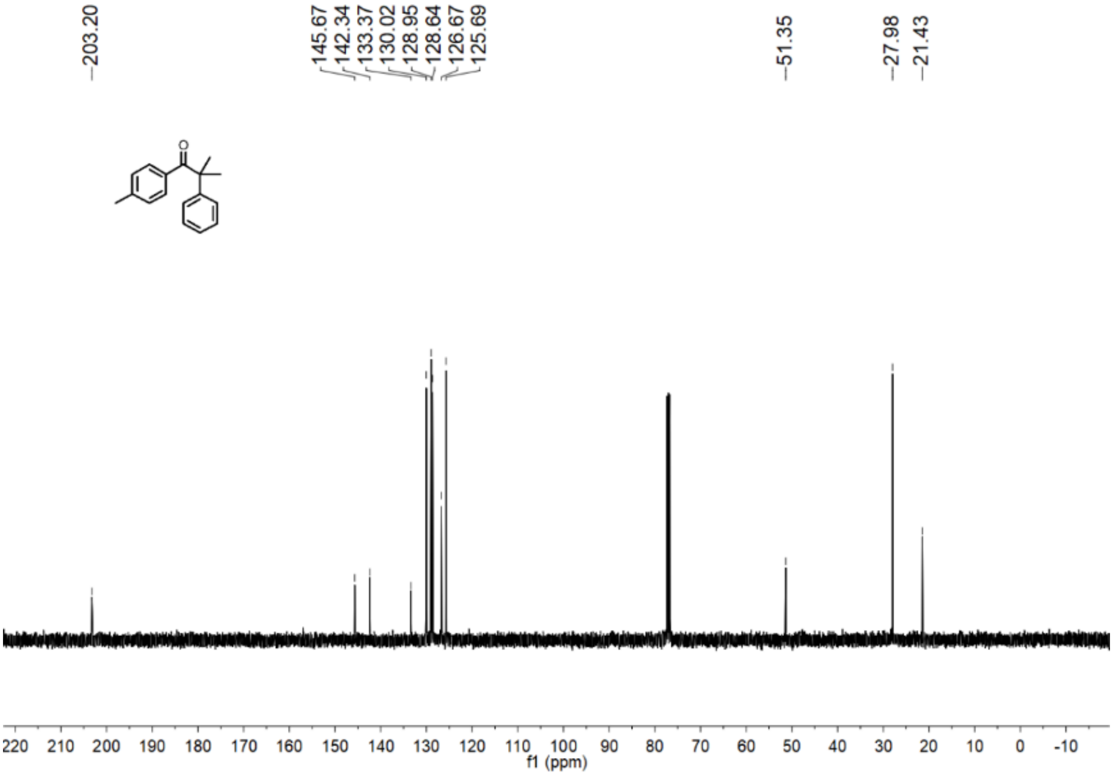

Supplementary Figure 96 ^{13}C NMR (100 MHz) spectrum of compound **43** in $CDCl_3$

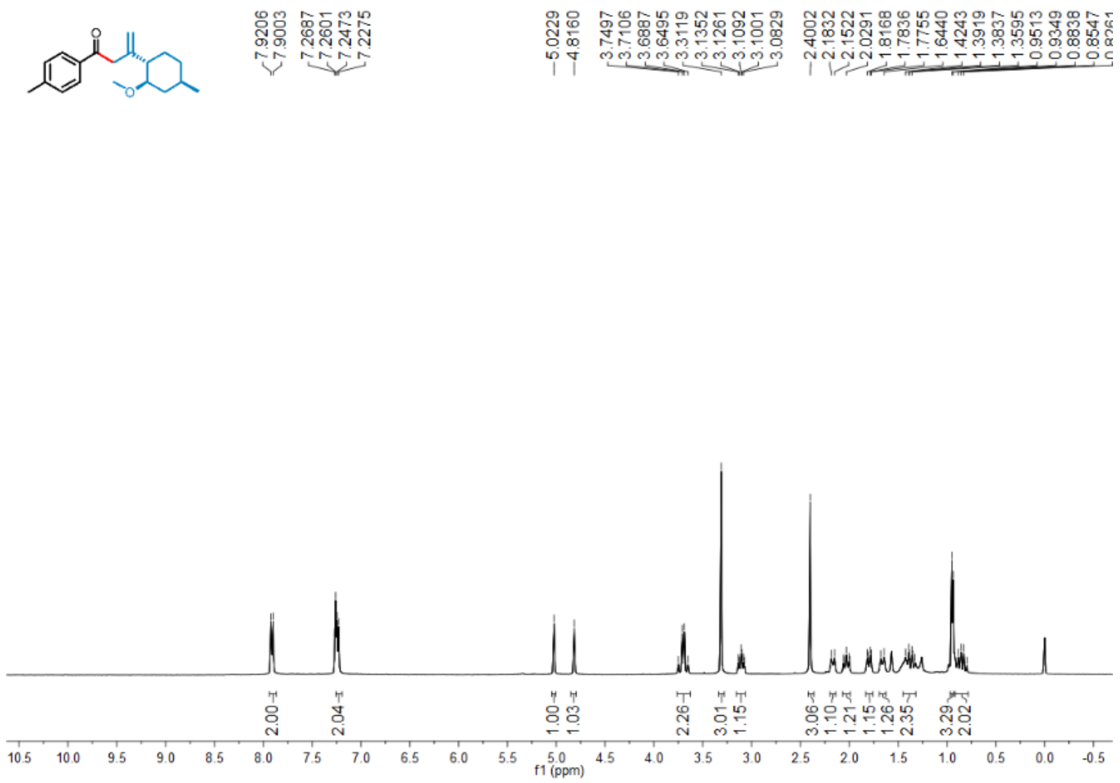

Supplementary Figure 97 ^{1}H NMR (400 MHz) spectrum of compound **44** in $CDCl_3$

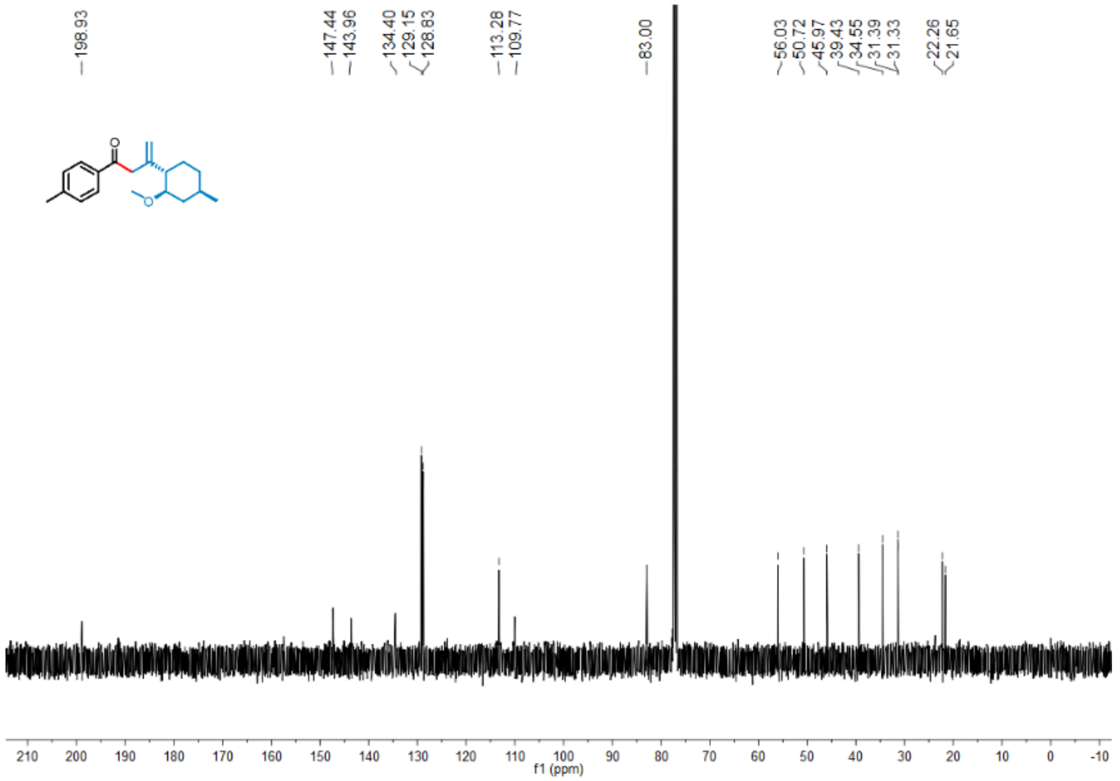

Supplementary Figure 98 ^{13}C NMR (100 MHz) spectrum of compound **44** in $CDCl_3$

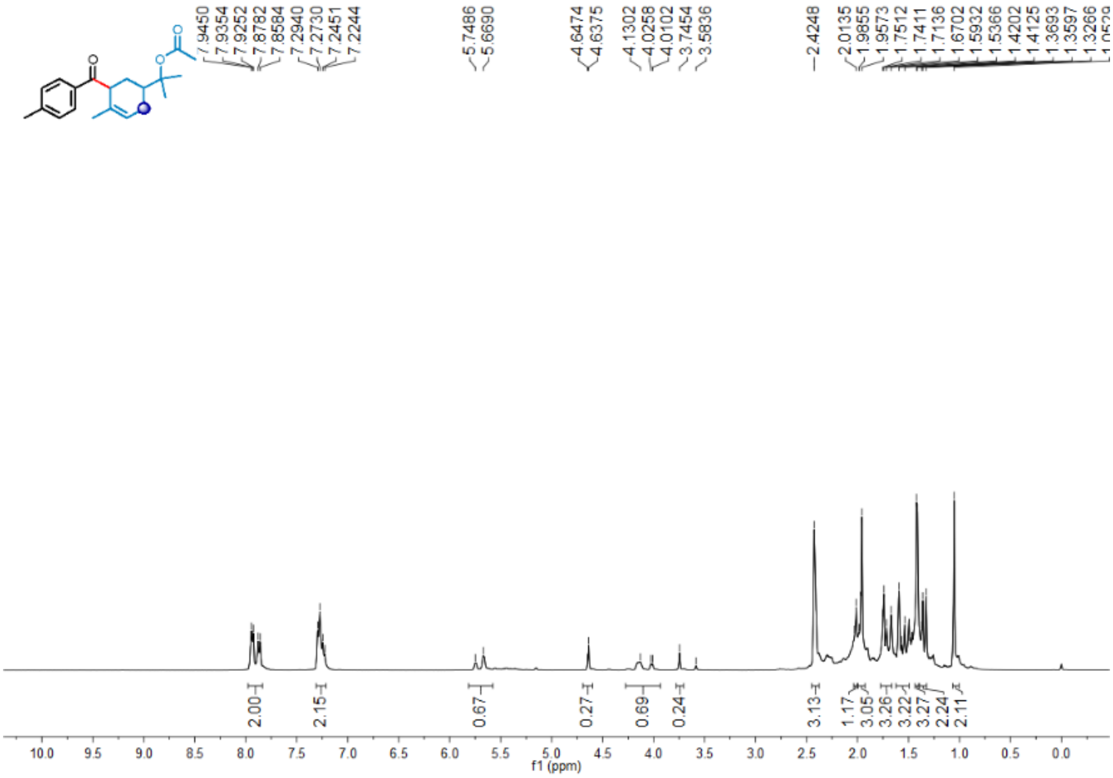

Supplementary Figure 99 ^{1}H NMR (400 MHz) spectrum of compound **45** in $CDCl_3$

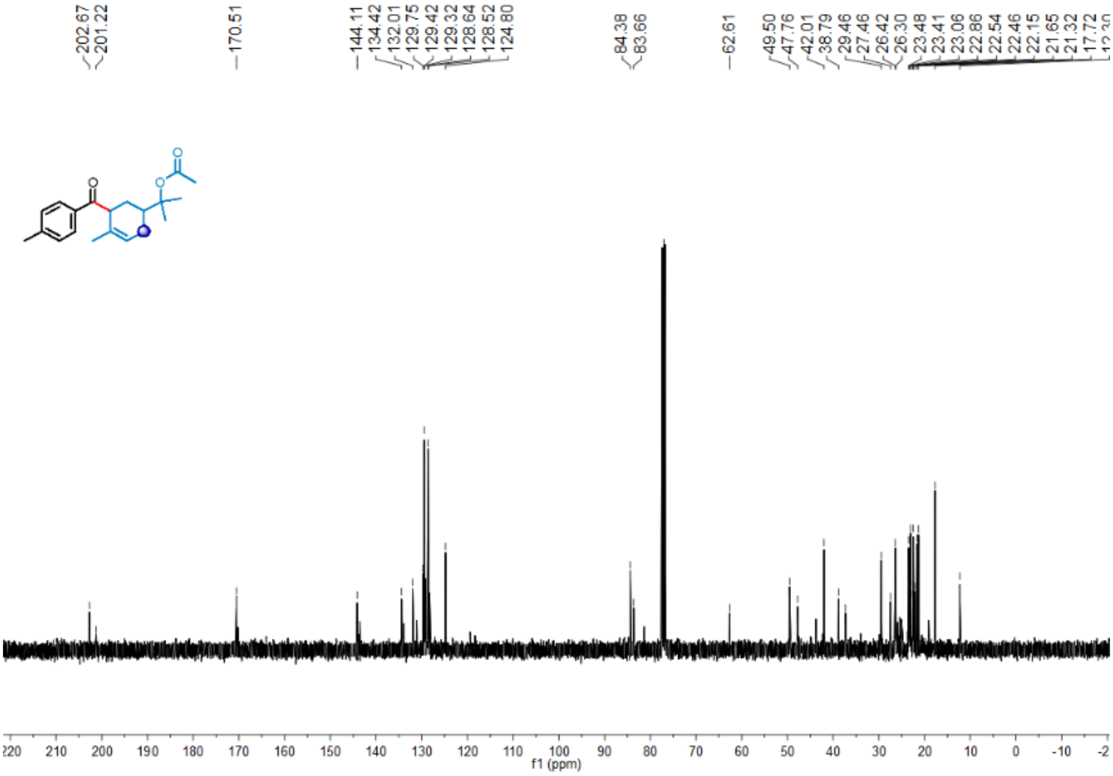

Supplementary Figure 100 ^{13}C NMR (100 MHz) spectrum of compound **45** in $CDCl_3$

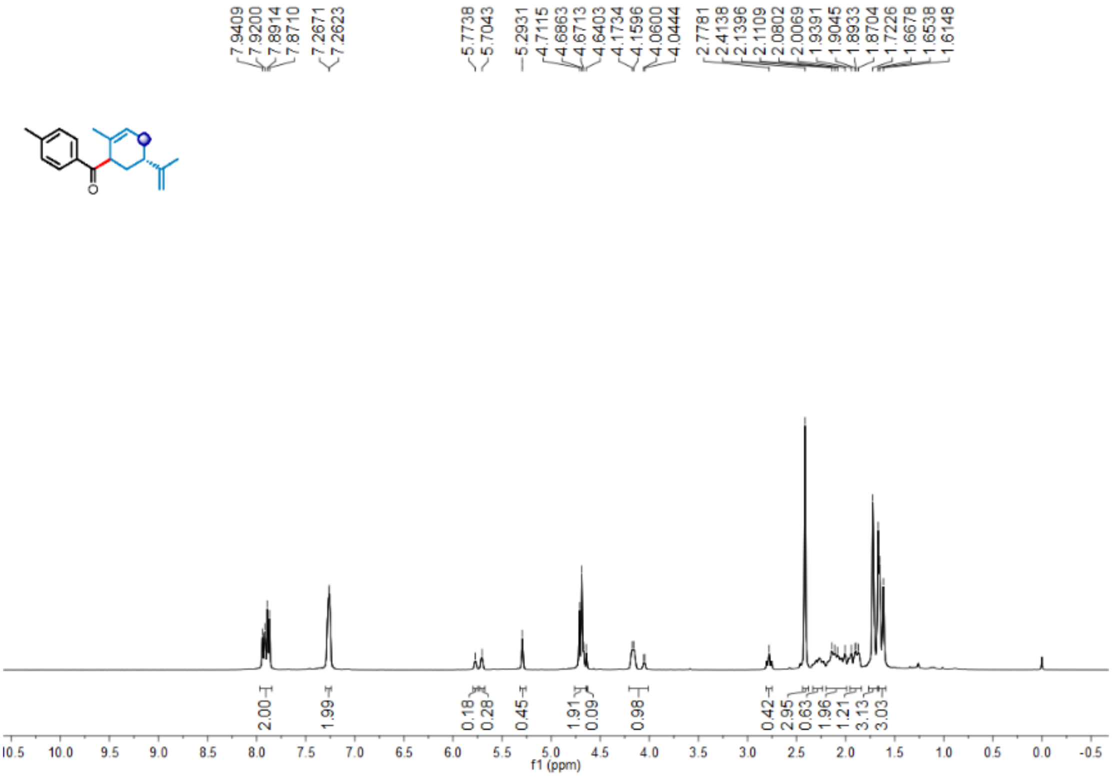

Supplementary Figure 101 ^{1}H NMR (400 MHz) spectrum of compound **46** in $CDCl_3$

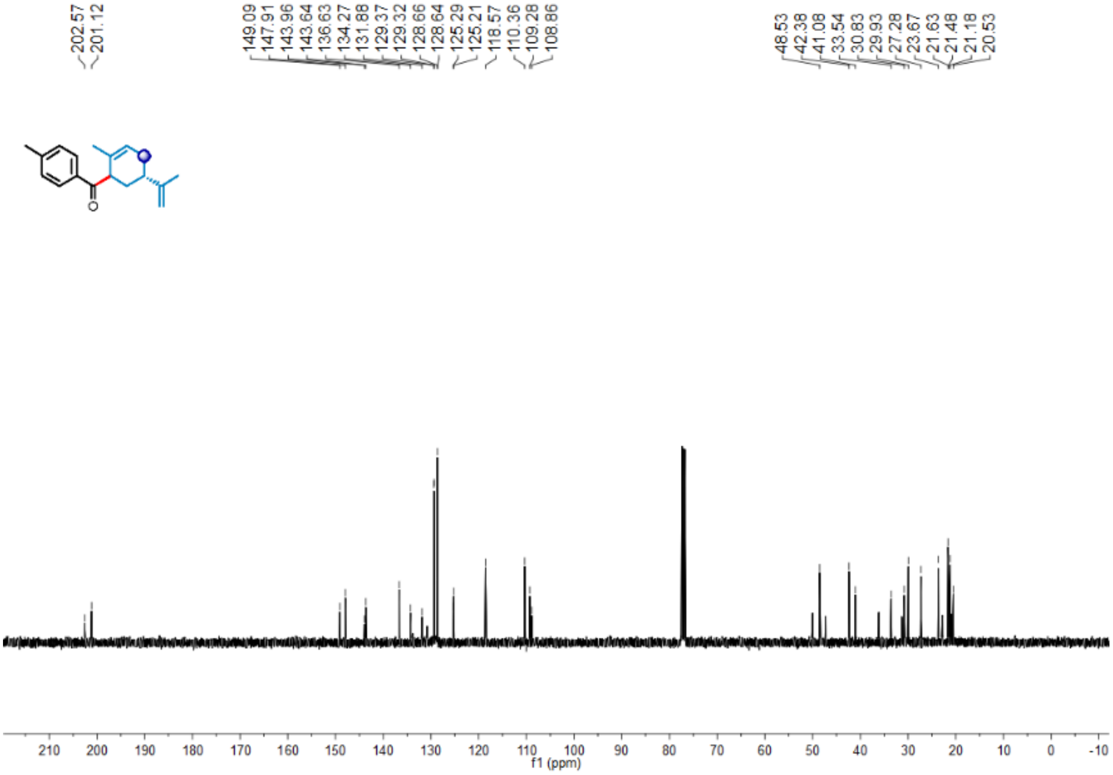

Supplementary Figure 102 ^{13}C NMR (100 MHz) spectrum of compound **46** in $CDCl_3$

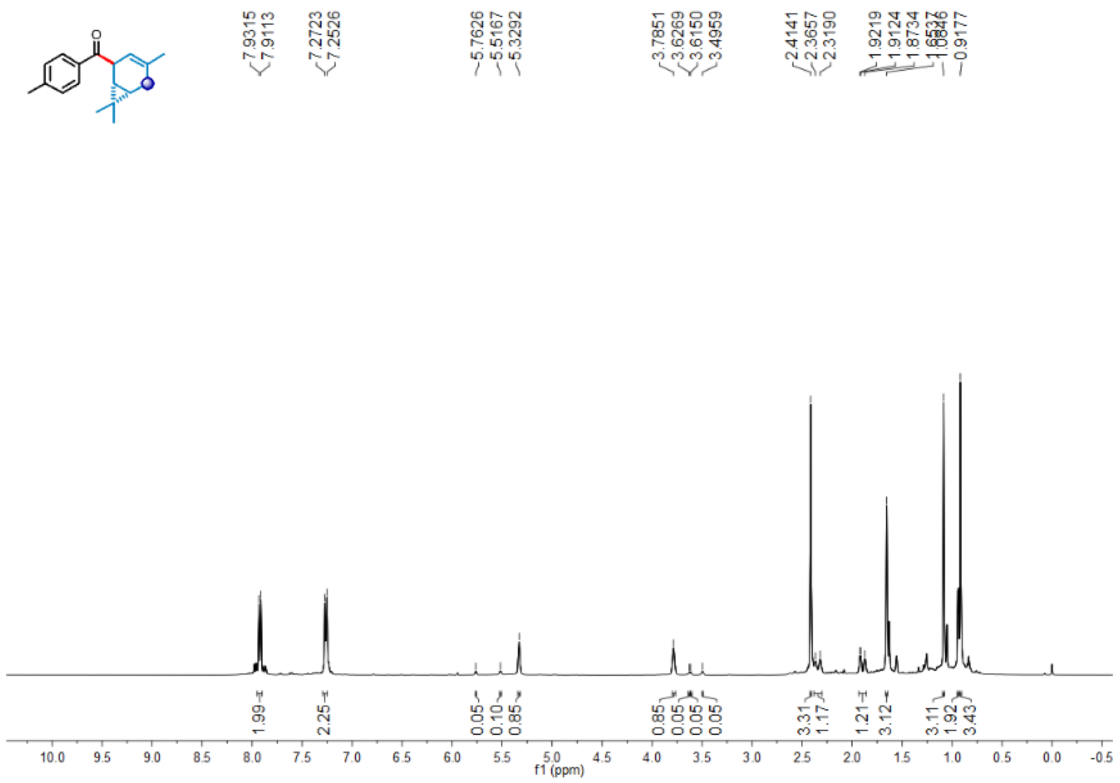

Supplementary Figure 103 ^{1}H NMR (400 MHz) spectrum of compound **47** in $CDCl_3$

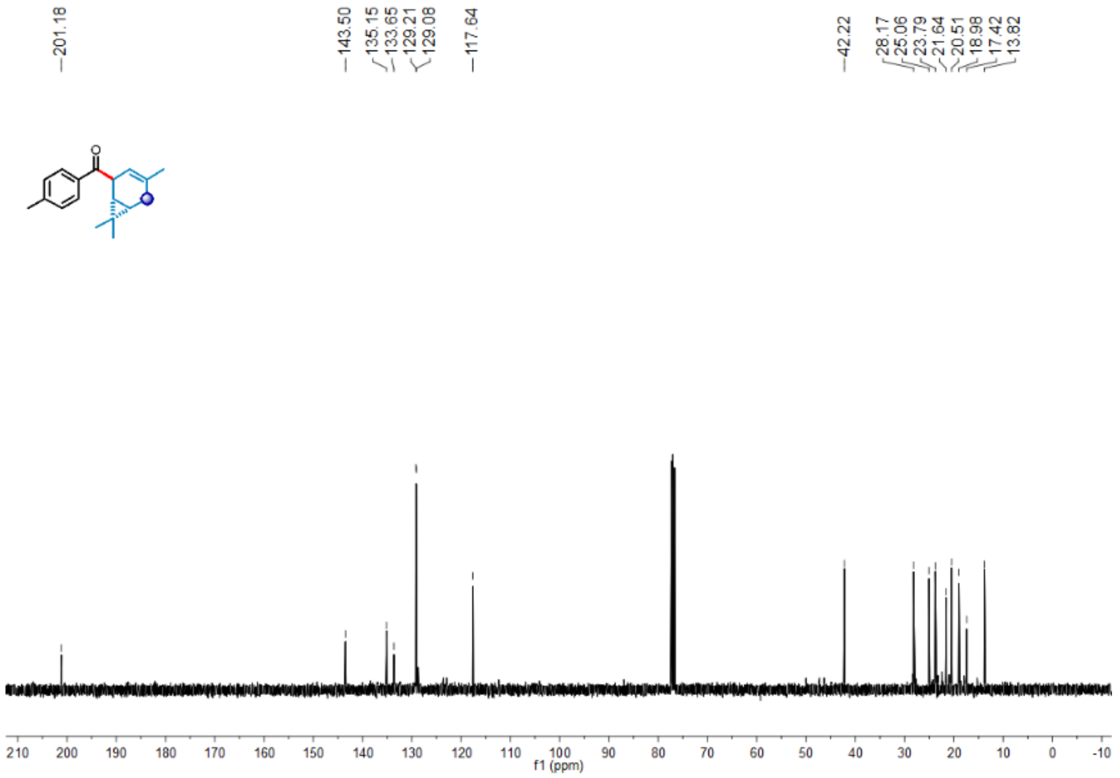

Supplementary Figure 104 ^{13}C NMR (100 MHz) spectrum of compound **47** in $CDCl_3$

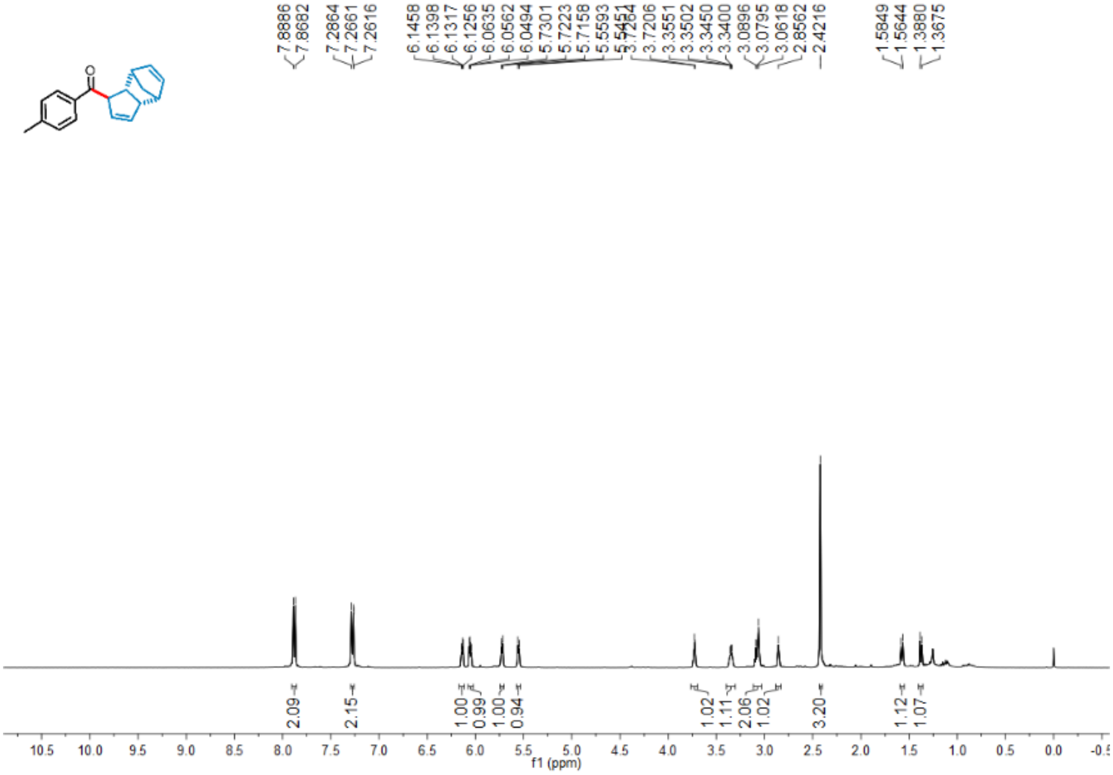

Supplementary Figure 105 ^{1}H NMR (400 MHz) spectrum of compound **48** in $CDCl_3$

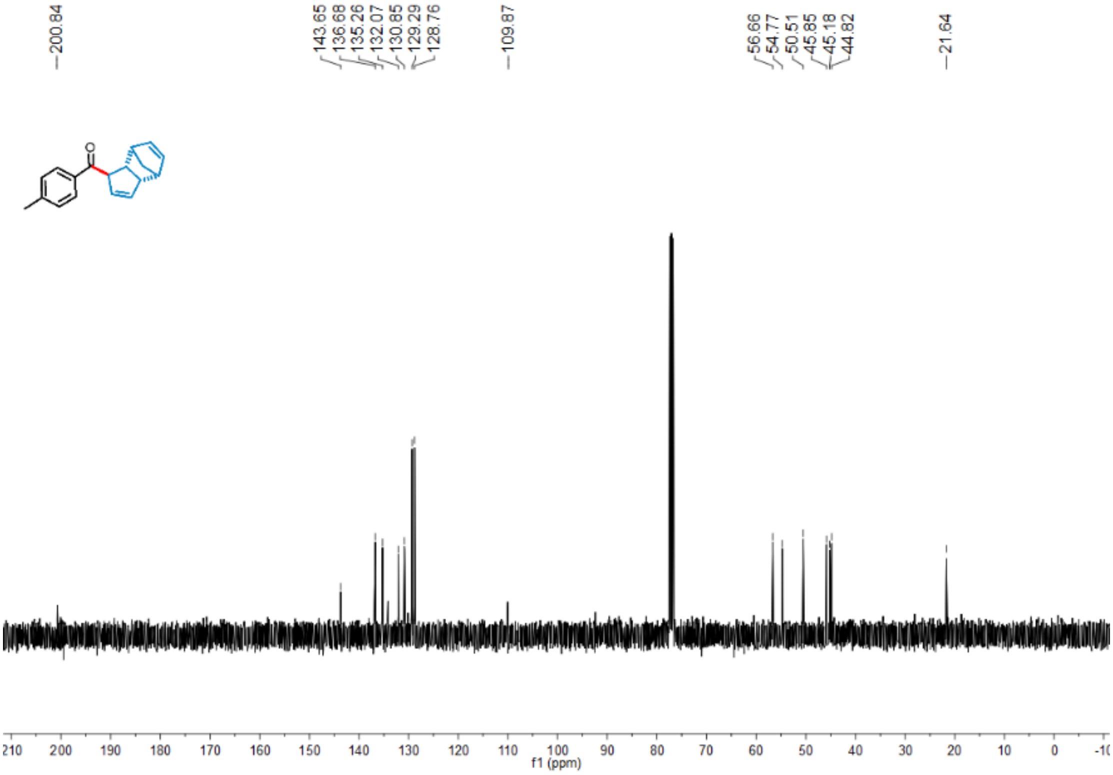

Supplementary Figure 106 ^{13}C NMR (100 MHz) spectrum of compound **48** in $CDCl_3$

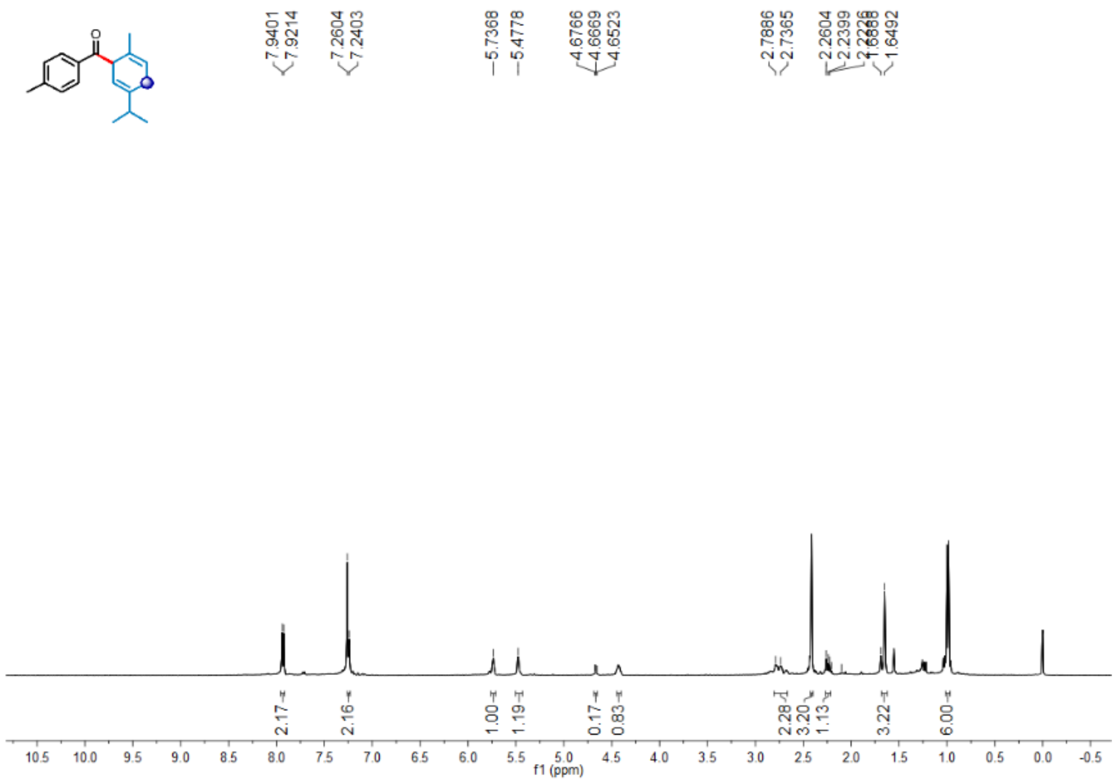

Supplementary Figure 107 ^{1}H NMR (400 MHz) spectrum of compound **49** in $CDCl_3$

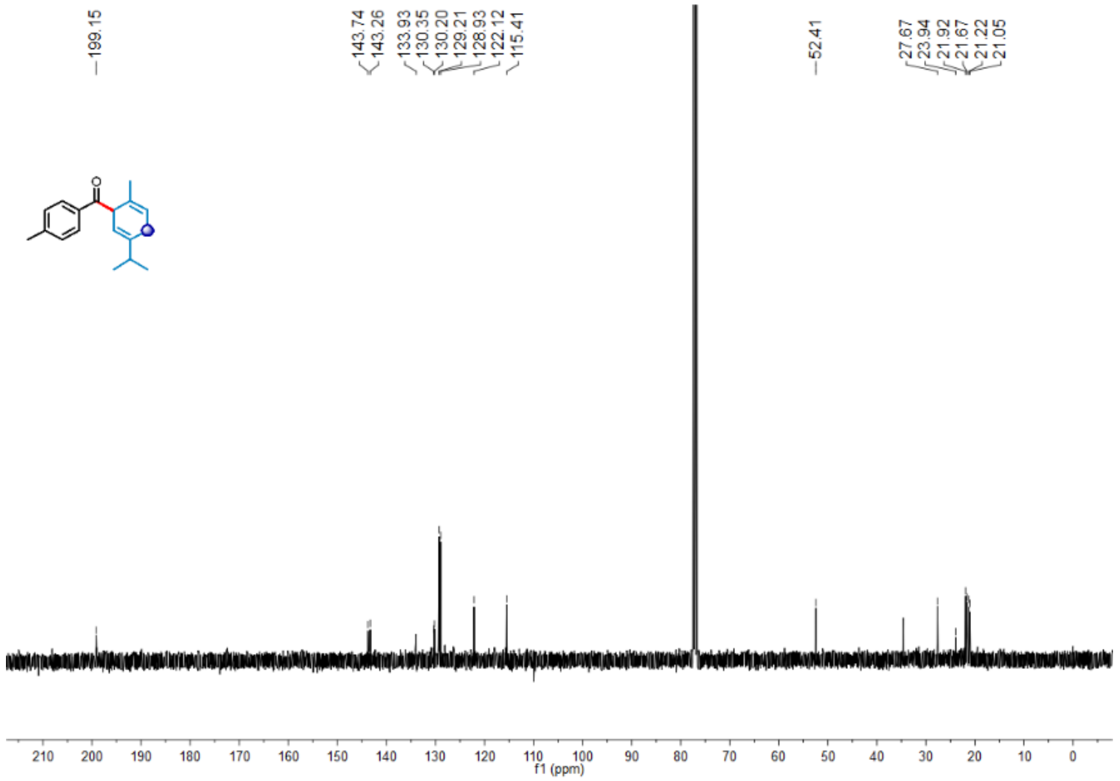

Supplementary Figure 108 ^{13}C NMR (100 MHz) spectrum of compound **49** in $CDCl_3$

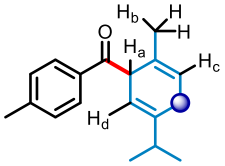

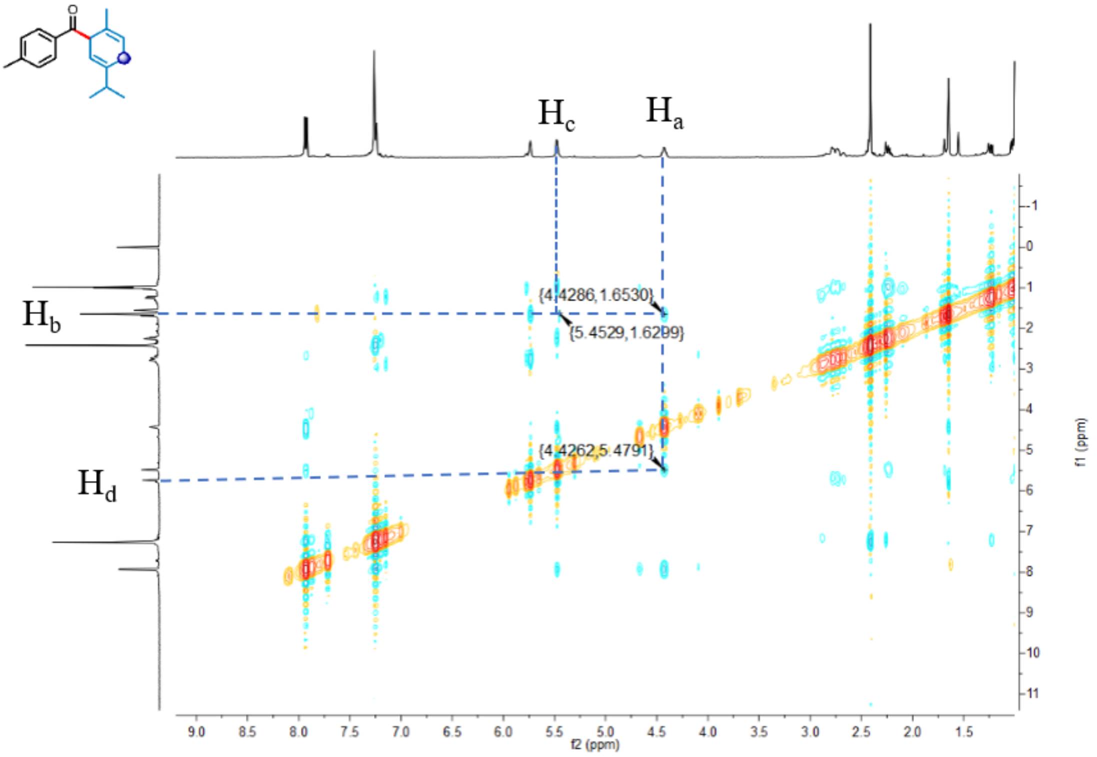

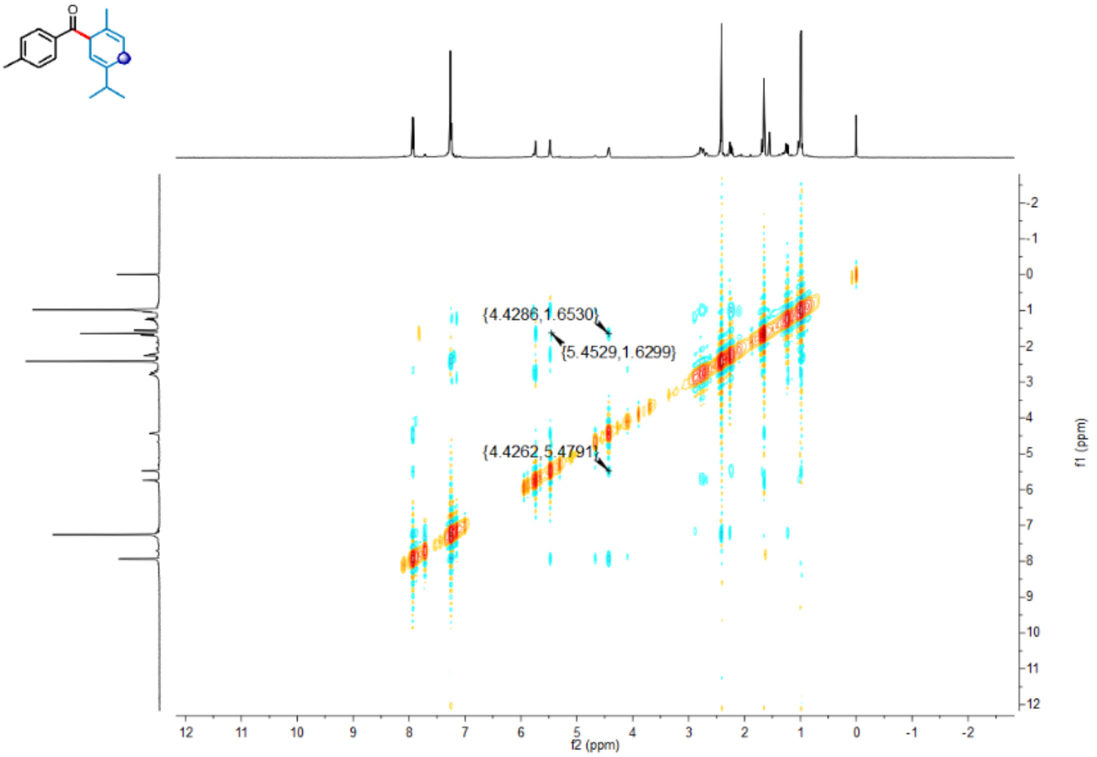

Supplementary Figure 109 NOESY spectrum of compound **49** in $CDCl_3$

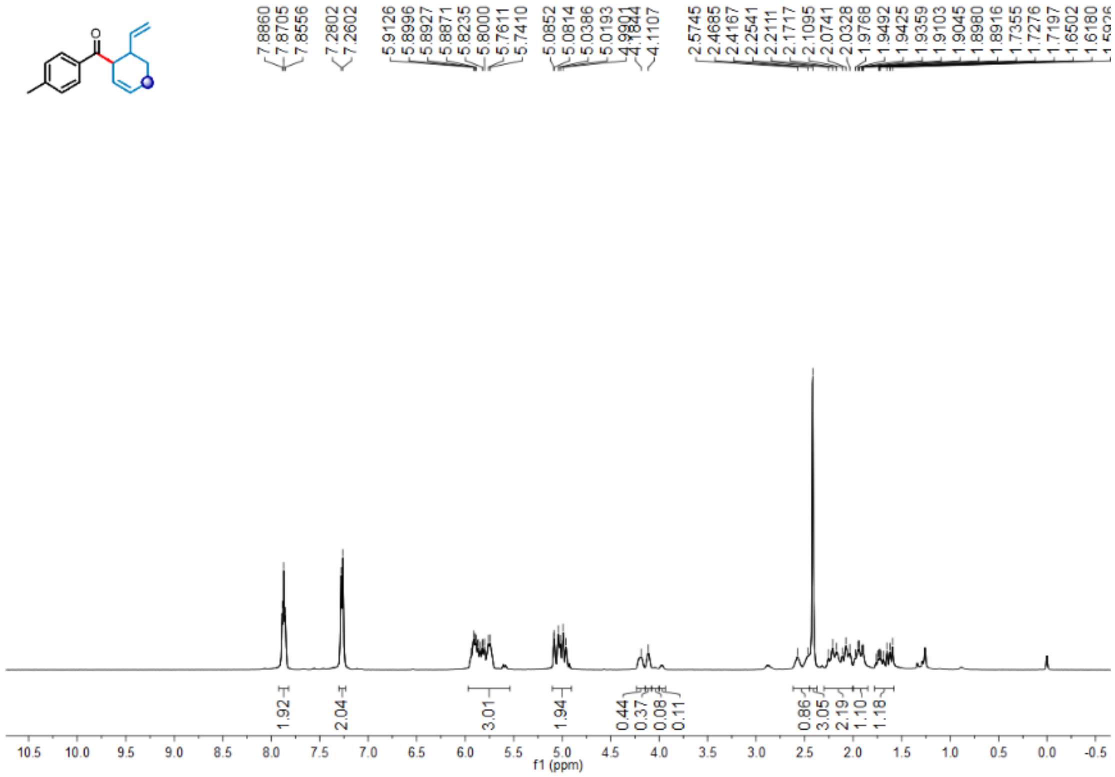

Supplementary Figure 110 ^{1}H NMR (400 MHz) spectrum of compound **50** in $CDCl_3$

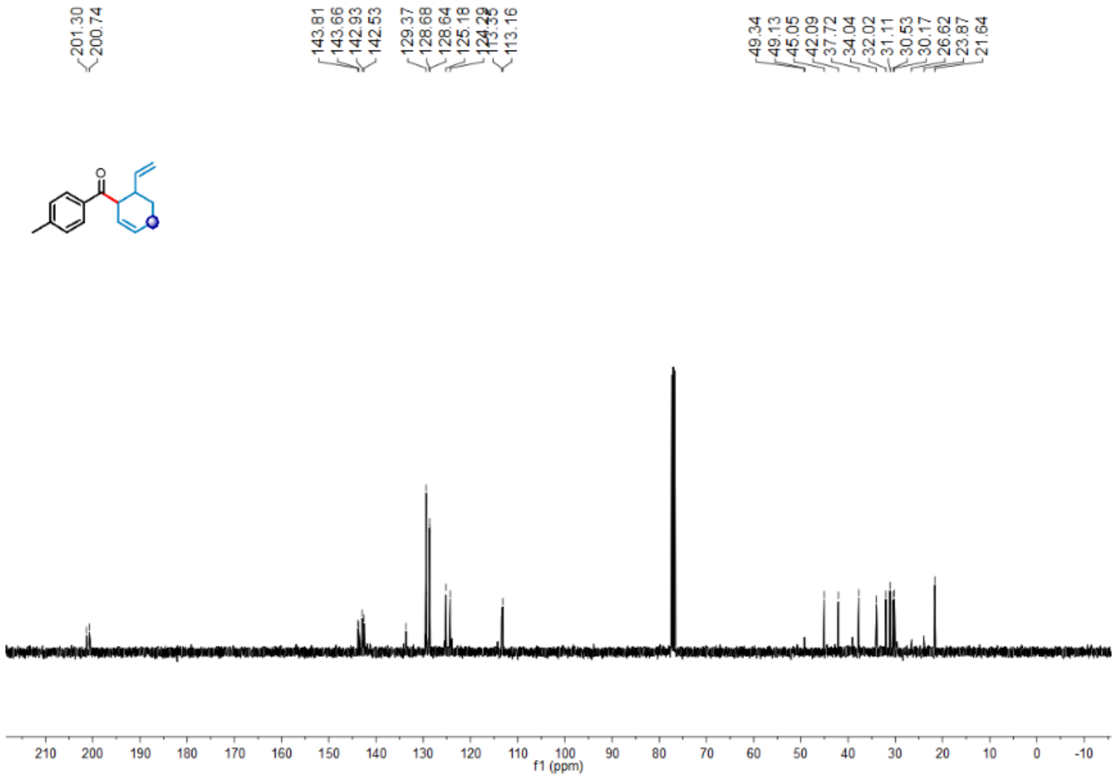

Supplementary Figure 111 ^{13}C NMR (100 MHz) spectrum of compound **50** in $CDCl_3$

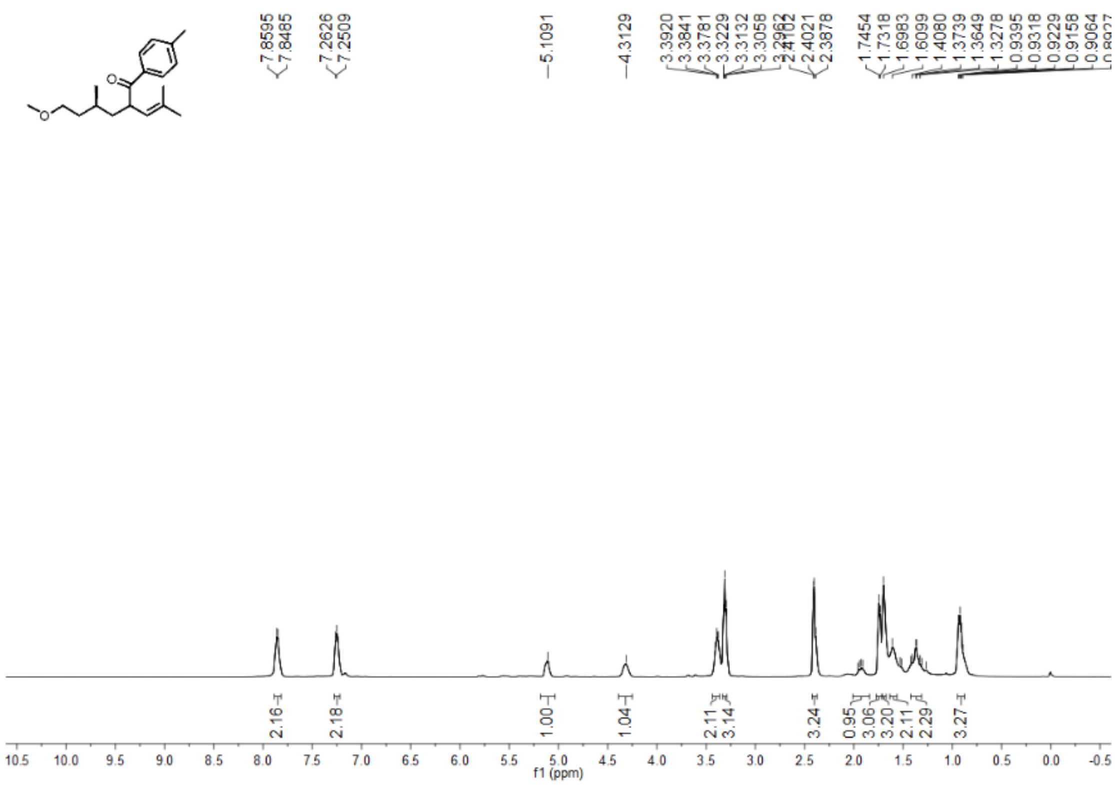

Supplementary Figure 112 ^{1}H NMR (400 MHz) spectrum of compound **51** in $CDCl_3$

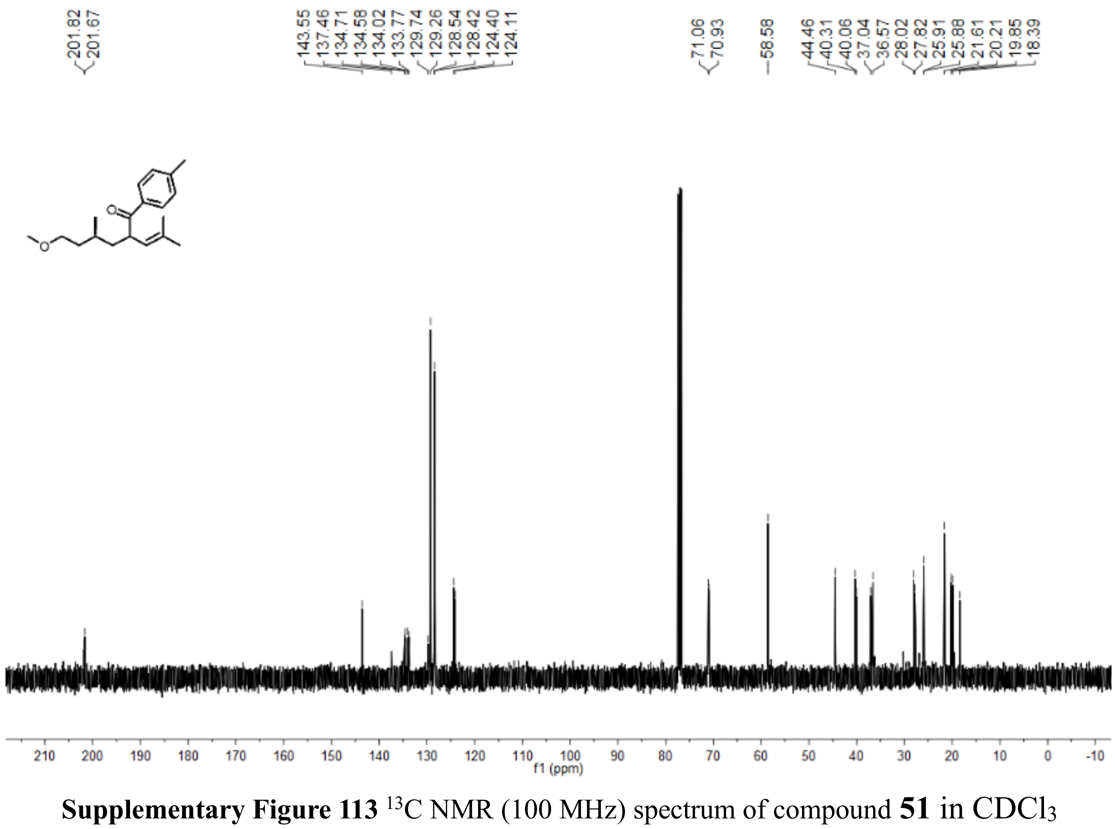

Supplementary Figure 113 ^{13}C NMR (100 MHz) spectrum of compound **51** in $CDCl_3$

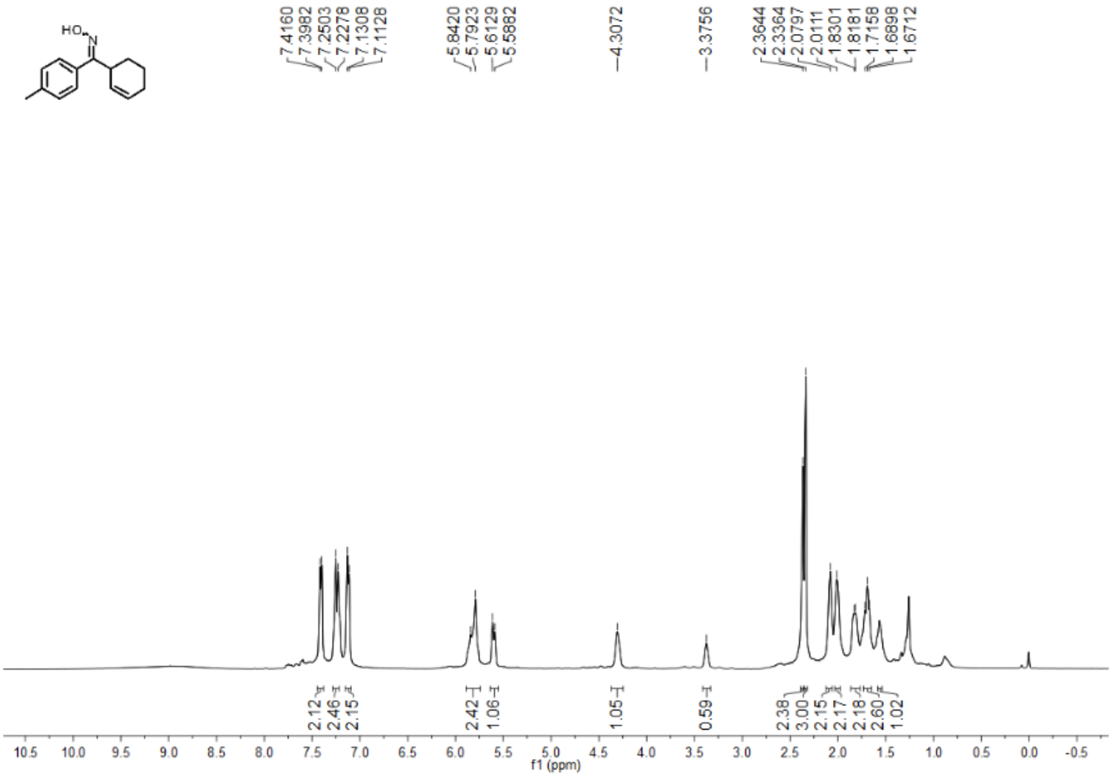

Supplementary Figure 114 ^{1}H NMR (400 MHz) spectrum of compound **52** in $CDCl_3$

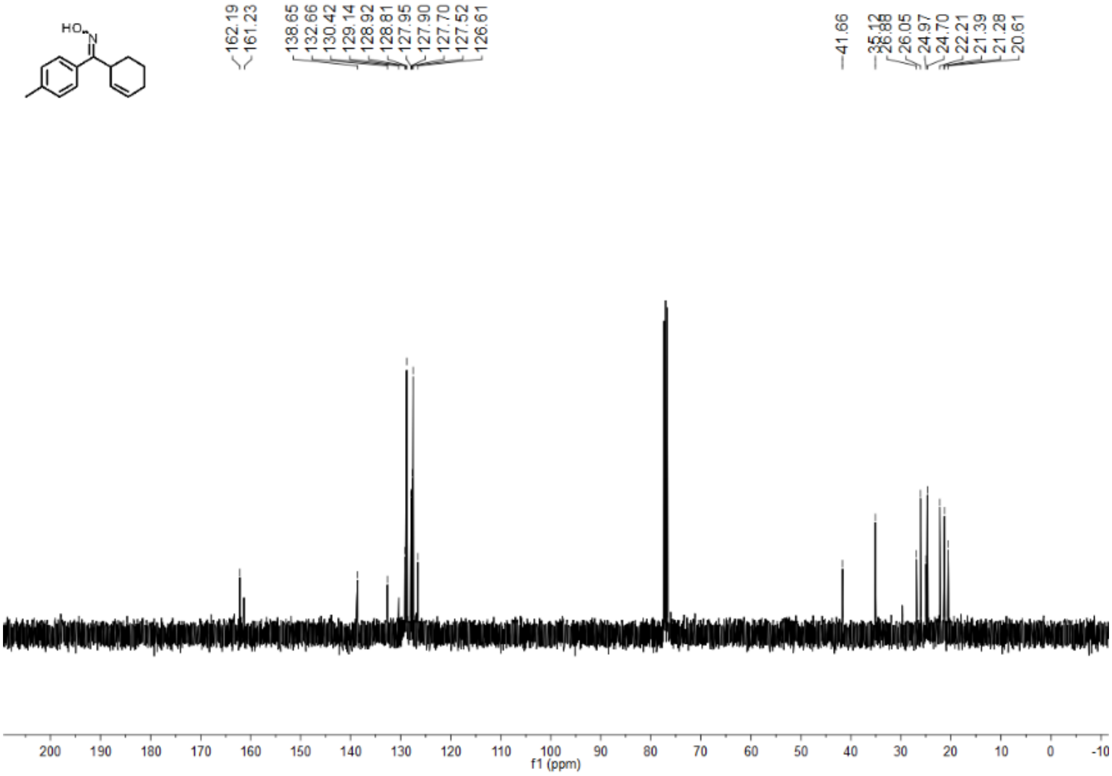

Supplementary Figure 115 ^{13}C NMR (100 MHz) spectrum of compound **52** in $CDCl_3$

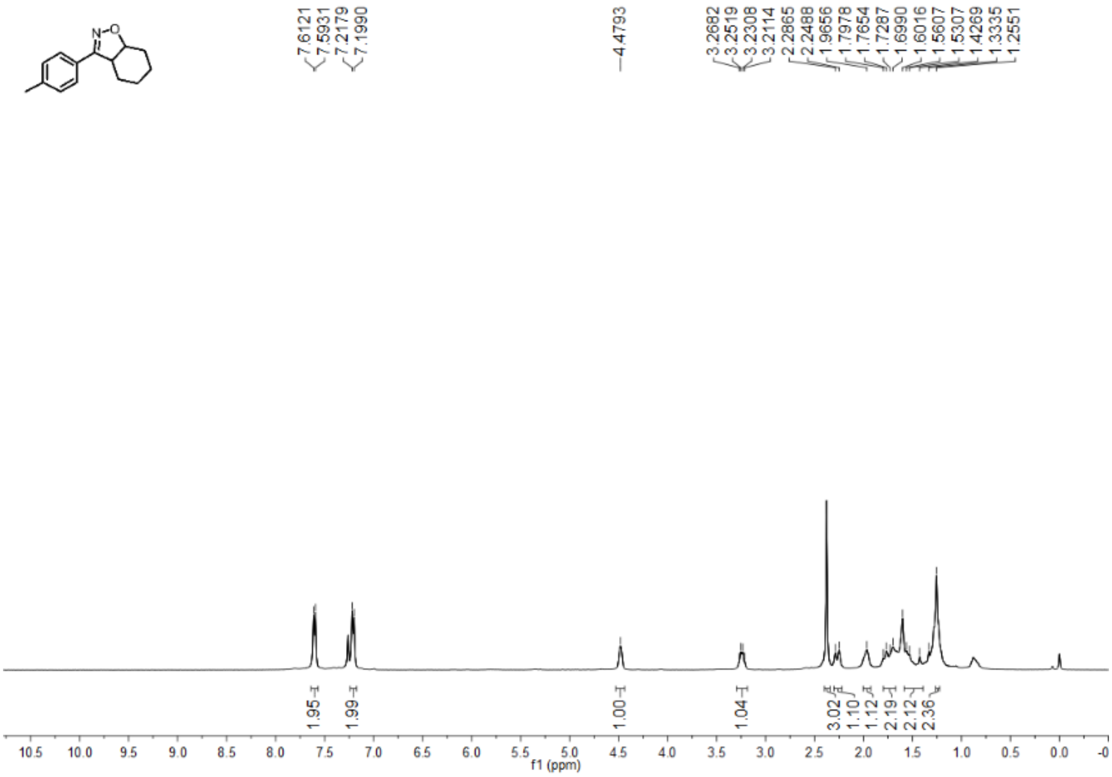

Supplementary Figure 116 ^{1}H NMR (400 MHz) spectrum of compound **53** in $CDCl_3$

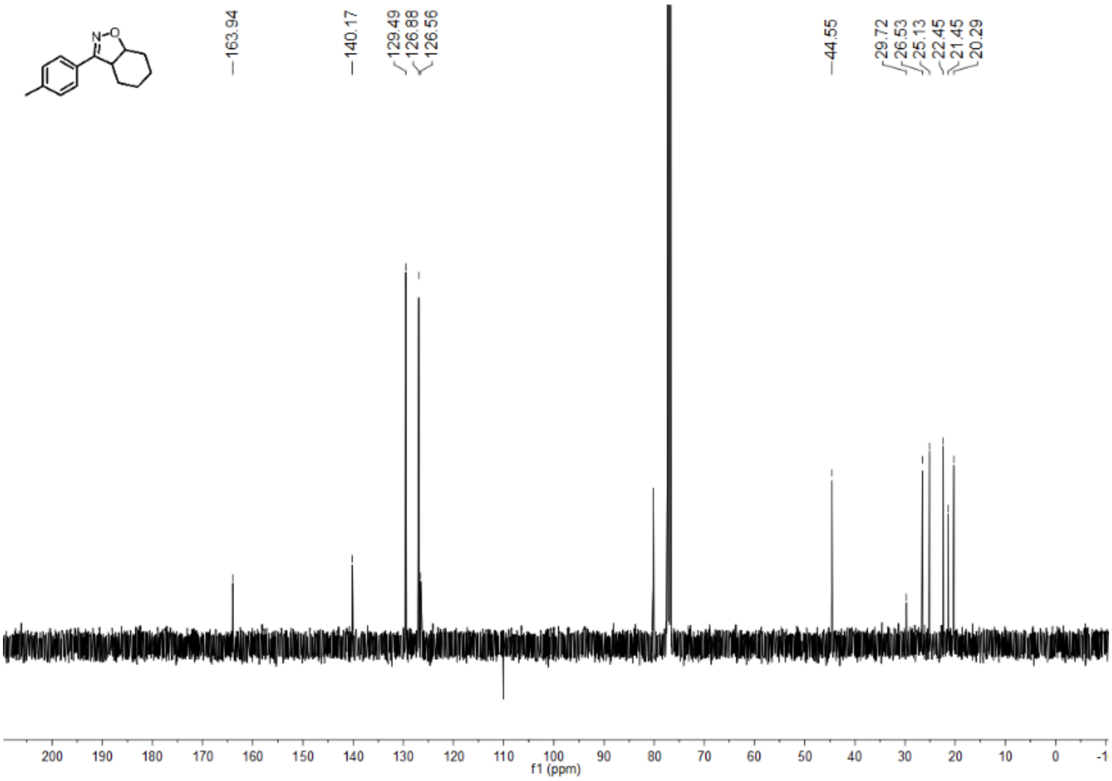

Supplementary Figure 117 ^{13}C NMR (100 MHz) spectrum of compound **53** in $CDCl_3$

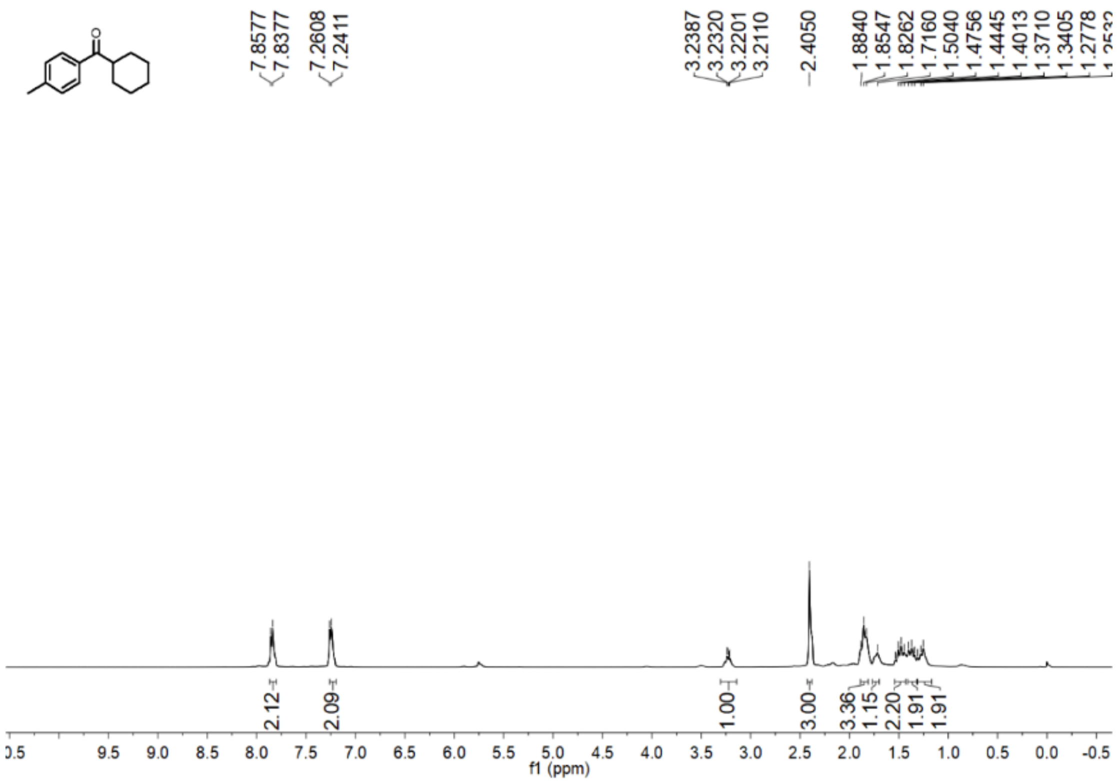

Supplementary Figure 118 ^{1}H NMR (400 MHz) spectrum of compound **54** in $CDCl_3$

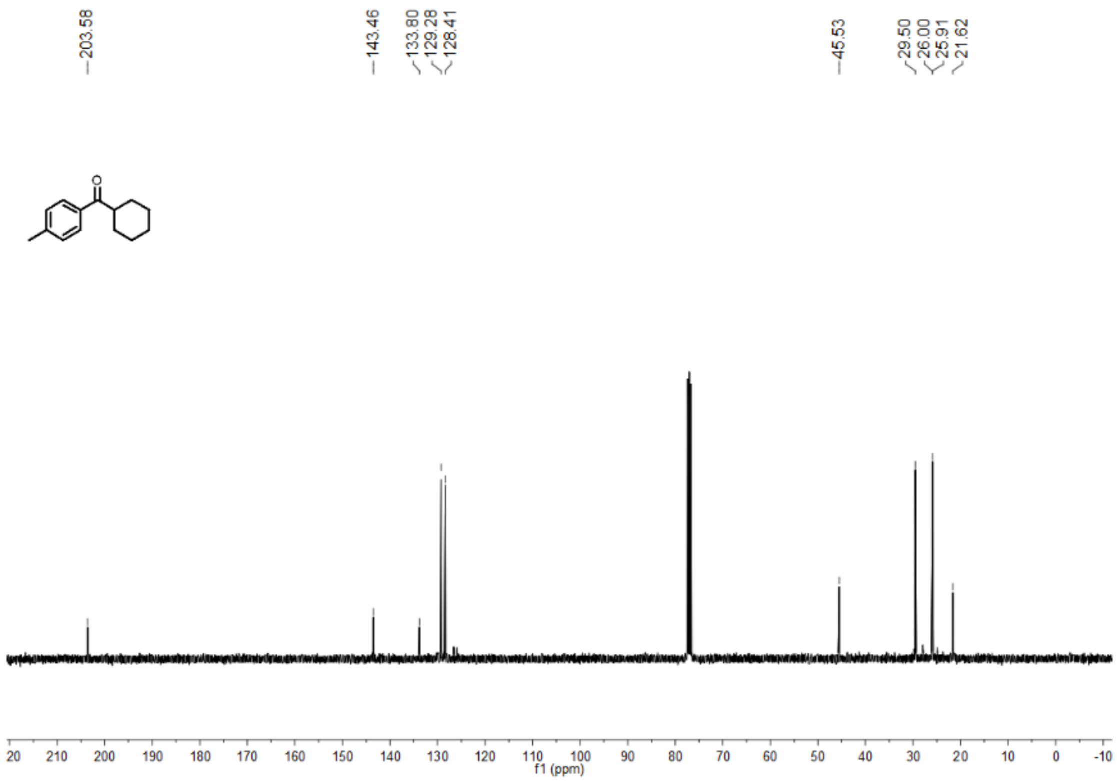

Supplementary Figure 119 ^{13}C NMR (100 MHz) spectrum of compound **54** in $CDCl_3$

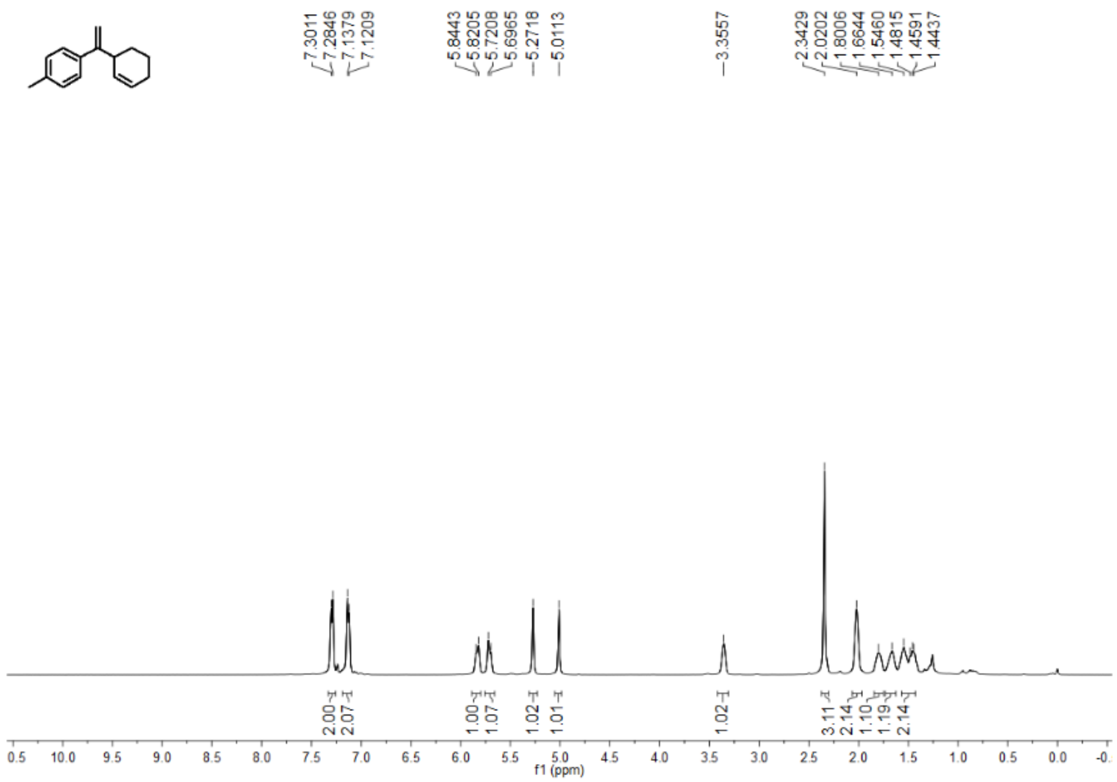

Supplementary Figure 120 ^{1}H NMR (400 MHz) spectrum of compound **55** in $CDCl_3$

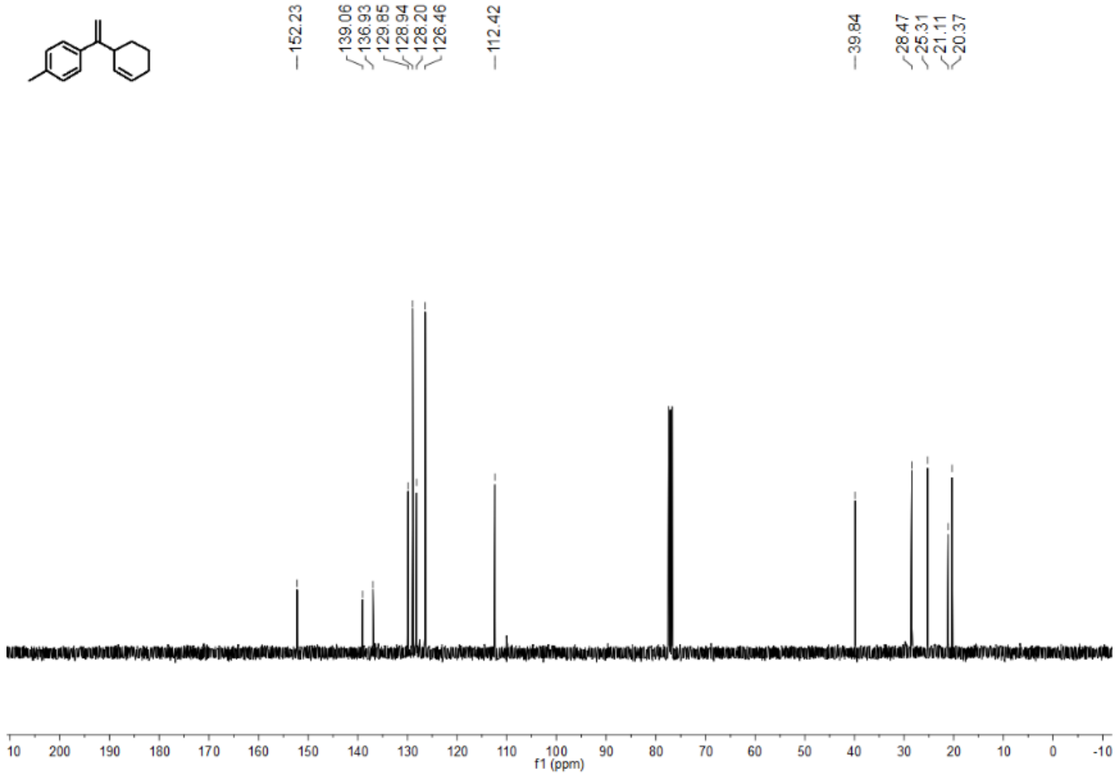

Supplementary Figure 121 ^{13}C NMR (100 MHz) spectrum of compound **55** in $CDCl_3$

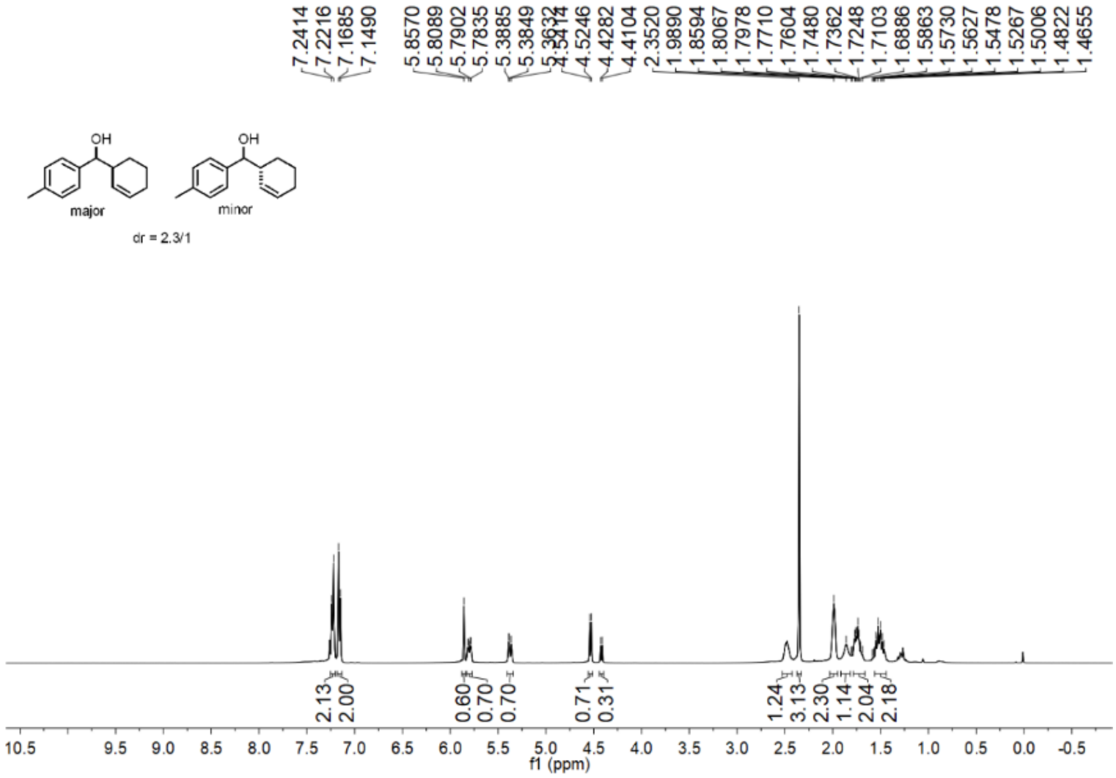

Supplementary Figure 122 ^{1}H NMR (400 MHz) spectrum of compound **56** in $CDCl_3$

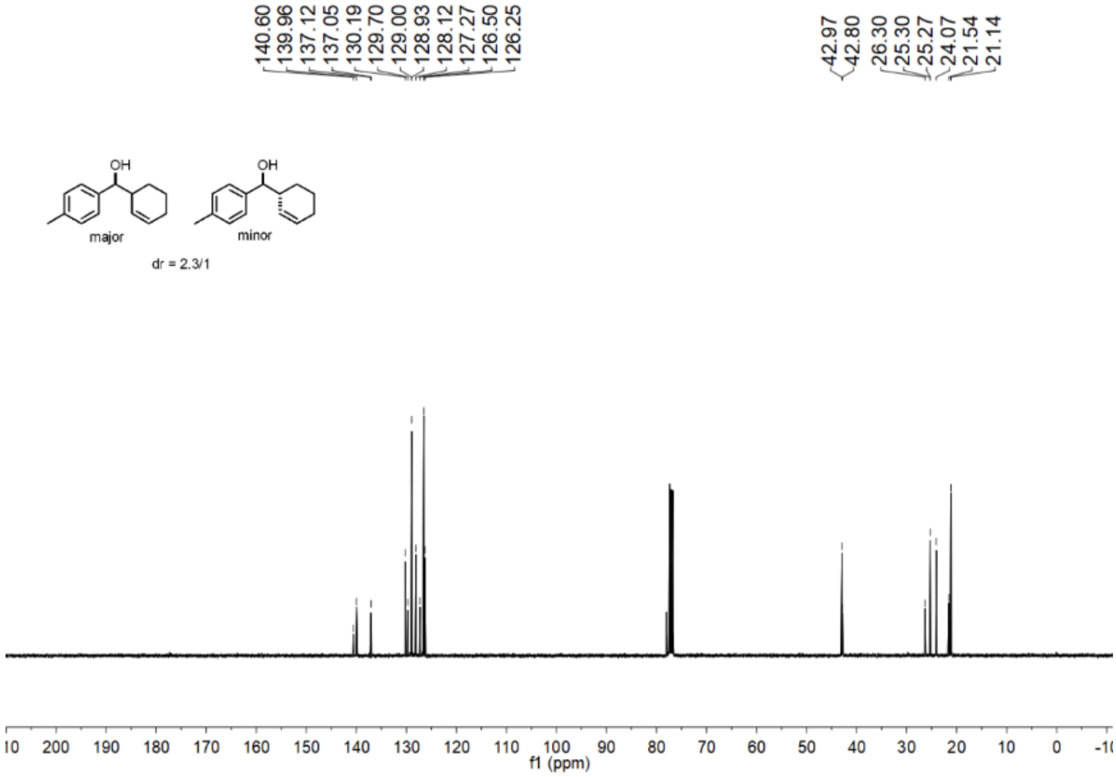

Supplementary Figure 123 ^{13}C NMR (100 MHz) spectrum of compound **56** in $CDCl_3$

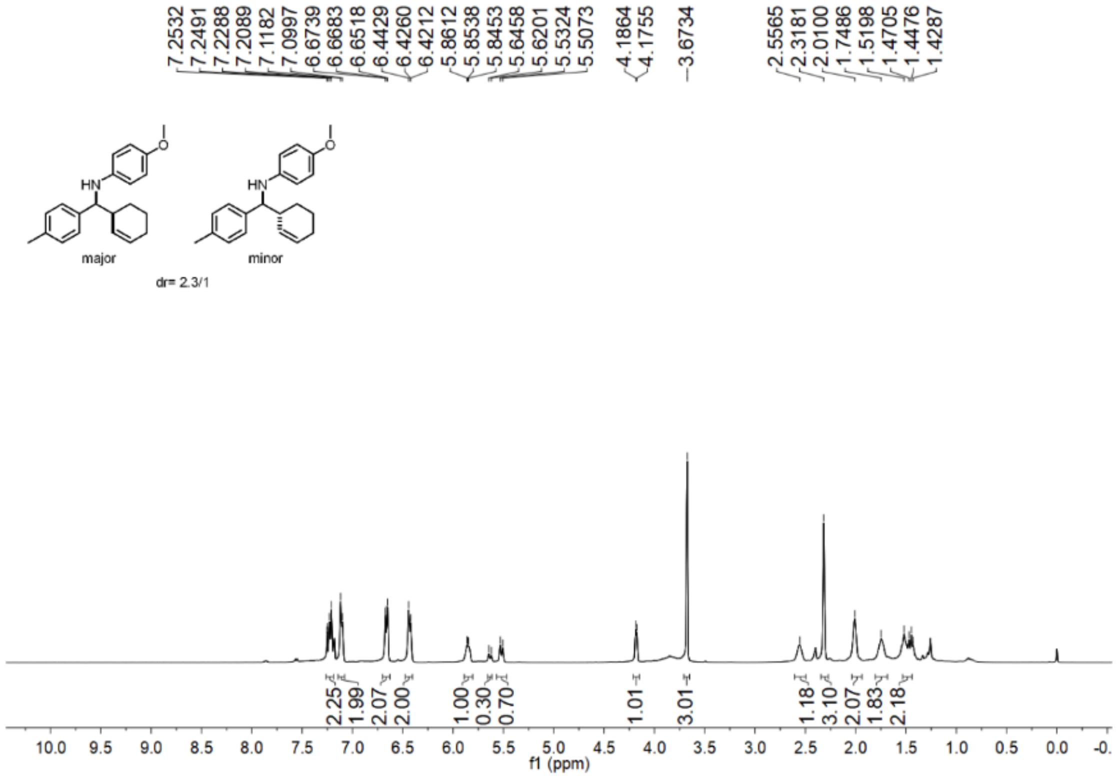

Supplementary Figure 124 ^{1}H NMR (400 MHz) spectrum of compound **57** in $CDCl_3$

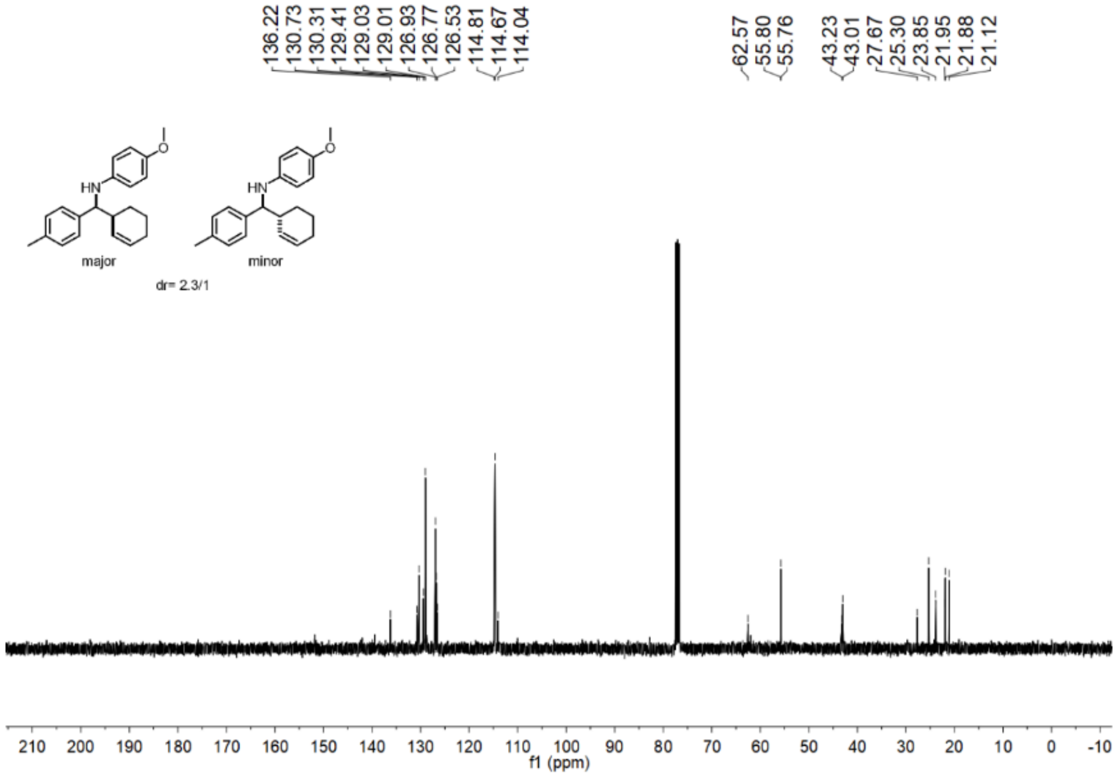

Supplementary Figure 125 ^{13}C NMR (100 MHz) spectrum of compound **57** in $CDCl_3$

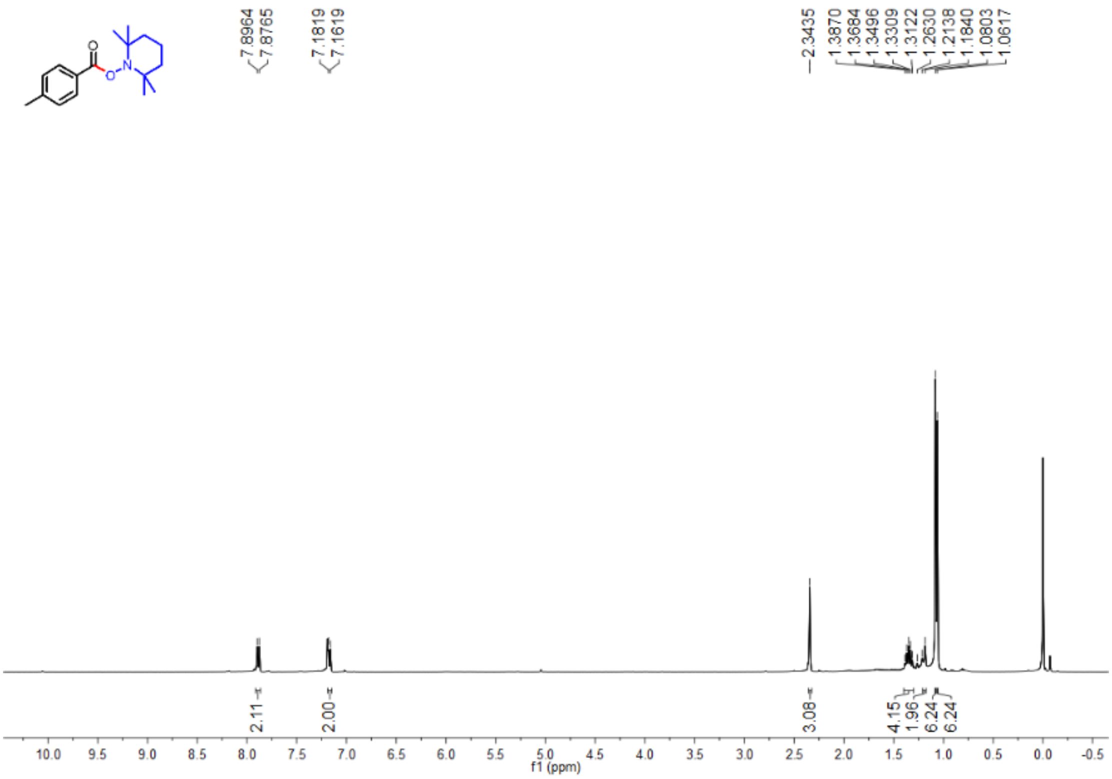

Supplementary Figure 126 ¹H NMR (400 MHz) spectrum of compound **58** in $CDCl_3$

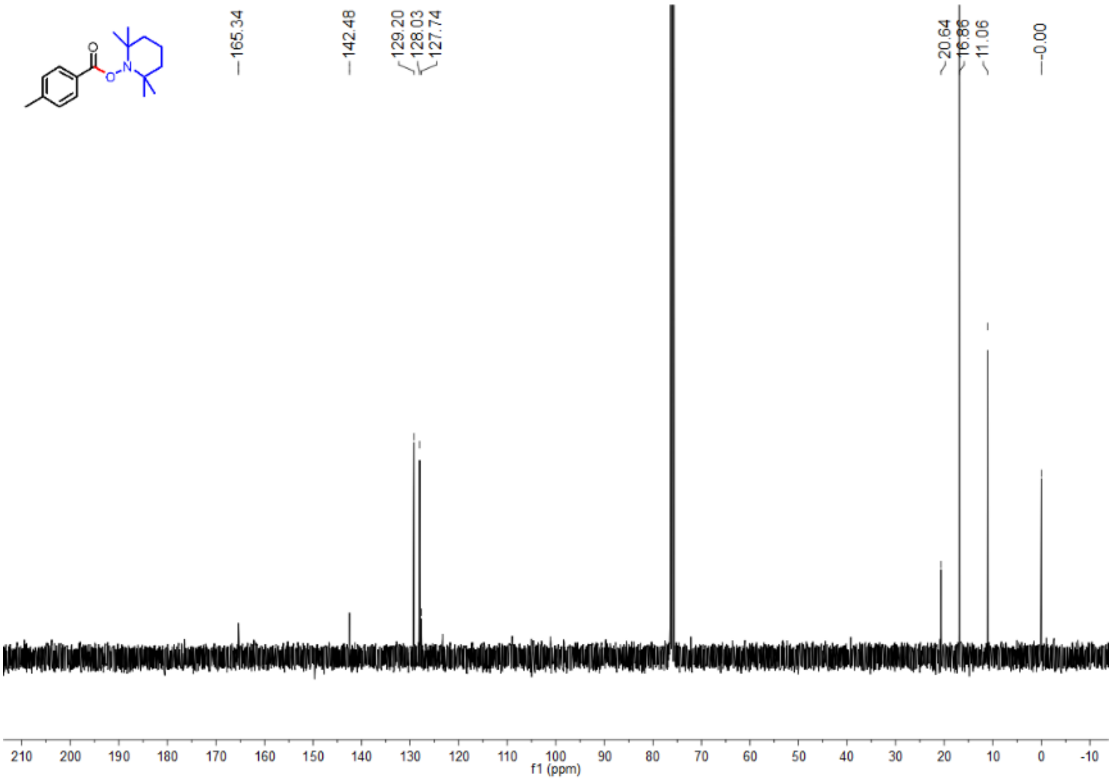

Supplementary Figure 127 ¹³C NMR (100 MHz) spectrum of compound **58** in $CDCl_3$

4. Supplementary References

[1] Bryden, M. A., & Zysman-Colman, E. Organic thermally activated delayed fluorescence (TADF) compounds used in photocatalysis. *Chem. Soc. Rev*. **50**, 7587–7680 (2021)

[2] Ishii, T., Kakeno, Y., Nagao, K., & Ohmiya, H. N-Heterocyclic Carbene-Catalyzed Decarboxylative Alkylation of Aldehydes. *J. Am. Chem. Soc.* **141**, 3854−3858 (2019).

[3] Pan, X., Lacôte, E., Lalevée, Jacques., & Curran D. P. Polarity Reversal Catalysis in Radical Reductions of Halides by N-Heterocyclic Carbene Boranes. *J. Am. Chem. Soc.* **134**, 5669–5674. (2012**)**

[4] Long, Y., Zheng, Y., Xia, Y., Qu, L., Yang, Y., Xiang, H., & Zhou, X. Nickel-Catalyzed Synthesis of an Aryl Nitrile via Aryl Exchange between an Aromatic Amide and a Simple Nitrile. *ACS Catal.* **12**, 4688–4695 (2022**)**.

[5] Tian, J., Luo, F., Zhang, C., Huang, X., Zhang, Y., Zhang, L., Kong, L., Hu, X., Wang, Z.-X., & Peng, B. Selective ortho C−H Cyanoalkylation of (Diacetoxyiodo)arenes through [3,3]-Sigmatropic Rearrangement. *Angew. Chem. Int. Ed.* **57**, 9078–9082 (2018).

[6] Zhang, M,. Yuan, X.-A., Zhu, C., & Xie, J. Deoxygenative Deuteration of Carboxylic Acids with D_2O. *Angew. Chem. Int. Ed.* **58**, 312–316 (2019).

[7] Triandafillidi, I., & Kokotos, C. G. Green Organocatalytic Synthesis of Isoxazolines via a One-Pot Oxidation of Allyloximes. *Org. Lett*. **19**, 106–109 (2017).

[8] Li, Z., Qian, L., Chen, H., & Xu, X. Generation of Iminoxyl Radicals by Photoredox Catalysis Enables Oxidant-Free Hydroxygenation of β,γ-Unsaturated Oximes. *Synlett*. **33**, 293–295 (2022).

[9] Yuan, Y., Zhang, X., Qian, H., & Ma, S. Catalytic enantioselective allene–anhydride approach to β,γ-unsaturated enones bearing an α-all-carbon-quarternary center. *Chem. Sci.* **11**, 9115–9121 (2020).

[10] Skucas, E., & MacMillan, D. W. C. Enantioselective α-Vinylation of Aldehydes via the Synergistic Combination of Copper and Amine Catalysis. *J. Am. Chem. Soc.* **134**, 9090–9093 (2012).

[11] Xu, G., Xiao, T., Feng, G., Liu, C., Zhang, B., & Xu, P. Metal-Free α-C(sp^3)–H Aroylation of Amines via a Photoredox Catalytic Radical–Radical Cross-Coupling Process. *Org. Lett.* **23**, 2846–2852 (2021).

[12] Liu, H., Han, Y.-F., Gao, Z.-H., Zhang, C.-L., Wang, C., & Ye, S. Cooperative N-Heterocyclic Carbene/Nickel-Catalyzed Hydroacylation of 1,3-Dienes with Aldehydes in Water. *ACS Catal.* **12**, 1657–1663 (2022).

[13] Trongsiriwat, N., Li, M., Pascual-Escudero, A., Yucel, B., & Walsh, P. J. Palladium-Catalyzed Allylic Alkylation of 2-Aryl-1,3-Dithianes, an Umpolung Synthesis of β,γ-Unsaturated Ketones. *Adv. Synth. Catal.* **361**, 502–509 (2019).

[14] Sämann, C., & Knochel, P. A Convenient Synthesis of α-Substituted β,γ-Unsaturated Ketones and Esters via the Direct Addition of Substituted Allylic Zinc Reagents Prepared by Direct Insertion. *Synthesis* **45**, 1870–1876 (2013).

[15] Moss, M., Han, X., & Ready, J. M. Zirconocene-Mediated Carbonylative Coupling of Grignard Reagents. *Angew. Chem. Int. Ed.* **55**, 10017–10021 (2016).

[16] Ellwart, M., & Knochel, P. Preparation of Solid, Substituted Allylic Zinc Reagents and Their Reactions with Electrophiles. *Angew. Chem. Int. Ed.*, **54**, 10662–10665 (2015).

[17] Kou, X., Shao, Q., Ye, C., Yang, G., & Zhang, W. Asymmetric Aza-Wacker-Type Cyclization of N-Ts Hydrazine-Tethered Tetrasubstituted Olefins: Synthesis of Pyrazolines Bearing One Quaternary or Two Vicinal Stereocenters. *J. Am. Chem. Soc.* **140**, 7587–9597 (2018).

[18] Morack, T., Onneken, C., Nakakohara, H., Mück-Lichtenfeld, C., & Gilmour, R. Enantiodivergent Prenylation via Deconjugative Isomerization. *ACS Catalysis.* **11**, 11929–11937 (2021).
[19] Ma, W., Xue, D., Yu, T., Wang, C., & Xiao, J. Carbonylative coupling of allylic acetates with aryl boronic acids. *Chem. Commun.* **51**, 8797–8800 (2015).
[20] Shu, B., Wang, X.-T., Shen, Z.-X., Che, T., Zhong, M., Song, J.-L., Kang, H., Xie, H., Zhang, L., & Zhang, S.-S. Iridium-catalyzed arylation of sulfoxonium ylides and arylboronic acids: a straightforward preparation of α-aryl ketones. *Org. Chem. Front.* **7**, 1802–1808 (2020).
[21] Churruca, F., SanMartin, R., Carril, M., Tellitu, I., & Domínguez, E. Towards a facile synthesis of triarylethanones: palladium-catalyzed arylation of ketone enolates under homogeneous and heterogeneous conditions. *Tetrahedron* **60**, 2393–2408 (2004).
[22] Zeysing, B., Gosch, C., & Terfort, A. Protecting Groups for Thiols Suitable for Suzuki Conditions. *Org. Lett.* **2**, 1843–1845 (2000).
[23] Wang, C.-A., Rahman, M., Bisz, E., Dziuk, B., Szostak, R., & Szostak, M. Palladium-NHC (NHC = N-heterocyclic Carbene)-Catalyzed Suzuki–Miyaura Cross-Coupling of Alkyl Amides. *ACS Catal.* **12**, 2426–2433 (2022)
[24] Liu, X.-Y., Cheng, B.-Q., Guo, Y.-C., Chu, X.-Q., Li, Y.-X., Loh, T.-P., & Shen, Z.-L. Bismuth-Mediated Diastereoselective Allylation Reaction of Carbonyl Compounds with Cyclic Allylic Halides or Cinnamyl Halide. *Adv. Synth. Catal.* **361**, 542–549 (2019).
